# Mechanistic insights on the Pd-catalyzed addition of C–X bonds across alkynes – a combined experimental and computational study†
†Electronic supplementary information (ESI) available: Computational details, cartesian coordinates of calculated species, experimental procedures, and spectroscopic data are given. See DOI: 10.1039/c6sc05001h



**DOI:** 10.1039/c6sc05001h

**Published:** 2017-01-27

**Authors:** Theresa Sperger, Christine M. Le, Mark Lautens, Franziska Schoenebeck

**Affiliations:** a RWTH Aachen University , Institute of Organic Chemistry , Landoltweg 1 , 52074 Aachen , Germany . Email: franziska.schoenebeck@rwth-aachen.de; b University of Toronto , Davenport Laboratories , Department of Chemistry , 80 St. George Street , Toronto , Ontario M5S 3H6 , Canada . Email: mlautens@chem.utoronto.ca

## Abstract

The Pd-catalyzed intramolecular addition of carbamoyl chlorides and aryl halides across alkynes is investigated by means of DFT calculations and mechanistic test experiments. The data suggest a mechanistic pathway that involves oxidative addition, alkyne insertion, *cis* → *trans* isomerization and reductive elimination. Our data indicate that oxidative addition is the reactivity limiting step in the addition of aryl chlorides and bromides across alkynes. However, for the corresponding addition of carbamoyl chlorides, alkyne insertion is found to be limiting. Full energetic reaction pathways for the intramolecular additions across alkynes are presented herein and the role of ligands, alkyne substituents and tether moieties are discussed. Notably, the calculations could rationalize a pronounced effect of the alkyne substituent, which accounts for the exceptional reactivity of TIPS-substituted alkynes. In particular, the bulky silyl moiety is shown to significantly destabilize the formed Pd(ii)-intermediates, thus facilitating both *cis* → *trans* isomerization and reductive elimination, which overall results in a flatter energetic landscape and a therefore increased catalytic efficiency.

## Abstract 2

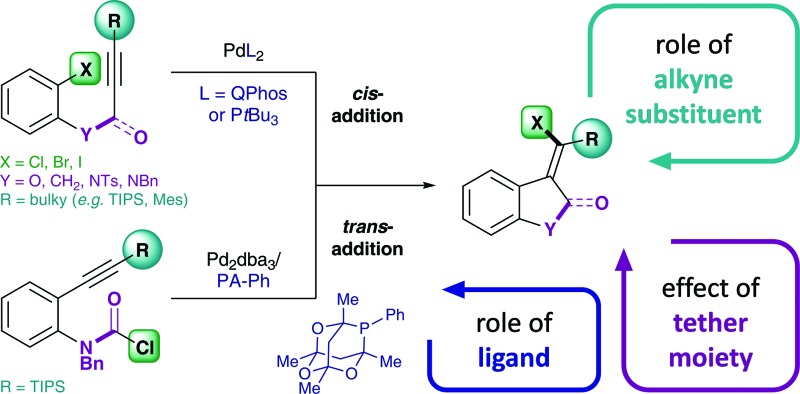
A mechanistic study of the Pd-catalyzed intramolecular addition of carbamoyl chlorides and aryl halides across alkynes is presented.

## Introduction

Methylene oxindoles are a highly relevant motif in many biologically active molecules and natural products.^1–8^ Despite their use in medicinal chemistry and natural product synthesis, methods to access methylene oxindoles in a highly stereoselective manner are limited.^9–15^ In this context, Lautens and co-workers have developed the synthesis of methylene oxindoles *via* Pd(0)-catalyzed carbohalogenation of alkynes, which involves an unusual and remarkable reductive elimination of C(sp^2^)–X (X = I, Br, Cl) from Pd(ii) as key step.^16^

**Scheme 1:**
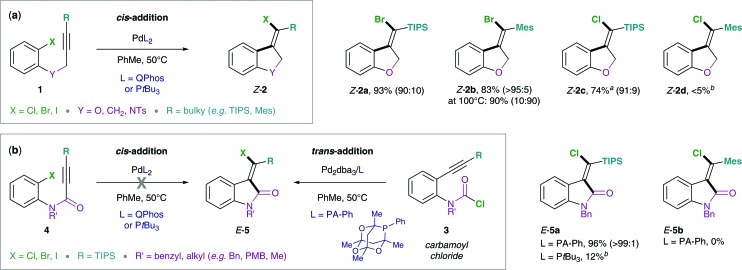
Experimental data by Lautens and co-workers: (a) intramolecular addition of aryl halides (**1**) across alkynes,^16^ (b) intramolecular addition of carbamoyl chlorides (**3**) across alkynes.^15^ ^*a*^Reaction was performed at 110 °C using 1,2,2,6,6-pentamethylpiperidine (PMP, 0.25 equiv.) as an additive.^16,33^ ^*b*^Yield was determined by ^1^H NMR analysis of the crude reaction mixture using 1,3,5-trimethoxybenzene as an internal standard.

While oxidative additions of aryl and vinyl halides to Pd(0) are widely studied and a relatively well understood step in Pd-catalysis, the corresponding back reaction, *i.e.* the reductive elimination of C–X bonds from Pd(ii) has had significantly less precedence.^17^ A reason for this is that reductive elimination from Pd(ii) is generally slow and disfavored. Therefore, chemists have turned to alternative protocols, *e.g.* oxidatively accessing the reductive elimination from higher oxidation state Pd intermediates, such as Pd(iii) and Pd(iv).^18–27^ However, C–X bond formation *via* reductive elimination from Pd(ii) may become feasible under sterically demanding conditions (bulky ligands and substituents) and if potential side-reactions are suppressed (substrate control, *e.g.* avoiding the β-H elimination).^28–32^ Advances in substrate design as well as catalyst development by the Lautens group recently allowed for the reductive elimination of C(sp^2^)–X (X = I, Br, Cl) from Pd(ii) in the carbohalogenation of alkynes (Scheme 1).^16^ Interestingly, the synthetic protocol was compatible with ether, alkyl and amine tethers (Scheme 1, **1**), but the use of an amide tether (**4**) required an alternative synthetic route *via* the chlorocarbamoylation of alkynes.^15^ This complementary synthesis utilizes the reactivity of carbamoyl chlorides, a relatively underexplored class of substrates. In contrast to the original synthetic method which employs aryl halides (**1**) in combination with bulky phosphines, such as Q-Phos and P*t*Bu_3_, the use of carbamoyl chlorides (**3**) required a less bulky aryl phosphaadamantane ligand (PA-Ph = 1,3,5,7-tetramethyl-2,4,8-trioxa-6-phenyl-6-phosphaadamantane) in order to reach good conversions. Furthermore, the chlorocarbamoylation reaction (Scheme 1b) exclusively allows for a bulky tri-iso-propylsilyl (TIPS) alkyne substituent, but is not effective using less bulky silyls or bulky aryl moieties (such as Mes = mesityl). This is in contrast to the carbohalogenation reaction (Scheme 1a) that displays good yields for a number of aryl halides with either bulky silyl or aryl alkyne substituents (*e.g.* TIPS, TBS = *tert*-butyldimethylsilyl, Mes, 1-naphthyl, 9-anthracenyl).

Herein we present a combined computational and experimental study in an effort to understand the underlying mechanism of these transformations and its implication on reactivity. More specifically, we aim to shed light on selectivity- and reactivity-controlling factors of ligand and substrate and the implicit requirements on alkyne substituent as well phosphine ligand. We hope these results will aid future substrate and catalyst design to further expand the scope of these atom-economic and synthetically relevant Pd-catalyzed intramolecular transformations.

## Computational methods

DFT calculations were performed using Gaussian 09, Revision D.01.^34^ Geometry optimizations and frequency calculations were conducted in the gas-phase at the B3LYP/6-31G(d) level of theory, employing LANL2DZ as an ECP for Pd. All stationary points were verified as either minima or transition states. Additionally, transition states (TSs) were confirmed by following the intrinsic reaction coordinate (IRC) to the corresponding intermediates. Energies were calculated at the M06L/def2-TZVP level of theory, employing the CPCM solvation model to account for toluene as the solvent.^35^ All energies were converted to 1 M standard state.

## Results and discussion

### General mechanism

**Scheme 2:**
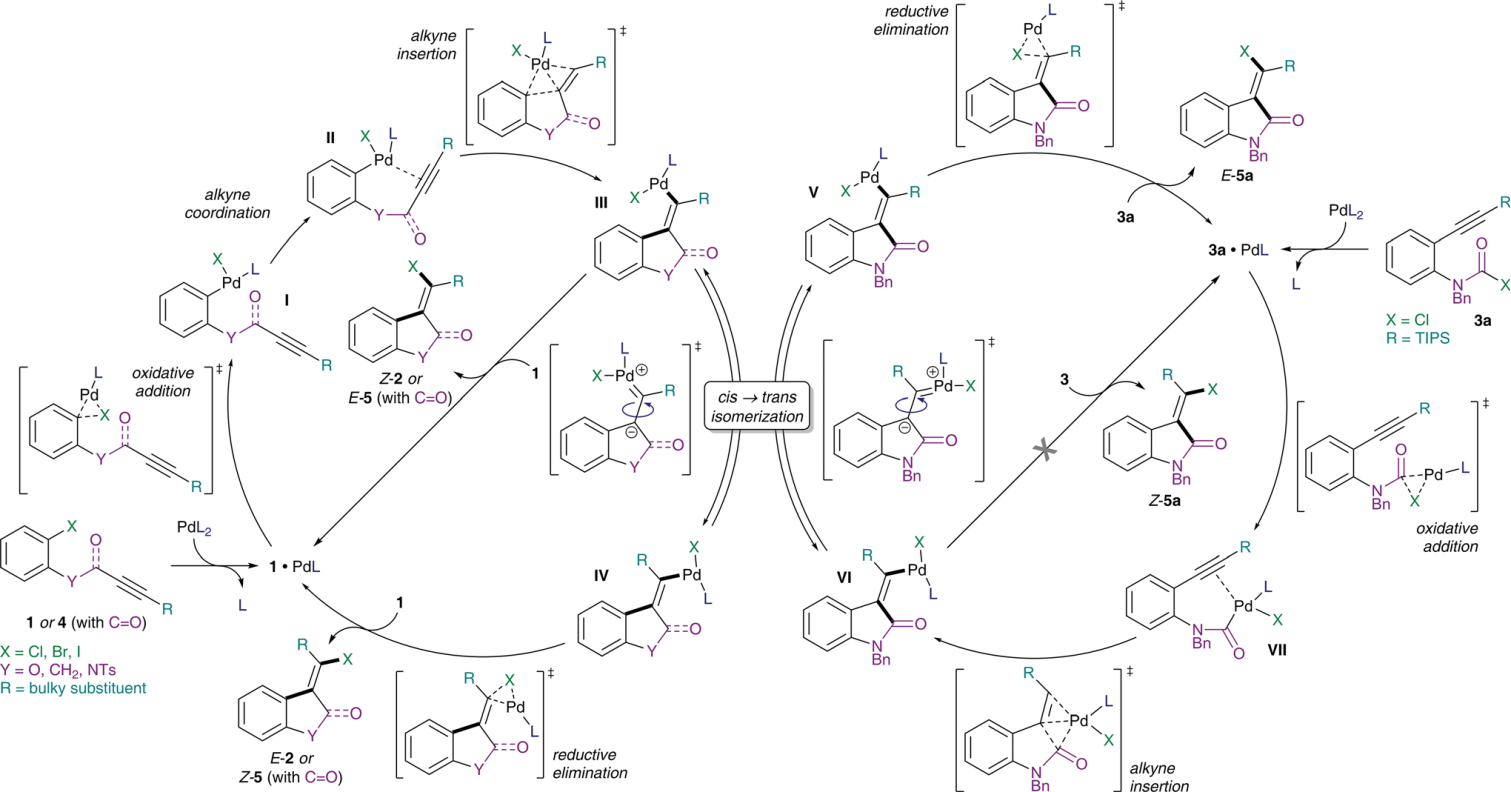
Proposed mechanisms for the addition of aryl halides (**1** and **4**, left) and carbamoyl chlorides (**3**, right) across alkynes.

The proposed mechanism of the intramolecular addition of aryl halides and carbamoyl chlorides is shown in Scheme 2. On the basis of our calculations, we propose that initial oxidative addition of either aryl halide **1** or carbamoyl chloride **3** to monophosphine Pd(0) is followed by insertion of the alkyne, *i.e. cis*-carbopalladation. Direct reductive elimination (for aryl halide substrates **1**) or rapid *cis* → *trans* isomerization and successive reductive elimination (in the case of carbamoyl chloride substrates **3**) then yields the observed methylene oxindole products *Z*-**2** and *E*-**5**, respectively.

#### Origin of the superior reactivity of carbamoyl chlorides over aryl chlorides

Our calculations on the intramolecular addition of C(sp^2^)–X (X = Br, Cl) across alkynes indicate that oxidative addition of aryl halide **1** or **4** is the elementary step with the highest activation barrier (Schemes 2 and 4, left). By contrast, the corresponding intramolecular addition of aryl halides across alkenes proceeds with reductive elimination of the C(sp^3^)–X bond (X = I, Br, Cl) as the rate-determining step.^36,37^ In the case of the addition of carbamoyl chlorides across alkynes (see Schemes 2 and 4, right), oxidative addition was found to be the TS with the highest activation barrier when a concerted 3-membered TS geometry was considered.

However, while oxidative additions to aryl halides have been subject to extensive computational and mechanistic studies,^38–45^ little is known on the nature of the transition state for reactions with carbamoyl chlorides. The high electrophilicity of these species may imply an ionic/electron transfer or formal nucleophilic substitution reaction. By means of computations it is challenging to unambiguously distinguish between these charged and neutral pathways due to the applied computational approximations.^46,47^

**Scheme 3:**
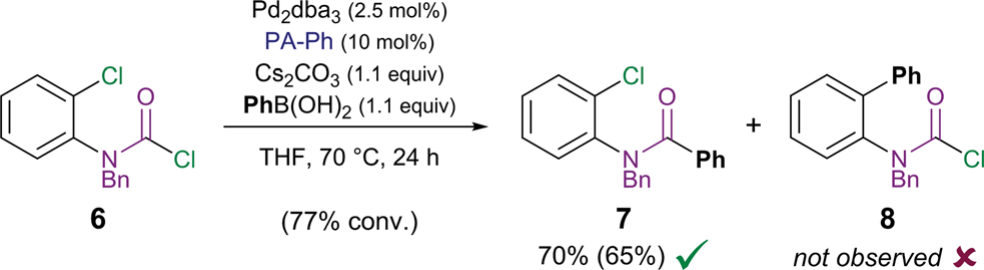
Competitive Suzuki cross-coupling of aryl *versus* carbamoyl chloride (isolated yield shown in parenthesis).

We therefore designed a test experiment and performed a competitive Suzuki cross-coupling using substrate **6**, possessing both aryl chloride and carbamoyl chloride moieties (Scheme 3).^48^ An exclusive activation of carbamoyl chloride over aryl chloride was observed experimentally.^49^

**Scheme 4:**
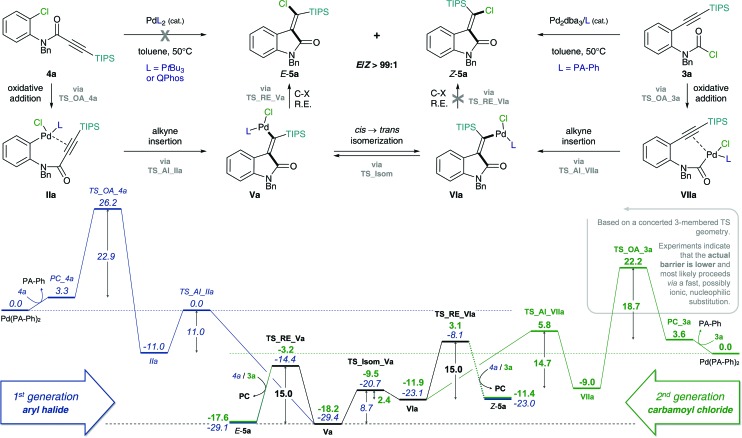
Calculated Gibbs free energy pathways of first and second generation substrates **3a** and **4a** possessing an amide tether. Energies (in kcal mol^–1^) were calculated at the CPCM (toluene) M06L/def2-TZVP//B3LYP/6-31G(d)(LANL2DZ) level of theory. Abbreviations: oxidative addition (OA), alkyne insertion (AI), *cis* → *trans* isomerization (Isom) and reductive elimination (RE).

However, despite the clear preference for oxidative addition of carbamoyl chloride over aryl chloride, the arguably fast oxidative addition step (*i.e.* fast compared to oxidative addition to aryl chloride) may still be the rate-determining TS of the reaction. Therefore, a resting state analysis of the reaction of **3a** and **4a** with catalytic Pd/PA-Ph was performed.^48^ Almost instantaneous conversion of carbamoyl chloride **3a** with concomitant formation of new phosphine-containing species and free ligand was observed by ^31^P NMR. In contrast, aryl chloride **4a** only yielded traces of product under the same reaction conditions and only one major P-containing species was observed, which is most likely the result of catalyst decomposition. In addition, two species (6.8 and 6.7 ppm by ^31^P NMR) were observed for both substrates and are likely to be *cis*- and *trans*-Pd(ii) intermediates, **Va** and **VIa** for substrate **3a** as well as **IIIa** and **IVa** for substrate **4a**, respectively. Furthermore, in the reaction of carbamoyl chloride **3a**, a species at 5.2 ppm in the ^31^P NMR spectrum was formed, which decreased over time and might be the oxidative addition intermediate **VIIa**. With this information in hand, oxidative addition of carbamoyl chloride **3a** is unlikely to be the elementary step with the highest activation barrier. Instead, the observation of potential oxidative addition intermediate **VIIa** (species at 5.2 ppm in ^31^P NMR) suggests alkyne insertion, *i.e.* carbopalladation to be the turnover-determining transition state (TDTS). Thus, oxidative addition of carbamoyl chloride **3a** is suspected to proceed *via* a fast, possibly ionic, nucleophilic substitution TS rather than a concerted, 3-membered oxidative addition TS.^50^ Based on the experimental results, we assume oxidative addition of **3a** to be fast and alkyne insertion to be the TDTS for the addition of carbamoyl chlorides across alkynes. Since catalytic turnover depends on both activation barriers (Δ*G* ^‡^) and driving force (*i.e.* reaction free energy, Δ_r_ *G*) of the reaction, the free energy difference between the rate-determining intermediate (TDI) and transition state (TDTS), commonly referred to as the energetic span (δ*E*),^51,52^ determines the efficiency and speed of the catalytic cycle. Therefore, in order to assess and compare the reactivities of carbamoyl and aryl chlorides in their addition reactions across alkynes, full reaction pathways and their corresponding energetic spans were calculated (Scheme 4).

Table 1 shows the calculated energetic spans for the addition of aryl halides (TDTS = oxidative addition) and carbamoyl chlorides (TDTS = alkyne insertion) across alkynes. Further analysis of calculated Gibbs free energy pathways and energetic spans of substrates **3** and **4** revealed insights on the effects of ligand, halide and alkyne substituent on the reaction outcome – the results of which are discussed in detail in the following sections.

**Table 1:** Energetic spans^*a*^ and experimental yields of alkyne carbohalogenation and carbamoylation of substrates **3** and **4** bearing an amide tether moiety

Entry	Ligand	Substrate	X	R	Δ_r_ *G*	δ*E*	Yield of 5 (ref. 15)
1	P*t*Bu_3_	**4a**	Cl	TIPS	–21.9	28.9	0% (16%)^*b*^
2	P*t*Bu_3_	**4b**	Cl	Mes	–15.5	45.0	—
3	PA-Ph	**4a**	Cl	TIPS	–25.8	29.8	—
4	PA-Ph	**3a**	Cl	TIPS	–14.0	10.0	99%
5	P*t*Bu_3_	**3a**	Cl	TIPS	–10.8	16.4	12%
6	P*t*Bu_3_	**3b**	Cl	Mes	–2.9	38.9	0%

^*a*^Energetic spans (in kcal mol^–1^) were calculated according to δ*E* = *G*(TDTS) – *G*(TDI) + Δ_r_ *G* (with *G*(TDTS) = Gibbs free energy of turnover-determining TS, *G*(TDI) = Gibbs free energy of turnover-determining intermediate, Δ_r_ *G* = Gibbs free energy of reaction), at the CPCM (toluene) M06L/def2-TZVP//B3LYP/6-31G(d)(LANL2DZ) level of theory.

^*b*^Reaction was conducted using stoichiometric amounts of Pd and was heated at 100 °C for 24 h.

#### Effect of tether moiety

While the developed synthetic protocol for alkyne carbohalogenation was compatible with ether, alkyl and amine tethers (**1**, Y = O, CH_2_, NTs), employing amide substrates (**4**) only yielded trace amounts of methylene oxindole product **5**.^15^ To address the underlying reasons for the incompatibility of the amide tether, DFT calculations were combined with stoichiometric studies.

**Fig. 1:**
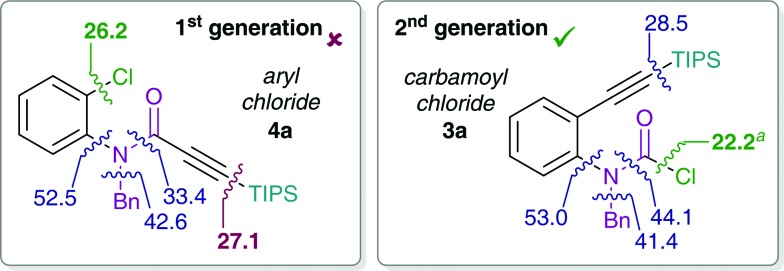
Activation free energies for insertion of Pd(PA-Ph) to potentially activated bonds of substrates **4a** (left) and **3a** (right). Values (in kcal mol^–1^) refer to Gibbs free energies calculated at the CPCM (toluene) M06L/def2-TZVP//B3LYP/6-31G(d)(LANL2DZ) level of theory. ^*a*^Calculated barrier for oxidative addition of carbamoyl chloride *via* a concerted 3-membered TS. Experiments however indicate that the actual barrier is lower and most likely proceeds *via* a fast, possibly ionic, nucleophilic substitution.

Stoichiometric studies of **4a** (R = TIPS) employing Pd(P*t*Bu_3_)_2_ showed no conversion at 50 °C, but when heated at 100 °C for 24 h yielded 16% of *E*-**5a**, along with 64% of recovered starting aryl chloride **4a**. This result indicates that reaction of **4a** to form **5a** is possible, at least in a stoichiometric manner. However, the higher reaction temperatures lead to catalyst decomposition and thus prevent a catalytic reaction.^52^ The observed decomposition of catalyst may be facilitated by the amide moiety. Hence, possible deactivation/side reaction pathways have been investigated computationally (Fig. 1). More specifically, transition states for the oxidative insertion of Pd(PA-Ph) to potentially activated bonds of substrates **3a** and **4a** were calculated and the corresponding activation barriers compared. In the case of the second generation carbamoyl chloride substrate **3a**, all bond activations are of higher barrier than oxidative addition *via* a concerted, 3-membered TS. Notably, this is despite the fact that experiments indicate an even lower barrier for oxidative addition to the carbamoyl chloride *via* a rapid, possibly ionic, nucleophilic substitution (*vide supra*). In contrast, for the first generation aryl chloride substrate **4a**, which did not react under analogous reaction conditions, calculations suggest that an activation of the C–Si bond of the alkyne is competing with oxidative addition to the C–Cl bond. This observation is most likely due to an increase in reactivity of the C–Si bond as a result of the conjugation of the alkyne with the amide.

This activation of the alkyne substituent is not present for all other tether moieties (amine, ether, alkyl), therefore providing a potential explanation for the observed difference in reactivity.

#### Effect of phosphine ligand

**Scheme 5:**
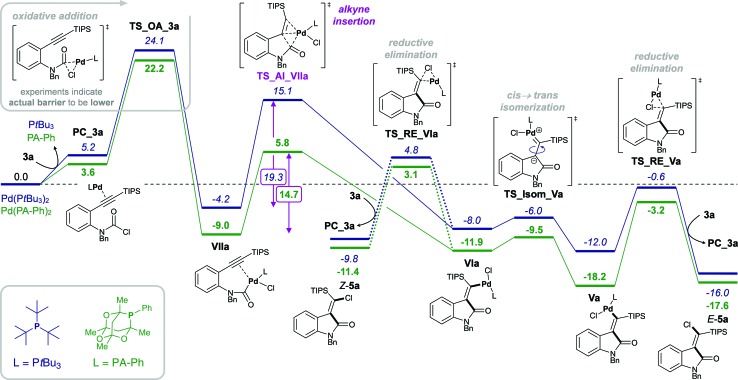
Ligand effect on alkyne chlorocarbamoylation of **3a**: calculated Gibbs free energy pathways employing L = P*t*Bu_3_ (blue, italics) and L = PA-Ph (green, bold). Energies (in kcal mol^–1^) were calculated at the CPCM (toluene) M06L/def2-TZVP//B3LYP/6-31G(d)(LANL2DZ) level of theory.

While bulky, electron-rich phosphine ligands such as P*t*Bu_3_ and QPhos were well-suited for the addition of aryl halides across alkenes^36,37^ and alkynes^16^ (*vide infra*), their use in the addition of carbamoyl chlorides across alkynes only led to small amounts of product being formed. This might be due to the change in TDTS. While oxidative addition is the rate-determining TS for the first generation aryl halide substrate **4**, alkyne insertion was considered to be the TDTS for the second generation carbamoyl chloride substrate **3**. In the case of aryl halide substrate **4**, the bulky P*t*Bu_3_ ligand facilitates the reductive elimination of product (Scheme 5), thereby reducing the Gibbs free energy of the reaction (Δ_r_ *G*) compared to the corresponding reaction of **4** with the less bulky PA-Ph ligand (Table 1, entries 1 and 3). In contrast, in the case of carbamoyl chloride **3**, the less bulky PA-Ph facilitates the presumed rate-determining alkyne insertion and lowers its activation by approximately 5 kcal mol^–1^ compared to the corresponding process employing the bulkier P*t*Bu_3_ ligand (Scheme 5). This directly causes a significant decrease in energetic span (Table 1, entry 4), thus explaining the superior reactivity observed for PA-Ph in comparison to the bulkier P*t*Bu_3_ ligand (entry 5).

### Silyl effect

**Scheme 6:**
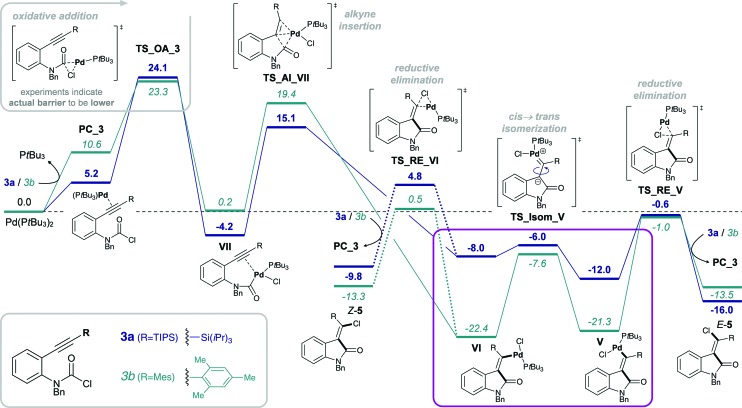
Effect of silyl substituent on the alkyne: calculated Gibbs free energy pathways of **3a** (R = TIPS, blue, bold) and **3b** (R = Mes, teal, italics) at the CPCM (toluene) M06L/def2-TZVP//B3LYP/6-31G(d)(LANL2DZ) level of theory (energies are given in kcal mol^–1^).

Experimentally, only substrates bearing a silyl-substituent on the alkyne were reactive in the chlorocarbamoylation reaction, whereas substrates with a mesityl-substituted alkyne did not cyclize under analogous reaction conditions. Next, we investigated the role of the alkyne substituent computationally. A significant increase in energetic span for mesityl-substituted substrates **4b** and **3b** (Table 1, entries 2 and 6, respectively) was observed compared to substrates **4a** and **3a** bearing a TIPS-substituent (entries 1 and 5, respectively). When comparing the energetic pathways for substrates **3a** (R = TIPS) and **3b** (R = Mes), an effect of the TIPS-moiety was observed on (i) *cis* → *trans* isomerization and (ii) reductive elimination (Scheme 6). More specifically, the TIPS-group leads to a significant destabilization of Pd(ii) intermediates **VI** and **V**, along with a stabilization of the transition state for *cis* → *trans* isomerization, which causes a substantial decrease in the barrier of isomerization and overall results in a flatter energetic pathway and thus a smaller energetic span.

#### Steric effects of the silyl group

While for TIPS-substituted Pd(ii) intermediates, the *trans*-intermediate **Va** is more stable than its corresponding *cis*-intermediate **VIa**, the opposite preference is observed for the mesityl-substituted Pd(ii) intermediates, *i.e.* **VIb** is more stable than **Vb**. Overall, both Pd(ii) intermediates **VIa** and **Va** are significantly destabilized by the TIPS-moiety compared to the corresponding mesityl-substituted intermediates (**VIb** and **Vb**). However, the degree of destabilization is more pronounced for the *cis*-Pd(ii) intermediate **VIa**. This would be in agreement with an increased steric interaction of the TIPS moiety with the aryl group of the oxindole in **VIa** compared to **Va**. In contrast, the mesityl moiety can rotate away (into a side-on conformation), in which there is significantly less steric interaction, thus rendering the Pd-substituent the most sterically congesting moiety. Therefore, the preference of **VIb** over **Vb** appears to be due to a decreased steric interaction of PdCl(P*t*Bu_3_) with the aromatic backbone of the oxindole.^48^

#### Electronic effects of the silyl group

**Fig. 2:**
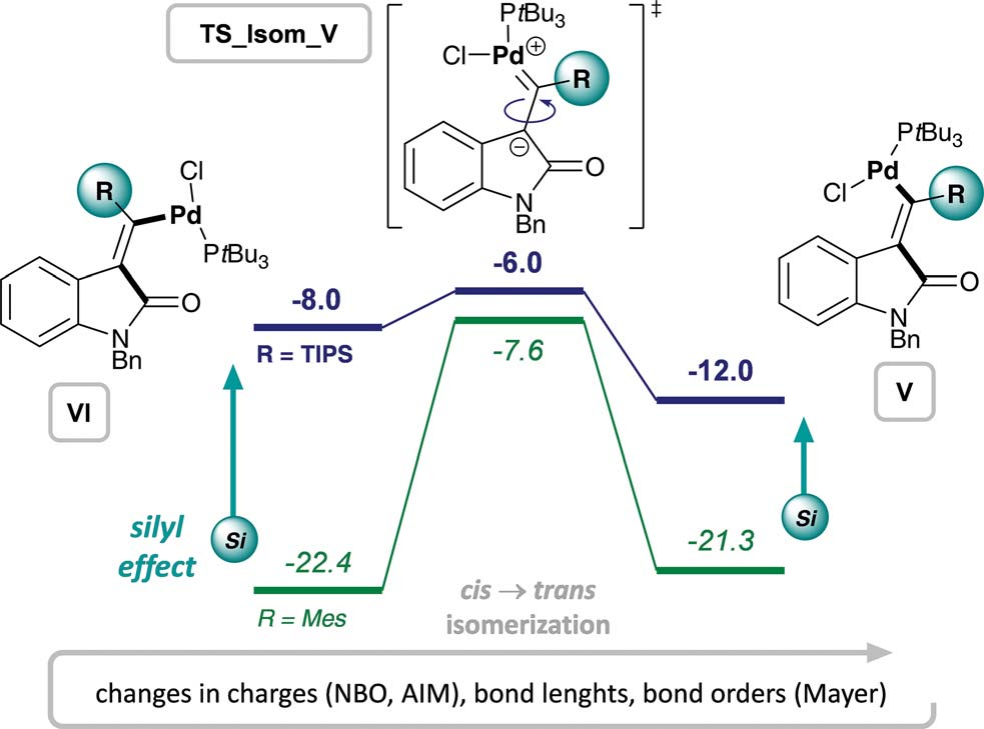
Effect of alkyne substituent on Pd(ii) intermediates, **VI** and **V**, and *cis* → *trans* isomerization: analysis of charges, bond lengths and bond orders.

In order to investigate the nature of the *cis* → *trans* isomerization TS, we analyzed (i) the charge distribution in the Pd(ii)-intermediates, **VIa** and **Va**, as well as during the isomerization TS and (ii) the change in bond lengths from *cis*-intermediate **VIa** *via* the TS to *trans*-intermediate **Va** (Fig. 2). For this reason, a natural bond order analysis (NBO analysis)^54–57^ was performed, which showed that only a minor change in charge separation occurs in the case of TIPS, whereas a significant buildup of charge separation takes place for R = Mes. More specifically, TIPS-substituted intermediates **VIa** and **Va** already possess a high degree of charge separation with a positive charge of +0.53 on the silyl substituent and only a minor increase of 4% leads to the isomerization TS (positive charge of +0.55 on the silyl moiety). In contrast, mesityl-substituted intermediates do not exhibit significant charge separation (+0.03 and +0.08 on mesityl for intermediates **VIb** and **Vb**, respectively) and a substantial separation of charges needs to be established (increase of 136%) in order to reach the charge-separated TS (with a positive charge of +0.16 on mesityl). This analysis is in line with calculated Atoms-In-Molecules (AIM)^58^ charges, which indicate essentially no change in charge on Si (increase of 0.2%) to reach the isomerization TS, but a strong increase in charge of 77% on the *ipso*-C of the mesityl-substituent in order to undergo isomerization.^48^ These results are congruent with the observed energetic destabilization of TIPS-substituted Pd(ii)-intermediates **VIa** and **Va** and indicate that the low barrier for *cis* → *trans* isomerization for R = TIPS is primarily a result of a destabilization of intermediates rather than a stabilization of the TS. Moreover, charge separation is much larger in the presence of the silyl substituent, suggesting that the TIPS-moiety can better stabilize charge buildup and thus lowers the barrier for the isomerization process.

This result is in agreement with the known effect of silyl groups to be able to stabilize carbocations in α,^59^ β^60,61^ and γ^62^ positions. In addition, analyzing the changes in bond lengths during the isomerization from *cis*- to *trans*-Pd(ii) intermediate (**VIa** to **Va**) shows a slight elongation of the double bond and concomitant shortening of both C–Si and C–Pd bonds in the TS, suggesting a delocalization of positive charge between Pd, C and Si.^48^ This corresponds with the calculated Mayer bond orders,^63,64^ which indicate a decrease in bond order of the C=C double bond during the TS for both R = TIPS and R = Mes, although more pronounced for the latter. At the same time, the C–R (R = TIPS or Mes) bond order increases during isomerization (indicating delocalization of charge into the R-substituent). This increase is only minor for the silyl moiety (increase of 8%), but more evident for mesityl (increase of 20%) as expected due to the bigger required changes in order to reach the TS.^48^

Overall, the silyl moiety exerts a combination of effects on several intermediates and steps in the chlorocarbamoylation reaction, giving rise to its unique reactivity.

### Origins of observed divergence in selectivity

In the reaction of carbamoyl chlorides an exclusive *E*-selectivity is reached by means of a rapid *cis* → *trans* isomerization (see Scheme 1b). However, in the corresponding reaction of aryl halides medium to good levels of *Z*-selectivity are observed as a result of direct reductive elimination. In the case of mesityl substituted alkynes, a switch in selectivity (*i.e.* to obtain *E*-**2b**) can be reached at elevated reaction temperatures (see Scheme 1a). Additionally, the substrate scope is not limited to TIPS-substituted alkynes, tolerating other bulky groups, such as aryls for example.

**Scheme 7:**
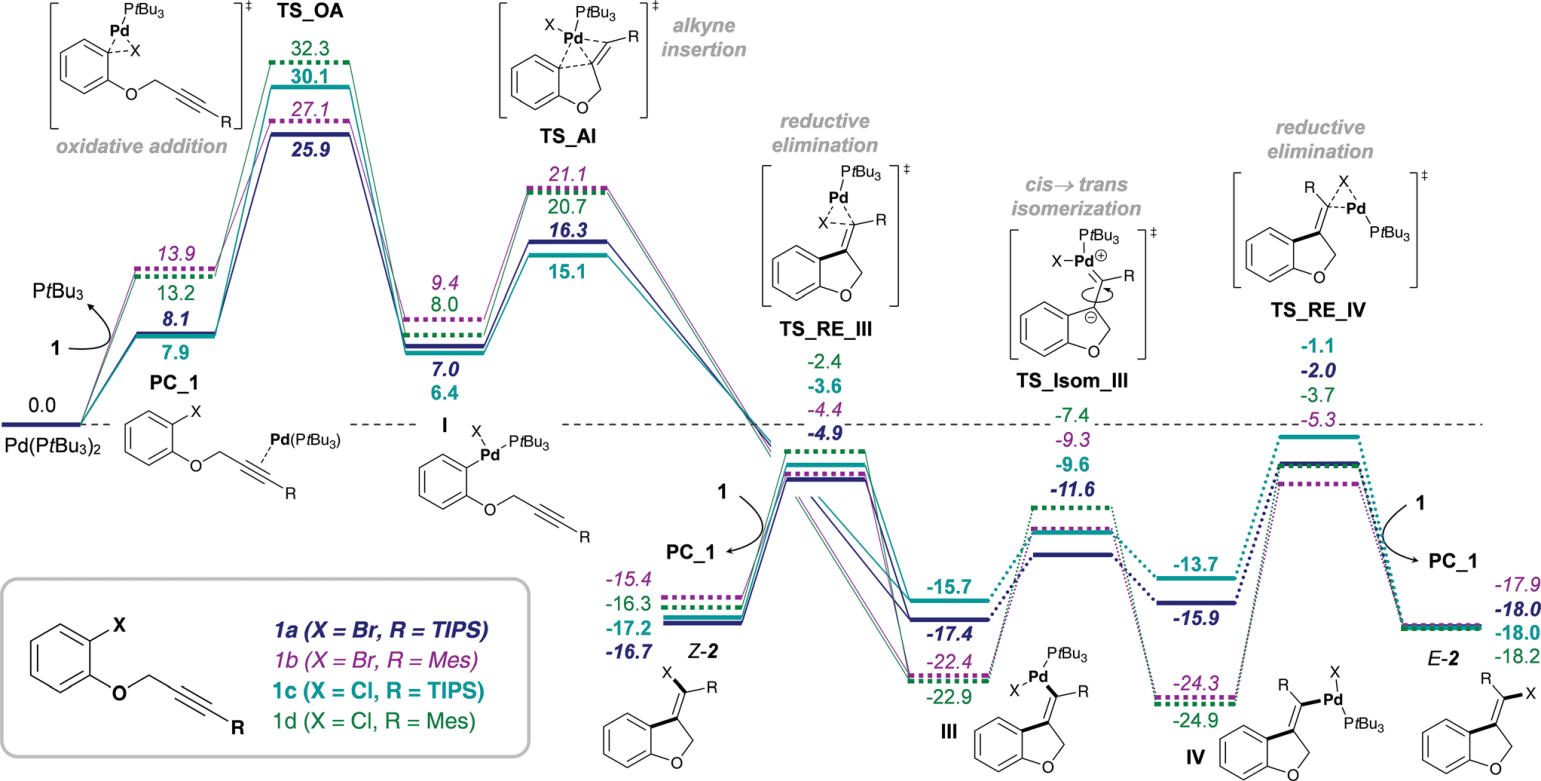
Gibbs free energy pathway of the Pd(P*t*Bu_3_)_2_-mediated alkyne carbohalogenation of **1a–d**, calculated at the CPCM (toluene) M06L/def2-TZVP//B3LYP/6-31G(d)(LANL2DZ) level of theory (values are given in kcal mol^–1^).

Analysis of the calculated free energy pathways revealed a pronounced effect of the alkyne substituent on the reactivity profile (Scheme 7). Analogous to reactions of the amide-tethered substrates **3** and **4** (Schemes 4–6), a TIPS-substituent favors the *cis*-Pd(ii) intermediate **IIIa**, but the corresponding *trans*-intermediate **IV** is more stable when possessing a mesityl-substituent (**IVb**). In addition, the barrier towards isomerization is significantly lower for R = TIPS compared to R = Mes, *i.e.* Δ*G* ^‡^ = 5.8 and 13.1 kcal mol^–1^ for substrates **1a** (R = TIPS) and **1b** (R = Mes), respectively. Moreover, the stability of the pre-complexes PC_1, where the active monophosphine Pd(0) (P*t*Bu_3_) species coordinates to the alkyne group of substrate **1**, is more pronounced when R = TIPS, indicating that the silyl group increases the electrophilic character of the alkyne moiety. This would suggest that the reductive elimination from the vinyl Pd(ii) intermediates, **III** and **IV**, is reversible for substrates **1b** and **1d** bearing a mesityl-substituent. In combination with the *trans*-Pd(ii) intermediate **IV** being more stable than the *cis*-intermediate **III**, a reversal of the observed *Z*-selectivity for R = Mes (at 50 °C) can be reached at elevated reaction temperatures (at 100 °C).^16,65^

## Conclusions

The mechanisms of alkyne carbohalogenation and chlorocarbamoylation have been investigated by means of DFT calculations and experiments. Catalytic pathways involving oxidative addition, alkyne insertion, *cis* → *trans* isomerization and reductive elimination are proposed. Oxidative addition is suggested to be reactivity limiting in the case of addition of aryl halides across alkynes: in the corresponding reaction of carbamoyl chlorides, oxidative addition was however shown to be fast and our data indicated that instead alkyne insertion, *i.e.* carbopalladation was reactivity limiting. Furthermore, the effects of halide, phosphine ligand and alkyne substituent on reactivity were investigated.

While the bulky P*t*Bu_3_ was vital for reactivity in the intramolecular addition of aryl halides across alkynes due to a lowering of the barriers for reductive elimination, the less bulky phosphaadamantane ligand PA-Ph is uniquely suited for the corresponding addition reaction of carbamoyl chlorides. Calculations indicate that this is due to a significant decrease in the barrier for the reactivity limiting alkyne insertion with the less bulky PA-Ph ligand compared to P*t*Bu_3_. Notably, a pronounced effect of the alkyne substituent on reactivity was unravelled, which accounts for the exceptional reactivity of substrates bearing a TIPS-substituent. More specifically, the bulky TIPS-group was shown to cause a significant destabilization of Pd(ii) intermediates **VI** and **V**, along with a stabilization of the *cis* → *trans* isomerization TS. This overall results in a smaller energetic span and thus significantly increases catalytic turnover.

## References

[cit1] Davis S. T., Dickerson S. H., Frye S. V., Harris P. A., Hunter R. N., Kuyper L. F., Lackey K. E., Luzzio M. J., Veal J. M. and Walker D. H., Patent WO9915500, 1999.

[cit2] Heckel A., Roth G. J., Kley J., Hoerer S. and Uphues I., Patent WO2005087727 A1, 2005.

[cit3] Hauf S., Cole R. W., LaTerra S., Zimmer C., Schnapp G., Walter R., Heckel A., van Meel J., Rieder C. L., Peters J.-M. (2003). J. Cell Biol..

[cit4] Grandinetti C. A., Goldspiel B. R. (2007). Pharmacotherapy.

[cit5] Hilberg F., Roth G. J., Krssak M., Kautschitsch S., Sommergruber W., Tontsch-Grunt U., Garin-Chesa P., Bader G., Zoephel A., Quant J., Heckel A., Rettig W. J. (2008). Cancer Res..

[cit6] Trost B. M., Cramer N., Bernsmann H. (2007). J. Am. Chem. Soc..

[cit7] Trost B. M., Cramer N., Silverman S. M. (2007). J. Am. Chem. Soc..

[cit8] Lin S., Danishefsky S. J. (2001). Angew. Chem., Int. Ed..

[cit9] Cantagrel G., de Carné-Carnavalet B., Meyer C., Cossy J. (2009). Org. Lett..

[cit10] Tang S., Yu Q.-F., Peng P., Li J.-H., Zhong P., Tang R.-Y. (2007). Org. Lett..

[cit11] Sassatelli M., Debiton É., Aboab B., Prudhomme M., Moreau P. (2006). Eur. J. Med. Chem..

[cit12] Pedras M. S. C., Sorensen J. L., Okanga F. I., Zaharia I. L. (1999). Bioorg. Med. Chem. Lett..

[cit13] Beccalli E. M., Marchesini A. (1995). Tetrahedron.

[cit14] Beccalli E. M., Marchesini A., Pilati T. (1994). Tetrahedron.

[cit15] Le C. M., Hou X., Sperger T., Schoenebeck F., Lautens M. (2015). Angew. Chem., Int. Ed..

[cit16] Le C. M., Menzies P. J. C., Petrone D. A., Lautens M. (2015). Angew. Chem., Int. Ed..

[cit17] Jiang X., Liu H., Gu Z. (2012). Asian J. Org. Chem..

[cit18] Higher Oxidation State Organopalladium and Platinum Chemistry, ed. A. J. Canty, Springer-Verlag, Berlin Heidelberg, 2011.

[cit19] Canty A. J. (1992). Acc. Chem. Res..

[cit20] Hickman A. J., Sanford M. S. (2012). Nature.

[cit21] Engle K. M., Mei T.-S., Wang X., Yu J.-Q. (2011). Angew. Chem., Int. Ed..

[cit22] Powers D. C., Ritter T. (2009). Nat. Chem..

[cit23] Powers D. C., Benitez D., Tkatchouk E., Goddard W. a., Ritter T. (2010). J. Am. Chem. Soc..

[cit24] Powers D. C., Lee E., Ariafard A., Sanford M. S., Yates B. F., Canty A. J., Ritter T. (2012). J. Am. Chem. Soc..

[cit25] Kalyani D., Dick A. R., Anani W. Q., Sanford M. S. (2006). Org. Lett..

[cit26] Stowers K. J., Sanford M. S. (2009). Org. Lett..

[cit27] Arnold P. L., Sanford M. S., Pearson S. M. (2009). J. Am. Chem. Soc..

[cit28] Roy A. H., Hartwig J. F. (2001). J. Am. Chem. Soc..

[cit29] Roy A. H., Hartwig J. F. (2003). J. Am. Chem. Soc..

[cit30] Roy A. H., Hartwig J. F. (2004). Organometallics.

[cit31] Watson D. A., Su M., Teverovskiy G., Zhang Y., García-Fortanet J., Kinzel T., Buchwald S. L. (2009). Science.

[cit32] Shen X., Hyde A. M., Buchwald S. L. (2010). J. Am. Chem. Soc..

[cit33] Petrone D. A., Yoon H., Weinstabl H., Lautens M. (2014). Angew. Chem., Int. Ed..

[cit34] Frisch M. J., Trucks G. W., Schlegel H. B., Scuseria G. E., Robb M. A., Cheeseman J. R., Scalmani G., Barone V., Mennucci B., Petersson G. A., Nakatsuji H., Caricato M., Li X., Hratchian H. P., Izmaylov A. F., Bloino J., Zheng G., Sonnenberg J. L., Hada M., Ehara M., Toyota K., Fukuda R., Hasegawa J., Ishida M., Nakajima T., Honda Y., Kitao O., Nakai H., Vreven T., Montgomery Jr J. A., Peralta J. E., Ogliaro F., Bearpark M., Heyd J. J., Brothers E., Kudin K. N., Staroverov V. N., Keith T., Kobayashi R., Normand J., Raghavachari K., Rendell A., Burant J. C., Iyengar S. S., Tomasi J., Cossi M., Rega N., Millam J. M., Klene M., Knox J. E., Cross J. B., Bakken V., Adamo C., Jaramillo J., Gomperts R., Stratmann R. E., Yazyev O., Austin A. J., Cammi R., Pomelli C., Ochterski J. W., Martin R. L., Morokuma K., Zakrzewski V. G., Voth G. A., Salvador P., Dannenberg J. J., Dapprich S., Daniels A. D., Farkas O., Foresman J. B., Ortiz J. V., Cioslowski J., and Fox D. J., Gaussian 09, Revision D.01, Gaussian, Inc., Wallingford CT, 2013.

[cit35] For appropriateness of computational method, see: Sperger T. Sanhueza I. A. Kalvet I. Schoenebeck F., Chem. Rev., 2015, 115 , 9532 –9586 . 26207572 10.1021/acs.chemrev.5b00163

[cit36] Lan Y., Liu P., Newman S. G., Lautens M., Houk K. N. (2012). Chem. Sci..

[cit37] Newman S. G., Lautens M. (2011). J. Am. Chem. Soc..

[cit38] McMullin C. L., Fey N., Harvey J. N. (2014). Dalton Trans..

[cit39] McMullin C. L., Jover J., Harvey J. N., Fey N. (2010). Dalton Trans..

[cit40] Vikse K., Naka T., McIndoe J. S., Besora M., Maseras F. (2013). ChemCatChem.

[cit41] Besora M., Gourlaouen C., Yates B., Maseras F. (2011). Dalton Trans..

[cit42] Barrios-Landeros F., Carrow B. P., Hartwig J. F. (2009). J. Am. Chem. Soc..

[cit43] Ahlquist M., Norrby P.-O. (2007). Organometallics.

[cit44] Ahlquist M., Fristrup P., Tanner D., Norrby P.-O. (2006). Organometallics.

[cit45] Senn H. M., Ziegler T. (2004). Organometallics.

[cit46] Sperger T., Fisher H. C., Schoenebeck F. (2016). Wiley Interdiscip. Rev.: Comput. Mol. Sci..

[cit47] Proutière F., Schoenebeck F. (2011). Angew. Chem., Int. Ed..

[cit48] Please see ESI for further details

[cit49] Calculations are in line with the experiment and predict a difference in activation barriers of oxidative addition of ΔΔ*G* ^‡^ = 8.3 kcal mol^–1^ in favor of carbamoyl chloride activation

[cit50] An ionic, nucleophilic addition TS could not be located owing to the difficulties associated with the description of charges, which represent a well-known challenge. For further information, please refer to ref. 46

[cit51] Amatore C., Jutand A. (1999). J. Organomet. Chem..

[cit52] Kozuch S., Shaik S. (2011). Acc. Chem. Res..

[cit53] Notably, catalyst decomposition was observed *via* ^31^P NMR, no Pd(PtBu_3_)_2_ or free PtBu_3_ were remaining after the reaction

[cit54] Foster J. P., Weinhold F. (1980). J. Am. Chem. Soc..

[cit55] Reed A. E., Weinstock R. B., Weinhold F. (1985). J. Chem. Phys..

[cit56] Reed A. E., Weinhold F. (1985). J. Chem. Phys..

[cit57] Reed A. E., Curtiss L. A., Weinhold F. (1988). Chem. Rev..

[cit58] Bader R. F. W., Atoms in Molecules: A Quantum Theory, Oxford University Press, New York, 1990.

[cit59] Kostenko A., Müller B., Kaufmann F.-P., Apeloig Y., Siehl H.-U. (2012). Eur. J. Org. Chem..

[cit60] Sproul K. C., Chalifoux W. A. (2015). Org. Lett..

[cit61] Nokami T., Yamane Y., Oshitani S., Kobayashi J.-k., Matsui S.-i., Nishihara T., Uno H., Hayase S., Itoh T. (2015). Org. Lett..

[cit62] Creary X., Kochly E. D. (2009). J. Org. Chem..

[cit63] Mayer I. (1983). Chem. Phys. Lett..

[cit64] Mayer I. (2012). Chem. Phys. Lett..

[cit65] Observedselectivities for substrate **1b** (X = Br, R = Mes), determined by ^1^H NMR analysis of the crude reaction mixture using 1,3,5-trimethoxybenzene as an internal standard after reaction for 18 h in toluene (0.1 m) employing 5 mol% Pd(QPhos)_2_: *Z*/*E* > 95 : 5 at 50 °C and 10 : 90 at 100 °C

