# Supplementary material for: Mechanistic insights on the Pd-catalyzed addition of C–X bonds across alkynes – a combined experimental and computational study
Source: Chem Sci. 2017 Jan 27;8(4):2914–22. doi: 10.1039/c6sc05001h (PMC5376711; doi:10.1039/c6sc05001h)
Supplement: Supplementary file 1 [file SC-008-C6SC05001H-s001.pdf]

Electronic Supplementary Material (ESI) for Chemical Science.
This journal is © The Royal Society of Chemistry 2017

Supporting Information

Mechanistic Insights on the Pd-Catalyzed Addition of C-X Bonds across Alkynes – A Combined Experimental and Computational Study

Theresa Sperger[a], Christine M. Le[b], Mark Lautens*[b] and Franziska Schoenebeck*[a]

[a] RWTH Aachen University, Institute of Organic Chemistry,
Landoltweg 1, 52074 Aachen, Germany.
E-mail: franziska.schoenebeck@rwth-aachen.de

[b] University of Toronto, Davenport Research Laboratories, Department of Chemistry,
80 St. George St., Toronto, Ontario M5S 3H6, Canada.
E-mail: mlautens@chem.utoronto.ca

Contents

1. Experimental Details S3
1.1. General considerations S3
1.2. Competitive Suzuki cross-coupling S3
1.3. Resting state analysis S4
2. Computational Details S6
2.1. Full reference for Gaussian 09 Revision D.01 .... S6
2.2. General computational details S6
2.3. Free energetic span S6
2.4. Analysis of steric and electronic influences of the silyl group S6
2.5. Full energetic pathways S9
2.6. XYZ Coordinates and Energies for Optimized Structures S14
3. References S94

1. Experimental Details

1.1. General considerations

Commercial reagents were purchased from Sigma Aldrich, Combi-Blocks, Strem or Alfa Aesar and used without further purification. Unless otherwise stated, all reactions were carried out under argon, whereas the work-up and isolation of the products were conducted on the bench-top using standard techniques. Tetrahydrofuran was distilled from sodium and benzophenone ketal, DCM was distilled over calcium hydride, and toluene was distilled over sodium. Pyridine was purchased from Sigma Aldrich and used without further purification. For the resting state NMR experiments, toluene-d_8 was used directly from a 1 mL ampule. Reactions were monitored by Thin Layer Chromatography (TLC) using EM Separations pre-coated silica gel 0.2 mm layer UV 254 fluorescent sheets, and visualization was accomplished with 250 nm UV light followed by immersion in $KMnO_4$ stain. Organic solutions were concentrated by rotary evaporation at reduced pressure (15 – 30 torr, house vacuum) at 25-50 °C. Unless otherwise stated, flash chromatography was performed using Ultra Pure 230-400 mesh silica gel purchased from Silicycle. NMR characterization data was collected at 296 K on a Varian Mercury 300, Varian Mercury 400, Agilent DD2 500, Agilent DD2 600, or a Bruker Advance III spectrometer operating at 300, 400, 500, or 600 MHz for ^{1}H NMR, and 75, 100, 125, or 150 MHz for ^{13}C NMR. ^{1}H NMR spectra were internally referenced to the residual solvent signal ($CDCl_3$ = 7.26 ppm) or TMS (0 ppm). ^{13}C NMR spectra were internally referenced to the residual solvent signal ($CDCl_3$ = 77.16 ppm) and are reported as observed. Data for ^{1}H NMR are reported as follows: chemical shift (δ ppm), multiplicity (s = singlet, d = doublet, t = triplet, q = quartet, m = multiplet, b = broad), coupling constant (Hz), integration. IR spectra were obtained on a PerkinElmer Spectrum 100 instrument equipped with a single-bounce diamond / ZnSe ATR accessory in the solid state. NMR yields for were obtained by ^{1}H NMR analysis of the crude reaction mixture using a 10 second relaxation delay and 1,3,5-trimethoxybenzene as an internal standard. High resolution mass spectra (HRMS) were obtained on an ABI/Sciex QStar Mass Spectrometer (ESI) or a JEOL AccuTOF medel JMS-T1000LC mass spectrometer equipped with an IONICS® Direct Analysis in real Time (DART) ion source at Advanced Instrumentation for Molecular Structure (AIMS) in the Department of Chemistry at the University of Toronto.

1.2. Competitive Suzuki cross-coupling

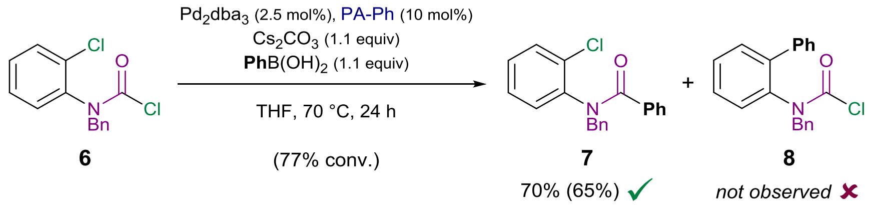

***N*-benzyl-*N*-(2-chlorophenyl)benzamide** (**7**): An oven-dried 2 mL dram vial was charged with Pd_2dba_3 (5.7 mg, 0.00625 mmol, 2.5 mol%), PA-Ph (7.3 mg, 0.025 mmol, 10 mol%), Cs_2CO_3 (89.6 mg, 0.275 mmol. 1.1 equiv) and $PhB(OH)_2$ (33.5, 0.275 mmol, 1.1 equiv). After purging reaction with a flow of argon for 15 minutes, a solution of **6** (70 mg, 0.25 mmol, 1.0 equiv) in anhydrous THF (1.0 mL) was added. The vial was sealed with a Teflon-lined cap and immediately placed in a pre-heated oil bath. After 24 h, the reaction was cooled to room temperature and passed through a plug of silica gel, washing with EtOAc (5 mL). The solvent was removed under reduced pressure. NMR yields were obtained by ^{1}H NMR analysis of the crude reaction mixture using 1,3,5-trimethoxybenzene as internal standard. The crude material was purified by column chromatography (20% EtOAc/hexanes) to afford the desired product as a white solid (52.3 mg, 0.163 mmol, 65%). **^{1}H NMR** (400 MHz, $CDCl_3$) δ 7.39 – 7.10 (m, 11H), 7.08 – 7.02 (m, 1H), 6.93 (t, *J* = 7.6 Hz, 1H), 6.68 (d, *J* = 7.6 Hz, 1H), 5.71 (d, *J* = 14.3 Hz, 1H), 4.41 (d, *J* = 14.3 Hz, 1H). **^{13}C NMR** (101 MHz, $CDCl_3$) δ 171.0, 140.2, 137.0, 136.0, 132.7, 132.1, 130.5, 129.9, 129.5, 129.0, 128.5, 128.0, 127.8, 127.7,

127.3, 52.1. **IR** ($CHCl_3$, cm^{-1}) 3063, 2928, 1647, 1479, 1383, 1319, 1296. **HRMS** (ESI): Calcd. for $[C_{20}H_{17}ClNO]^+$ $[M+H]^+$ 322.09987, found 322.09961.

Synthesis of **8** using independent methods:

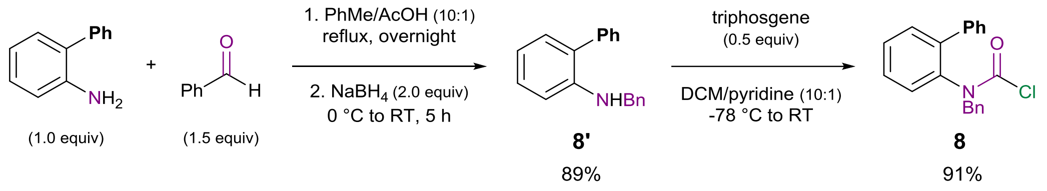

***N*-benzyl-[1,1'-biphenyl]-2-amine** (**8'**): A round bottom flask equipped with a stir bar was charged with 2-aminobiphenyl (846 mg, 5.0 mmol) and dissolved in toluene (10 mL). To this solution, benzaldehyde (796 mg, 7.5 mmol) and AcOH (3 mL) were sequentially added. The flask was equipped with a Dean-Stark apparatus and refluxed 16 h. The reaction was cooled to 0 °C and $NaBH_4$ (378 mg, 10.0 mmol) was added portionwise. The reaction was warmed to room temperature and stirred for 5 h. Upon full consumption of the in situ generated imine by ^1H NMR analysis of an aliquot sample, the reaction was cooled to 0 °C and quenched with H_20. The aqueous layer was extracted with DCM (3x) and the combined organics were washed with brine, dried over Na_2SO_4 and concentrated under reduced pressure. The crude material was purified by column chromatography (30% DCM/hexanes) to afford **8'** as a white solid (1.146 g, 4.41 mmol, 89%), The characterization data is consistent with literature values.[1]

[1,1'-biphenyl]-2-yl(benzyl)carbamic chloride (**8**): Following a literature procedure,[2] a solution of triphosgene (559 mg, 1.90 mmol) in anhydrous DCM (10 mL) was cooled to -78 °C. Pyridine (2.3 mL) was added dropwise under argon, resulting in the formation of a yellow precipitate. After stirring for 5 minutes, a solution of **8'** (978 mg, 3.80 mmol) in DCM (13 mL) was added and the reaction mixture was subsequently warmed to rt. The reaction became a homogeneous solution after 10-15 minutes. Upon full consumption of the starting material, the reaction was quenched by the addition of 1M HCl (15 mL) and the aqueous layer was extracted with DCM (3x). The combined organics were washed with brine (1x), dried over Na_2SO_4 and concentrated under reduced pressure. The crude material was purified by column chromatography (gradient elution: 5→10% Et_2O/hexanes) to afford **8** as a white solid (1.117 g, 3.47 mmol, 91%). **^{1}H NMR** (400 MHz, $CDCl_3$) δ 7.55 – 7.36 (m, 7H), 7.26 – 7.18 (m, 4H), 7.13 – 7.03 (m, 2H), 6.84 (d, ***J*** = 7.8 Hz, 1H), 4.98 (d, *J* = 14.3 Hz, 1H), 3.62 (d, *J* = 14.3 Hz, 1H). **^{13}C NMR** (126 MHz, $CDCl_3$) δ 150.3, 139.6, 138.7, 138.5, 135.3, 131.4, 131.2, 129.3, 129.2, 129.0, 128.6, 128.6, 128.2, 128.2, 128.1, 55.4. **IR** ($CHCl_3$, cm^{-1}) 3039, 1731, 1708, 1599, 1458, 1434, 1374, 1350, 1256, 1218, 1198. **HRMS** (DART): Calcd. for $[C_{20}H_{17}ClNO]^+$ $[M+H]^+$ 322.09987, found 322.09991.

1.3. Resting state analysis

A resting state analysis of the reaction of carbamoyl chloride **3a** and aryl chloride **4a** with catalytic amounts of Pd_2dba_3/PA-Ph was conducted and monitored via ^{31}P NMR.

Figure S1 (a): *Ligand complexation study*
An oven-dried J Young tube was charged with Pd_2dba_3 (9.2 mg, 0.01 mmol) and 1,3,5,7-tetramethyl-6-phenyl-2,4,8-trioxa-6-phosphaadamantane [PA-Ph] (11.7 mg, 0.04 mmol). After purging the vessel with a flow of argon for 15 minutes, 1.0 mL of toluene-d_8 was added. The J Young tube was sealed with a teflon-lined cap, shaken vigorously at rt for 2 minutes until the deep red solution turned orange/yellow and the ^{31}P NMR was taken immediately.

Figure S1 (b)-(c): *Resting state analysis with carbamoyl chloride **3a***

An oven-dried J Young tube was charged with Pd_2dba_3 (1.7 mg, 1.9 µmol), 1,3,5,7-tetramethyl-6-phenyl-2,4,8-trioxa-6-phosphaadamantane [PA-Ph] (2.2 mg, 7.5 µmol) and **3a** (63.9 mg, 0.15 mmol). After purging the vessel with a flow of argon for 15 minutes, 0.75 mL of toluene-d_8 was added. The J Young tube was sealed with a teflon-lined cap, shaken vigorously at rt for 2 minutes until the deep red solution turned orange/yellow, and placed in a pre-heated oil bath at 50 °C for 1 h. The ^{31}P NMR was taken upon cooling of the reaction to rt [t_0, Fig. S1 (b)]. The J Young tube was placed in the same oil bath for another 2 h and the ^{31}P NMR was taken again upon cooling of the reaction to rt [Fig. S1 (c)].

Figure S1 (d)-(e): *Resting state analysis with aryl chloride **4a***

An oven-dried J Young tube was charged with Pd_2dba_3 (1.7 mg, 1.9 µmol), 1,3,5,7-tetramethyl-6-phenyl-2,4,8-trioxa-6-phosphaadamantane [PA-Ph] (2.2 mg, 7.5 µmol) and **4a** (63.9 mg, 0.15 mmol). After purging the vessel with a flow of argon for 15 minutes, 0.75 mL of toluene-d_8 was added. The J Young tube was sealed with a teflon-lined cap, shaken vigorously at rt for 2 minutes until the deep red solution turned orange/yellow, and the ^{31}P NMR was taken immediately [t_0, Fig. S1 (d)]. The reaction was then placed in a pre-heated oil bath at 50 °C for 24 h and the ^{31}P NMR was taken again upon cooling of the reaction to rt [Fig. S1 (e)].

- species **A** (at 16.3 ppm) was formed in both cases and is most likely a result of catalyst decomposition, since it increases over time and was also observed in absence of any product formation (i.e. in the reaction of **4a**)
- a ligand complexation study (Fig. S1 (a)) showed species **B** at 13.4 ppm to be of the form $Pd(PA\text{-}Ph)_n(dba)_m$
- species **C** and **D** (at 6.8 and 6.7 ppm) were observed for both substrates and are likely the cis- and trans-Pd(II) intermediates **Va** and **VIa**
- species **E** (at 5.2 ppm) might be the oxidative addition intermediate **VIIa**, since it is only formed initially and decreases over time

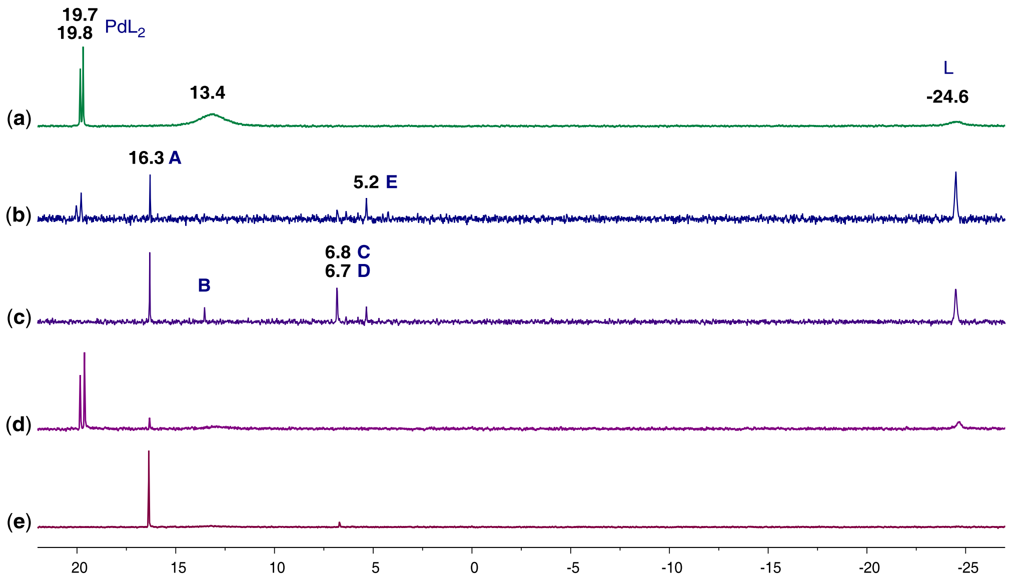

Fig. S1 (a) ligand complexation of Pd_2dba_3 with PA-Ph; reaction with **3a** at t_0 (b) and after 2h at 50°C (c); reaction with **4a** at t_0 (d) and after 24h at 50°C (e).

2. Computational Details

2.1. Full reference for Gaussian 09 Revision D.01

Gaussian 09, Revision D.01, M. J. Frisch, G. W. Trucks, H. B. Schlegel, G. E. Scuseria, M. A. Robb, J. R. Cheeseman, G. Scalmani, V. Barone, B. Mennucci, G. A. Petersson, H. Nakatsuji, M. Caricato, X. Li, H. P. Hratchian, A. F. Izmaylov, J. Bloino, G. Zheng, J. L. Sonnenberg, M. Hada, M. Ehara, K. Toyota, R. Fukuda, J. Hasegawa, M. Ishida, T. Nakajima, Y. Honda, O. Kitao, H. Nakai, T. Vreven, J. A. Montgomery, Jr., J. E. Peralta, F. Ogliaro, M. Bearpark, J. J. Heyd, E. Brothers, K. N. Kudin, V. N. Staroverov, T. Keith, R. Kobayashi, J. Normand, K. Raghavachari, A. Rendell, J. C. Burant, S. S. Iyengar, J. Tomasi, M. Cossi, N. Rega, J. M. Millam, M. Klene, J. E. Knox, J. B. Cross, V. Bakken, C. Adamo, J. Jaramillo, R. Gomperts, R. E. Stratmann, O. Yazyev, A. J. Austin, R. Cammi, C. Pomelli, J. W. Ochterski, R. L. Martin, K. Morokuma, V. G. Zakrzewski, G. A. Voth, P. Salvador, J. J. Dannenberg, S. Dapprich, A. D. Daniels, O. Farkas, J. B. Foresman, J. V. Ortiz, J. Cioslowski, and D. J. Fox, Gaussian, Inc., Wallingford CT, 2013.

2.2. General computational details

DFT calculations were performed using Gaussian 09, Revision D.01. Geometry optimization was conducted in the gas-phase at the B3LYP/6-31G(d) level of theory employing LANL2DZ as an ECP on Pd. Frequencies were calculated at the same level of theory and used to verify the nature of all stationary points as either minima (no imaginary frequencies) or transition states (one imaginary frequency). Additionally, transition states were confirmed by following the intrinsic reaction coordinate (IRC) to their corresponding intermediates. Single point energies were calculated at the M06L/def2-TZVP level of theory employing the CPCM model for toluene to account for solvation. All energies were corrected to 1M standard state (addition of 1.89 kcal/mol to every species). Images were created using the CYLview software.[3]

2.3. Free energetic span

Based on the energetic span model by Kozuch and Shaik, the efficiency and feasibility of the different catalytic cycles was addressed.[4] The smaller the energetic span, which corresponds to the appearent activation energy of the catalytic cycle, the faster the reaction. The energetic span δE is defined as the energy difference between the turnover frequency (TOF) determining transition state (TDTS) and the TOF-determining intermediate (TDI).

Since the oxidative addition of carbamoyl chlorides **3** to PdL_2 likely occurs via a fast, possibly ionic, nucleophilic substitution pathway, alkyne insertion was assumed to be the TDTS. Thus, the free energy spans were determined from calculated Gibbs free energies (at the CPCM (toluene) M06L/def2-TZVP//B3LYP/6-31G(d)(LANL2DZ) level of theory) as δE = G(TS_AI) – G(VI) or δE = G(TS_AI) – G(V), for intermediate **VI** or **V**, respectively, being the most stable intermediate.

2.4. Analysis of steric and electronic influences of the silyl group

a) *Steric influences*

TIPS is arguably the most bulky moiety (more bulky than $PdCl(P\mathit{t}Bu_3)$) and might favor **Va** over **VIa** due to a decreased interaction with the aryl moiety of the oxindole. However, mesityl can orient itself in a side-on position which would make the Pd-substituent the sterically more demanding substituent. Hence, the

increased stability of **VIb** over **Vb** is most likely the result of fewer steric interactions of the Pd-substituent (rather than mesityl) with the aryl moiety of the oxindole (Fig. S2).

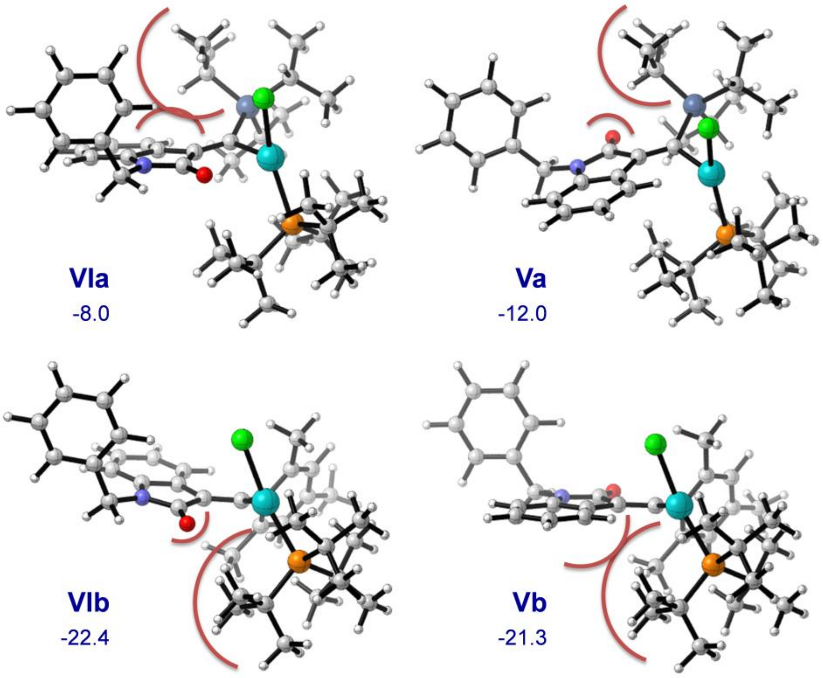

Fig. S2 Steric interactions and relative Gibbs free energies (in kcal/mol, relative to Pd(P*t*Bu$_3$)$_2$, calculated at the CPCM (toluene) M06L/def2-TZVP//B3LYP/6-31G(d)(LANL2DZ) level of theory) of Pd(II) intermediates **V** and **VI**.

b) *Natural bond order analysis* (NBO analysis, NBO charges)[5-8]

NBO analysis was performed using the Gaussian suite of programs at the M06L/def2-TZVP level of theory, based on geometries optimized at B3LYP/6-31G(d)(LANL2DZ) level of theory.

Table S1. NBO charges[a] of TIPS and Mes-substituents in Pd(II) intermediates (**VI** and **V**) and during isomerization (**TS_Isom**) as well as changes in NBO charges during isomerization (*i.e.* to and from **TS_Isom**).

	**VI** (*cis*)	**TS_Isom**	**V** (*trans*)	**VI→TS_Isom**	**TS_Isom→V**
**TIPS**	+0.53	+0.55	+0.54	+4.3%	-1.3%
**Mes**	+0.03	+0.16	+0.08	+136%	-88%

[a] calculated at the M06L/def2-TZVP//B3LYP/6-31G(d)(LANL2DZ) level of theory.

c) *AIM charges* (Atoms-In-Molecules analysis according to Bader)[9]

AIM analysis was performed at the M06L/def2-TZVP level of theory, based on geometry calculations at B3LYP/6-31G(d)(LANL2DZ), using the Multiwfn software.[10]

Table S2. AIM charges[a] of TIPS and Mes-substituents in Pd(II) intermediates (**VI** and **V**) and during isomerization (**TS_Isom**) as well as changes in AIM charges during isomerization (*i.e.* to and from **TS_Isom**).

	**VI** (*cis*)	**TS_Isom**	**V** (*trans*)	**VI→TS_Isom**	**TS_Isom→V**
**TIPS**	+2.74	+2.76	+2.74	+0.2%	-0.3%
**Mes**	+0.00	+0.04	+0.02	+77%	-32%

[a] calculated at the M06L/def2-TZVP//B3LYP/6-31G(d)(LANL2DZ) level of theory.

d) *Bond lengths and Mayer bond orders*[11, 12]

Mayer bond orders were calculated at the M06L/def2-TZVP//B3LYP/6-31G(d)(LANL2DZ) level of theory, using the Multiwfn software.[10]

Table S3. Bond lengths[a] of relevant bonds in Pd(II) intermediates (**VI** and **V**) and during isomerization (**TS_Isom**).

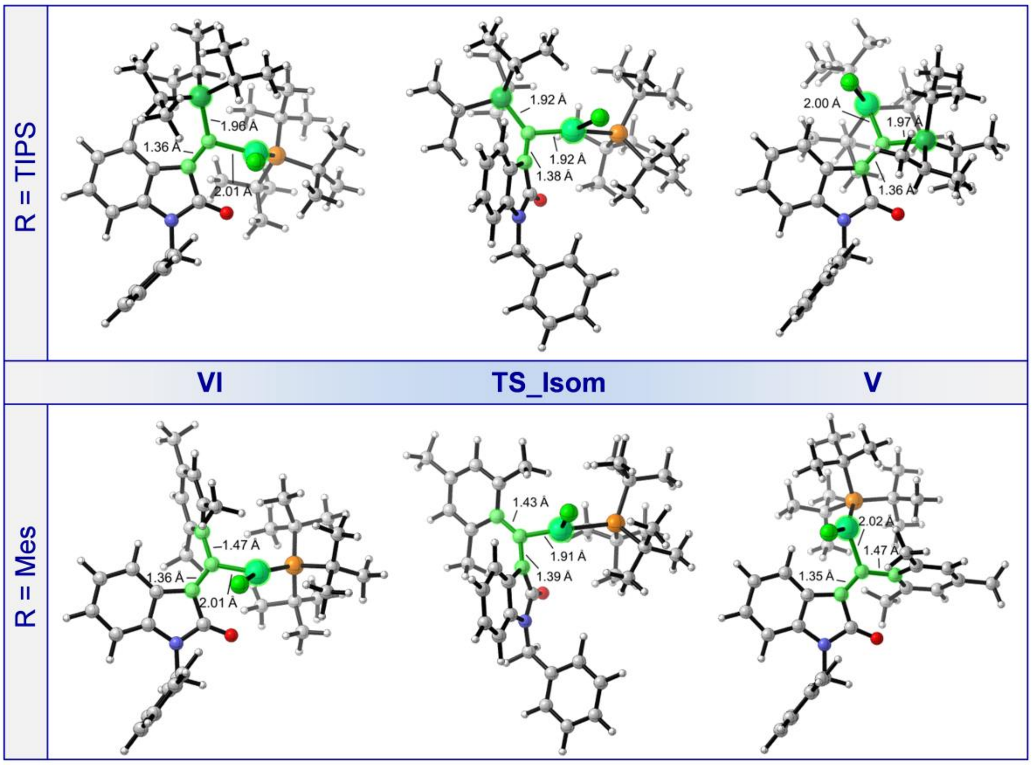

	bond	VI (*cis*)	TS_Isom	V (*trans*)	VI→TS_Isom	TS_Isom→V
**TIPS**	C=C	1.36	1.38	1.36	+0.5%	-0.5%
	C-Si	1.96	1.92	1.97	-0.7%	+0.9%
	C-Pd	2.01	1.92	2.00	-1.5%	+1.3%
**Mes**	C=C	1.36	1.39	1.35	+0.7%	-1.0%
	C-C_{ipso}(Mes)	1.47	1.43	1.47	-0.9%	+0.9%
	C-Pd	2.01	1.91	2.02	-1.7%	+1.9%

[a] calculated at the B3LYP/6-31G(d)(LANL2DZ) level of theory.

Table S4. Mayer bond orders[a] of relevant bonds in Pd(II) intermediates (**VI** and **V**) and during isomerization (**TS_Isom**).

	bond	VI (*cis*)	TS_Isom	V (*trans*)	VI→TS_Isom	TS_Isom→V
**TIPS**	C=C	1.30	0.78	1.24	-16%	+14%
	C-Si	0.82	1.03	0.90	+8%	-5%
	C-Pd	0.98	1.09	0.87	+4%	-7%
**Mes**	C=C	1.57	0.54	1.60	-28%	+28%
	C-C_{ipso}(Mes)	0.60	1.03	0.48	+20%	-26%
	C-Pd	0.85	1.11	0.87	+9%	-8%

[a] calculated at the M06L/def2-TZVP//B3LYP/6-31G(d)(LANL2DZ) level of theory.

2.5. Full energetic pathways

a) reaction of carbamoyl chloride **3a** (R = TIPS), L = PA-Ph

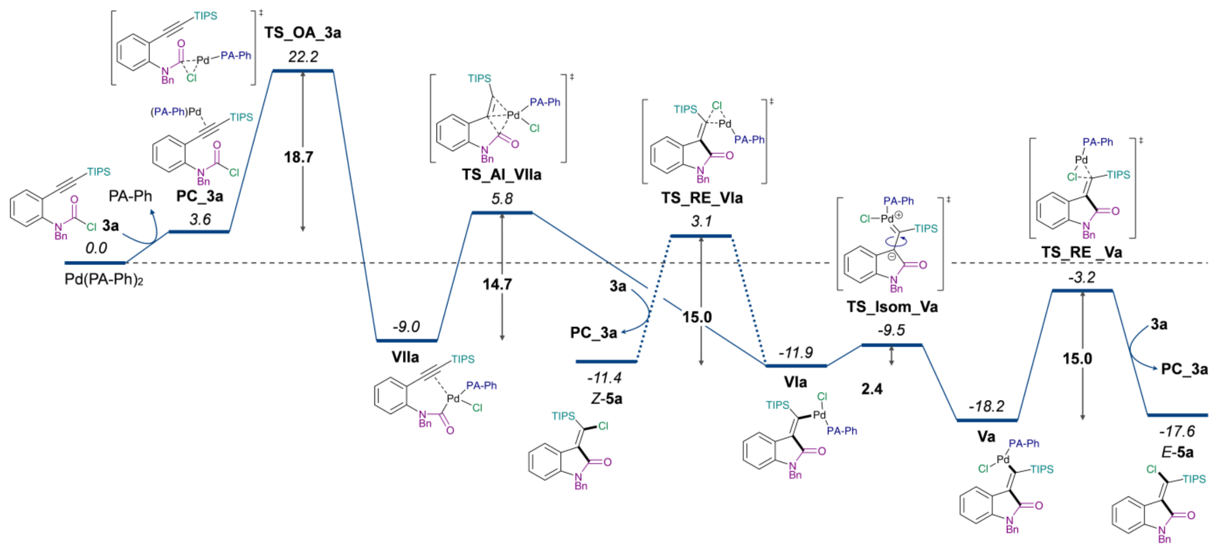

b) reaction of carbamoyl chloride **3a** (R = TIPS), L = PtBu$_3$

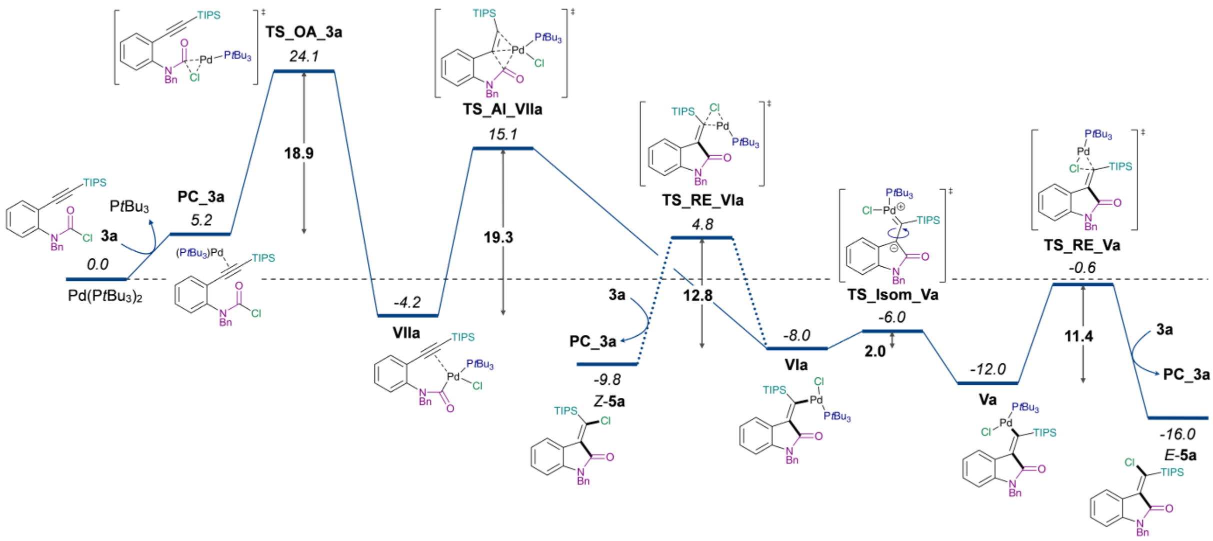

c) reaction of carbamoyl chloride **3b** (R = Mes), L = P*t*Bu₃

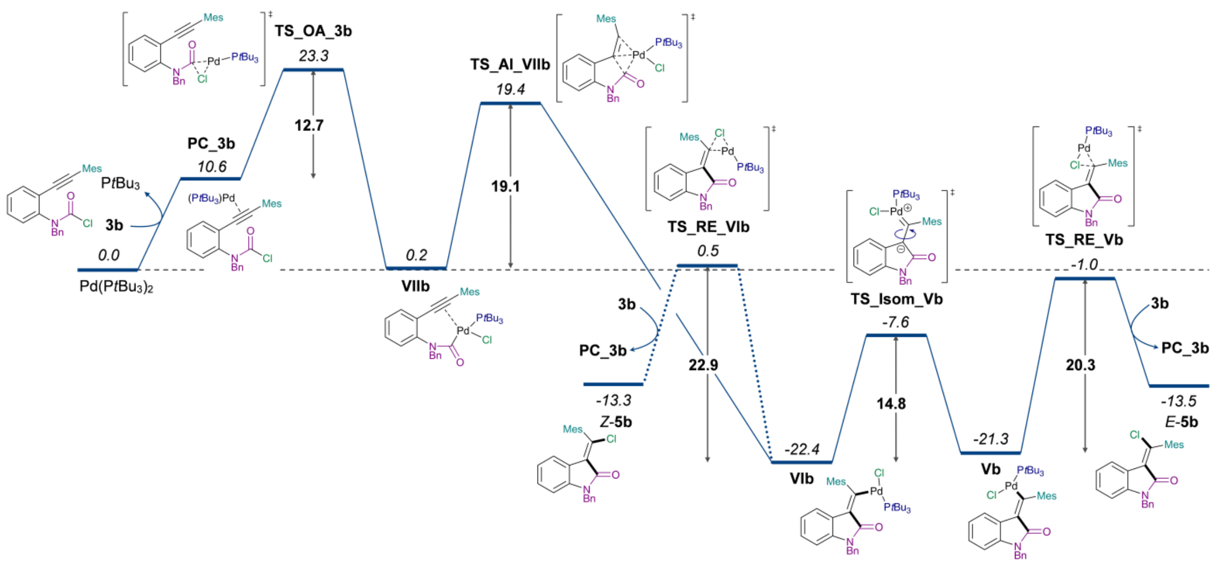

d) reaction of aryl chloride **4a** (R = TIPS), L = PA-Ph

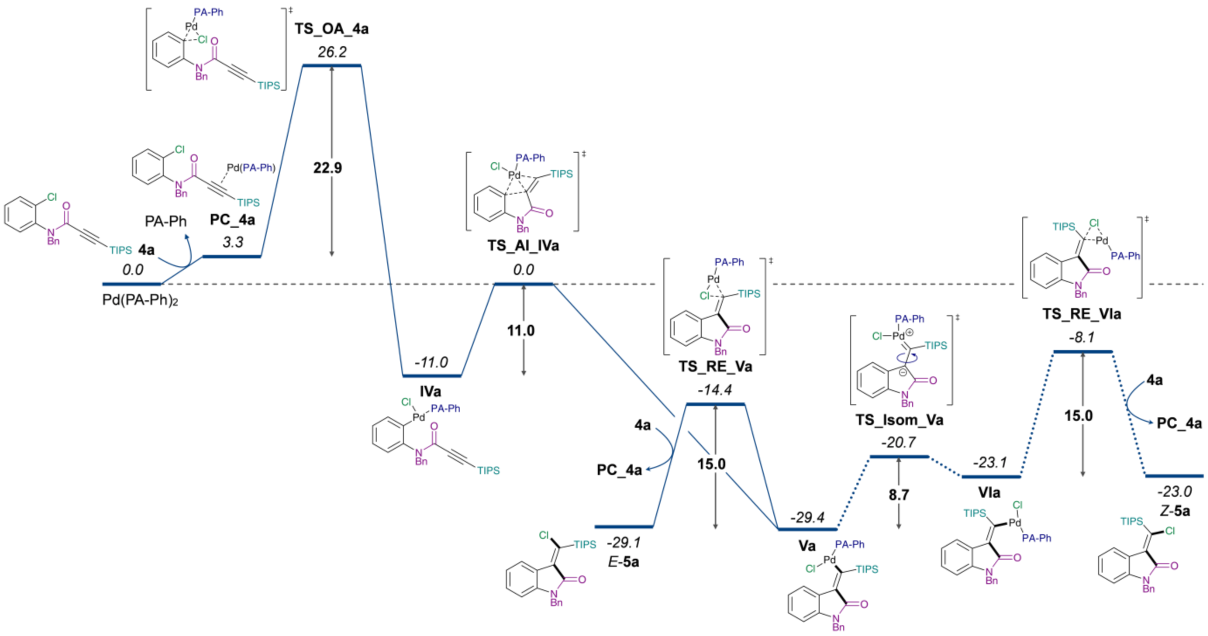

e) reaction of aryl chloride **4a** (R = TIPS), L = P*t*Bu$_3$

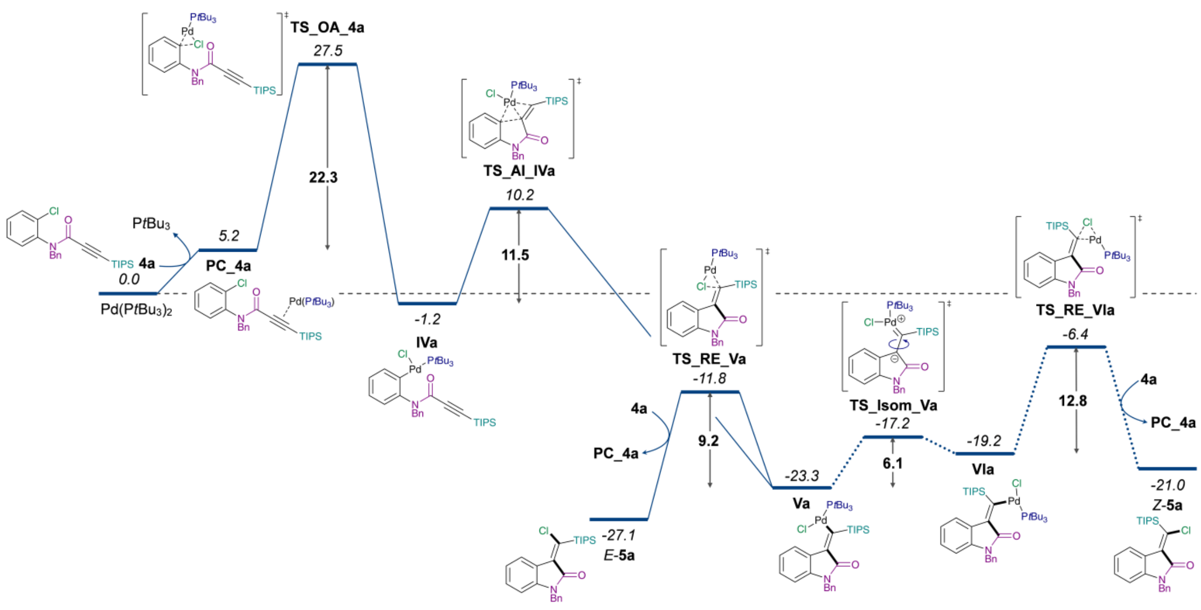

f) reaction of aryl chloride **4b** (R = Mes), L = P*t*Bu$_3$

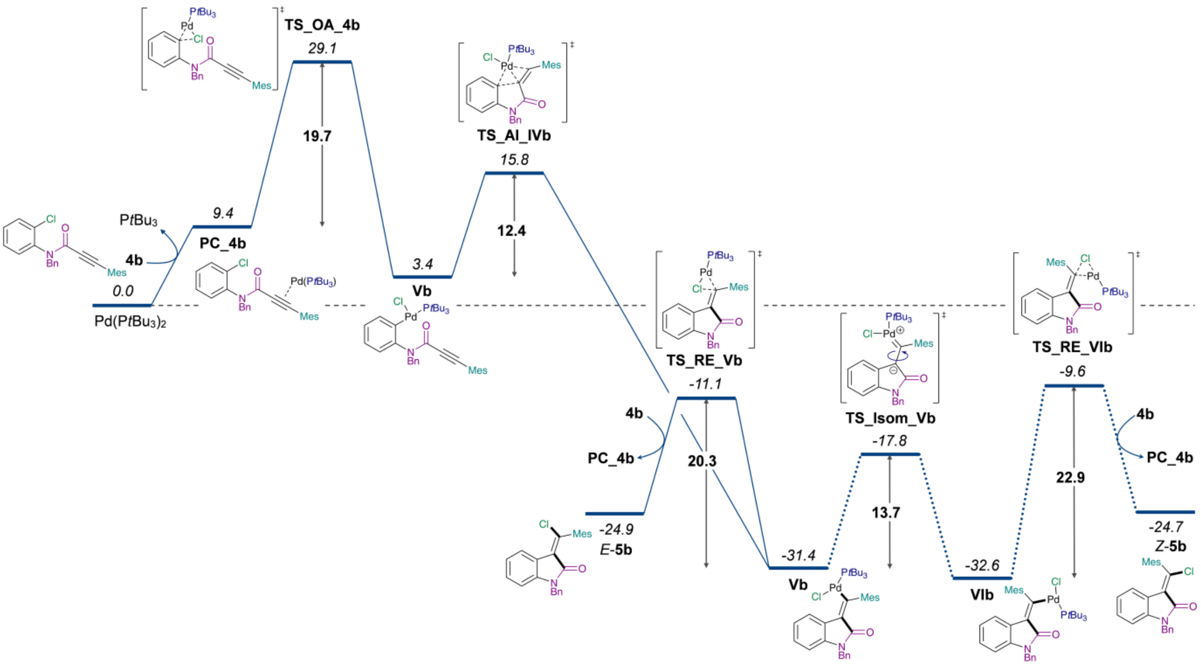

g) reaction of aryl bromide **1a** (R = TIPS), L = P*t*Bu3

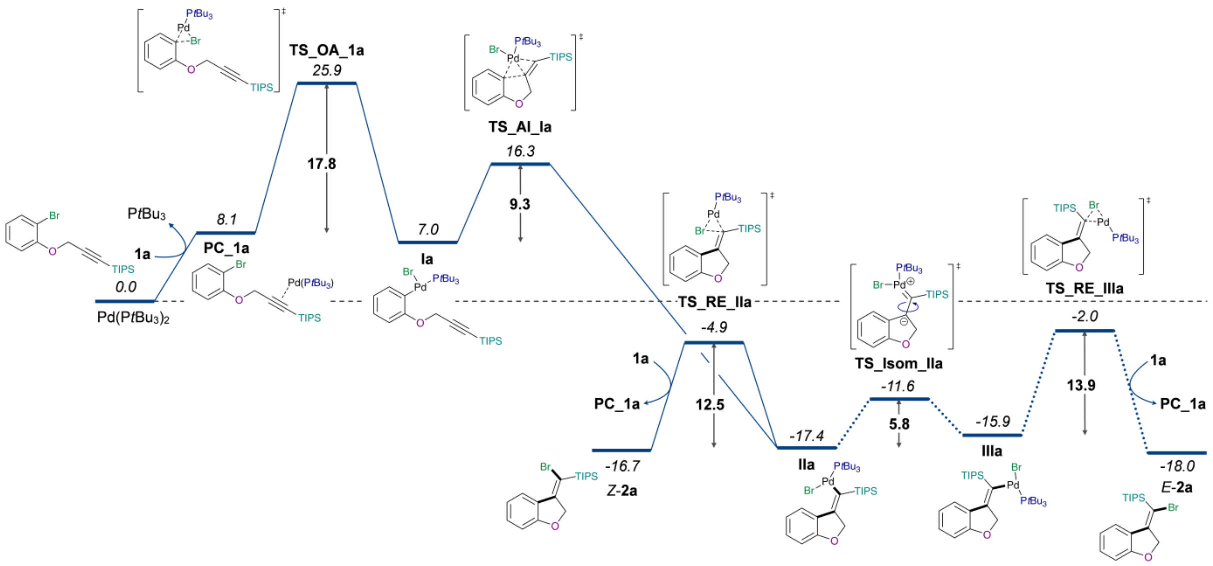

h) reaction of aryl bromide **1b** (R = Mes), L = P*t*Bu3

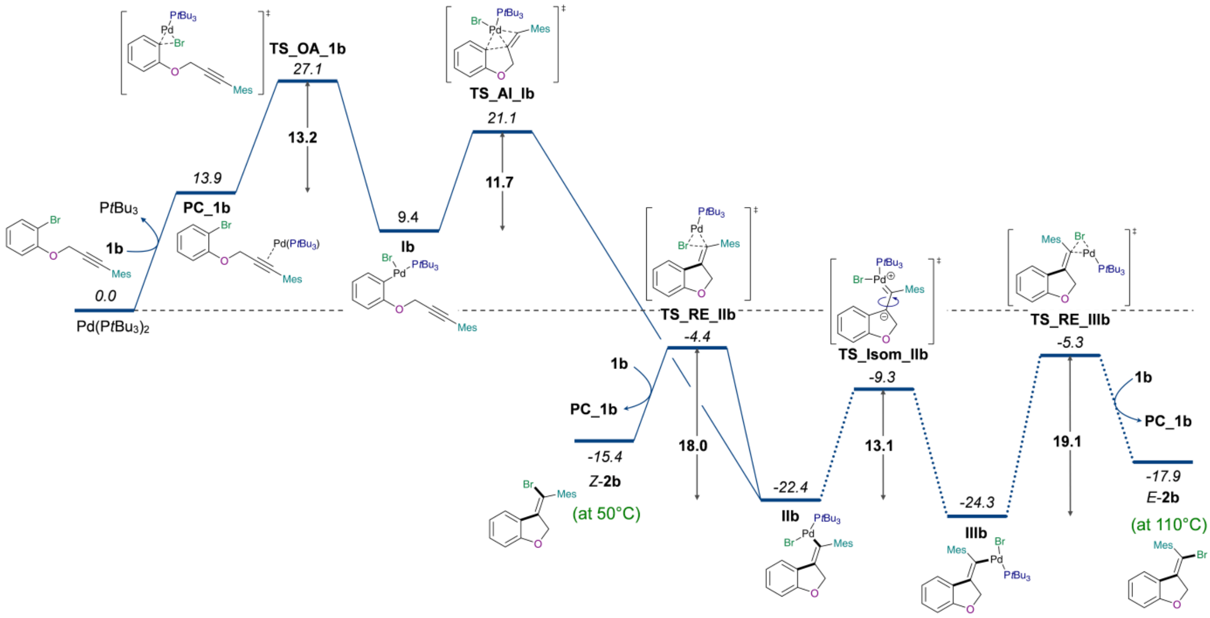

i) reaction of aryl chloride **1c** (R = TIPS), L = P*t*Bu3

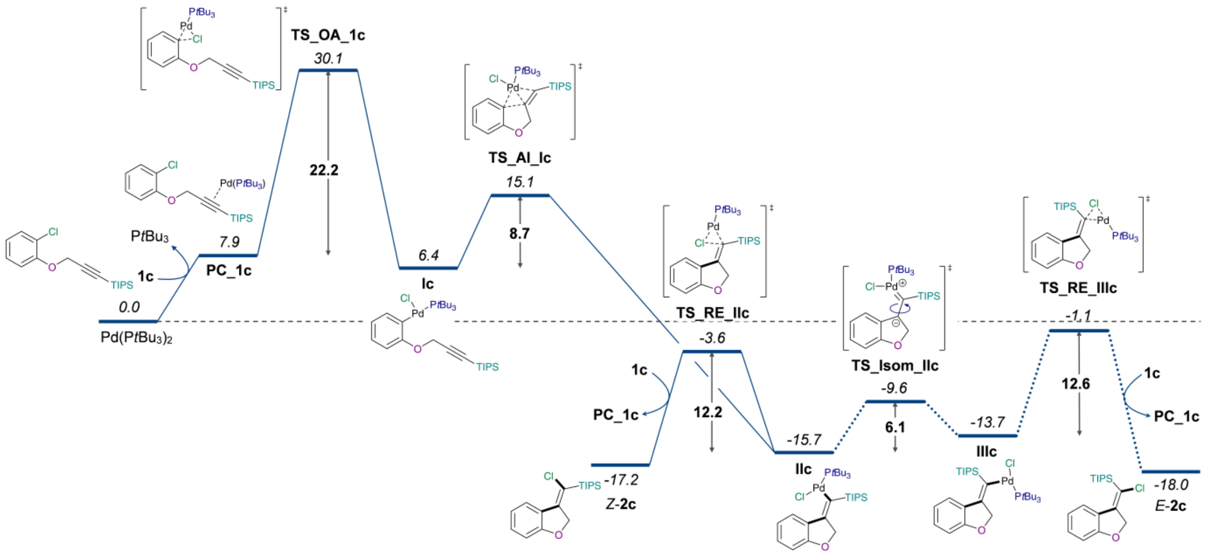

j) reaction of aryl chloride **1d** (R = Mes), L = P*t*Bu3

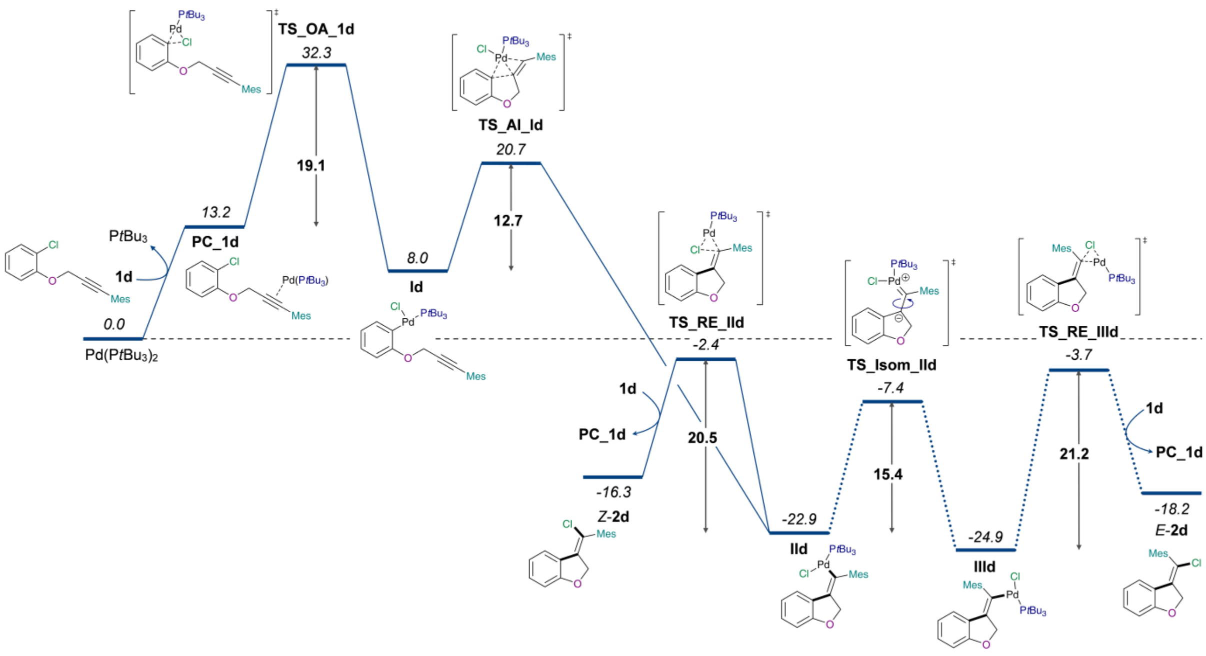

2.6. XYZ Coordinates and Energies for Optimized Structures

a) catalysts and ligands

Pd(PA-Ph)₂

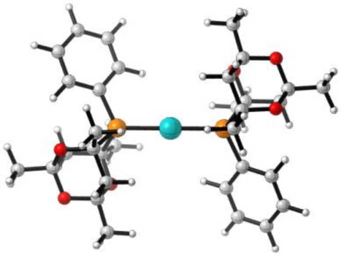

C	-2.95244500	-1.82089400	1.48503900
H	-3.38233000	-2.25620100	2.39549900
H	-1.87068400	-1.98765600	1.48744100
C	-3.26511100	-0.31367600	1.46433100
C	-3.59945200	-2.50839500	0.27182800
C	-5.30909800	-0.87559100	0.24925500
C	-4.81943600	-0.37529300	-1.11843700
H	-5.35423400	-0.93436500	-1.89618300
H	-5.04560800	0.68729500	-1.24471600
C	-3.31637400	-0.63868000	-1.27010700
P	-2.29307000	0.38066200	-0.01663100
C	-2.81678100	2.14059200	-0.23595700
C	-4.01278400	2.70673600	0.24256500
C	-1.91225900	2.96778800	-0.92627300
C	-4.29222700	4.05785400	0.02725100
H	-4.71726100	2.09142400	0.79047600
C	-2.20339500	4.31332500	-1.15522900
H	-0.96889800	2.54801100	-1.26841700
C	-3.39435700	4.86266300	-0.67690300
H	-5.21690500	4.48139900	0.41128300
H	-1.49327400	4.93347300	-1.69604700
H	-3.61856000	5.91280500	-0.84533600
C	-2.88737600	0.36806600	2.77337300
H	-1.81629300	0.24547700	2.96277400
H	-3.11491100	1.43784600	2.74460800
H	-3.45079600	-0.08381300	3.59801200
C	-2.82465000	-0.42333400	-2.69367400
H	-2.98249300	0.61610400	-3.00123800
H	-1.75649500	-0.65089900	-2.76324900
H	-3.37345800	-1.08000800	-3.37913100
C	-6.80742200	-0.73598500	0.44971600
H	-7.34134800	-1.30654000	-0.31526900
H	-7.07791400	-1.12233900	1.43611500
H	-7.10281400	0.31537700	0.38489600
C	-3.40871400	-4.01375500	0.25479200
H	-3.83730300	-4.45661400	1.15818700
H	-3.91241900	-4.43234500	-0.62077500
H	-2.34323000	-4.25519900	0.20445000
O	-4.69379800	-0.14720700	1.31691500
O	-5.00483400	-2.26364400	0.33874100
O	-3.05726300	-2.02322800	-0.95821300
Pd	-0.00001400	0.00001200	-0.03073300
C	3.26516600	0.31341600	1.46424000
C	3.31631200	0.63891300	-1.27014300
C	2.95255200	1.82064200	1.48523200
C	4.81937000	0.37543800	-1.11858700
H	3.38249400	2.25577000	2.39575000
H	1.87079800	1.98744200	1.48771300
C	3.59953100	2.50833900	0.27211700
C	5.30911600	0.87547300	0.24917200
H	5.04549100	-0.68713900	-1.24506100
H	5.35415600	0.93462400	-1.89625900
C	2.81674300	-2.14052000	-0.23649000
C	4.01282600	-2.70671600	0.24176300
C	1.91214200	-2.96761500	-0.92682500
C	4.29226300	-4.05779400	0.02619200
H	4.71738300	-2.09147100	0.78964700
C	2.20326900	-4.31311000	-1.15603700
H	0.96872600	-2.54780000	-1.26876800
C	3.39430700	-4.86250600	-0.67796400
H	5.21700800	-4.48138300	0.41001600
H	1.49308400	-4.93318300	-1.69685800
H	3.61850600	-5.91261500	-0.84660000
P	2.29304500	-0.38063000	-0.01680600
C	2.88746100	-0.36854300	2.77317800
H	3.45093100	0.08317200	3.59787400
H	1.81639000	-0.24594800	2.96264500
H	3.11495400	-1.43832600	2.74421800
C	2.82451300	0.42384100	-2.69372600
H	1.75636200	0.65145400	-2.76320700
H	3.37330800	1.08062300	-3.37908900
H	2.98230800	-0.61554500	-3.00148600
C	3.40884700	4.01370800	0.25536300
H	2.34337000	4.25520300	0.20511400
H	3.83749500	4.45638800	1.15881700
H	3.91252700	4.43243800	-0.62015200
C	6.80744400	0.73577500	0.44953800
H	7.34135500	1.30644400	-0.31537400
H	7.07799600	1.12194700	1.43599100
H	7.10279200	-0.31558700	0.38452000
O	5.00490600	2.26352100	0.33891900
O	3.05726300	2.02341400	-0.95798500
O	4.69383900	0.14692300	1.31673300

Zero-point correction = 0.692302 (Hartree/Particle)
Thermal correction to Energy = 0.732725
Thermal correction to Enthalpy = 0.733669
Thermal correction to Gibbs Free Energy = 0.619130
Sum of electronic and zero-point Energies = -2504.856537
Sum of electronic and thermal Energies = -2504.816115
Sum of electronic and thermal Enthalpies = -2504.815170
Sum of electronic and thermal Free Energies = -2504.929709
E(RM06L) = -2507.25659046

PA-Ph

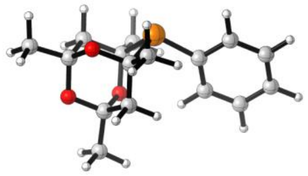

C	2.05361200	-0.16979800	1.54432400
H	2.67724600	-1.00880700	1.87657900
H	2.04584800	0.59174000	2.33081500
C	0.62999300	-0.68935700	1.26611600
C	2.66361900	0.40231900	0.25334800
C	1.35297800	-1.09170400	-1.02782200
C	0.58868500	0.09561000	-1.63368500
H	1.11323700	0.40284400	-2.54723900
H	-0.42903200	-0.19865800	-1.90548500
C	0.57257800	1.27085600	-0.65112500
P	-0.41012400	0.87484700	0.94603000
C	-2.06109900	0.27817300	0.35156300
C	-2.36435000	-1.00218800	-0.14921100
C	-3.10889200	1.21092900	0.46438100
C	-3.66845500	-1.32788500	-0.52732800
H	-1.57708600	-1.74219200	-0.23872200
C	-4.40854400	0.89021600	0.06886100
H	-2.90317300	2.19667300	0.87548500
C	-4.69234600	-0.38344700	-0.42635100
H	-3.88373700	-2.32412800	-0.90585400
H	-5.19926400	1.63050300	0.15939200
H	-5.70497800	-0.64066500	-0.72588600
C	0.08593400	-1.50868600	2.43111800
H	0.06309300	-0.90355000	3.34348800
H	-0.92790500	-1.86569400	2.22800100
H	0.73053200	-2.37890700	2.60171400

C	0.08266400	2.56184300	-1.29106800
H	-0.95675500	2.45787600	-1.62015000
H	0.14474100	3.38997300	-0.57802900
H	0.70317000	2.80648500	-2.16150200
C	1.49857700	-2.26988900	-1.97390500
H	2.03455500	-1.96122400	-2.87574600
H	2.06291900	-3.06439500	-1.47810100
H	0.51362800	-2.65398900	-2.25528000
C	4.09813300	0.87179200	0.41399900
H	4.72941200	0.04526100	0.75200100
H	4.46929800	1.23322500	-0.54884800
H	4.14674600	1.68604800	1.14263700
O	0.69930100	-1.59439600	0.13950800
O	2.67056000	-0.64348000	-0.71686800
O	1.92524500	1.53114700	-0.21501800

Zero-point correction = 0.345130 (Hartree/Particle)
Thermal correction to Energy = 0.363250
Thermal correction to Enthalpy = 0.364195
Thermal correction to Gibbs Free Energy = 0.301522
Sum of electronic and zero-point Energies = -1189.018340
Sum of electronic and thermal Energies = -1189.000220
Sum of electronic and thermal Enthalpies = -1188.999275
Sum of electronic and thermal Free Energies = -1189.061948
E(RM06L) = -1189.58169855

Pd(P*t*Bu$_3$)$_2$

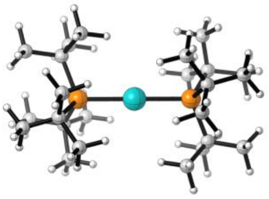

Pd	0.00006800	0.00287900	0.00232700
C	-4.55244600	2.00569200	0.28731200
C	-3.06646800	1.82072500	-0.07990400
H	-4.76034800	1.75389600	1.33048900
H	-4.82634000	3.06165800	0.15310300
H	-5.22068300	1.41395900	-0.34373600
P	-2.36509700	0.00045800	0.00011900
C	-2.20057000	2.69858400	0.85655700
C	-2.84052800	2.38751200	-1.49918300
C	-3.06036600	-0.97984900	-1.53816500
C	-3.06498400	-0.84238700	1.61590900
H	-1.13766700	2.58779600	0.61680000
H	-2.48334400	3.75138200	0.71690000
H	-2.33173600	2.46374500	1.91330500
H	-3.04675800	3.46613900	-1.48186500
H	-1.80399300	2.25292400	-1.82521600
H	-3.50398000	1.94729200	-2.24711300
C	-2.19173200	-0.60633500	-2.76426300
C	-2.83227900	-2.49186500	-1.31878600
C	-4.54577500	-0.75686400	-1.88590100
C	-2.84217500	0.10379900	2.81658400
C	-4.54980500	-1.25680600	1.59137900
C	-2.19578700	-2.08984200	1.90839200
H	-2.32540900	0.42533100	-3.09101600
H	-1.12903700	-0.75444600	-2.54466700
H	-2.46943700	-1.25570900	-3.60631000
H	-3.49669800	-2.92071600	-0.56513500
H	-3.03523800	-3.01655200	-2.26207000
H	-1.79595400	-2.70483800	-1.03679700
H	-4.81602300	-1.40031200	-2.73502600
H	-5.21594700	-1.00991700	-1.06020800
H	-4.75449800	0.27256900	-2.18834800
H	-1.80655000	0.45611100	2.86341200
H	-3.04717800	-0.45127100	3.74186500
H	-4.75487500	-2.03570000	0.85236900
H	-4.82373800	-1.66811300	2.57315800
H	-5.21991100	-0.41634500	1.39263500
H	-2.32291800	-2.88737400	1.17578700
H	-1.13381300	-1.82340800	1.93444000
H	-2.47899300	-2.49696500	2.88917400
H	-3.50798300	0.96983800	2.80925500
H	5.21966000	0.10681300	1.45188100
C	4.55225200	0.89023300	1.82021400
C	3.06585400	0.48570300	1.75661700
H	4.76126800	1.80463000	1.25902100
H	4.82599600	1.08771800	2.86623300
P	2.36540900	0.00049000	0.00016500
C	2.20086500	1.64846100	2.30162100
C	2.83721900	-0.68898900	2.73352100
C	3.06070000	-1.76563800	-0.45733900
C	3.06548500	1.27779000	-1.29973100
H	1.13771500	1.38737800	2.26931800
H	2.48288900	1.84036100	3.34636600
H	2.33349800	2.58094000	1.75208500
H	3.04488100	-0.34071600	3.75422800
H	1.79960200	-1.03750300	2.70635000
H	3.49806500	-1.53810300	2.54482100
C	2.19031800	-2.81646700	0.27444400
C	2.83495300	-2.02278600	-1.96382000
C	4.54541700	-2.02847900	-0.13537100
C	2.84225700	2.71164600	-0.76985700
C	4.55043700	1.12733800	-1.68641900
C	2.19653200	1.17146900	-2.57675800
H	2.32050700	-2.80798100	1.35707500
H	1.12820000	-2.65402500	0.06221600
H	2.46964000	-3.81794900	-0.08175200
H	3.50049600	-1.43809500	-2.60303100
H	3.03801800	-3.08187100	-2.17208800
H	1.79916900	-1.81982300	-2.25500700
H	4.81655300	-3.03359300	-0.48797600
H	5.21651900	-1.31951400	-0.62710400
H	4.75196500	-2.00189300	0.93769700
H	1.80640700	2.86498600	-0.44994100
H	3.04802200	3.42083200	-1.58287200
H	3.50728600	2.97151700	0.05689400
H	4.75598500	0.18377300	-2.19847500
H	4.82364800	1.93391800	-2.38131700
H	5.22080900	1.19878200	-0.82594500
H	2.32601200	0.22980200	-3.11109400
H	1.13429100	1.27546000	-2.33121100
H	2.47786900	1.98067000	-3.26516500

Zero-point correction = 0.745540 (Hartree/Particle)
Thermal correction to Energy = 0.784989
Thermal correction to Enthalpy = 0.785933
Thermal correction to Gibbs Free Energy = 0.678584
Sum of electronic and zero-point Energies = -1755.807831
Sum of electronic and thermal Energies = -1755.768383
Sum of electronic and thermal Enthalpies = -1755.767439
Sum of electronic and thermal Free Energies = -1755.874788
E(RM06L) = -1758.07815378

P*t*Bu$_3$

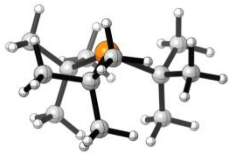

P	0.00018300	-0.00026700	-0.71063900
C	1.69182500	-0.66765300	-0.00171900
C	-0.26747400	1.79854600	-0.00188100
C	-1.42418900	-1.13098000	-0.00190900
C	-2.66588200	-0.88715500	-0.89619000
C	-1.05399200	-2.61525700	-0.21869400
C	-1.81736900	-0.94374900	1.47656100
C	0.08989600	2.04568000	1.47690500
C	0.56494200	2.75182200	-0.89588900
C	-1.73787800	2.22003500	-0.21985700
C	1.72653800	-1.09998200	1.47718300
C	2.10114200	-1.86572500	-0.89509600
H	-3.08448100	0.11438500	-0.79285500
H	-3.45495300	-1.60004600	-0.61884800
H	-2.42787100	-1.04491500	-1.95364100
H	-0.74948600	-2.81383600	-1.25194200
H	-0.26185200	-2.96092800	0.44932700
H	-1.93915700	-3.23275100	-0.01379100
H	-2.21510200	0.05370800	1.68113600

H	-0.97788300	-1.11688600	2.15535600
H	-2.60632200	-1.66321000	1.74026800
H	1.15212400	1.89015300	1.68281800
H	-0.48153800	1.40647300	2.15547800
H	-0.13754900	3.08914000	1.73974200
H	0.34253100	3.79166600	-0.61837300
H	1.64152900	2.61311200	-0.79269000
H	0.30918400	2.62483800	-1.95334400
H	-1.83041700	3.29523500	-0.01449700
H	-2.06094600	2.05614100	-1.25354700
H	-2.43397100	1.70643200	0.44717700
H	2.74390800	-1.42402800	1.74094200
H	1.45756900	-0.28535300	2.15509900
H	1.06105800	-1.94239300	1.68272500
H	2.11711700	-1.58185700	-1.95289300
H	3.11367000	-2.19161100	-0.61879200
H	1.44383100	-2.72939700	-0.78998400
C	2.79224200	0.39476600	-0.22002900
H	2.69583100	1.25464900	0.44675800
H	3.76950100	-0.06297500	-0.01451400
H	2.81212700	0.75618100	-1.25383800

Zero-point correction = 0.371322 (Hartree/Particle)
Thermal correction to Energy = 0.389215
Thermal correction to Enthalpy = 0.390159
Thermal correction to Gibbs Free Energy = 0.329824
Sum of electronic and zero-point Energies = -814.493916
Sum of electronic and thermal Energies = -814.476024
Sum of electronic and thermal Enthalpies = -814.475080
Sum of electronic and thermal Free Energies = -814.535414
E(RM06L) = -814.98741509

b) reaction of carbamoyl chloride **3a** (R = TIPS), L = PA-Ph

3a

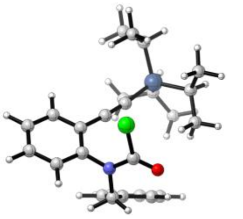

C	0.21968400	-0.90786100	-0.68993900
C	-0.92514900	-0.59455300	-0.40000500
Si	-2.70422100	-0.25100800	-0.03034800
C	-2.91987600	0.19096500	1.82735800
H	-3.86841400	0.75314900	1.85726200
C	-3.30570300	1.20146300	-1.14702300
H	-4.38036900	1.00128000	-1.29050000
C	-3.69532000	-1.84814700	-0.45959100
H	-4.61885800	-1.76605800	0.13723700
C	-4.11685900	-1.96025200	-1.93740400
H	-3.24357600	-2.05116500	-2.59543300
H	-4.73140500	-2.85762300	-2.09322900
H	-4.70287000	-1.09940800	-2.27657700
C	-2.97249600	-3.14184900	-0.03290100
H	-2.70345400	-3.14710200	1.02723000
H	-3.60947800	-4.01787300	-0.21810200
H	-2.04730900	-3.27838000	-0.60443900
C	-3.08112700	-1.02093300	2.76562800
H	-3.26619800	-0.68260000	3.79430500
H	-3.91716500	-1.66823800	2.47902400
H	-2.17114500	-1.63260800	2.78835600
C	-1.80898900	1.11485100	2.36564900
H	-2.04702700	1.44401200	3.38653400
H	-0.84641600	0.59430100	2.40896500
H	-1.66966700	2.00996700	1.75254500
C	-3.18843800	2.60316300	-0.51896300
H	-3.64150700	3.35468600	-1.18064100
H	-3.69381200	2.67383000	0.45032000
H	-2.14043800	2.89017300	-0.37506500
C	-2.63768400	1.20086600	-2.53705300
H	-3.07418700	1.98221100	-3.17486600
H	-1.56382300	1.40288700	-2.45183100
H	-2.75252000	0.24642600	-3.06072200
C	1.51639400	-1.35366300	-1.08257800
C	2.68374200	-1.05853700	-0.34001200
C	1.64616000	-2.15118400	-2.23846100
C	3.92337100	-1.54771400	-0.75888800
C	2.88629600	-2.62728100	-2.65053300
H	0.75080300	-2.38507500	-2.80518900
C	4.03158300	-2.32699500	-1.90908900
H	4.80345800	-1.31990900	-0.16440100
H	2.95876300	-3.23630200	-3.54703000
H	5.00216600	-2.70393300	-2.21786400
N	2.63965500	-0.19876200	0.80565400
C	3.22532700	1.16604900	0.67932700
H	3.56459500	1.45099700	1.67669100
H	4.10190300	1.06254100	0.03447800
C	2.29178800	2.21853800	0.11885400
C	1.46546000	2.96068900	0.97357900
C	2.26845500	2.49006700	-1.25509900
C	0.63566000	3.95901100	0.46178400
H	1.47734800	2.74954100	2.03875200
C	1.43300700	3.48291600	-1.76959300
H	2.91357200	1.92678800	-1.92564200
C	0.61714400	4.22223500	-0.91069000
H	0.00862200	4.53674000	1.13565400
H	1.42749300	3.68600400	-2.83716300
H	-0.02517100	5.00379700	-1.30758000
C	2.08253900	-0.51115000	2.00711800
O	1.91811300	0.24043100	2.93370500
Cl	1.65252300	-2.26501300	2.18268900

Zero-point correction = 0.513759 (Hartree/Particle)
Thermal correction to Energy = 0.545788
Thermal correction to Enthalpy = 0.546732
Thermal correction to Gibbs Free Energy = 0.447754
Sum of electronic and zero-point Energies = -1851.059245
Sum of electronic and thermal Energies = -1851.027216
Sum of electronic and thermal Enthalpies = -1851.026271
Sum of electronic and thermal Free Energies = -1851.125249
E(RM06L) = -1851.87224866

PC_3a_PAPh

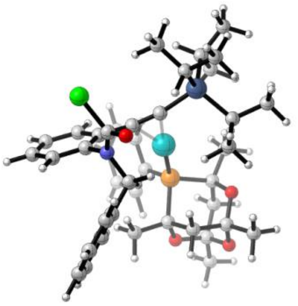

C	1.49734900	0.37905800	1.13273100
C	1.47895500	1.45169400	0.48782600
C	1.89220100	-0.78933200	1.87466700
C	2.95817300	-1.60974200	1.43686500
C	1.21975100	-1.14586200	3.05992600
C	3.31365700	-2.75150200	2.15697400
C	1.59849300	-2.27061500	3.78612000
H	0.39346800	-0.52509400	3.38992000
C	2.64539200	-3.07930200	3.33534800
H	4.12380600	-3.37069300	1.78604100
H	1.06965400	-2.52159000	4.70122200
H	2.93589600	-3.96484700	3.89288600
N	3.62602100	-1.30935300	0.20428800
C	2.94537900	-1.70019000	-1.06080600
H	1.88172400	-1.48907000	-0.92427800

H	3.32797400	-1.03688300	-1.83820100
C	3.16505200	-3.15281400	-1.42966200
C	2.16196900	-4.10415200	-1.20901400
C	4.38148000	-3.56642200	-1.99422500
C	2.36550600	-5.44499300	-1.54344600
H	1.21416200	-3.79307800	-0.77512100
C	4.58684500	-4.90540000	-2.32559100
H	5.16289500	-2.83234900	-2.17042300
C	3.57932100	-5.84807500	-2.10145000
H	1.57650600	-6.17173700	-1.36867900
H	5.53205700	-5.21303200	-2.76502100
H	3.73952300	-6.89038100	-2.36426200
C	4.84963700	-0.73260200	0.09434100
O	5.44191600	-0.48697200	-0.92599300
Cl	5.60428200	-0.30637100	1.69362800
Si	1.87106000	3.08809100	-0.32706100
C	3.78814600	3.16847700	-0.40329000
C	1.03134800	3.01948300	-2.05065600
C	1.11569400	4.42767600	0.82573400
H	4.08962700	2.18431400	-0.79255600
C	4.36639400	4.22728900	-1.36394900
C	4.40859100	3.30939100	1.00170100
H	0.00223400	2.69898300	-1.82580300
C	1.65431700	1.93469700	-2.95115000
C	0.94261600	4.36547000	-2.79661500
H	1.45750400	4.13344600	1.83015400
C	-0.42452300	4.39445900	0.83966900
C	1.62325100	5.86211500	0.57325300
H	4.08602300	5.24709100	-1.07481300
H	5.46388500	4.18117500	-1.36240800
H	4.03675300	4.07307300	-2.39705100
H	4.04890100	2.53748000	1.69058300
H	5.50120800	3.21695300	0.95146900
H	4.18386500	4.28680500	1.44658300
H	1.09427600	1.84253400	-3.89220800
H	2.69300800	2.16954000	-3.21519400
H	1.64687600	0.95281100	-2.46455100
H	1.93192500	4.77334700	-3.03435800
H	0.40887100	4.23971200	-3.74875800
H	0.40215600	5.12378100	-2.21973500
H	-0.80840200	3.38859900	1.05596400
H	-0.82115500	5.07808900	1.60339600
H	-0.84243200	4.70919300	-0.12510300
H	1.35148900	6.22495400	-0.42491600
H	1.17965900	6.55715800	1.29950200
H	2.71102700	5.94187000	0.66918700
C	-3.05491000	-1.65385900	-0.70171000
C	-3.95579100	0.95098500	-0.74034700
C	-2.48707200	-1.31696800	-2.09230300
C	-5.34268100	0.30338000	-0.84086600
H	-2.50371100	-2.22512100	-2.70698700
H	-1.45492400	-0.96296900	-2.01002100
C	-3.36529000	-0.25725300	-2.77683500
C	-5.24968100	-1.03884000	-1.58137000
H	-5.78449400	0.16871200	0.15051000
H	-5.99658400	0.96648400	-1.42038200
C	-3.65338700	-0.34906900	1.95717700
C	-4.69127500	-1.27211400	2.18269000
C	-3.21375200	0.44171400	3.03398800
C	-5.27006100	-1.39083800	3.44795500
H	-5.03872300	-1.90027900	1.37047900
C	-3.80712400	0.33335900	4.29243400
H	-2.39025400	1.13457700	2.87727900
C	-4.83621000	-0.58613500	4.50332900
H	-6.06495800	-2.11506000	3.60718600
H	-3.45696000	0.95915300	5.10920500
H	-5.29319800	-0.67951300	5.48498900
P	-2.77747000	-0.09057700	0.34910900
C	-2.39285100	-2.88400100	-0.09348200
H	-2.55304300	-3.74777900	-0.74900900
H	-1.31752400	-2.71326300	0.01891500
H	-2.81075800	-3.11531100	0.89079000
C	-4.02345200	2.39700800	-0.27227800
H	-3.02477200	2.84297400	-0.25926300
H	-4.65790600	2.97641200	-0.95326600
H	-4.44692400	2.45555700	0.73620600
C	-2.92281900	0.07855600	-4.18880400
H	-1.91088600	0.49316900	-4.17587000
H	-2.93685400	-0.82107300	-4.81033400
H	-3.60628700	0.81739100	-4.61586900
C	-6.59385300	-1.70651800	-1.80812100
H	-7.24014400	-1.05301200	-2.40051800
H	-6.44479800	-2.64510100	-2.34866700
H	-7.07875400	-1.91902400	-0.85079300
O	-4.68730100	-0.78767900	-2.86490000
O	-3.35846500	0.97632700	-2.05215800
O	-4.45515800	-1.97846400	-0.84892700
Pd	-0.59101900	0.67641500	0.47821200

Zero-point correction= 0.859554 (Hartree/Particle)
Thermal correction to Energy= 0.914213
Thermal correction to Enthalpy= 0.915158
Thermal correction to Gibbs Free Energy= 0.763945
Sum of electronic and zero-point Energies= -3166.889759
Sum of electronic and thermal Energies= -3166.835100
Sum of electronic and thermal Enthalpies= -3166.834155
Sum of electronic and thermal Free Energies= -3166.985368
E(RM06L) = -3169.54004213

TS_OA_3a_PAPh

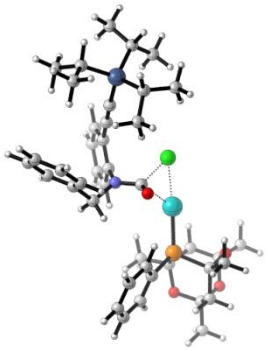

C	3.50641500	-0.69952800	1.13340600
C	4.44385400	-0.77645700	0.35372900
Si	5.86404000	-1.05879200	-0.79386100
C	5.28143200	-0.85098700	-2.61405400
H	6.20961200	-0.64076400	-3.17139400
C	7.27761900	0.18799100	-0.38775100
H	8.19761200	-0.35909000	-0.65142100
C	6.49564300	-2.85463300	-0.48541300
H	7.02737500	-3.12283000	-1.41354000
C	7.50504800	-2.98288700	0.67209700
H	7.04259400	-2.72992200	1.63444900
H	7.86669700	-4.01739200	0.75167300
H	8.38165300	-2.33895900	0.54348900
C	5.35491200	-3.87261100	-0.28397500
H	4.63886500	-3.87645500	-1.11058200
H	5.75895600	-4.89019100	-0.18956600
H	4.79359900	-3.65259100	0.63131800
C	4.65289100	-2.11146800	-3.23946300
H	4.40515100	-1.92845000	-4.29403800
H	5.32359200	-2.97708100	-3.20654100
H	3.71937600	-2.38570000	-2.73369400
C	4.33280500	0.34901900	-2.81045900
H	4.11904900	0.49626300	-3.87821900
H	3.37476100	0.18194400	-2.30633100

H	4.74751800	1.28465800	-2.42350300
C	7.27258300	1.48453500	-1.21948800
H	8.16475900	2.08501600	-0.99237300
H	7.27368200	1.29322000	-2.29813500
H	6.39914000	2.10449800	-0.98758100
C	7.35276100	0.53666500	1.11267500
H	8.23211800	1.16257900	1.31998300
H	6.46466200	1.09717300	1.42634900
H	7.42288500	-0.35128900	1.74911200
C	2.44954400	-0.70894400	2.09131200
C	1.19734400	-0.09392000	1.86004600
C	2.64094000	-1.39207800	3.31042700
C	0.19258900	-0.16245000	2.82812200
C	1.63386600	-1.45450500	4.26784200
H	3.59834800	-1.87134500	3.48714500
C	0.40307200	-0.83854300	4.02886900
H	-0.75955400	0.31518000	2.62049300
H	1.80885000	-1.98507900	5.19949500
H	-0.38996300	-0.88746800	4.76950900
N	0.96662000	0.68266700	0.67822000
C	0.91512900	2.16740400	0.83125100
H	0.20778700	2.53984300	0.08874800
H	0.49380700	2.36067300	1.82128300
C	2.25084500	2.86549600	0.69297000
C	2.73193700	3.23545800	-0.57058900
C	3.00448500	3.18861200	1.82820700
C	3.94099500	3.92048200	-0.69287600
H	2.15428900	2.98080000	-1.45452500
C	4.21798400	3.86808700	1.70757900
H	2.63548100	2.91299900	2.81356700
C	4.68672800	4.23900800	0.44578800
H	4.29817100	4.21286300	-1.67679900
H	4.79045500	4.11601500	2.59740300
H	5.62543200	4.77801500	0.34963200
C	0.59528400	0.19736700	-0.54144400
O	0.55251100	0.79448900	-1.58589300
Cl	0.80009200	-1.80970900	-0.62668400
Pd	-1.50206300	-0.97065000	-0.38094600
P	-3.52737800	0.06892200	-0.26324500
C	-4.64737300	-0.37848900	1.21127400
C	-4.79654200	-0.59014500	-1.53414100
C	-3.55304100	1.90919600	-0.44247600
C	-4.77211000	-1.91315700	1.18100300
C	-4.09420900	0.11746400	2.54182200
O	-5.96995400	0.18823400	1.08681200
C	-6.16263100	0.08457800	-1.35287800
C	-4.27201000	-0.47819000	-2.95782800
O	-4.93487300	-1.99914400	-1.26338100
C	-4.49866900	2.76649900	0.14959600
C	-2.53379600	2.47381400	-1.23135700
H	-5.30395500	-2.23803500	2.08354600
H	-3.78242600	-2.38051200	1.16725400
C	-5.59071000	-2.35115000	-0.04392300
H	-4.78085600	-0.16407700	3.34843100
H	-3.11840300	-0.33951300	2.73267300
H	-3.98075500	1.20569900	2.55026000
C	-6.77004700	-0.30211400	0.00361100
H	-6.07742000	1.17132400	-1.44173000
H	-6.83641100	-0.27233100	-2.14137200
H	-3.31299000	-0.99654400	-3.05220300
H	-4.98983400	-0.93167100	-3.65137300
H	-4.13174700	0.57200500	-3.23511300
C	-4.41907100	4.14776100	-0.04147900
H	-5.29720300	2.35284000	0.75459000
C	-2.47097300	3.85376800	-1.43496900
H	-1.77891600	1.83054800	-1.67533200
C	-5.83281800	-3.84759000	-0.10902200
O	-6.86888900	-1.72100900	0.04833300
C	-8.16654600	0.24898700	0.22728900
C	-3.41050600	4.69538700	-0.83636000
H	-5.15302800	4.79619600	0.43035400
H	-1.68103000	4.26891500	-2.05536700
H	-4.87944800	-4.37792600	-0.18578900
H	-6.36236100	-4.18201100	0.78747100
H	-6.44044800	-4.07710700	-0.98845300
H	-8.84230500	-0.12073200	-0.54879100
H	-8.53283800	-0.07850400	1.20401300
H	-8.15164800	1.34247000	0.19941800
H	-3.35592800	5.77044100	-0.98631200

Zero-point correction = 0.859390 (Hartree/Particle)
Thermal correction to Energy = 0.913260
Thermal correction to Enthalpy = 0.914204
Thermal correction to Gibbs Free Energy = 0.766801
Sum of electronic and zero-point Energies = -3166.860181
Sum of electronic and thermal Energies = -3166.806311
Sum of electronic and thermal Enthalpies = -3166.805367
Sum of electronic and thermal Free Energies = -3166.952769
E(RM06L) = -3169.51315766

VIIa_PAPh

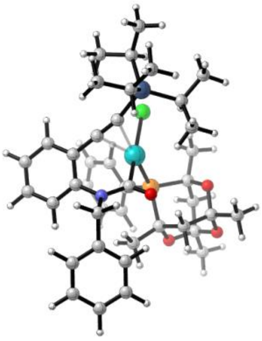

C	-2.21618700	1.04022000	1.06570200
C	-2.88821400	0.31557500	0.32091200
C	-1.48001000	1.94308200	1.89402200
C	-0.40991500	2.66694900	1.31218100
C	-1.77800400	2.11027500	3.25685900
C	0.33068900	3.54582600	2.10662300
C	-1.04231600	3.00382500	4.03001400
H	-2.59174000	1.53755600	3.68973300
C	0.00953300	3.72070300	3.45371900
H	1.16263500	4.08419200	1.66603200
H	-1.28427700	3.13536300	5.08040900
H	0.59395600	4.40979500	4.05671100
N	-0.13276200	2.52488700	-0.07288700
C	-0.19086800	3.75138100	-0.92434400
H	-0.69056400	3.44485000	-1.84668800
H	-0.83496000	4.46376800	-0.40210300
C	1.13735800	4.40302900	-1.25750300
C	1.97782500	3.85037200	-2.23593600
C	1.52484800	5.59670700	-0.63423100
C	3.18526700	4.46753700	-2.56356000
H	1.67085400	2.93863900	-2.73939100
C	2.73303000	6.21650900	-0.96161500
H	0.87000300	6.05226300	0.10562700
C	3.56849500	5.65021500	-1.92524200
H	3.82434100	4.03005600	-3.32623600
H	3.01587300	7.14348100	-0.46976600
H	4.50758900	6.13156100	-2.18473100
C	-0.13806800	1.29452700	-0.72019600
O	-0.04863700	1.23796900	-1.94051800
Pd	-0.56314000	-0.41419100	0.29568700
Si	-4.26210200	-0.57292100	-0.60787800
C	-5.25310100	0.88935900	-1.37206900
C	-5.20627500	-1.62739000	0.69002700
C	-3.44455500	-1.67583000	-1.93975200
H	-4.48673600	1.45783600	-1.92196200
C	-6.32750300	0.46921100	-2.39594700
C	-5.85517500	1.83737000	-0.31647400
H	-4.62769300	-2.56154400	0.72589400
C	-5.19481300	-1.04427300	2.11623400
C	-6.64906700	-1.97234400	0.26423800
H	-2.63214000	-2.17184600	-1.39004000
C	-2.81282500	-0.87316900	-3.09401100
C	-4.36515300	-2.78828800	-2.48193200
H	-6.78949200	1.35667600	-2.85037000
H	-5.91266200	-0.13486900	-3.20975600
H	-7.13229400	-0.10977700	-1.92956700
H	-5.10129300	2.19789500	0.39252000
H	-6.30517300	2.71714300	-0.79702700

H	-6.64715300	1.34605400	0.26182100
H	-4.17316700	-0.93127400	2.48918600
H	-5.69235300	-0.06714900	2.16557700
H	-5.72890700	-1.71529600	2.80300600
H	-7.08912500	-2.69324000	0.96661500
H	-7.29379600	-1.08495400	0.27069800
H	-6.70540400	-2.41578100	-0.73595700
H	-3.56977400	-0.33749600	-3.68114600
H	-2.07727100	-0.14210100	-2.74211100
H	-2.29098100	-1.55009500	-3.78447200
H	-3.81272500	-3.42330500	-3.18760400
H	-4.73941500	-3.43958000	-1.68460200
H	-5.23155000	-2.38704200	-3.02265200
Cl	-1.54937900	-2.44141700	1.33104900
P	1.70104800	-1.23073200	0.37853500
C	3.07763200	-0.35782300	-0.62648200
C	2.04001300	-2.92943500	-0.46457600
C	2.28498600	-1.32780400	2.13074900
C	2.66473800	-0.45550100	-2.10480500
C	3.32899200	1.07389900	-0.17052800
O	4.34263200	-1.03514400	-0.46811200
C	3.51439000	-3.31647600	-0.25573500
C	1.11322900	-4.04653900	-0.01628700
O	1.79426200	-2.72914700	-1.86459000
C	3.63571100	-1.18292100	2.50485400
C	1.32637400	-1.54446800	3.13685500
H	3.38402500	0.12678000	-2.69410400
H	1.66643300	-0.04562100	-2.26874100
C	2.73129900	-1.91295100	-2.57765600
H	4.13290400	1.50358700	-0.77797300
H	2.43785500	1.69063900	-0.29263600
H	3.63541200	1.10678800	0.87865600
C	4.43959600	-2.35815800	-1.00985100
H	3.76437500	-3.34351700	0.80848500
H	3.65664000	-4.32342900	-0.66534400
H	0.06901800	-3.79874900	-0.20448700
H	1.37895500	-4.95745200	-0.56595000
H	1.22418900	-4.23520500	1.05594400
C	4.00818700	-1.24819800	3.84823200
H	4.39370200	-1.02177000	1.74866500
C	1.71070300	-1.62042400	4.47684500
H	0.28224500	-1.67257400	2.87034700
C	2.40638900	-2.08753500	-4.04903900
O	4.06563900	-2.37917500	-2.38100100
C	5.90767100	-2.73727500	-0.94017600
C	3.04934600	-1.46824900	4.83847700
H	5.05433900	-1.12773400	4.11777400
H	0.95435600	-1.79728500	5.23678700
H	1.38604100	-1.74891700	-4.24821600
H	3.10539600	-1.50792800	-4.65847700
H	2.49227800	-3.14442600	-4.31555900
H	6.05700700	-3.72894700	-1.37594600
H	6.49652400	-2.00856500	-1.50379600
H	6.24929500	-2.74639700	0.09899300
H	3.34463400	-1.52165300	5.88317600

Zero-point correction = 0.860869 (Hartree/Particle)
Thermal correction to Energy = 0.914888
Thermal correction to Enthalpy = 0.915833
Thermal correction to Gibbs Free Energy = 0.771560
Sum of electronic and zero-point Energies = -3166.905116
Sum of electronic and thermal Energies = -3166.851096
Sum of electronic and thermal Enthalpies = -3166.850152
Sum of electronic and thermal Free Energies = -3166.994425
E(RM06L) = -3169.56761514

TS_AI_VIIa_PAPh

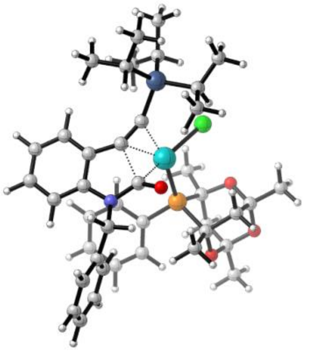

C	-1.82473400	1.39848400	0.13703200
C	-2.63917800	0.39963000	0.02887400
C	-1.44882500	2.60243700	0.84025500
C	-0.30590200	3.22422300	0.29936700
C	-2.04672300	3.12047100	1.99624300
C	0.20789900	4.39000300	0.86998500
C	-1.54009600	4.28867100	2.56304200
H	-2.90044900	2.61389000	2.43382500
C	-0.42693200	4.91989200	1.99544200
H	1.08624000	4.86902400	0.45402900
H	-2.01283100	4.70878200	3.44579500
H	-0.03833400	5.83142400	2.44079600
N	0.20305600	2.56866000	-0.83324100
C	0.78438800	3.33923900	-1.94674600
H	0.74408400	2.67068800	-2.81148000
H	0.11840800	4.18515000	-2.16277800
C	2.19942500	3.84907200	-1.74365600
C	2.61596700	4.96638100	-2.48099600
C	3.11518600	3.22001200	-0.89428700
C	3.92594900	5.43584400	-2.38564200
H	1.90970700	5.47026400	-3.13783700
C	4.42500500	3.69502400	-0.79233700
H	2.80081100	2.36864800	-0.29972500
C	4.83667300	4.79964100	-1.53907600
H	4.23206000	6.30190300	-2.96644700
H	5.11952400	3.19705500	-0.12203800
H	5.85629500	5.16655700	-1.45829200
C	-0.41663300	1.35456300	-1.13653500
O	-0.46331900	0.88789300	-2.26349600
Pd	-0.71291900	-0.56405000	0.00944600
Cl	-1.64363700	-2.76055000	0.42197800
Si	-4.37729800	-0.29814700	-0.24070400
C	-5.46829100	1.24925500	-0.61427600
C	-4.85438400	-1.27666900	1.33948800
C	-4.21663800	-1.42854100	-1.77817700
H	-4.90261600	1.78503700	-1.39249300
C	-6.85130100	0.91567800	-1.21166500
C	-5.62389600	2.21238100	0.57776500
H	-4.37434100	-2.25208000	1.18288900
C	-4.26388000	-0.69413200	2.63842400
C	-6.37310500	-1.49928900	1.48975600
H	-3.34097300	-2.04312000	-1.53437700
C	-3.89235900	-0.63960000	-3.06211800
C	-5.39696000	-2.39640600	-1.99308400
H	-7.39297700	1.83994300	-1.45705700
H	-6.77517100	0.32891300	-2.13242000
H	-7.47673800	0.35281400	-0.50947600
H	-4.65713700	2.55280600	0.96197400
H	-6.18938900	3.10696600	0.28142200
H	-6.16960400	1.74720500	1.40761300
H	-3.17121600	-0.64602400	2.59368800
H	-4.64688000	0.31288100	2.85006300
H	-4.53183400	-1.32720000	3.49553800
H	-6.57514000	-2.17270900	2.33396000
H	-6.90339100	-0.56110500	1.69437200
H	-6.82644600	-1.94861300	0.59929800
H	-4.70999000	0.03146900	-3.35564900
H	-2.98266400	-0.03781300	-2.95226600
H	-3.72675700	-1.32954300	-3.90104300
H	-5.18984400	-3.05559300	-2.84733400
H	-5.56191900	-3.04044800	-1.12213200
H	-6.33818900	-1.87709600	-2.21021700

P	1.59574300	-1.24134100	0.38669200
C	2.60139400	-1.71543500	-1.15789600
C	1.96198600	-2.91843800	1.25539000
C	2.54978000	0.01204100	1.35278700
C	1.86895400	-2.93554600	-1.74864800
C	2.72951500	-0.58064100	-2.16548700
O	3.94788800	-2.09789300	-0.81079900
C	3.48118200	-3.13308400	1.35771700
C	1.26298600	-3.01573500	2.60413800
O	1.42412900	-3.95619600	0.43323100
C	3.93658900	0.23096300	1.24906000
C	1.81705800	0.77997700	2.27550600
H	2.31821900	-3.16804200	-2.72144100
H	0.80569600	-2.72128200	-1.89414500
C	2.04064300	-4.15912600	-0.83417600
H	3.31237800	-0.93353500	-3.02401700
H	1.74327100	-0.26157200	-2.51327200
H	3.24812600	0.28119700	-1.73476600
C	4.09302200	-3.30189700	-0.04217300
H	3.96868500	-2.31878100	1.89954200
H	3.64876800	-4.06180200	1.91619600
H	0.18194800	-2.92534700	2.47390900
H	1.48304800	-3.99100100	3.05397300
H	1.62094300	-2.23185000	3.28068500
C	4.56472600	1.17978400	2.05880100
H	4.51822000	-0.34096600	0.53546400
C	2.44944600	1.72446200	3.08568600
H	0.74192100	0.63944800	2.35250000
C	1.42929900	-5.42604800	-1.40036800
O	3.44553000	-4.38943700	-0.68362500
C	5.57878300	-3.61311600	-0.02432200
C	3.82638900	1.92559500	2.98072200
H	5.63766800	1.33171200	1.97045300
H	1.86204600	2.30684800	3.78993700
H	0.34923500	-5.29510700	-1.50681400
H	1.87082600	-5.65645600	-2.37410200
H	1.62194900	-6.25615000	-0.71533900
H	5.75688800	-4.54577900	0.51800200
H	5.93701300	-3.72459400	-1.05124500
H	6.13237600	-2.80419000	0.46164800
H	4.32095400	2.66129800	3.60925900

Zero-point correction = 0.859175 (Hartree/Particle)
Thermal correction to Energy = 0.912602
Thermal correction to Enthalpy = 0.913546
Thermal correction to Gibbs Free Energy = 0.771250
Sum of electronic and zero-point Energies = -3166.875251
Sum of electronic and thermal Energies = -3166.821824
Sum of electronic and thermal Enthalpies = -3166.820880
Sum of electronic and thermal Free Energies = -3166.963176
E(RM06L) = -3169.54383974

VIa_PAPh

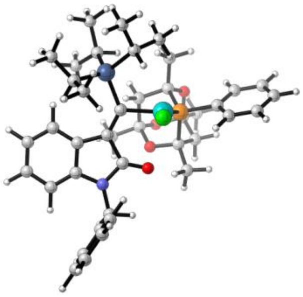

Pd	-0.57616300	0.36414400	-1.42906100
C	2.62079500	0.36475300	1.51806200
C	2.92063700	1.30499000	2.50538700
C	3.40588600	-0.81256000	1.47057700
C	3.98766500	1.08927000	3.38576800
H	2.33426200	2.20508100	2.60955800
C	4.47457800	-1.03955400	2.33031400
C	4.76211200	-0.06742100	3.29408400
H	4.21055600	1.83458700	4.14367400
H	5.07610900	-1.93826600	2.24839700
H	5.59382600	-0.22122900	3.97595700
C	1.58275100	0.25646100	0.45783100
C	0.65045000	1.12163700	-0.02374400
Si	0.48849300	3.06896600	0.11465700
C	2.31879400	3.69000500	0.11902100
H	2.86566900	2.97897400	0.74427800
C	-0.42735300	3.81088600	-1.41649100
H	0.02239500	3.28195700	-2.26510900
C	-0.56176300	3.47729900	1.69654800
H	-1.49041400	2.92099100	1.50440900
C	-0.17058200	5.31833700	-1.64541700
H	-0.64715800	5.62916200	-2.58523000
H	-0.58918200	5.94853600	-0.85312300
H	0.89193800	5.55891900	-1.73124700
C	-1.94238600	3.54057000	-1.47603600
H	-2.48818600	4.03151300	-0.65989800
H	-2.35819200	3.92408100	-2.41779500
H	-2.16497500	2.46787000	-1.43920500
C	2.53543600	5.08053600	0.75132300
H	3.60262300	5.34194200	0.72870000
H	1.99905000	5.87380200	0.21935900
H	2.21647100	5.11237000	1.79999000
C	2.97382500	3.59919400	-1.27701400
H	2.85187100	2.60850400	-1.72565400
H	2.55848000	4.32629700	-1.98235300
H	4.05023400	3.80454100	-1.19617000
C	-0.03759000	2.99027800	3.05864000
H	0.21026600	1.92473300	3.06501900
H	0.85727100	3.54482000	3.36741300
H	-0.79403700	3.15752900	3.83836100
C	-0.96150300	4.96423700	1.81721400
H	-1.61029700	5.10913600	2.69255100
H	-0.08960400	5.61357100	1.95275100
H	-1.51071000	5.32416600	0.94385500
Cl	0.80520700	1.01868800	-3.23620000
C	1.81661800	-1.11780800	-0.14919600
O	1.21455400	-1.67930500	-1.05855900
N	2.92137000	-1.66454300	0.47724400
C	3.46777900	-2.96714200	0.12128400
H	3.61771600	-3.54862300	1.03876500
H	2.68516500	-3.45906300	-0.46362300
C	4.75948800	-2.89867000	-0.67603600
C	4.81849900	-2.15737000	-1.86475400
C	5.89466500	-3.59810500	-0.25413100
C	5.99659500	-2.11433700	-2.60916700
H	3.93881500	-1.61874500	-2.20808000
C	7.07455900	-3.55943100	-1.00180500
H	5.85653600	-4.18123800	0.66424700
C	7.12800600	-2.81474600	-2.18001900
H	6.02995200	-1.53564900	-3.52831000
H	7.94926100	-4.10684000	-0.66031500
H	8.04486500	-2.77951900	-2.76242000
P	-2.33886500	-0.76176600	-0.24510900
C	-3.28137300	-0.08724500	1.26506900
C	-2.07634400	-2.48206500	0.55360900
C	-3.53313800	-1.01026000	-1.62483800
C	-2.24340800	-0.09841400	2.40252000
C	-3.89606200	1.28593500	1.02725600
O	-4.38278000	-0.93759500	1.63538300
C	-3.42561200	-3.07905800	0.98646800
C	-1.31731000	-3.44370400	-0.34742800
O	-1.26831700	-2.24793300	1.72018200
C	-4.93305900	-1.00852200	-1.48924600
C	-2.97775600	-1.17120900	-2.90906900
H	-2.67813000	0.40087300	3.27641700
H	-1.33657200	0.43709000	2.11003000
C	-1.89990800	-1.54580700	2.79739700
H	-4.37205000	1.63038200	1.95219200
H	-3.13801700	2.01078300	0.72711400
H	-4.65765300	1.24597800	0.24376300
C	-4.05087600	-2.24874800	2.10973200
H	-4.10935900	-3.15486100	0.13614200
H	-3.23960100	-4.09059300	1.36669300
H	-0.35783000	-3.02734100	-0.65708400
H	-1.14664200	-4.37904300	0.19900500
H	-1.91206500	-3.67073600	-1.23914700
C	-5.74827600	-1.15340900	-2.61284300

H	-5.38089200	-0.90297700	-0.50767100
C	-3.79789300	-1.31736200	-4.02878400
H	-1.89619400	-1.19395900	-3.04484600
C	-0.94911400	-1.64085000	3.97569900
O	-3.11618800	-2.18386800	3.18052900
C	-5.33356400	-2.83401700	2.67043600
C	-5.18565100	-1.30554300	-3.88229200
H	-6.82854800	-1.14779700	-2.49397000
H	-3.34868800	-1.43578800	-5.01054700
H	0.00595800	-1.16999600	3.72652600
H	-1.38140300	-1.14420800	4.84869900
H	-0.77601700	-2.69360400	4.21446600
H	-5.14114200	-3.82933300	3.08010100
H	-5.70833500	-2.18710200	3.46816500
H	-6.09138000	-2.90868300	1.88519200
H	-5.82640900	-1.41510400	-4.75289900

Zero-point correction = 0.863159 (Hartree/Particle)
Thermal correction to Energy = 0.916163
Thermal correction to Enthalpy = 0.917108
Thermal correction to Gibbs Free Energy = 0.775155
Sum of electronic and zero-point Energies = -3166.907910
Sum of electronic and thermal Energies = -3166.854905
Sum of electronic and thermal Enthalpies = -3166.853961
Sum of electronic and thermal Free Energies = -3166.995914
E(RM06L) = -3169.57582980

TS_RE_VIa_PAPh

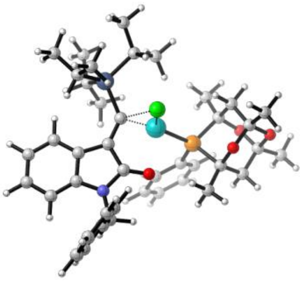

C	-3.89152200	1.35688100	1.88195500
C	-3.29045600	0.31384100	1.17452400
C	-3.83672100	-0.98661800	1.31600300
C	-4.94800000	-1.24836000	2.10862000
C	-5.53186100	-0.18007200	2.79925100
C	-5.00709700	1.10840900	2.68952600
H	-3.50508800	2.36359400	1.81829200
H	-5.36259800	-2.24831800	2.17693300
H	-6.40281300	-0.36086300	3.42303500
H	-5.46546900	1.93012400	3.23196200
N	-3.08625500	-1.89263300	0.56967700
C	-2.02877400	-1.26059300	-0.07699200
O	-1.21938800	-1.86110800	-0.77255600
C	-2.13787700	0.21889700	0.25625300
C	-3.30010100	-3.33139500	0.49358600
H	-3.46553000	-3.71659300	1.50684100
H	-2.35620600	-3.74381900	0.12438800
C	-4.44509000	-3.73962300	-0.41775800
C	-4.43894400	-3.36118600	-1.76787800
C	-5.50239100	-4.51751200	0.06423400
C	-5.47769400	-3.74869400	-2.61296300
H	-3.61266600	-2.76911900	-2.15308000
C	-6.54254300	-4.91022100	-0.78248400
H	-5.51216000	-4.82359500	1.10875100
C	-6.53317600	-4.52375300	-2.12260200
H	-5.46169100	-3.44957400	-3.65764300
H	-7.35834400	-5.51360600	-0.39296100
H	-7.34166500	-4.82505100	-2.78336600
C	-1.34162200	1.20539000	-0.28975100
Cl	-0.67272200	0.71091000	-2.36094700
Si	-1.49506700	3.14920700	-0.30661800
C	-0.20235000	3.91345900	-1.51109200
H	-0.33465500	3.33059100	-2.42941700
C	-1.24118800	3.77984700	1.51356900
H	-2.22555900	3.71538200	1.99553600
C	-3.29431400	3.49652100	-0.91217400
H	-3.90306800	2.73120300	-0.41207400
C	-0.46350400	5.39137100	-1.87663100
H	0.25127600	5.71324200	-2.64652400
H	-1.46691600	5.55242500	-2.28250600
H	-0.33768900	6.06446800	-1.02250600
C	1.26398900	3.73333900	-1.07693600
H	1.49682000	2.67977900	-0.86757800
H	1.94437400	4.07092000	-1.87130000
H	1.50360500	4.31228400	-0.17689000
C	-3.47309600	3.27470900	-2.42783100
H	-2.92504400	4.01773200	-3.01901500
H	-3.12690200	2.28457300	-2.74057000
H	-4.53351700	3.36214700	-2.70142000
C	-3.86195600	4.86531500	-0.48360900
H	-4.90956100	4.95836400	-0.80122900
H	-3.83919300	5.00431000	0.60366200
H	-3.31480600	5.70104800	-0.93502500
C	-0.27698900	2.91905800	2.35326500
H	-0.63102600	1.88815300	2.45557800
H	0.72587600	2.87979700	1.90989900
H	-0.16917900	3.33514400	3.36480700
C	-0.83427400	5.26708200	1.59975000
H	-0.82476800	5.59255600	2.64907700
H	0.17095500	5.43965200	1.20161100
H	-1.52418300	5.92465100	1.06053100
Pd	0.54209200	0.48284200	-0.13568400
P	2.70181900	-0.49156000	0.38471000
C	4.27710100	0.38470300	-0.22231000
C	3.10058400	-2.08847900	-0.58873000
C	2.94047100	-0.88173300	2.17400400
C	4.10885500	0.51763400	-1.74715700
C	4.49851800	1.73143600	0.45607700
O	5.46423600	-0.39402700	0.03969300
C	4.48915200	-2.62515300	-0.21836100
C	2.00342000	-3.12972800	-0.42733900
O	3.10474900	-1.70735700	-1.97598300
C	4.18219300	-0.95831500	2.83155300
C	1.77091200	-1.09748400	2.92484900
H	4.93491600	1.12484900	-2.13646900
H	3.16418000	1.00932900	-1.99702900
C	4.17804900	-0.86953600	-2.40743700
H	5.41298500	2.18902600	0.06182000
H	3.65690400	2.40210800	0.25947000
H	4.60913300	1.61857800	1.53858100
C	5.57781500	-1.63849500	-0.66454600
H	4.56070200	-2.82120900	0.85534300
H	4.64774400	-3.57104200	-0.75029600
H	1.03107400	-2.72462800	-0.72139900
H	2.23156100	-3.99643400	-1.05911200
H	1.94903900	-3.46525300	0.61468900
C	4.24340500	-1.24305900	4.19705600
H	5.09649400	-0.78906500	2.27448000
C	1.83627300	-1.39747400	4.28601900
H	0.80177200	-1.02368600	2.43623200
C	4.09726200	-0.82456000	-3.92151400
O	5.44162100	-1.44192100	-2.06625300
C	6.99120700	-2.13428500	-0.41895000
C	3.07444000	-1.46810700	4.92671600
H	5.21056000	-1.29010600	4.69097500
H	0.91977000	-1.56461800	4.84550800
H	3.13712500	-0.40262500	-4.23080800
H	4.91044700	-0.21374100	-4.32371300
H	4.18432600	-1.83993900	-4.31734800
H	7.15826000	-3.06785100	-0.96321900
H	7.70434800	-1.38415000	-0.77125500
H	7.15312900	-2.30752200	0.64903200
H	3.12799500	-1.69230500	5.98874800

Zero-point correction = 0.860712 (Hartree/Particle)
Thermal correction to Energy = 0.913727
Thermal correction to Enthalpy = 0.914671
Thermal correction to Gibbs Free Energy = 0.770301
Sum of electronic and zero-point Energies = -3166.888952
Sum of electronic and thermal Energies = -3166.835937
Sum of electronic and thermal Enthalpies = -3166.834993
Sum of electronic and thermal Free Energies = -3166.979363
E(RM06L) = -3169.54714954

***cis*-5a**

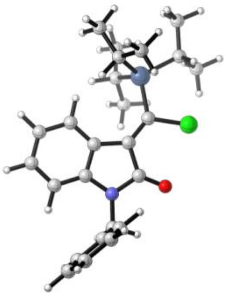

C	-0.06764500	2.26872500	0.26830300
C	0.53022600	1.10395200	-0.21748200
C	1.89355900	1.16949600	-0.59837100
C	2.63305600	2.34563800	-0.53399000
C	1.99620600	3.50005800	-0.06801800
C	0.66075800	3.46141000	0.33502400
H	-1.09200900	2.26878800	0.60999700
H	3.68029500	2.36209600	-0.81460700
H	2.55488700	4.42992100	-0.00827000
H	0.17979800	4.35911500	0.71183200
N	2.33260300	-0.08730200	-1.00808200
C	1.32696500	-1.04009100	-0.87544700
O	1.48501100	-2.22133200	-1.13502800
C	0.08512400	-0.29544700	-0.39576100
C	3.66213600	-0.42248200	-1.50229300
H	3.95525100	0.32401900	-2.24952900
H	3.54422000	-1.38340300	-2.01220500
C	4.72316700	-0.52756800	-0.41962600
C	4.57330800	-1.44908300	0.62655600
C	5.87231100	0.26823600	-0.46269400
C	5.55171000	-1.56114700	1.61318700
H	3.69088200	-2.08293100	0.65673600
C	6.85600100	0.15477700	0.52348300
H	6.00374400	0.97891600	-1.27661500
C	6.69566000	-0.75857800	1.56526400
H	5.42494400	-2.27977800	2.41859400
H	7.74305300	0.78103400	0.47687300
H	7.45761900	-0.84807300	2.33492900
C	-1.14401400	-0.86045900	-0.23801000
Cl	-1.25806100	-2.62262500	-0.42459900
Si	-2.87219300	-0.07381600	0.20857100
C	-4.21873600	-1.44068700	0.05533800
H	-3.78897700	-2.30613200	0.57647900
C	-3.21654300	1.40941800	-0.97989800
H	-2.65232500	2.26309300	-0.58593400
C	-2.64896500	0.46552000	2.04783800
H	-1.64586500	0.91173500	2.08509800
C	-5.54076800	-1.09911600	0.77803600
H	-6.22996500	-1.95233400	0.71629900
H	-5.39400800	-0.87836900	1.83967600
H	-6.05210100	-0.24164600	0.32823000
C	-4.51677000	-1.88574000	-1.39214200
H	-3.61247500	-2.17301800	-1.93645000
H	-5.18876600	-2.75477100	-1.39067300
H	-5.01790500	-1.09580900	-1.96360300
C	-2.61201500	-0.74461800	3.00479900
H	-3.58221300	-1.25009700	3.06804900
H	-1.86997900	-1.48978900	2.69642200
H	-2.34950500	-0.42042600	4.02082300
C	-3.63482700	1.54138500	2.54503300
H	-3.38791500	1.84194800	3.57243300
H	-3.60653800	2.44541800	1.92506700
H	-4.66998600	1.18127500	2.55522400
C	-2.71239100	1.17197400	-2.41939400
H	-1.63409600	0.99002700	-2.45288400
H	-3.20929300	0.31584500	-2.88998600
H	-2.92007600	2.05155300	-3.04377600
C	-4.69758700	1.84767200	-1.00369800
H	-4.80558700	2.76538800	-1.59762600
H	-5.33882700	1.09035200	-1.46674000
H	-5.09641500	2.05639500	-0.00509200

Zero-point correction = 0.515930 (Hartree/Particle)
Thermal correction to Energy = 0.546898
Thermal correction to Enthalpy = 0.547842
Thermal correction to Gibbs Free Energy = 0.452613
Sum of electronic and zero-point Energies = -1851.087186
Sum of electronic and thermal Energies = -1851.056217
Sum of electronic and thermal Enthalpies = -1851.055273
Sum of electronic and thermal Free Energies = -1851.150503
E(RM06L) = -1851.90101479

TS_Isom_Va_PAPh

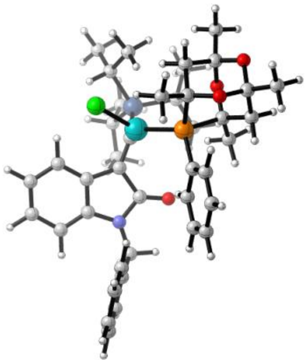

Pd	0.36711800	0.41267900	0.92522200
C	-2.90659800	1.44388400	0.93607700
C	-2.99242100	2.16569000	2.12490300
C	-4.05025300	0.80477600	0.41057000
C	-4.23300900	2.26397600	2.76330400
H	-2.10489400	2.61680900	2.55811700
C	-5.28613200	0.89181200	1.03877900
C	-5.36106200	1.63804900	2.22336000
H	-4.31641600	2.81894500	3.69283000
H	-6.15998200	0.38741200	0.64069600
H	-6.31582200	1.71734100	2.73571700
C	-1.78997400	1.17137400	0.02354600
C	-0.55370300	1.76837600	-0.03561300
Si	0.22631100	3.46615000	-0.46181200
C	1.40859600	3.12800700	-1.93402700
H	2.01721200	2.27838000	-1.59155400
C	-1.22581800	4.67603500	-0.86967000
H	-1.60033100	4.99338400	0.11628500
C	1.12473900	4.05970700	1.13408800
H	0.44292600	3.76386200	1.94545400
C	-0.73472400	5.94410400	-1.60642600
H	-0.43827600	5.71267400	-2.63590700
H	-1.54537600	6.68297000	-1.66431500
H	0.11517200	6.42908300	-1.11716400
C	1.32127100	5.58560100	1.24036300
H	0.37517300	6.13447700	1.18492800
H	1.78688000	5.83615000	2.20294600
H	1.98112800	5.97295500	0.45432000
C	2.38005600	4.28092500	-2.26181300
H	3.08143200	3.96854600	-3.04746700
H	1.85573500	5.16828600	-2.63303800
H	2.97746900	4.58635300	-1.39619000
C	-2.41869100	4.07755800	-1.63943900
H	-2.11555000	3.66616600	-2.60893200
H	-2.91954500	3.28529900	-1.07829300
H	-3.16655900	4.85772500	-1.83737400
C	2.45623300	3.32899600	1.38446400
H	3.21021800	3.59333300	0.63297000
H	2.86036300	3.59596000	2.36954000
H	2.32917800	2.24131700	1.37501400
C	0.65659000	2.68242000	-3.20554300
H	-0.03722000	1.85514700	-3.01781000
H	0.07950400	3.50899100	-3.63777000
H	1.36741100	2.35140200	-3.97475700
C	-2.35639300	0.28270600	-1.06480600
O	-1.78805800	-0.19807700	-2.03847200
N	-3.70571900	0.12573600	-0.77114400
C	-4.61724800	-0.60834200	-1.63265400
H	-5.41377900	0.06744000	-1.97045600
H	-4.02265800	-0.87870000	-2.51218600
C	-5.23162300	-1.84821600	-1.00150500
C	-4.50864000	-2.65339000	-0.11298400

C	-6.53530600	-2.22380000	-1.34736200
C	-5.07818000	-3.81341100	0.41460800
H	-3.50099100	-2.36907100	0.17636500
C	-7.10456700	-3.38677000	-0.82506600
H	-7.10972300	-1.60243200	-2.03166800
C	-6.37669500	-4.18509500	0.05892700
H	-4.50542300	-4.42407600	1.10729600
H	-8.11816200	-3.66323800	-1.10316900
H	-6.81987900	-5.08691200	0.47246600
Cl	0.67637300	0.34153000	3.32800600
P	1.76988800	-1.48193900	0.23808900
C	3.31534300	-1.84349900	1.29705800
C	2.78749500	-1.15775100	-1.34230300
C	0.88109600	-3.06676400	-0.06234200
C	4.11559000	-0.52941300	1.30675200
C	2.99190100	-2.32784700	2.70444100
O	4.12651600	-2.86980800	0.68675600
C	3.68137600	-2.36597200	-1.65958800
C	1.90878800	-0.78522900	-2.52556900
O	3.61607100	-0.01751300	-1.04680100
C	1.39080600	-4.33302200	0.27824100
C	-0.37667100	-2.98587500	-0.68578800
H	4.96470400	-0.65330600	1.98913300
H	3.49880900	0.29437000	1.67517100
C	4.67122800	-0.23066000	-0.09442900
H	3.93327700	-2.51527600	3.23387800
H	2.41430000	-1.57148300	3.24190600
H	2.41723000	-3.25782600	2.68831000
C	4.73938700	-2.54844600	-0.56576900
H	3.08158300	-3.27370400	-1.77066900
H	4.19549300	-2.17415000	-2.60914300
H	1.22366200	0.02719600	-2.27690500
H	2.54541200	-0.47394300	-3.36180500
H	1.31134000	-1.64603400	-2.84194100
C	0.64709800	-5.48474100	0.01506000
H	2.37352300	-4.42129100	0.72486400
C	-1.10617800	-4.14381900	-0.96174800
H	-0.77950400	-2.02058300	-0.97867100
C	5.54813900	1.00572100	-0.15276700
O	5.48324800	-1.33706500	-0.47911600
C	5.73060400	-3.66188700	-0.85050300
C	-0.60134600	-5.39474500	-0.60288100
H	1.05112700	-6.45548800	0.29024800
H	-2.07385100	-4.06248200	-1.44843300
H	4.97120400	1.88981900	0.13070600
H	6.39533100	0.89466700	0.52950900
H	5.92507600	1.13373100	-1.17112100
H	6.25953000	-3.46160800	-1.78638100
H	6.45693200	-3.71643500	-0.03519500
H	5.20949500	-4.62036900	-0.92918300
H	-1.17515700	-6.29461800	-0.80807500

Zero-point correction = 0.861345 (Hartree/Particle)
Thermal correction to Energy = 0.914128
Thermal correction to Enthalpy = 0.915072
Thermal correction to Gibbs Free Energy = 0.774020
Sum of electronic and zero-point Energies = -3166.902110
Sum of electronic and thermal Energies = -3166.849327
Sum of electronic and thermal Enthalpies = -3166.848383
Sum of electronic and thermal Free Energies = -3166.989436
E(RM06L) = -3169.57093241

Va_PAPh

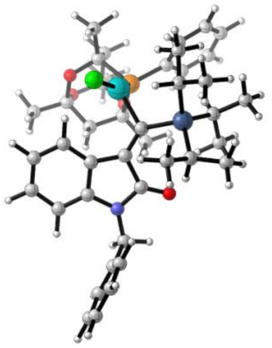

Pd	0.94977300	0.49442500	1.55061700
C	-1.86820700	-1.17744100	0.92347500
C	-1.40087800	-1.76090500	2.10317800
C	-2.93642100	-1.80176000	0.23891100
C	-1.96548500	-2.96285600	2.54936400
H	-0.62474500	-1.28012200	2.68594100
C	-3.50849800	-2.99260900	0.67109800
C	-3.00044000	-3.57471500	1.83842300
H	-1.60292000	-3.40870400	3.47116400
H	-4.33875800	-3.44215700	0.13715800
H	-3.43346000	-4.50290600	2.20131200
C	-1.55256600	0.08599800	0.21632600
C	-0.61140300	1.04491200	0.42254700
Si	-0.60420000	2.96550000	-0.00790800
C	-2.45428300	3.46890200	-0.21773700
H	-2.86188800	2.70573700	-0.88344400
C	0.13205400	3.93391900	1.49957500
H	-0.32257800	3.45435400	2.37568000
C	0.49550200	3.20774000	-1.57249000
H	1.42845700	2.68530600	-1.31421800
C	-0.27592900	5.42463000	1.53464600
H	0.14223900	5.89652200	2.43449900
H	0.10283100	5.98684800	0.67325400
H	-1.35849800	5.56615700	1.57279200
C	1.65610400	3.83416500	1.68897700
H	2.21069400	4.26277400	0.84526600
H	1.96036800	4.37731000	2.59405300
H	1.97791100	2.79560700	1.82008300
C	-2.68430600	4.81463900	-0.93907800
H	-3.76297700	4.99499000	-1.05051800
H	-2.26984300	5.67266800	-0.40039300
H	-2.25395700	4.81356500	-1.94648200
C	-3.24799000	3.39540700	1.10356100
H	-3.14234600	2.42146600	1.59461300
H	-2.93565000	4.15740900	1.82636900
H	-4.31854500	3.55568600	0.91169600
C	-0.05116300	2.56062400	-2.85893400
H	-0.28090200	1.50075600	-2.73082700
H	-0.97897000	3.04110600	-3.18929300
H	0.68127900	2.66178900	-3.67269100
C	0.87477900	4.67787900	-1.85307700
H	1.56091900	4.73034900	-2.70996500
H	-0.00118100	5.28578400	-2.10583700
H	1.37558100	5.15561100	-1.00576500
Cl	0.02976600	1.10308300	3.63195300
C	-2.50761700	0.10668000	-0.96817200
O	-2.56613900	0.88734100	-1.91145800
N	-3.29195800	-1.03510800	-0.87517100
C	-4.25902100	-1.40473000	-1.89702200
H	-4.01453300	-2.40580000	-2.27479700
H	-4.09779300	-0.69234000	-2.71295200
C	-5.70723900	-1.36807300	-1.43650100
C	-6.19993800	-0.28929900	-0.69118800
C	-6.58285000	-2.39929700	-1.79428100
C	-7.54093700	-0.24630200	-0.31085400
H	-5.52950600	0.51679800	-0.40550100
C	-7.92774600	-2.35564900	-1.41935300
H	-6.21101500	-3.24282500	-2.37281500
C	-8.40965900	-1.27878100	-0.67461000
H	-7.90869700	0.59523400	0.27011700
H	-8.59435300	-3.16542700	-1.70411700
H	-9.45405100	-1.24406000	-0.37662300

P	2.39776800	-0.49603000	-0.10285000
C	1.83055300	-1.88460800	-1.27982800
C	3.54125000	-1.67073600	0.89175200
C	3.53776800	0.61125500	-1.04269700
C	1.11935200	-2.93159600	-0.40799100
C	0.93911600	-1.38134000	-2.40876400
O	2.97241700	-2.50557900	-1.90154900
C	4.56603200	-2.32006500	-0.04788800
C	4.19283300	-0.96090700	2.07141000
O	2.69543900	-2.68728900	1.44825500
C	4.03202800	0.34108400	-2.33235500
C	3.94465100	1.79546400	-0.40334800
H	0.69155400	-3.69105800	-1.07316200
H	0.30856500	-2.48351700	0.16948100
C	2.12318400	-3.62347200	0.52335200
H	0.67836300	-2.22483900	-3.05774200
H	0.02061300	-0.94870400	-2.00449100
H	1.43725700	-0.62137800	-3.01603000
C	3.85309600	-3.24783100	-1.04274500
H	5.15262600	-1.56287700	-0.57499100
H	5.25044100	-2.92913200	0.55465700
H	3.43604200	-0.55155200	2.75068400
H	4.80056300	-1.67657600	2.63661100
H	4.83918400	-0.14665400	1.72842600
C	4.89677800	1.24008800	-2.95922300
H	3.76103000	-0.57788400	-2.83647000
C	4.82284800	2.68243500	-1.02714800
H	3.56220900	2.03501800	0.58297500
C	1.51279000	-4.73077200	1.35990000
O	3.13446300	-4.21506600	-0.29035600
C	4.79755700	-4.00092700	-1.96083900
C	5.29663100	2.40996700	-2.31111900
H	5.26213400	1.01891000	-3.95851700
H	5.12486400	3.58971700	-0.51152300
H	0.70508800	-4.33173100	1.97876300
H	1.11192000	-5.51034100	0.70613300
H	2.28286800	-5.16574000	2.00263100
H	5.47991200	-4.61732400	-1.36924100
H	4.21767100	-4.64825200	-2.62403400
H	5.37907400	-3.29859400	-2.56495700
H	5.97187600	3.10430100	-2.80352000

Zero-point correction = 0.862291 (Hartree/Particle)
Thermal correction to Energy = 0.915671
Thermal correction to Enthalpy = 0.916616
Thermal correction to Gibbs Free Energy = 0.773259
Sum of electronic and zero-point Energies = -3166.914806
Sum of electronic and thermal Energies = -3166.861425
Sum of electronic and thermal Enthalpies = -3166.860481
Sum of electronic and thermal Free Energies = -3167.003838
E(RM06L) = -3169.58407973

TS_RE_Va_PAPh

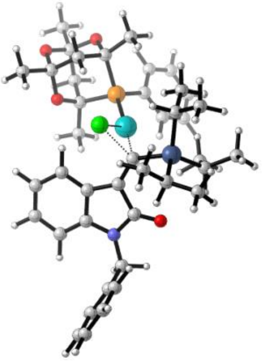

C	-0.96231500	-2.44508300	-1.13133000
C	-1.82608600	-1.65836300	-0.36330200
C	-2.85390200	-2.30041700	0.36839900
C	-3.03575200	-3.67786300	0.34978100
C	-2.15823200	-4.44276100	-0.42725400
C	-1.13502900	-3.83334700	-1.15722200
H	-0.15788200	-1.99018400	-1.69326900
H	-3.84361600	-4.14490400	0.90263600
H	-2.28131800	-5.52180700	-0.46194900
H	-0.45908200	-4.43963400	-1.75290100
N	-3.59926100	-1.34591400	1.07052000
C	-3.13656200	-0.06700900	0.80955900
O	-3.64395200	0.95238100	1.27064200
C	-1.94966000	-0.20809200	-0.11496200
C	-4.70783200	-1.61087100	1.97775300
H	-4.83827400	-0.68729300	2.55028700
H	-4.40720500	-2.40155600	2.67554100
C	-6.00655200	-1.98975900	1.28601300
C	-6.66658800	-3.17988400	1.60796800
C	-6.57794600	-1.13194500	0.33593900
C	-7.87476100	-3.51493800	0.99088300
H	-6.23555000	-3.84960400	2.34976000
C	-7.78083500	-1.46614200	-0.28422400
H	-6.07831200	-0.19821900	0.09134100
C	-8.43274400	-2.65945800	0.04102600
H	-8.37477600	-4.44435100	1.25045000
H	-8.21330700	-0.79298800	-1.01970900
H	-9.37043300	-2.91839000	-0.44332000
C	-1.26440200	0.90105500	-0.55122300
Cl	-0.29791600	0.51802900	-2.55147900
Si	-1.75981500	2.79858800	-0.52476700
C	-0.45136000	3.76808600	-1.55529800
H	-0.34687300	3.18111700	-2.47549300
C	-1.83676700	3.40647300	1.30852900
H	-2.83510600	3.10328000	1.63927500
C	-3.49592000	2.86431800	-1.35880300
H	-4.02935400	2.02606800	-0.89430600
C	-0.90080000	5.18447800	-1.97644400
H	-0.13802800	5.64177700	-2.62211300
H	-1.83765400	5.17424100	-2.54203200
H	-1.03692200	5.85244600	-1.11934100
C	0.94278300	3.83717900	-0.90435400
H	1.32151300	2.83923300	-0.63655900
H	1.66928900	4.29001900	-1.59420700
H	0.94297700	4.44228200	0.00974900
C	-3.46096800	2.61112500	-2.87820600
H	-2.95886800	3.42228600	-3.41917700
H	-2.94261900	1.68001600	-3.12919700
H	-4.48295000	2.54351500	-3.27679300
C	-4.30727400	4.13320900	-1.02607400
H	-5.30880600	4.06945100	-1.47418100
H	-4.44215200	4.26182800	0.05332500
H	-3.83965300	5.04427300	-1.41772500
C	-0.83026200	2.72155200	2.25169500
H	-0.99345500	1.63980800	2.29499500
H	0.20988900	2.89473800	1.94698700
H	-0.93691900	3.11166600	3.27421000
C	-1.73803500	4.93979400	1.45557400
H	-1.93092200	5.22792000	2.49853100
H	-0.74163700	5.31704800	1.19935600
H	-2.46488100	5.47248000	0.83322200
Pd	0.68505100	0.49573900	-0.20084300
P	2.92648700	-0.15838800	0.40098600
C	4.27299800	0.19302800	-0.89613500
C	3.21030700	-2.04601100	0.32137700
C	3.56332000	0.41021300	2.03673600
C	3.77360500	-0.46392500	-2.19549800
C	4.54221400	1.68250000	-1.07132000
O	5.53631900	-0.40682000	-0.53583200
C	4.67671100	-2.39687900	0.60205600
C	2.24543400	-2.79576900	1.22792000
O	2.88717700	-2.43164000	-1.02858900
C	4.91940600	0.60985800	2.35580300
C	2.59600100	0.67532900	3.02238600
H	4.45834100	-0.19002600	-3.00708600
H	2.76909600	-0.11409400	-2.45281800
C	3.79016800	-1.99366700	-2.05099700
H	5.32261800	1.82154400	-1.82798600
H	3.63521300	2.19712000	-1.40215900
H	4.88226000	2.14016800	-0.13764700
C	5.57707300	-1.83908000	-0.51024800
H	4.98933700	-2.01879000	1.57965800
H	4.77941200	-3.48876500	0.60782000
H	1.20948200	-2.57221800	0.95650800
H	2.40443100	-3.87530000	1.12296900

H	2.40828300	-2.51890800	2.27525300
C	5.28781400	1.05800700	3.62555500
H	5.68128200	0.42065400	1.60836600
C	2.96904900	1.10792900	4.29565200
H	1.54224200	0.55117700	2.78470600
C	3.38585900	-2.72569300	-3.31659900
O	5.12825200	-2.38397800	-1.74551300
C	7.03918800	-2.21795200	-0.36101500
C	4.31736100	1.30270300	4.59940500
H	6.33891200	1.21524200	3.85338800
H	2.20513600	1.30330500	5.04325600
H	2.36460500	-2.45335900	-3.59710500
H	4.06500100	-2.46396100	-4.13272900
H	3.43648100	-3.80410200	-3.14350300
H	7.14559500	-3.30618300	-0.37205500
H	7.60883900	-1.79592800	-1.19327100
H	7.43875300	-1.82756100	0.57956000
H	4.61012200	1.64880600	5.58705200

Zero-point correction = 0.860785 (Hartree/Particle)
Thermal correction to Energy = 0.913760
Thermal correction to Enthalpy = 0.914705
Thermal correction to Gibbs Free Energy = 0.770911
Sum of electronic and zero-point Energies = -3166.900646
Sum of electronic and thermal Energies = -3166.847671
Sum of electronic and thermal Enthalpies = -3166.846727
Sum of electronic and thermal Free Energies = -3166.990520
E(RM06L) = -3169.55776767

***trans*-5a**

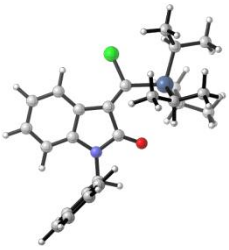

C	-1.00755600	3.41405400	-0.57910200
C	-0.99773600	2.11613100	-0.06055000
C	-2.21476900	1.57269300	0.41922700
C	-3.41081500	2.27709500	0.39006900
C	-3.39116000	3.57653000	-0.13148900
C	-2.20610200	4.13722100	-0.60883700
H	-0.10149600	3.86646800	-0.95658600
H	-4.33471100	1.83154500	0.74238800
H	-4.31424700	4.14855100	-0.16641900
H	-2.20825600	5.14557100	-1.01204500
N	-1.99709400	0.27512500	0.90051800
C	-0.67266400	-0.09191000	0.74530500
O	-0.21651900	-1.17603500	1.09470400
C	0.03742800	1.08031900	0.11030900
C	-2.98503500	-0.58770300	1.53571600
H	-3.52526500	-0.00391900	2.29016700
H	-2.40400800	-1.35523800	2.05643300
C	-3.96499900	-1.23075000	0.56899300
C	-3.49768800	-2.01332300	-0.49587200
C	-5.34409300	-1.07964000	0.74508100
C	-4.39539800	-2.62410400	-1.37012900
H	-2.42756700	-2.14708900	-0.63134200
C	-6.24592300	-1.69447100	-0.12754200
H	-5.71763400	-0.48085300	1.57363700
C	-5.77263500	-2.46585700	-1.18881500
H	-4.02086300	-3.22854700	-2.19197600
H	-7.31481000	-1.56682900	0.02164300
H	-6.47096700	-2.94322800	-1.87091400
C	1.36427600	1.02461400	-0.17763100
Cl	2.08321100	2.51294100	-0.83370200
Si	2.65137500	-0.43620400	0.07067300
C	4.31257200	0.17457300	-0.69613000
H	4.43598300	1.20126500	-0.32613000
C	2.00875700	-2.03776600	-0.78718000
H	1.29520000	-2.45579300	-0.07030500
C	2.79198800	-0.59843900	1.98277100
H	1.74663200	-0.60528500	2.31274200
C	5.54804800	-0.61725200	-0.21293000
H	6.46222000	-0.18432800	-0.64238500
H	5.65958000	-0.59516200	0.87547900
H	5.51555000	-1.66753500	-0.52217400
C	4.31499400	0.24324500	-2.23782600
H	3.48017200	0.82839500	-2.63523100
H	5.24266900	0.70910700	-2.59817600
H	4.26438300	-0.75633400	-2.68365300
C	3.47719800	0.62102400	2.63107500
H	4.53669000	0.69476500	2.35855900
H	2.99737200	1.56423400	2.34395300
H	3.43074100	0.55050000	3.72654800
C	3.41296700	-1.91963400	2.47782900
H	3.39171900	-1.96057900	3.57580800
H	2.85940100	-2.79045400	2.10997900
H	4.45968700	-2.03396700	2.17290100
C	1.23390700	-1.77639700	-2.09487200
H	0.38038800	-1.10845400	-1.94153300
H	1.86849500	-1.32804200	-2.86857100
H	0.84603600	-2.72031900	-2.50273300
C	3.10495100	-3.09801100	-1.02580100
H	2.65046400	-4.02889800	-1.39230800
H	3.83150400	-2.77853100	-1.78118700
H	3.65983400	-3.34519100	-0.11432900

Zero-point correction = 0.515862 (Hartree/Particle)
Thermal correction to Energy = 0.546888
Thermal correction to Enthalpy = 0.547832
Thermal correction to Gibbs Free Energy = 0.453029
Sum of electronic and zero-point Energies = -1851.100940
Sum of electronic and thermal Energies = -1851.069914
Sum of electronic and thermal Enthalpies = -1851.068970
Sum of electronic and thermal Free Energies = -1851.163773
E(RM06L) = -1851.91124356

c) reaction of carbamoyl chloride **3a** (R = TIPS), L = P*t*Bu_3

PC_3a

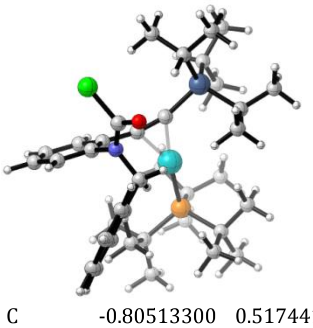

C	-0.80513300	0.51744100	-1.08906500
C	-0.58123900	1.57003200	-0.44673800
C	-1.42326600	-0.52663600	-1.86346200
C	-2.59060500	-1.18508100	-1.41058200
C	-0.87479900	-0.92012600	-3.10013600
C	-3.16107100	-2.21261000	-2.16392500
C	-1.46623200	-1.92596100	-3.85826000
H	0.02096900	-0.41698400	-3.44935000
C	-2.60918000	-2.57999600	-3.39001800
H	-4.04686500	-2.70916400	-1.78156000
H	-1.03063800	-2.20497300	-4.81364600
H	-3.06692800	-3.37369300	-3.97305300
N	-3.15281400	-0.84093500	-0.13720700
C	-2.51186300	-1.41044500	1.08032000
H	-1.43327500	-1.39903400	0.90275100
H	-2.72948100	-0.72095500	1.89764400

C	-2.99505600	-2.80838900	1.40776500
C	-2.21250400	-3.92575100	1.09220900
C	-4.23813800	-3.00318000	2.02945200
C	-2.65975300	-5.21532500	1.38935200
H	-1.24617700	-3.78497700	0.61358300
C	-4.68654000	-4.29053600	2.32381000
H	-4.84906700	-2.13995500	2.27838300
C	-3.89836500	-5.40003300	2.00496800
H	-2.03998200	-6.07304000	1.14115000
H	-5.64974200	-4.42819600	2.80810000
H	-4.24740000	-6.40233700	2.23908000
C	-4.24492700	-0.05773300	0.05051600
O	-4.74928400	0.23789100	1.10399700
Cl	-4.96023800	0.58436900	-1.49523000
Si	-0.75715000	3.28433300	0.27540100
C	-2.61782200	3.72052600	0.07922200
C	-0.19016500	3.15048200	2.10264300
C	0.38740800	4.41024500	-0.78089100
H	-3.14734300	2.83123700	0.45289300
C	-3.10865900	4.91891800	0.91639400
C	-3.00860200	3.89295800	-1.40266400
H	0.78802800	2.65081100	2.03261300
C	-1.11279100	2.22887200	2.92421300
C	0.03226200	4.49225900	2.82798500
H	0.14287300	4.13406200	-1.81830800
C	1.87929200	4.09187600	-0.56508200
C	0.12903900	5.92392400	-0.63908900
H	-2.59991400	5.85146500	0.64500200
H	-4.18366700	5.07857000	0.75611800
H	-2.96143200	4.76093800	1.99016200
H	-2.71905400	3.02683000	-2.00746900
H	-4.09519000	4.01243000	-1.50321200
H	-2.54030600	4.78193800	-1.84353200
H	-0.71884000	2.08906000	3.94045600
H	-2.12301100	2.64472500	3.02292500
H	-1.20435900	1.23815000	2.46520800
H	-0.89228400	5.07442500	2.91450000
H	0.39918100	4.31848200	3.84917300
H	0.77176100	5.12053700	2.31957800
H	2.08674000	3.02276600	-0.69995600
H	2.50361500	4.65029500	-1.27660500
H	2.20898000	4.37106300	0.44396000
H	0.31750600	6.28025600	0.38047800
H	0.79545500	6.48927100	-1.30527100
H	-0.89899700	6.19670400	-0.89943300
Pd	1.22172400	0.30814300	-0.28937500
P	3.25222700	-0.88675600	0.05146800
C	3.09851800	-2.70762600	-0.63106200
C	4.75205400	-0.02620500	-0.85285100
C	3.66030700	-0.98005700	1.95529700
C	4.16081400	-3.70753200	-0.13062200
C	1.69263700	-3.24150200	-0.26485000
C	3.14026700	-2.68208900	-2.17485500
C	6.02759600	-0.87636200	-1.02116300
C	4.26396500	0.43441300	-2.24829600
C	5.12778000	1.26796000	-0.09680300
C	3.34704800	0.40618300	2.56960000
C	2.68203700	-1.96152700	2.63831700
C	5.10012600	-1.38523800	2.32975600
H	4.00136600	-4.67486800	-0.62676400
H	5.18142900	-3.39014600	-0.35812600
H	4.09291500	-3.88709800	0.94523000
H	0.91315300	-2.57479200	-0.64677500
H	1.56124000	-4.22892800	-0.72869000
H	1.54091800	-3.36113600	0.80820200
H	4.13275400	-2.45983700	-2.57255400
H	2.86012400	-3.67656200	-2.54661600
H	2.42561300	-1.96192100	-2.58575100
H	5.87175300	-1.74239900	-1.66938400
H	6.42681000	-1.23299200	-0.06823100
H	6.80696400	-0.26146600	-1.49212700
H	5.05230100	1.04099300	-2.71464000
H	4.04721100	-0.39257400	-2.92508300
H	3.36021000	1.04655300	-2.16512100
H	5.83362100	1.83888800	-0.71432500
H	4.25548600	1.90579500	0.07806400
H	5.62112800	1.07804600	0.85907600
H	4.01144600	1.19715800	2.22094400
H	3.45689600	0.34249200	3.66082200
H	2.31675100	0.70491600	2.34719800
H	1.64176600	-1.73550400	2.38253100
H	2.88511500	-3.00752600	2.39757700
H	2.78619400	-1.85650400	3.72625700
H	5.84132700	-0.65384700	1.99795500
H	5.38257500	-2.36121300	1.92677200
H	5.18054700	-1.44790500	3.42375500

Zero-point correction= 0.886185 (Hartree/Particle)
Thermal correction to Energy= 0.940446
Thermal correction to Enthalpy= 0.941390
Thermal correction to Gibbs Free Energy= 0.793395
Sum of electronic and zero-point Energies= -2792.365316
Sum of electronic and thermal Energies= -2792.311055
Sum of electronic and thermal Enthalpies= -2792.310111
Sum of electronic and thermal Free Energies= -2792.458106
E(RM06L) = -2794.95158613

TS_OA_3a

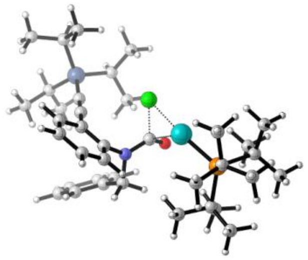

C	2.88197100	-0.18347800	1.24277700
C	3.78030700	-0.52899700	0.49016500
Si	5.14059300	-1.21382400	-0.55554800
C	4.48011300	-1.59401800	-2.32038900
H	5.38591500	-1.60742600	-2.94949200
C	6.59426800	0.05064900	-0.63428000
H	7.49089900	-0.58192300	-0.73984600
C	5.75411300	-2.83030200	0.29915200
H	6.23688700	-3.40332500	-0.51001000
C	6.81453000	-2.60677400	1.39494500
H	6.40271000	-2.03873400	2.23856800
H	7.16057400	-3.56996900	1.79481600
H	7.69576000	-2.06914300	1.02888600
C	4.60660100	-3.69010800	0.86666400
H	3.85260400	-3.94096800	0.11528600
H	4.99714700	-4.63334300	1.27344500
H	4.09248800	-3.16580500	1.68028000
C	3.80068500	-2.96863700	-2.47421200
H	3.50943600	-3.13281900	-3.52077300
H	4.45575100	-3.79708800	-2.18313100
H	2.88578700	-3.03141900	-1.87300600
C	3.54558300	-0.49451200	-2.86440100
H	3.28818000	-0.69617100	-3.91348600
H	2.60760800	-0.45829600	-2.29977300
H	3.99388400	0.50247500	-2.81891100
C	6.57455600	1.00521000	-1.84321500
H	7.48743700	1.61722700	-1.85897700
H	6.52159700	0.47313600	-2.79935300
H	5.72456000	1.69523300	-1.79360800
C	6.74582900	0.86610500	0.66597200

H	7.64316700	1.49931800	0.62229900
H	5.88185600	1.52373500	0.81528500
H	6.83328500	0.23167300	1.55384200
C	1.87255700	0.13141100	2.20049000
C	0.59770100	0.62818500	1.84069300
C	2.13438300	-0.10716000	3.56561200
C	-0.36286600	0.87063900	2.82555600
C	1.17202500	0.14199000	4.53863000
H	3.10931800	-0.49493800	3.84264200
C	-0.08359000	0.63008600	4.17010100
H	-1.33791400	1.23668700	2.52131700
H	1.40043400	-0.04925600	5.58334600
H	-0.84402800	0.81754200	4.92270100
N	0.29911200	0.97149200	0.48130200
C	0.28077200	2.41967900	0.12752000
H	-0.42136300	2.53211100	-0.70017400
H	-0.12936300	2.94567400	0.99424100
C	1.62539600	3.00772300	-0.24357300
C	2.08503000	2.94568300	-1.56617700
C	2.40605700	3.66174200	0.71768700
C	3.30020200	3.53276600	-1.91930200
H	1.48487800	2.43486000	-2.31381300
C	3.62624300	4.24276100	0.36800500
H	2.05323100	3.72269600	1.74474200
C	4.07370600	4.18276600	-0.95325400
H	3.64023200	3.49028000	-2.95066800
H	4.22037100	4.75037900	1.12321700
H	5.01733700	4.64483700	-1.23075300
C	-0.10117700	0.10798700	-0.49976800
O	-0.17659200	0.32946000	-1.68023400
Cl	0.09762900	-1.79307700	0.09844400
Pd	-2.21361200	-0.93564100	0.10091000
P	-4.37180300	-0.16246600	-0.27000600
C	-4.51398500	1.77502400	-0.43168900
C	-5.00356600	-0.95616900	-1.93889000
C	-5.55320600	-0.74518200	1.17011900
C	-5.93797800	2.36142700	-0.35028000
C	-3.64692500	2.41370000	0.67825400
C	-3.87406800	2.21810200	-1.76646900
C	-3.86197700	-0.86055800	-2.98084000
C	-5.23722600	-2.46777400	-1.72006400
C	-6.28522500	-0.34510500	-2.54026600
C	-5.12330100	-2.17343000	1.58654800
C	-5.31348400	0.13600500	2.41581500
C	-7.06329000	-0.74488500	0.85704500
H	-6.39893000	2.20762300	0.62892600
H	-5.88680100	3.44704300	-0.51266800
H	-6.60847600	1.95100300	-1.10910500
H	-4.03055500	2.23750800	1.68366400
H	-2.62330400	2.03129300	0.63332600
H	-3.61723600	3.50104000	0.52394000
H	-4.48642100	1.96642500	-2.63502200
H	-3.76706600	3.31110800	-1.75633200
H	-2.87802800	1.78505600	-1.90451500
H	-2.93577900	-1.29602700	-2.59256700
H	-4.16015900	-1.42156100	-3.87716200
H	-3.64329900	0.16016700	-3.29429500
H	-5.40296200	-2.93852500	-2.69794600
H	-4.36415700	-2.94836400	-1.26620300
H	-6.11641400	-2.68305600	-1.10852500
H	-6.14513100	0.69162800	-2.85702400
H	-6.56547600	-0.91648200	-3.43588500
H	-7.13511000	-0.37954800	-1.85385700
H	-5.70051100	-2.47094400	2.47292400
H	-5.30127300	-2.92257100	0.81492300
H	-4.05879300	-2.20187100	1.84437500
H	-4.24933200	0.19643900	2.66682600
H	-5.70958600	1.14849500	2.30782800
H	-5.82917300	-0.32099600	3.27070800
H	-7.43344800	0.23933200	0.55851600
H	-7.32821400	-1.45904600	0.07348700
H	-7.61547800	-1.04147300	1.75958000

Zero-point correction = 0.885851 (Hartree/Particle)
Thermal correction to Energy = 0.939354
Thermal correction to Enthalpy = 0.940298
Thermal correction to Gibbs Free Energy = 0.795142
Sum of electronic and zero-point Energies = -2792.334611
Sum of electronic and thermal Energies = -2792.281109
Sum of electronic and thermal Enthalpies = -2792.280165
Sum of electronic and thermal Free Energies = -2792.425320
E(RM06L) = -2794.92316411

VIIa

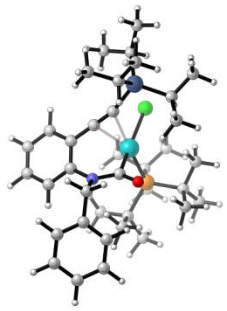

C	1.34592600	-1.08744300	1.26342500
C	2.23587900	-0.74788800	0.47381800
C	0.33682700	-1.61531000	2.12567400
C	-0.85198000	-2.09854200	1.52588100
C	0.49043000	-1.67112700	3.52077200
C	-1.85398300	-2.64047200	2.33550800
C	-0.51107000	-2.22739700	4.31229200
H	1.40181100	-1.28416500	3.96488800
C	-1.67857700	-2.71482400	3.71893200
H	-2.77040400	-2.99708700	1.87817100
H	-0.38203300	-2.27797100	5.38938300
H	-2.46463900	-3.14292500	4.33456800
N	-0.98945200	-2.05189100	0.11502100
C	-1.21956700	-3.33371600	-0.61350400
H	-0.63320600	-3.25636200	-1.53244600
H	-0.78915200	-4.12586300	0.00544600
C	-2.65805200	-3.67182500	-0.95686000
C	-3.30498500	-3.03367500	-2.02600200
C	-3.34794200	-4.66522100	-0.24950000
C	-4.61747900	-3.36710700	-2.36060500
H	-2.76595900	-2.28033600	-2.59226000
C	-4.66183600	-5.00181600	-0.58416800
H	-2.84934300	-5.18830400	0.56391100
C	-5.30133000	-4.34952200	-1.63898500
H	-5.10486900	-2.86605300	-3.19298400
H	-5.18022400	-5.77731400	-0.02633200
H	-6.32225100	-4.61109500	-1.90424500
C	-0.64811200	-0.93087400	-0.63772700
O	-0.68843400	-0.97283700	-1.86065000
Pd	0.24398700	0.68361800	0.20089100
Si	3.86530000	-0.57515500	-0.44995500
C	4.29506200	-2.41182600	-0.84267800
C	5.08853500	0.31908000	0.72944200
C	3.54585400	0.44597200	-2.03519400
H	3.39306600	-2.78236000	-1.35466300
C	5.47721500	-2.60004700	-1.81551900
C	4.49253500	-3.27724300	0.41732800
H	4.88709200	1.38337100	0.54309400
C	4.82111600	0.07371700	2.22697500
C	6.57056500	0.04528300	0.39742300
H	2.95013600	1.29255800	-1.66666800
C	2.70708100	-0.30581300	-3.08735600
C	4.82264900	1.03597100	-2.66790200
H	5.60641700	-3.66409300	-2.05812600
H	5.32758000	-2.06660400	-2.76012700
H	6.42183200	-2.25143000	-1.38352800
H	3.63929400	-3.21100900	1.10173400
H	4.61818700	-4.33469500	0.14594100
H	5.38901000	-2.97917300	0.97460000
H	3.81763900	0.40477700	2.50772500
H	4.92612600	-0.98527700	2.49601700
H	5.54191300	0.63616100	2.83645100
H	7.21991400	0.69094900	1.00427300
H	6.84965600	-0.99122400	0.62298400
H	6.81520000	0.23296600	-0.65392000
H	3.24067400	-1.17675100	-3.48907300

H	1.74765500	-0.65188400	-2.68841200
H	2.48511700	0.35297200	-3.93850400
H	4.56069100	1.66553100	-3.52921700
H	5.37858400	1.66490600	-1.96412600
H	5.50466700	0.25898600	-3.03531000
Cl	1.98447100	2.36359300	0.82622600
P	-1.68338700	2.24043000	-0.04885300
C	-1.51942300	3.70739300	1.25080700
C	-3.46380400	1.46642200	0.21613700
C	-1.58513000	2.97353500	-1.84817300
C	-1.06481500	3.09486700	2.59743600
C	-2.81703600	4.51648300	1.47633700
C	-0.43679300	4.72996500	0.83482400
C	-3.59871600	0.11154300	-0.51045300
C	-3.67030800	1.14340800	1.71402200
C	-4.62412100	2.35825500	-0.28458200
C	-0.09460500	3.23058800	-2.16901800
C	-2.07946700	1.93318000	-2.87824900
C	-2.38423200	4.27670700	-2.06456600
H	-0.98413600	3.90603700	3.33354100
H	-1.76563400	2.35998000	2.99735900
H	-0.08089200	2.63071700	2.50284900
H	-3.62847800	3.93672200	1.91849900
H	-2.58347400	5.32422000	2.18206800
H	-3.18452700	4.98839000	0.56094400
H	-0.72855200	5.32846900	-0.03086000
H	-0.30733900	5.42688500	1.67372300
H	0.52837000	4.26030000	0.65080800
H	-4.64759900	-0.20589700	-0.44352600
H	-3.33220000	0.14239500	-1.56469400
H	-3.00102400	-0.66059200	-0.02995300
H	-4.59063200	0.55253600	1.81086600
H	-2.85087400	0.53668500	2.11296500
H	-3.78786000	2.02611900	2.34371500
H	-4.64923000	2.41198900	-1.37614600
H	-5.56945300	1.89817400	0.03288800
H	-4.61002100	3.37415400	0.10745200
H	0.48264900	2.30216700	-2.13338100
H	-0.02712600	3.63322400	-3.18876500
H	0.38297600	3.94018700	-1.49420400
H	-1.83470500	2.31061600	-3.88003100
H	-1.58673800	0.96501500	-2.75906800
H	-3.16344000	1.79327700	-2.84884900
H	-2.02014500	5.10776600	-1.45855700
H	-2.27462800	4.57370400	-3.11618700
H	-3.45250300	4.15561200	-1.87327000

Zero-point correction = 0.888285 (Hartree/Particle)
Thermal correction to Energy = 0.941621
Thermal correction to Enthalpy = 0.942566
Thermal correction to Gibbs Free Energy = 0.802270
Sum of electronic and zero-point Energies = -2792.374511
Sum of electronic and thermal Energies = -2792.321174
Sum of electronic and thermal Enthalpies = -2792.320230
Sum of electronic and thermal Free Energies = -2792.460526
E(RM06L) = -2794.97537937

TS_AI_VIIa

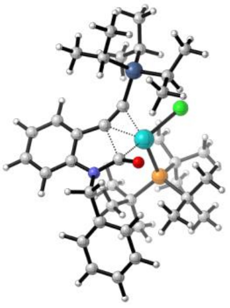

C	1.21984400	-1.24416900	0.58428700
C	2.10835800	-0.45305300	0.08986400
C	0.78719100	-2.16446900	1.60445600
C	-0.37903200	-2.87520800	1.25584700
C	1.36495300	-2.34346100	2.86782400
C	-0.94323400	-3.78923400	2.14753700
C	0.81184400	-3.26956100	3.75052700
H	2.24019000	-1.76450000	3.14346900
C	-0.33095100	-3.99049700	3.38636400
H	-1.84619800	-4.33249400	1.89108100
H	1.26791300	-3.42776500	4.72309700
H	-0.76186200	-4.70700200	4.07986800
N	-0.86477100	-2.55745100	-0.01916700
C	-1.51843700	-3.56964200	-0.88466500
H	-0.93297900	-3.60612600	-1.80834200
H	-1.40659600	-4.52953200	-0.37467200
C	-2.97519000	-3.31659600	-1.21985500
C	-3.32123800	-2.73065200	-2.44448800
C	-3.99950400	-3.70402900	-0.34490400
C	-4.66086400	-2.52857200	-2.78304800
H	-2.53358800	-2.42873800	-3.12872300
C	-5.33855300	-3.49970000	-0.67830400
H	-3.75267000	-4.17702700	0.60277700
C	-5.67250300	-2.91023900	-1.90005400
H	-4.91251700	-2.07612900	-3.73839700
H	-6.12038700	-3.80830400	0.01055300
H	-6.71528500	-2.75610300	-2.16410800
C	-0.28914700	-1.45437800	-0.63805600
O	-0.31832000	-1.28969000	-1.84450800
Pd	0.29628400	0.71152600	-0.05895400
Cl	1.62886000	2.68642400	-0.61744800
P	-1.84347400	2.01570800	0.34641200
C	-1.51930200	3.79150800	1.11333800
C	-3.05016200	1.09783700	1.57844000
C	-2.74611400	2.24613300	-1.36030800
C	-0.39563400	3.65874100	2.16916500
C	-2.75650000	4.44018800	1.77485100
C	-1.00738300	4.79190100	0.05002400
C	-3.04505200	-0.40823900	1.25159300
C	-2.49983000	1.19117600	3.02023200
C	-4.51700200	1.58028800	1.56061900
C	-1.66851400	2.61877800	-2.40704800
C	-3.34191500	0.89985100	-1.82192200
C	-3.87319700	3.29983400	-1.39074700
H	-0.20082900	4.65346000	2.59266300
H	-0.65716300	2.99905700	2.99780100
H	0.52893500	3.30082000	1.71196700
H	-3.11852000	3.89688200	2.64929000
H	-2.46803500	5.44182100	2.12064500
H	-3.58996600	4.56857400	1.07900500
H	-1.77166900	5.06563700	-0.68092800
H	-0.72482400	5.71471200	0.57470100
H	-0.12189400	4.42375400	-0.46730800
H	-3.76134700	-0.91126600	1.91547100
H	-3.33019900	-0.64405500	0.22783400
H	-2.06282900	-0.83992900	1.44140100
H	-3.07932500	0.50627900	3.65360800
H	-1.45244500	0.87525300	3.07165200
H	-2.58444700	2.18530900	3.46093700
H	-5.01124900	1.34829500	0.61367200
H	-5.06889600	1.04700000	2.34692600
H	-4.63015300	2.64792700	1.75203200
H	-0.88521900	1.85737300	-2.46120300
H	-2.15214200	2.68349100	-3.39144300
H	-1.18316700	3.57446500	-2.21161900
H	-3.68842100	1.01664700	-2.85750500
H	-2.59930100	0.09854500	-1.81900500
H	-4.20569100	0.58525500	-1.23135300
H	-3.51734300	4.30990700	-1.18057000
H	-4.30547600	3.31602500	-2.40060700
H	-4.68506900	3.07240600	-0.69567100
Si	3.88322500	-0.18000700	-0.51277500
C	4.71720800	-1.91274200	-0.33592800
C	4.66914800	1.20107400	0.56228200
C	3.71686800	0.32407900	-2.35385200
H	4.00389800	-2.59776200	-0.82034900
C	6.05562800	-2.04676900	-1.09158900
C	4.88351100	-2.38592200	1.12069100
H	4.31155800	2.12404300	0.08724300
C	4.16004300	1.22393200	2.01712400
C	6.21118000	1.20596400	0.52960800
H	2.95080700	1.10857400	-2.32270400

C	3.17781700	-0.81887700	-3.23644000
C	4.98337600	0.95885600	-2.96165500
H	6.44119600	-3.07247700	-1.00503000
H	5.95491700	-1.82593300	-2.15865300
H	6.82376100	-1.37907700	-0.68543800
H	3.93240800	-2.39789700	1.66194100
H	5.29011900	-3.40661000	1.15221800
H	5.57743900	-1.74566800	1.67842200
H	3.07344500	1.34938000	2.05779400
H	4.42220500	0.30661200	2.56050600
H	4.61081700	2.06302500	2.56506600
H	6.59741100	2.09138800	1.05307600
H	6.63265800	0.32749300	1.03318000
H	6.61291400	1.22882700	-0.48943900
H	3.86877600	-1.67050700	-3.28385000
H	2.20943400	-1.18647500	-2.87806100
H	3.03030300	-0.46668100	-4.26672800
H	4.78102700	1.28921700	-3.98983800
H	5.30910000	1.84037200	-2.39805400
H	5.82742500	0.26021100	-3.00976900

Zero-point correction = 0.886964 (Hartree/Particle)
Thermal correction to Energy = 0.939773
Thermal correction to Enthalpy = 0.940717
Thermal correction to Gibbs Free Energy = 0.802160
Sum of electronic and zero-point Energies = -2792.341901
Sum of electronic and thermal Energies = -2792.289093
Sum of electronic and thermal Enthalpies = -2792.288148
Sum of electronic and thermal Free Energies = -2792.426706
E(RM06L) = -2794.94451562

VIa

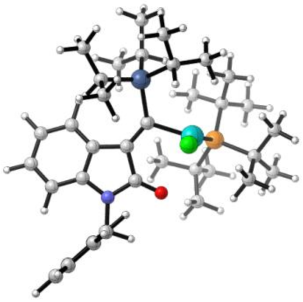

Pd	-1.03267900	-0.35968400	-1.07839300
C	2.14072400	0.81571800	1.61419700
C	2.18178600	1.89183400	2.50235800
C	3.19750800	-0.12410600	1.66568200
C	3.27937800	2.05837000	3.35560600
H	1.36419600	2.59679600	2.55409600
C	4.30212700	0.03268100	2.49521100
C	4.33643800	1.14755800	3.34001400
H	3.30187800	2.90545900	4.03503500
H	5.11476900	-0.68569800	2.48607900
H	5.18923200	1.29273500	3.99735500
P	-2.72377400	-1.53518000	0.32032500
C	-4.23291800	-0.56058000	1.07591500
C	-3.39217700	-2.65735800	-1.13508200
C	-1.99623000	-2.68894700	1.71241500
C	1.14738600	0.32368300	0.62447900
C	0.07223900	0.90572800	0.02068700
C	-1.10217700	-1.82245400	2.63102600
H	-1.66285900	-1.09624800	3.22042200
H	-0.58652700	-2.48509700	3.33847200
H	-0.34338400	-1.28283100	2.06281800
C	-1.07061100	-3.75354000	1.08170300
H	-0.55765800	-4.27844200	1.89867300
H	-1.61810200	-4.51079400	0.51603700
H	-0.30515700	-3.30794300	0.44291600
C	-3.04087800	-3.41841300	2.58312100
H	-2.50643400	-4.07768200	3.28010800
H	-3.64119100	-2.73669400	3.19007100
H	-3.71818800	-4.04583600	1.99892500
C	-3.81483500	0.07044800	2.42294900
H	-4.59753800	0.77488100	2.73259700
H	-3.71144700	-0.66441900	3.22365600
H	-2.88057400	0.63343400	2.34405800
C	-4.58564000	0.61616000	0.14126800
H	-4.97262900	0.30201200	-0.82767600
H	-5.36444800	1.22255500	0.62252000
H	-3.72319000	1.25806600	-0.03706700
C	-5.51104000	-1.39610600	1.30457400
H	-5.95246600	-1.74734400	0.36890700
H	-5.35159700	-2.25984900	1.95258500
H	-6.26063400	-0.75699100	1.79055700
C	-4.15785700	-3.92234200	-0.69665300
H	-5.02311900	-3.69718000	-0.06899000
H	-4.52973400	-4.43344000	-1.59500900
H	-3.52471600	-4.63479100	-0.16382500
C	-2.18226700	-3.07969300	-2.00728800
H	-2.54690500	-3.73051300	-2.81367900
H	-1.69764400	-2.22454600	-2.50017800
H	-1.40997600	-3.62116200	-1.46332400
C	-4.31365400	-1.83474100	-2.06425400
H	-5.28507200	-1.61347000	-1.61690300
H	-3.85099500	-0.89544800	-2.38438000
H	-4.50323400	-2.42717600	-2.96859300
Si	-0.36901300	2.79326000	-0.25332500
C	1.33127700	3.71142900	-0.20087100
H	1.89761200	3.23656100	0.60258500
C	-1.15053200	3.10912700	-1.99307700
H	-0.50192500	2.53881200	-2.66856300
C	-1.64247900	3.27556400	1.12263900
H	-2.45325500	2.55289700	0.95185700
C	-1.09683000	4.58584300	-2.44828600
H	-1.47728600	4.66238500	-3.47619500
H	-1.71810100	5.24366400	-1.83009300
H	-0.08427200	4.99566100	-2.45136600
C	-2.57733700	2.57575800	-2.20254800
H	-3.31044400	3.08047600	-1.55985100
H	-2.89192100	2.73908000	-3.24261600
H	-2.63543300	1.49935700	-2.01052400
C	1.26486700	5.21071400	0.15789100
H	2.27728400	5.63823400	0.17352300
H	0.67984000	5.79600900	-0.55902300
H	0.82936200	5.37572000	1.15057700
C	2.15964400	3.47039300	-1.48250400
H	2.23895100	2.40525000	-1.72424400
H	1.73471700	3.97084700	-2.35865700
H	3.17793400	3.86061700	-1.34827600
C	-1.17947600	3.07711300	2.57640300
H	-0.78860600	2.07005500	2.75748100
H	-0.39847900	3.79684400	2.85272300
H	-2.01480900	3.23775100	3.27258000
C	-2.26523300	4.67810200	0.96273900
H	-3.02226800	4.84749000	1.74165400
H	-1.51878100	5.47404100	1.06154300
H	-2.76012500	4.80587000	-0.00398200
Cl	0.29717000	0.09178200	-2.99613500
C	1.65919700	-1.05821100	0.24713400
O	1.14143900	-1.92070700	-0.45503800
N	2.91078000	-1.20062700	0.82381700
C	3.73661200	-2.38480100	0.63418700
H	4.04965900	-2.76087600	1.61602400
H	3.07342300	-3.13010500	0.18479100
C	4.94982700	-2.15204100	-0.25032900
C	4.79592800	-1.60711600	-1.53302700
C	6.22933100	-2.50718000	0.18843900
C	5.90653600	-1.41738600	-2.35431300
H	3.80508300	-1.33508400	-1.88861800
C	7.34258100	-2.32138400	-0.63548000
H	6.35777200	-2.93578700	1.18077900
C	7.18287600	-1.77354400	-1.90825300
H	5.77432000	-0.99364400	-3.34616900
H	8.33105100	-2.60050900	-0.27991600
H	8.04666200	-1.62436000	-2.55068700

Zero-point correction = 0.890045 (Hartree/Particle)
Thermal correction to Energy = 0.942543
Thermal correction to Enthalpy = 0.943487
Thermal correction to Gibbs Free Energy = 0.804727
Sum of electronic and zero-point Energies = -2792.381011
Sum of electronic and thermal Energies = -2792.328513

Sum of electronic and thermal Enthalpies = -2792.327569
Sum of electronic and thermal Free Energies = -2792.466329
E(RM06L) = -2794.98389636

TS_RE_VIa

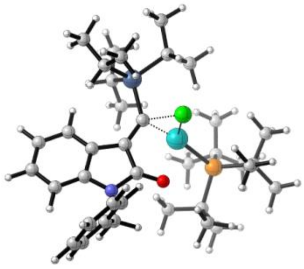

C	-2.73041200	1.94877200	2.07839400
C	-2.47206400	0.80960100	1.31334900
C	-3.33588200	-0.30364400	1.46801200
C	-4.43078500	-0.28796200	2.32376500
C	-4.66909400	0.87310400	3.06840400
C	-3.82501300	1.97814700	2.94993000
H	-2.09014300	2.81618800	2.01185000
H	-5.09188200	-1.14444100	2.40121900
H	-5.52048700	0.90911600	3.74234300
H	-4.01495300	2.87251600	3.53619100
N	-2.89304400	-1.35156900	0.66380800
C	-1.73833600	-1.00218400	-0.03040000
O	-1.15181400	-1.77886000	-0.77607300
C	-1.43844300	0.44290400	0.32528200
C	-3.48045100	-2.68141100	0.58420800
H	-3.68543400	-3.03762400	1.60089700
H	-2.69980800	-3.31611300	0.15403900
C	-4.74093800	-2.75222000	-0.26117100
C	-4.71408400	-2.34030900	-1.60116600
C	-5.93329300	-3.25276000	0.27089900
C	-5.86286600	-2.42166600	-2.38669900
H	-3.78709900	-1.96326400	-2.02567600
C	-7.08487600	-3.33881500	-0.51621100
H	-5.96266600	-3.58213900	1.30792900
C	-7.05213500	-2.92056100	-1.84638300
H	-5.82955100	-2.09931200	-3.42405700
H	-8.00469200	-3.72850400	-0.08790900
H	-7.94643500	-2.98329600	-2.46068000
C	-0.44910900	1.20894400	-0.26608100
Cl	-0.06311500	0.60377400	-2.36635700
Si	-0.15635100	3.13875000	-0.29980300
C	1.17365500	3.59287400	-1.61569900
H	0.81660200	3.08458000	-2.51821100
C	0.35844400	3.70148700	1.48885300
H	-0.58257600	3.91395300	2.01370900
C	-1.86736100	3.89716700	-0.77289400
H	-2.59767700	3.29150800	-0.21968400
C	1.25501900	5.09749700	-1.95566500
H	1.94496700	5.24774100	-2.79749900
H	0.28807600	5.51397000	-2.25465900
H	1.63302800	5.69784600	-1.12217600
C	2.58116600	3.04240300	-1.32535000
H	2.56661800	1.95604600	-1.16794600
H	3.25413200	3.24254500	-2.17111100
H	3.03228900	3.50270900	-0.43787500
C	-2.20603900	3.73507500	-2.26849900
H	-1.54455900	4.33377500	-2.90579900
H	-2.12572200	2.69387000	-2.59660700
H	-3.23385200	4.07057200	-2.46380300
C	-2.06731300	5.35783500	-0.31870300
H	-3.08786100	5.69168500	-0.55128400
H	-1.92368700	5.48281900	0.76093300
H	-1.38178100	6.04736100	-0.82493600
C	1.09450600	2.62560600	2.30972200
H	0.48847700	1.72354100	2.44325000
H	2.03324000	2.32348100	1.82903900
H	1.34888200	3.00807000	3.30838400
C	1.15417500	5.02410800	1.51832300
H	1.31552400	5.34171900	2.55777900
H	2.14193000	4.91775600	1.05759800
H	0.63267200	5.83910700	1.00569600
Pd	1.19273800	0.03071900	-0.19516500
P	3.03384400	-1.58947400	0.14963900
C	2.32811900	-3.12096800	1.12466400
C	3.71225600	-2.20837100	-1.56756700
C	4.51716700	-0.84818900	1.17333600
C	1.40071100	-2.58513100	2.24247000
C	1.41674200	-3.95361500	0.19417300
C	3.37796800	-4.06341100	1.74806900
C	2.50525700	-2.39862700	-2.51806700
C	4.53862700	-3.51019200	-1.53408600
C	4.57568600	-1.09753800	-2.20577700
C	4.78994000	0.58353100	0.65506000
C	4.09301500	-0.67695500	2.64933000
C	5.83408500	-1.65033800	1.13437600
H	0.93035200	-3.43953800	2.74785800
H	1.92163200	-2.00362100	3.00364900
H	0.60234700	-1.96371200	1.82403500
H	0.89675100	-4.70137300	0.80846200
H	0.65435900	-3.33957900	-0.29354300
H	1.97584700	-4.50317300	-0.56634200
H	3.96766600	-3.58249000	2.53266600
H	2.85913100	-4.91259900	2.21348900
H	4.06859600	-4.47231600	1.00620600
H	2.88859100	-2.65336400	-3.51565000
H	1.83276100	-3.19950900	-2.21271500
H	1.91219400	-1.48409300	-2.60728700
H	3.94557500	-4.37302600	-1.22130300
H	4.90369900	-3.72401800	-2.54794800
H	5.41231900	-3.44274100	-0.88102300
H	5.53786700	-0.96047200	-1.70641100
H	4.79019000	-1.38105200	-3.24431800
H	4.05037100	-0.13735800	-2.23250900
H	3.89474300	1.20731100	0.73036900
H	5.57226700	1.04031300	1.27641100
H	5.13390800	0.61620300	-0.37866000
H	4.86196900	-0.08729100	3.16567000
H	3.14705800	-0.13319600	2.74220000
H	4.00430200	-1.62489200	3.18390700
H	6.27342300	-1.68174000	0.13421700
H	6.56793200	-1.16326100	1.79128500
H	5.71438000	-2.67809600	1.48537100

Zero-point correction = 0.887838 (Hartree/Particle)
Thermal correction to Energy = 0.940229
Thermal correction to Enthalpy = 0.941173
Thermal correction to Gibbs Free Energy = 0.801260
Sum of electronic and zero-point Energies = -2792.366359
Sum of electronic and thermal Energies = -2792.313967
Sum of electronic and thermal Enthalpies = -2792.313023
Sum of electronic and thermal Free Energies = -2792.452936
E(RM06L) = -2794.96009181

TS_Isom_Va

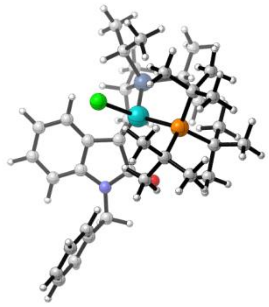

Pd	-0.74299400	-0.27439600	-0.67570000
C	1.94296200	1.93862800	-0.67935100
C	1.79200800	2.74470300	-1.80597100
C	3.23751500	1.63674600	-0.20409500
C	2.93198200	3.27117300	-2.42256100
H	0.80592100	2.94258400	-2.21339600
C	4.37830400	2.14677000	-0.81146700
C	4.20558100	2.97728400	-1.92703800
H	2.82553900	3.89963500	-3.30167600
H	5.36992400	1.89646700	-0.45015700

H	5.08256600	3.38555500	-2.42162300
P	-1.74758800	-2.40756000	0.11030000
C	0.97554800	1.24651200	0.18741600
C	-0.39432500	1.38346900	0.23324400
C	-3.49613300	-2.69822700	-0.70463700
C	-1.95666900	-2.48253400	2.05214500
C	-0.54120000	-3.83956100	-0.42888600
C	-3.44026900	-2.29936500	-2.19648700
H	-3.11924700	-1.26644800	-2.33746700
H	-2.77161900	-2.92395100	-2.78651700
H	-4.45130700	-2.40669600	-2.61246800
C	-4.53318100	-1.76086800	-0.04685300
H	-5.46153900	-1.81326400	-0.62995100
H	-4.78205200	-2.04246000	0.97830900
H	-4.20391200	-0.71694400	-0.05371000
C	-4.01348600	-4.15099200	-0.61656900
H	-4.09304500	-4.52356500	0.40576100
H	-5.01926500	-4.18569300	-1.05655800
H	-3.39292300	-4.84556900	-1.18723300
C	-0.66426200	-4.13993600	-1.93909100
H	0.15761200	-4.81601200	-2.21043800
H	-1.59456900	-4.65458000	-2.19286400
H	-0.56865600	-3.24037300	-2.55094400
C	-0.75001000	-5.17110000	0.32583900
H	-0.04868000	-5.90894700	-0.08574500
H	-0.54922600	-5.10264700	1.39601800
H	-1.75737400	-5.57389800	0.19087000
C	0.90094200	-3.32443000	-0.20216600
H	1.11132800	-2.47790900	-0.86360100
H	1.10191900	-3.01388700	0.82409100
H	1.60218200	-4.13189700	-0.45278700
C	-0.59223700	-2.65792600	2.75620800
H	0.09254000	-1.83281800	2.55149700
H	-0.78213900	-2.65137500	3.83839300
H	-0.09972800	-3.60485700	2.53230500
C	-2.49451500	-1.12059700	2.53916800
H	-3.48749500	-0.87936600	2.15731400
H	-2.56107000	-1.14491100	3.63482300
H	-1.80899300	-0.31788500	2.26211400
C	-2.90348400	-3.59787700	2.54847500
H	-2.90092700	-3.58402300	3.64630600
H	-3.93813000	-3.44890900	2.23236100
H	-2.58818800	-4.59615000	2.23684500
Si	-1.59469700	2.88091300	0.25470000
C	-2.92125500	2.48753200	1.58593100
H	-3.24079100	1.46588500	1.33530500
C	-0.55911300	4.46503300	0.65350500
H	-0.05936700	4.71417700	-0.29522700
C	-2.33859100	3.00042300	-1.52160500
H	-1.48594300	2.79007700	-2.18374400
C	-1.44476600	5.67232800	1.04000700
H	-1.89458900	5.53225400	2.02973800
H	-0.83475900	6.58431500	1.09270900
H	-2.25558200	5.86223200	0.33085500
C	-2.88149000	4.38900000	-1.91464400
H	-2.11634700	5.16978400	-1.84861100
H	-3.23880600	4.37046000	-2.95313900
H	-3.72851000	4.69543300	-1.28901400
C	-4.17745900	3.38224800	1.54159300
H	-4.91613000	3.03317700	2.27639700
H	-3.94771300	4.42427800	1.78904400
H	-4.66521600	3.37465500	0.56140900
C	0.54504200	4.29746900	1.71629900
H	0.13254600	4.01828900	2.69235500
H	1.28376600	3.54319600	1.43649100
H	1.08249600	5.24615400	1.85116800
C	-3.38872800	1.91008700	-1.80552800
H	-4.29244500	2.04184500	-1.19814900
H	-3.69339700	1.93702600	-2.85995700
H	-2.99055400	0.90787300	-1.61363900
C	-2.34509400	2.45384900	3.01634400
H	-1.44743800	1.83083400	3.09431200
H	-2.08066900	3.46023200	3.36174600
H	-3.08683000	2.05629900	3.72237600
C	1.81351300	0.49514100	1.20372600
O	1.45112100	-0.19613200	2.14540700
N	3.14058300	0.78665900	0.90793400
C	4.24905900	0.29690000	1.71008600
H	4.81617600	1.15245900	2.09950000
H	3.78205800	-0.20447100	2.56472700
C	5.18802500	-0.65205800	0.98186000
C	4.70313800	-1.60805700	0.08096100
C	6.56226900	-0.60482100	1.24418200
C	5.57700600	-2.49826400	-0.54375800
H	3.64014200	-1.64900700	-0.14001100
C	7.43757300	-1.49992000	0.62564600
H	6.95119400	0.13733500	1.93873700
C	6.94642400	-2.44896000	-0.27205100
H	5.18678500	-3.22990400	-1.24615600
H	8.50197200	-1.44909300	0.83949000
H	7.62579600	-3.14203800	-0.76083000
Cl	-0.43392700	-0.73332300	-3.04493600

Zero-point correction = 0.888688 (Hartree/Particle)
Thermal correction to Energy = 0.940837
Thermal correction to Enthalpy = 0.941781
Thermal correction to Gibbs Free Energy = 0.803903
Sum of electronic and zero-point Energies = -2792.374405
Sum of electronic and thermal Energies = -2792.322255
Sum of electronic and thermal Enthalpies = -2792.321311
Sum of electronic and thermal Free Energies = -2792.459190
E(RM06L) = -2794.97989235

Va

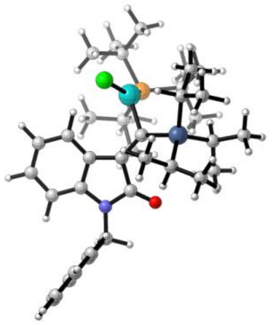

Pd	-1.51755800	0.07428700	-1.12256800
C	1.48987900	-1.31628700	-0.83195500
C	0.93293700	-1.98468600	-1.92390500
C	2.67119700	-1.83626200	-0.25482400
C	1.52110400	-3.17081100	-2.38195100
H	0.07175300	-1.57299900	-2.43551100
C	3.26724500	-3.01067400	-0.69874700
C	2.66755500	-3.68244300	-1.77085300
H	1.08579700	-3.68560500	-3.23377200
H	4.18288700	-3.37975300	-0.24936300
H	3.11691400	-4.59978500	-2.14155700
P	-2.88312700	-1.25913600	0.49640900
C	-3.87271900	-0.29688800	1.87272700
C	-4.16787400	-1.92872100	-0.82049300
C	-2.00296600	-2.78128500	1.33415700
C	1.16956200	-0.04339000	-0.14633700
C	0.11743200	0.81457500	-0.24332100
C	-0.72399000	-2.27118600	2.03834500
H	-0.92454900	-1.59494900	2.86911900
H	-0.18565400	-3.13747900	2.44484700
H	-0.05957700	-1.76483700	1.33737600
C	-1.53052600	-3.77700600	0.25255600
H	-0.90016400	-4.53259100	0.73868600
H	-2.35452100	-4.31016800	-0.22680800
H	-0.91947700	-3.29725200	-0.51588800
C	-2.86017000	-3.55508300	2.35784000
H	-2.27883700	-4.41661700	2.71238500
H	-3.10958800	-2.95743000	3.23730500
H	-3.78820300	-3.94325900	1.93171300
C	-2.95602800	-0.05481700	3.09294700
H	-3.45835900	0.65221900	3.76537000
H	-2.76475100	-0.96205900	3.66949500
H	-1.99748700	0.38951300	2.81166000
C	-4.24493700	1.10057400	1.33140800
H	-4.94853300	1.07309400	0.49986500

H	-4.71850400	1.67196200	2.14057900
H	-3.36299400	1.65255900	1.00597000
C	-5.16381100	-0.98758700	2.36227400
H	-5.92824200	-1.04546700	1.58373500
H	-4.99354600	-1.99350900	2.75060700
H	-5.58700500	-0.38923900	3.18021600
C	-5.00383800	-3.14318100	-0.36675100
H	-5.56908500	-2.95299200	0.54806700
H	-5.72972900	-3.38094500	-1.15599200
H	-4.39583300	-4.03762700	-0.21367000
C	-3.39873300	-2.31274200	-2.10977400
H	-4.12166600	-2.69793600	-2.84140700
H	-2.90867000	-1.44905600	-2.57781300
H	-2.64206700	-3.08146500	-1.95702400
C	-5.13808200	-0.79830800	-1.23338500
H	-5.86075900	-0.54882500	-0.45382400
H	-4.61172500	0.11531200	-1.52994300
H	-5.71015100	-1.13937800	-2.10571700
Si	0.06340200	2.75759900	0.09667200
C	1.87533000	3.36996800	-0.15941200
H	2.48081700	2.65785900	0.40413300
C	-1.05751400	3.59896300	-1.23818000
H	-0.75929300	3.11191600	-2.17419900
C	-0.68602200	3.05222600	1.85308700
H	-1.64149000	2.50929000	1.80749600
C	-0.79526200	5.11177900	-1.41833200
H	-1.43004200	5.49740200	-2.22823000
H	-1.03254600	5.69537800	-0.52173900
H	0.23960300	5.33137100	-1.69137300
C	-2.57100900	3.36786600	-1.08763600
H	-2.97259800	3.81317400	-0.16843800
H	-3.10904300	3.82188400	-1.93129300
H	-2.82033300	2.30110900	-1.08561600
C	2.18722200	4.75284500	0.45166500
H	3.24303600	5.00558100	0.27857500
H	1.58926000	5.56224800	0.02008200
H	2.02881000	4.76137200	1.53569000
C	2.32482800	3.29049600	-1.63366300
H	2.15242800	2.29837500	-2.06514000
H	1.80645500	4.01485900	-2.27148300
H	3.40002800	3.50573000	-1.71249400
C	0.10767000	2.46930700	3.03632000
H	0.30731000	1.40139800	2.92103800
H	1.08309800	2.95391500	3.14778400
H	-0.44852500	2.62085100	3.97302600
C	-1.03930400	4.52771300	2.14147400
H	-1.52296100	4.61152600	3.12499100
H	-0.14914100	5.16559400	2.16758400
H	-1.72820600	4.94890400	1.40356100
Cl	-0.84855700	0.76348900	-3.28665500
C	2.27571600	0.12063000	0.88460200
O	2.43134700	0.98678200	1.73784100
N	3.10538900	-0.98665800	0.76672800
C	4.23160600	-1.21885600	1.65743900
H	4.12847900	-2.21328700	2.10977700
H	4.12265900	-0.47603000	2.45451500
C	5.59464000	-1.09188500	0.99637400
C	5.89644400	0.00396000	0.17744400
C	6.58448000	-2.05178400	1.23381300
C	7.16192200	0.13255300	-0.39385500
H	5.13621500	0.75671600	-0.01315100
C	7.85476400	-1.92242800	0.66696100
H	6.36144600	-2.90705400	1.86890900
C	8.14567400	-0.82995300	-0.15040500
H	7.38125200	0.98628500	-1.02950000
H	8.61193700	-2.67787500	0.85997600
H	9.13103200	-0.72860600	-0.59720000

Zero-point correction = 0.889512 (Hartree/Particle)
Thermal correction to Energy = 0.942308
Thermal correction to Enthalpy = 0.943253
Thermal correction to Gibbs Free Energy = 0.803196
Sum of electronic and zero-point Energies = -2792.385993
Sum of electronic and thermal Energies = -2792.333197
Sum of electronic and thermal Enthalpies = -2792.332253
Sum of electronic and thermal Free Energies = -2792.472310
E(RM06L) = -2794.98885934

TS_RE_Va

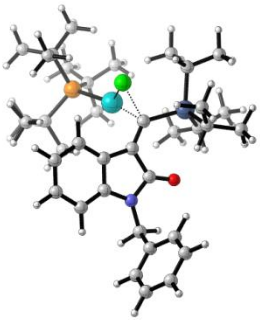

C	-0.82972200	-2.12305700	-1.92475600
C	-1.48241100	-1.46062600	-0.88009600
C	-2.54296400	-2.12622800	-0.21803000
C	-2.93809400	-3.41670200	-0.55005200
C	-2.25832200	-4.06336600	-1.58841000
C	-1.22187500	-3.42127600	-2.27035800
H	-0.03438900	-1.63114000	-2.46825800
H	-3.76505600	-3.89867300	-0.03987300
H	-2.55313800	-5.07008500	-1.87170000
H	-0.71444500	-3.92979100	-3.08523800
N	-3.08707900	-1.28689600	0.75959700
C	-2.46747200	-0.04685800	0.74746200
O	-2.79460900	0.88718500	1.47559200
C	-1.36570800	-0.11578000	-0.28243000
C	-4.16210600	-1.61805900	1.68585500
H	-4.10654000	-0.86075400	2.47395000
H	-3.94481600	-2.59313000	2.13765100
C	-5.54765600	-1.62691500	1.06220200
C	-6.35577700	-2.76615200	1.13095000
C	-6.04682500	-0.47696000	0.43462600
C	-7.63952000	-2.76455400	0.57886700
H	-5.98147100	-3.66156900	1.62365400
C	-7.32545700	-0.47445400	-0.12065800
H	-5.43049400	0.41723800	0.39219100
C	-8.12559400	-1.61891100	-0.05091000
H	-8.25496100	-3.65833400	0.63896700
H	-7.70101100	0.42311900	-0.60485500
H	-9.12224900	-1.61498900	-0.48401700
C	-0.51390900	0.94944800	-0.46771100
Cl	0.43189600	0.89492300	-2.55860700
Si	-0.78977800	2.85020400	-0.05996300
C	0.55490500	3.87458000	-0.98361100
H	0.51385500	3.49598400	-2.01154700
C	-0.74378700	3.10631800	1.85547100
H	-1.76447200	2.86291200	2.16705600
C	-2.54033700	3.24083300	-0.76788400
H	-3.14054100	2.37809800	-0.45489000
C	0.26206200	5.38953700	-1.05435200
H	1.01953400	5.88532400	-1.67744900
H	-0.71364400	5.60716200	-1.49989700
H	0.29289600	5.86935600	-0.07068400
C	1.98977900	3.64132000	-0.47960800
H	2.26092200	2.57768700	-0.50866500
H	2.71326700	4.18016700	-1.10809900
H	2.12847400	3.99435900	0.54964400
C	-2.57688700	3.30063000	-2.30705100
H	-2.01882100	4.16008900	-2.69776000
H	-2.15422100	2.39990900	-2.76455100
H	-3.61239900	3.40167900	-2.66114400
C	-3.21086600	4.48346600	-0.14743300
H	-4.22886600	4.60205700	-0.54450900
H	-3.29430100	4.40288700	0.94184200
H	-2.66995600	5.40956500	-0.37522200
C	0.18902000	2.14554400	2.61380700
H	-0.07673900	1.09874400	2.43537100
H	1.23973000	2.28190400	2.32846600
H	0.12375300	2.32289900	3.69696300
C	-0.45484800	4.56166800	2.27913700
H	-0.58177300	4.66730500	3.36581300
H	0.57322200	4.86251000	2.04637100
H	-1.12780600	5.28262700	1.80293800

Pd	1.33690000	0.18541500	-0.27968200
P	3.42863600	-1.01595500	0.27203500
C	2.97682500	-2.83770900	0.79209500
C	4.61236900	-1.08568800	-1.27314600
C	4.38706100	-0.18265200	1.74956600
C	1.71165300	-2.77418400	1.68242100
C	2.56459500	-3.65264700	-0.45409300
C	4.08111400	-3.61920500	1.53317100
C	3.74514600	-1.34416500	-2.52902300
C	5.73734700	-2.13924700	-1.21151900
C	5.25296700	0.30343300	-1.48949200
C	4.36214800	1.34905700	1.53520400
C	3.61843400	-0.42615300	3.06788200
C	5.84915100	-0.63321200	1.94565300
H	1.39522000	-3.79930400	1.91764500
H	1.86874800	-2.25615300	2.62872000
H	0.88557000	-2.28303000	1.15693900
H	2.14891100	-4.61073800	-0.11558600
H	1.78563700	-3.15026900	-1.03467000
H	3.40509100	-3.88352900	-1.11205000
H	4.32871900	-3.18309900	2.50385800
H	3.72602700	-4.64128000	1.72290800
H	5.00278300	-3.69814700	0.95078000
H	4.39281800	-1.29264100	-3.41469600
H	3.26954300	-2.32512900	-2.53262800
H	2.96317200	-0.58694100	-2.63999100
H	5.35292100	-3.16223300	-1.19940100
H	6.36212600	-2.04353800	-2.11002200
H	6.39161800	-2.01198800	-0.34554500
H	6.00613200	0.55036600	-0.73770000
H	5.75940300	0.30243500	-2.46338700
H	4.50200900	1.09966800	-1.51359500
H	3.33681300	1.71995100	1.45565400
H	4.82824700	1.83285200	2.40418300
H	4.90647200	1.67603500	0.64948300
H	4.06646300	0.20005000	3.85042600
H	2.56474900	-0.14039800	2.98584900
H	3.67501600	-1.46002700	3.41441300
H	6.48769900	-0.35355500	1.10408500
H	6.25761100	-0.13604900	2.83610800
H	5.94442600	-1.71037100	2.10192200

Zero-point correction = 0.888053 (Hartree/Particle)
Thermal correction to Energy = 0.940409
Thermal correction to Enthalpy = 0.941353
Thermal correction to Gibbs Free Energy = 0.801955
Sum of electronic and zero-point Energies = -2792.375845
Sum of electronic and thermal Energies = -2792.323488
Sum of electronic and thermal Enthalpies = -2792.322544
Sum of electronic and thermal Free Energies = -2792.461942
E(RM06L) = -2794.96939527

d) reaction of carbamoyl chloride **3b** (R = Mes), L = P*t*Bu$_3$

3b

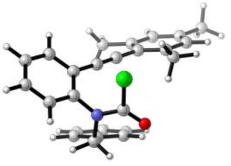

C	-0.44969500	-0.37672100	1.13933900
C	0.74787600	-0.46455200	0.93933300
C	-1.84264400	-0.26966300	1.41513200
C	-2.81289800	-0.27159100	0.38398200
C	-2.29222000	-0.16712700	2.74755700
C	-4.17001100	-0.16499900	0.69094600
C	-3.64746300	-0.06059700	3.04373300
H	-1.55414200	-0.17321100	3.54314500
C	-4.59297600	-0.05944400	2.01527100
H	-4.89243800	-0.17727300	-0.12017100
H	-3.96682800	0.01822600	4.07909500
H	-5.65253900	0.01569300	2.24130600
N	-2.41715100	-0.30423200	-0.99465800
C	-2.44428300	0.97481600	-1.75735100
H	-2.48740500	0.69997000	-2.81265000
H	-3.38142400	1.47276100	-1.49186600
C	-1.27183200	1.89494800	-1.48985800
C	-0.03513200	1.66911800	-2.11138300
C	-1.40951600	2.98685500	-0.62553500
C	1.04374300	2.51637600	-1.86315100
H	0.07472400	0.82361900	-2.78452100
C	-0.33039600	3.83801600	-0.37716100
H	-2.36722300	3.17414100	-0.14499800
C	0.89891600	3.60286700	-0.99517000
H	1.99790300	2.33123100	-2.34875100
H	-0.45215700	4.68477600	0.29322000
H	1.73911200	4.26646400	-0.80822600
C	-1.92289600	-1.39497100	-1.63711000
O	-1.42032300	-1.42029400	-2.73189500
Cl	-2.15432000	-2.94445900	-0.71746200
C	2.15482400	-0.53699700	0.71567000
C	2.68896600	-1.51234100	-0.16243500
C	3.01274100	0.37835500	1.37633800
C	4.07121600	-1.55170800	-0.35510800
C	4.38606700	0.29643200	1.14711300
C	4.93789700	-0.66250400	0.28932900
H	4.48339800	-2.30016500	-1.02882000
H	5.04447700	1.00138500	1.65118900
C	1.78678500	-2.48830500	-0.87593000
H	1.16049800	-3.04542700	-0.16997700
H	1.10122300	-1.97985900	-1.56404000
H	2.37162700	-3.20868500	-1.45596900
C	2.44749100	1.43278200	2.29562100
H	1.77491600	2.10923700	1.75418600
H	1.85871300	0.98474900	3.10523000
H	3.24679600	2.03009300	2.74517900
C	6.43228300	-0.75053500	0.08678200
H	6.90092200	0.23944500	0.11581500
H	6.90455700	-1.35460800	0.87349000
H	6.68031000	-1.21584400	-0.87297400

Zero-point correction = 0.402294 (Hartree/Particle)
Thermal correction to Energy = 0.428669
Thermal correction to Enthalpy = 0.429613
Thermal correction to Gibbs Free Energy = 0.342337
Sum of electronic and zero-point Energies = -1555.640482
Sum of electronic and thermal Energies = -1555.614107
Sum of electronic and thermal Enthalpies = -1555.613163
Sum of electronic and thermal Free Energies = -1555.700439
E(RM06L) = -1556.33261997

PC_3b

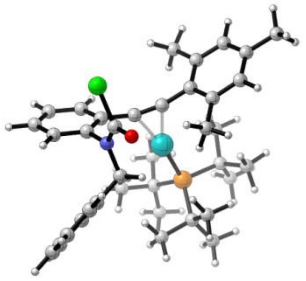

C	0.74220400	1.03592400	1.11758400
C	0.03636000	1.86968100	0.51433300
C	1.82203000	0.44147200	1.85974200
C	3.08191100	0.20398100	1.26479200
C	1.64661300	0.08234200	3.21043600
C	4.11446900	-0.38204900	1.99831900
C	2.68733800	-0.48273000	3.94132000
H	0.68055600	0.25929800	3.67225800
C	3.92525900	-0.71938000	3.33771000

H	5.06628300	-0.55993700	1.50852800
H	2.53023200	-0.74377700	4.98424600
H	4.73700700	-1.16738900	3.90313400
N	3.27744500	0.50060300	-0.12477800
C	2.82129500	-0.51473500	-1.11162200
H	1.85275500	-0.88393500	-0.76076700
H	2.66248600	0.01631200	-2.05142200
C	3.80462700	-1.65201500	-1.29481100
C	3.55467500	-2.91041900	-0.73524600
C	4.98465700	-1.45694600	-2.02902400
C	4.46331100	-3.95820700	-0.90382200
H	2.64263700	-3.07082700	-0.16460900
C	5.89426900	-2.50082800	-2.19458300
H	5.18225100	-0.48297600	-2.46891400
C	5.63548700	-3.75467200	-1.63284600
H	4.25472900	-4.93060000	-0.46534100
H	6.80335500	-2.33829000	-2.76765300
H	6.34343700	-4.56845200	-1.76622100
C	3.84860700	1.63025500	-0.61219300
O	4.04636800	1.89754200	-1.77130100
Cl	4.32843200	2.81945100	0.67629100
Pd	-0.98271900	-0.10271800	0.43223600
P	-2.69477600	-1.66012200	-0.08779600
C	-2.89463800	-2.92180700	1.38440600
C	-4.38945600	-0.73035300	-0.34143800
C	-2.30193700	-2.66906800	-1.70990000
C	-3.70067300	-4.20047300	1.08047800
C	-1.47661000	-3.31711100	1.86502100
C	-3.55094700	-2.20301400	2.58417800
C	-5.65601900	-1.60731700	-0.27129700
C	-4.48857400	0.38777600	0.72479700
C	-4.37304500	-0.00060000	-1.70315800
C	-1.71804900	-1.68332300	-2.75092700
C	-1.16946800	-3.67846200	-1.41810500
C	-3.48292000	-3.43145800	-2.34410300
H	-3.78494100	-4.79731200	1.99918900
H	-4.71601600	-3.98664900	0.73701600
H	-3.21610400	-4.83370900	0.33308400
H	-0.88519000	-2.42813400	2.11057700
H	-1.56948800	-3.92960400	2.77242100
H	-0.91798400	-3.90184100	1.13377900
H	-4.61099900	-1.99005500	2.42882100
H	-3.48015000	-2.85762600	3.46296800
H	-3.03499800	-1.26761500	2.82414800
H	-5.80987200	-2.04197000	0.71969600
H	-5.64889400	-2.42056900	-1.00137800
H	-6.53419400	-0.98296100	-0.48610400
H	-5.39147800	0.98151400	0.52644100
H	-4.56413300	0.00976600	1.74459600
H	-3.62128400	1.05381000	0.67444900
H	-5.24278300	0.66792600	-1.74858500
H	-3.47674000	0.61694700	-1.82083000
H	-4.44774600	-0.68042700	-2.55484900
H	-2.44337400	-0.95437100	-3.11280600
H	-1.36599200	-2.25536900	-3.62019900
H	-0.86905600	-1.13364400	-2.33180900
H	-0.31101400	-3.19481000	-0.94008800
H	-1.49094800	-4.51698200	-0.79623800
H	-0.82250900	-4.09892600	-2.37107900
H	-4.26806800	-2.76185800	-2.70428600
H	-3.93858500	-4.15164300	-1.65950900
H	-3.11892000	-3.99404500	-3.21490900
C	-0.58744500	3.05052800	-0.01549400
C	-1.33150100	3.88924800	0.85382500
C	-0.46074000	3.37835300	-1.38828100
C	-1.93218500	5.03393500	0.33010900
C	-1.08259000	4.53590300	-1.85944600
C	-1.82452500	5.37641200	-1.02279800
H	-2.49830900	5.67977400	0.99855000
H	-0.98167600	4.78981600	-2.91278300
C	-1.46679300	3.55653000	2.31908700
H	-1.88571300	2.55246200	2.46131500
H	-0.49239400	3.56494400	2.82294600
H	-2.11604600	4.27751300	2.82539700
C	0.33522300	2.50594500	-2.32622100
H	1.41231500	2.57357400	-2.12862200
H	0.05273700	1.45189700	-2.21693800
H	0.17223100	2.80287800	-3.36710800
C	-2.51194100	6.60578700	-1.56869000
H	-1.98253000	7.00779300	-2.43914900
H	-3.53837700	6.37928300	-1.88860000
H	-2.57588900	7.39778100	-0.81455900

Zero-point correction= 0.774948 (Hartree/Particle)
Thermal correction to Energy= 0.823431
Thermal correction to Enthalpy= 0.824375
Thermal correction to Gibbs Free Energy= 0.686309
Sum of electronic and zero-point Energies= -2496.941858
Sum of electronic and thermal Energies= -2496.893375
Sum of electronic and thermal Enthalpies= -2496.892431
Sum of electronic and thermal Free Energies= -2497.030497
E(RM06L) = -2499.40166984

TS_OA_3b

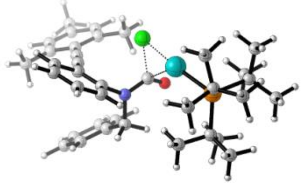

C	0.96900500	-1.61070500	0.44101700
C	2.11525600	-1.15922000	0.09090300
C	0.18021400	-2.38748000	1.35835000
C	-1.15235300	-2.58480500	0.94672100
C	0.61430600	-2.87930200	2.59612100
C	-2.04804000	-3.27634600	1.76464000
C	-0.27344400	-3.59005800	3.40120900
H	1.64070500	-2.71236100	2.90789200
C	-1.59512000	-3.78364500	2.98459700
H	-3.07930700	-3.41096700	1.46251700
H	0.06175700	-3.98950500	4.35379300
H	-2.28748500	-4.32828700	3.62042400
N	-1.45004700	-2.05773900	-0.32386100
C	-2.29601200	-2.84359000	-1.26144900
H	-2.00542300	-2.49983900	-2.25695900
H	-1.99820800	-3.89447500	-1.16790800
C	-3.79761900	-2.72719500	-1.08316100
C	-4.46982900	-1.53058000	-1.36636600
C	-4.55206900	-3.84439600	-0.70188300
C	-5.85650400	-1.44606200	-1.24498800
H	-3.90375400	-0.66529400	-1.69598000
C	-5.94176800	-3.76549900	-0.58365700
H	-4.04768400	-4.78694100	-0.49967400
C	-6.59729700	-2.56314700	-0.84997000
H	-6.36091600	-0.50989400	-1.46904500
H	-6.50906500	-4.64364800	-0.28707000
H	-7.67807800	-2.49775600	-0.75896100
C	-0.50024500	-1.20106100	-0.87668400
O	-0.39431000	-1.01208900	-2.07450600
Pd	0.77888200	0.53697700	-0.17796700
Cl	2.71388700	1.88007000	-0.83762600
P	-0.78902700	2.49466700	0.15847200
C	0.12816300	4.10550000	0.79675000
C	-2.16785600	2.08635100	1.48102100
C	-1.64436300	2.92171100	-1.53575400
C	1.18087800	3.67978000	1.84861400
C	-0.79633100	5.17633800	1.41863200

C	0.91139600	4.79414700	-0.34548400
C	-2.64834300	0.63636900	1.27319400
C	-1.55332500	2.09950100	2.89972500
C	-3.40475800	3.00979300	1.45963800
C	-0.55487100	2.85662600	-2.63351400
C	-2.67115800	1.82637600	-1.89239100
C	-2.36332200	4.28520300	-1.60559300
H	1.70957400	4.58044700	2.18966200
H	0.74916700	3.20443900	2.73029700
H	1.91731000	3.00560100	1.40748700
H	-1.27634800	4.85380000	2.34479900
H	-0.17829200	6.04854900	1.67003900
H	-1.57255900	5.52095300	0.73059300
H	0.25967200	5.27044700	-1.08155700
H	1.51747500	5.59209100	0.10431800
H	1.59308800	4.10668500	-0.84701100
H	-3.43444300	0.41952700	2.00914500
H	-3.06639200	0.44402500	0.28634600
H	-1.83492400	-0.07021800	1.44270400
H	-2.28638400	1.67046400	3.59558300
H	-0.65082200	1.48171600	2.95358900
H	-1.31180200	3.09913200	3.26340200
H	-3.98207600	2.90160600	0.53769800
H	-4.06854000	2.72594500	2.28783300
H	-3.15916000	4.06496400	1.58580000
H	-0.06819400	1.87771300	-2.65454100
H	-1.03545200	3.02087700	-3.60778700
H	0.22415700	3.60903400	-2.51664900
H	-3.02868200	2.01058300	-2.91425400
H	-2.21748800	0.83232700	-1.88395800
H	-3.54758800	1.83079700	-1.23923400
H	-1.68249200	5.12913100	-1.48283300
H	-2.82250500	4.38615100	-2.59847400
H	-3.16290500	4.38112200	-0.86673000
C	3.52136900	-1.34471200	-0.14557500
C	4.47763600	-0.82256200	0.75914900
C	3.93239200	-2.06505600	-1.29459300
C	5.82853100	-1.06086900	0.50678800
C	5.29706900	-2.25881200	-1.50547500
C	6.26228100	-1.77229700	-0.61687800
H	6.56537500	-0.66721900	1.20392200
H	5.61605100	-2.80216200	-2.39254600
C	4.05345700	-0.03138300	1.96998700
H	3.55920300	0.89512400	1.65655500
H	3.34429200	-0.58759000	2.59623400
H	4.91787800	0.22781600	2.58914900
C	2.91990700	-2.60074800	-2.27764000
H	2.30547800	-3.39203800	-1.82823900
H	2.23339500	-1.81670300	-2.61571200
H	3.41757400	-3.02408800	-3.15542500
C	7.73232500	-2.02535600	-0.85296600
H	8.06834000	-2.93384900	-0.33415400
H	7.94930500	-2.16010400	-1.91785600
H	8.34541000	-1.19625300	-0.48332600

Zero-point correction = 0.774190 (Hartree/Particle)
Thermal correction to Energy = 0.822135
Thermal correction to Enthalpy = 0.823079
Thermal correction to Gibbs Free Energy = 0.688572
Sum of electronic and zero-point Energies = -2496.916228
Sum of electronic and thermal Energies = -2496.868283
Sum of electronic and thermal Enthalpies = -2496.867339
Sum of electronic and thermal Free Energies = -2497.001846
E(RM06L) = -2499.38372264

VIIb

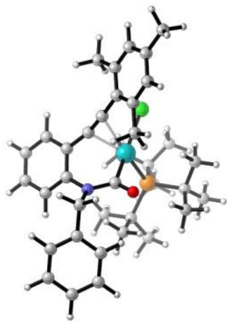

C	-1.45056300	1.31965600	1.24308900
C	-2.49987100	1.31309400	0.59683300
C	-0.36065000	1.64875500	2.11475100
C	0.88894600	1.99113200	1.54410900
C	-0.50636400	1.64657300	3.51286200
C	1.95204300	2.34811000	2.37755600
C	0.55962100	2.01141800	4.33093400
H	-1.46377500	1.36595500	3.93958100
C	1.78569100	2.36861900	3.76386600
H	2.90947300	2.60070600	1.93476900
H	0.43299500	2.01701200	5.40965400
H	2.62041900	2.64979200	4.39968000
N	1.02956600	1.99409900	0.13149900
C	1.40068200	3.26975700	-0.54370800
H	0.81413100	3.29585200	-1.46549500
H	1.05992900	4.08033200	0.10678300
C	2.86976800	3.45514400	-0.87328900
C	3.44174200	2.79279200	-1.97004000
C	3.66880900	4.32677500	-0.12192700
C	4.78577600	2.98365500	-2.29082000
H	2.82015600	2.13280700	-2.56756200
C	5.01474500	4.52036400	-0.44239800
H	3.23194000	4.86645600	0.71574700
C	5.57738400	3.84543600	-1.52643400
H	5.21455900	2.46576400	-3.14489900
H	5.61890300	5.20278100	0.14977500
H	6.62325400	3.99626400	-1.78046500
C	0.55708000	0.94823400	-0.65411400
O	0.59109300	1.02658600	-1.87698700
Pd	-0.50732600	-0.55488800	0.17529400
Cl	-2.43699200	-1.98042500	0.79836500
P	1.16937700	-2.33194100	-0.19953800
C	0.84230700	-3.81237800	1.05482600
C	3.04452400	-1.81278800	0.03424300
C	0.92064800	-2.97602700	-2.01855100
C	0.51571800	-3.19826200	2.43743100
C	2.02486100	-4.79622400	1.20596100
C	-0.38166600	-4.66141300	0.64092100
C	3.34069500	-0.46131500	-0.64998300
C	3.33439100	-1.57763600	1.53447900
C	4.05935000	-2.83292100	-0.53341700
C	-0.59943600	-3.01395100	-2.29958600
C	1.52853600	-1.97769700	-3.02914900
C	1.52531800	-4.36831300	-2.30202400
H	0.35681100	-4.02053700	3.14818100
H	1.31756400	-2.57369400	2.83487300
H	-0.40334800	-2.60990500	2.39516100
H	2.91814500	-4.34916700	1.64458200
H	1.70361300	-5.59551000	1.88642300
H	2.30114900	-5.27235400	0.26133000
H	-0.19881500	-5.26357400	-0.25175900
H	-0.58318600	-5.36303100	1.46155000
H	-1.27717700	-4.05679500	0.50296500
H	4.42403100	-0.28904400	-0.60371800
H	3.04490700	-0.41685200	-1.69594100
H	2.86385700	0.36465200	-0.12545500
H	4.32984200	-1.12253200	1.62119500
H	2.61789500	-0.87978600	1.97971500
H	3.34620500	-2.49139400	2.12973600
H	4.04264300	-2.85404300	-1.62622500
H	5.06701400	-2.51230800	-0.23682000
H	3.92267100	-3.85026900	-0.16953700
H	-1.04582500	-2.01970800	-2.20651400

H	-0.74901700	-3.35996100	-3.33135400
H	-1.14983400	-3.68123200	-1.63729500
H	1.20908700	-2.28293600	-4.03454100
H	1.17806900	-0.95597200	-2.86444400
H	2.62191000	-1.98935200	-3.02727100
H	1.06254600	-5.16365300	-1.71583300
H	1.35097700	-4.60628600	-3.35991900
H	2.60428800	-4.40398900	-2.13698300
C	-3.75822600	1.31816100	-0.05955300
C	-4.91502100	0.92383800	0.66719400
C	-3.86351700	1.74629600	-1.40887100
C	-6.14669400	0.95383100	0.01636400
C	-5.12143200	1.74861800	-2.00947000
C	-6.27269300	1.34984800	-1.32060700
H	-7.03545500	0.65388600	0.56715400
H	-5.20680200	2.07345900	-3.04413500
C	-4.82136700	0.49519800	2.10941100
H	-4.14453000	-0.35989800	2.20936600
H	-4.43216700	1.30410000	2.74119300
H	-5.80602600	0.21239300	2.49372300
C	-2.64985700	2.19718000	-2.17993100
H	-2.14600000	3.03147200	-1.67610200
H	-1.90608000	1.39909300	-2.27971900
H	-2.92929700	2.52912500	-3.18446700
C	-7.61646900	1.32707000	-2.00792300
H	-7.65890800	2.04642700	-2.83250900
H	-7.82338900	0.33414800	-2.42967400
H	-8.42868200	1.55731500	-1.30995300

Zero-point correction = 0.776905 (Hartree/Particle)
Thermal correction to Energy = 0.824504
Thermal correction to Enthalpy = 0.825449
Thermal correction to Gibbs Free Energy = 0.695788
Sum of electronic and zero-point Energies = -2496.951152
Sum of electronic and thermal Energies = -2496.903553
Sum of electronic and thermal Enthalpies = -2496.902608
Sum of electronic and thermal Free Energies = -2497.032268
E(RM06L) = -2499.42768310

TS_AI_VIIb

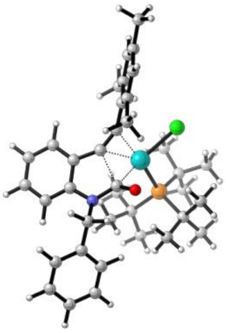

C	0.96900500	-1.61070500	0.44101700
C	2.11525600	-1.15922000	0.09090300
C	0.18021400	-2.38748000	1.35835000
C	-1.15235300	-2.58480500	0.94672100
C	0.61430600	-2.87930200	2.59612100
C	-2.04804000	-3.27634600	1.76464000
C	-0.27344400	-3.59005800	3.40120900
H	1.64070500	-2.71236100	2.90789200
C	-1.59512000	-3.78364500	2.98459700
H	-3.07930700	-3.41096700	1.46251700
H	0.06175700	-3.98950500	4.35379300
H	-2.28748500	-4.32828700	3.62042400
N	-1.45004700	-2.05773900	-0.32386100
C	-2.29601200	-2.84359000	-1.26144900
H	-2.00542300	-2.49983900	-2.25695900
H	-1.99820800	-3.89447500	-1.16790800
C	-3.79761900	-2.72719500	-1.08316100
C	-4.46982900	-1.53058000	-1.36636600
C	-4.55206900	-3.84439600	-0.70188300
C	-5.85650400	-1.44606200	-1.24498800
H	-3.90375400	-0.66529400	-1.69598000
C	-5.94176800	-3.76549900	-0.58365700
H	-4.04768400	-4.78694100	-0.49967400
C	-6.59729700	-2.56314700	-0.84997000
H	-6.36091600	-0.50989400	-1.46904500
H	-6.50906500	-4.64364800	-0.28707000
H	-7.67807800	-2.49775600	-0.75896100
C	-0.50024500	-1.20106100	-0.87668400
O	-0.39431000	-1.01208900	-2.07450600
Pd	0.77888200	0.53697700	-0.17796700
Cl	2.71388700	1.88007000	-0.83762600
P	-0.78902700	2.49466700	0.15847200
C	0.12816300	4.10550000	0.79675000
C	-2.16785600	2.08635100	1.48102100
C	-1.64436300	2.92171100	-1.53575400
C	1.18087800	3.67978000	1.84861400
C	-0.79633100	5.17633800	1.41863200
C	0.91139600	4.79414700	-0.34548400
C	-2.64834300	0.63636900	1.27319400
C	-1.55332500	2.09950100	2.89972500
C	-3.40475800	3.00979300	1.45963800
C	-0.55487100	2.85662600	-2.63351400
C	-2.67115800	1.82637600	-1.89239100
C	-2.36332200	4.28520300	-1.60559300
H	1.70957400	4.58044700	2.18966200
H	0.74916700	3.20443900	2.73029700
H	1.91731000	3.00560100	1.40748700
H	-1.27634800	4.85380000	2.34479900
H	-0.17829200	6.04854900	1.67003900
H	-1.57255900	5.52095300	0.73059300
H	0.25967200	5.27044700	-1.08155700
H	1.51747500	5.59209100	0.10431800
H	1.59308800	4.10668500	-0.84701100
H	-3.43444300	0.41952700	2.00914500
H	-3.06639200	0.44402500	0.28634600
H	-1.83492400	-0.07021800	1.44270400
H	-2.28638400	1.67046400	3.59558300
H	-0.65082200	1.48171600	2.95358900
H	-1.31180200	3.09913200	3.26340200
H	-3.98207600	2.90160600	0.53769800
H	-4.06854000	2.72594500	2.28783300
H	-3.15916000	4.06496400	1.58580000
H	-0.06819400	1.87771300	-2.65454100
H	-1.03545200	3.02087700	-3.60778700
H	0.22415700	3.60903400	-2.51664900
H	-3.02868200	2.01058300	-2.91425400
H	-2.21748800	0.83232700	-1.88395800
H	-3.54758800	1.83079700	-1.23923400
H	-1.68249200	5.12913100	-1.48283300
H	-2.82250500	4.38615100	-2.59847400
H	-3.16290500	4.38112200	-0.86673000
C	3.52136900	-1.34471200	-0.14557500
C	4.47763600	-0.82256200	0.75914900
C	3.93239200	-2.06505600	-1.29459300
C	5.82853100	-1.06086900	0.50678800
C	5.29706900	-2.25881200	-1.50547500
C	6.26228100	-1.77229700	-0.61687800
H	6.56537500	-0.66721900	1.20392200
H	5.61605100	-2.80216200	-2.39254600
C	4.05345700	-0.03138300	1.96998700
H	3.55920300	0.89512400	1.65655500
H	3.34429200	-0.58759000	2.59623400
H	4.91787800	0.22781600	2.58914900
C	2.91990700	-2.60074800	-2.27764000
H	2.30547800	-3.39203800	-1.82823900
H	2.23339500	-1.81670300	-2.61571200
H	3.41757400	-3.02408800	-3.15542500
C	7.73232500	-2.02535600	-0.85296600
H	8.06834000	-2.93384900	-0.33415400
H	7.94930500	-2.16010400	-1.91785600
H	8.34541000	-1.19625300	-0.48332600

Zero-point correction = 0.775477 (Hartree/Particle)
Thermal correction to Energy = 0.822610
Thermal correction to Enthalpy = 0.823555
Thermal correction to Gibbs Free Energy = 0.695007
Sum of electronic and zero-point Energies = -2496.917914
Sum of electronic and thermal Energies = -2496.870781
Sum of electronic and thermal Enthalpies = -2496.869837
Sum of electronic and thermal Free Energies = -2496.998384

E(RM06L) = -2499.39642049

VIb

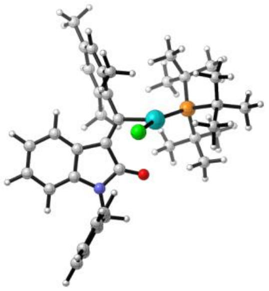

Pd	-0.72331800	-0.76016100	-0.87202800
C	2.27716300	1.90265500	0.58915700
C	2.31333400	3.28217300	0.38606500
C	3.45625400	1.23935000	1.00325900
C	3.50542300	3.97975400	0.61770600
H	1.42980300	3.81343000	0.04930900
C	4.64772000	1.91862600	1.22683400
C	4.65597500	3.30580300	1.03336600
H	3.53377900	5.05478100	0.46437400
H	5.54904400	1.39058100	1.51967300
H	5.57585900	3.85917600	1.20159600
P	-2.69831300	-1.37553300	0.34336900
C	-4.19982500	-0.15574700	0.51186900
C	-3.19493000	-2.80660400	-0.89183000
C	-2.29481400	-2.15526500	2.07612200
C	1.24614700	0.86694300	0.38829800
C	0.00205000	0.97068000	-0.14033400
C	-1.48881300	-1.12117000	2.89522000
H	-2.04505100	-0.20923900	3.11308300
H	-1.22288900	-1.57799400	3.85773700
H	-0.55934000	-0.86375000	2.38220800
C	-1.35075100	-3.36630100	1.89825900
H	-1.02558200	-3.68624900	2.89679900
H	-1.83706800	-4.22649400	1.43306300
H	-0.45228400	-3.09868300	1.33592100
C	-3.52801200	-2.59980200	2.88995200
H	-3.17854100	-3.07690300	3.81509100
H	-4.16450400	-1.76188600	3.18519500
H	-4.14656500	-3.32973500	2.36220700
C	-3.99511400	0.77211900	1.72882700
H	-4.78264000	1.53642300	1.71037000
H	-4.07639400	0.24968300	2.68423300
H	-3.03787800	1.29526400	1.68845000
C	-4.22679500	0.76194200	-0.73230300
H	-4.43237600	0.22704000	-1.65927500
H	-5.02722000	1.50163500	-0.59787900
H	-3.28799500	1.30633100	-0.85154800
C	-5.57203400	-0.84684600	0.66044500
H	-5.85565400	-1.41795400	-0.22621000
H	-5.62151200	-1.51162100	1.52600800
H	-6.33717100	-0.07145800	0.79978000
C	-4.12574700	-3.88902600	-0.30935700
H	-5.07158100	-3.48231300	0.05687300
H	-4.36515900	-4.60752400	-1.10495600
H	-3.65917200	-4.45365600	0.50079400
C	-1.88702500	-3.48007900	-1.38354100
H	-2.15195400	-4.28132900	-2.08681200
H	-1.24075500	-2.79348600	-1.95274800
H	-1.28777600	-3.92228900	-0.58964700
C	-3.85991800	-2.21930000	-2.15766700
H	-4.86683000	-1.83687300	-1.97829700
H	-3.25547800	-1.42613200	-2.60890600
H	-3.94883100	-3.02332500	-2.89952800
Cl	0.99792400	-0.98024600	-2.53003200
C	1.88991300	-0.42640300	0.79700600
O	1.38710100	-1.54366800	0.90000500
N	3.20318200	-0.13260100	1.13118100
C	4.15861200	-1.14722700	1.54331000
H	4.63319400	-0.83346100	2.48152200
H	3.55712100	-2.03720500	1.75602200
C	5.22026600	-1.46015100	0.50038700
C	4.87054200	-1.64989300	-0.84382300
C	6.55838800	-1.60393500	0.88303700
C	5.84894300	-1.97609300	-1.78307800
H	3.83597100	-1.53896300	-1.16007800
C	7.53703800	-1.93592300	-0.05694200
H	6.83835600	-1.45701800	1.92475200
C	7.18344800	-2.12141000	-1.39381500
H	5.56504800	-2.11741700	-2.82242500
H	8.57283300	-2.04221300	0.25537400
H	7.94254000	-2.37486400	-2.12929000
C	-0.74662000	2.22556600	-0.31416700
C	-1.09007300	2.99081300	0.83937700
C	-1.12433700	2.71323500	-1.59927100
C	-1.81140500	4.17905200	0.68357600
C	-1.82784400	3.91565900	-1.69251500
C	-2.19486100	4.66228200	-0.56844300
H	-2.06860000	4.75018400	1.57354200
H	-2.09387700	4.28266600	-2.68175600
C	-0.76961500	1.98802900	-2.87182700
H	0.29561400	1.75081400	-2.93086700
H	-1.28730900	1.02350300	-2.94059100
H	-1.04536100	2.58925200	-3.74422600
C	-0.66150400	2.61069400	2.23970300
H	-1.30622500	3.09478600	2.98075000
H	-0.68319800	1.53350500	2.41098200
H	0.36694200	2.93999800	2.43294500
C	-2.98811200	5.93929700	-0.70836900
H	-2.63628300	6.53784000	-1.55627900
H	-4.05193300	5.72902200	-0.88349600
H	-2.92001600	6.55526500	0.19419600

Zero-point correction = 0.778262 (Hartree/Particle)
Thermal correction to Energy = 0.825081
Thermal correction to Enthalpy = 0.826025
Thermal correction to Gibbs Free Energy = 0.698294
Sum of electronic and zero-point Energies = -2496.986152
Sum of electronic and thermal Energies = -2496.939334
Sum of electronic and thermal Enthalpies = -2496.938389
Sum of electronic and thermal Free Energies = -2497.066120
E(RM06L) = -2499.46624816

TS_RE_VIb

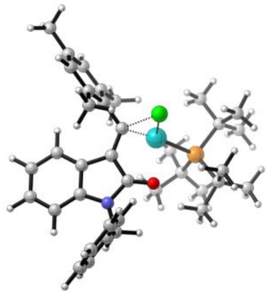

C	2.37196800	0.84133000	1.17628900
C	2.66854500	2.01318100	1.87666600
C	3.11788000	-0.32955400	1.45504400
C	3.70144600	2.00674200	2.82139800
H	2.10713900	2.92245700	1.69511400
C	4.14784500	-0.34563700	2.38824200
C	4.43318200	0.84331500	3.07130200
H	3.93479000	2.91924300	3.36257000
H	4.72540300	-1.24532100	2.57237600
H	5.23666300	0.85562500	3.80272700
C	1.36896300	0.49175500	0.15774300
C	0.47691600	1.35078400	-0.45981400
C	0.60317300	2.82855600	-0.45109400
C	-0.30827200	3.64199700	0.26407300
C	1.68666800	3.43828500	-1.14223500
C	-0.13097000	5.02877900	0.27057800
C	1.80645400	4.82956100	-1.11849500
C	0.90943700	5.64729800	-0.42552600
H	-0.83122100	5.64184500	0.83421900
H	2.63393500	5.28807000	-1.65600000
C	-1.44972000	3.06161700	1.06547800
H	-2.16336100	2.52649500	0.42399300
H	-1.09428100	2.35083100	1.82250000

H	-2.00274300	3.85149000	1.58535500
C	2.73895900	2.63724600	-1.87391800
H	3.40143400	2.12351800	-1.16664300
H	2.29723600	1.87694300	-2.52242900
H	3.35910300	3.29703700	-2.48840700
C	1.05092700	7.15047400	-0.44680500
H	0.57577000	7.61109900	0.42571900
H	2.10341300	7.45429300	-0.46230300
H	0.57769200	7.57861400	-1.34076200
C	1.56771000	-0.97145000	-0.12713900
O	0.99854200	-1.72292500	-0.91432300
N	2.63329800	-1.38145600	0.67573600
C	3.11807500	-2.75416200	0.68594100
H	3.24349700	-3.07455700	1.72710700
H	2.31339300	-3.34836600	0.24233500
C	4.40939300	-2.96317600	-0.08711500
C	4.46396800	-2.66612500	-1.45655100
C	5.54893200	-3.47405300	0.54184400
C	5.64083500	-2.86931100	-2.17561000
H	3.57625800	-2.28387700	-1.95397400
C	6.72861700	-3.68181600	-0.17812400
H	5.51451900	-3.71616100	1.60245900
C	6.77727400	-3.37692600	-1.53827900
H	5.67075300	-2.63611400	-3.23674600
H	7.60670500	-4.07804500	0.32523000
H	7.69359700	-3.53505300	-2.10084600
Cl	-0.17586700	0.84506200	-2.45623400
Pd	-1.27334900	0.33024300	-0.23674800
P	-3.22729300	-1.10753700	0.12433200
C	-3.59784700	-1.26717300	2.02937700
C	-2.24042300	-1.40881900	2.76080000
C	-4.21278800	0.05045800	2.55144300
C	-4.51888200	-2.43129900	2.44742500
H	-1.57562600	-0.56963800	2.52668300
H	-2.41983900	-1.40516600	3.84456500
H	-1.71034400	-2.32991000	2.51902700
H	-4.24393400	0.00981500	3.64825100
H	-3.60794000	0.91996200	2.27381800
H	-5.23664700	0.21308500	2.20766100
H	-4.08049400	-3.40875400	2.23253600
H	-4.68496800	-2.38531400	3.53263300
H	-5.49905600	-2.38561100	1.96586400
C	-4.80187700	-0.37329400	-0.75476500
C	-4.80059400	1.15900100	-0.53732400
C	-4.67863900	-0.56751600	-2.28310900
C	-6.15787500	-0.94793900	-0.29692100
H	-5.65071500	1.59295100	-1.08089800
H	-4.89759400	1.45435400	0.50781700
H	-3.88543000	1.60777400	-0.93752800
H	-5.47094900	0.01657000	-2.76980800
H	-3.71966000	-0.20352600	-2.66508000
H	-4.80876200	-1.60581600	-2.59488500
H	-6.38113300	-0.71757500	0.74800600
H	-6.95822600	-0.49968100	-0.90153700
H	-6.22028900	-2.03127300	-0.42613600
H	-2.29162200	-3.90975500	1.27640300
C	-1.87614500	-3.62293300	0.30736900
C	-2.88747400	-2.88296300	-0.59675600
H	-1.58010900	-4.54935800	-0.20183100
H	-0.96552500	-3.03585400	0.45878100
C	-2.17300700	-2.71803800	-1.96111600
C	-4.13123900	-3.77750900	-0.77438000
H	-1.92229600	-3.71930000	-2.33781500
H	-2.78836900	-2.23332600	-2.71884400
H	-1.24020900	-2.15764000	-1.85810000
H	-4.82849400	-3.38345500	-1.51798500
H	-3.80762400	-4.76415300	-1.13274000
H	-4.68058500	-3.93420200	0.15775100

Zero-point correction = 0.776118 (Hartree/Particle)
Thermal correction to Energy = 0.822854
Thermal correction to Enthalpy = 0.823798
Thermal correction to Gibbs Free Energy = 0.693852
Sum of electronic and zero-point Energies = -2496.958992
Sum of electronic and thermal Energies = -2496.912256
Sum of electronic and thermal Enthalpies = -2496.911311
Sum of electronic and thermal Free Energies = -2497.041258
E(RM06L) = -2499.42471288

***cis*-5b**

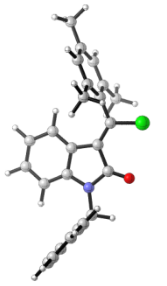

C	-0.79807800	2.01474600	-0.18698000
C	-0.01468600	0.88937300	-0.44733700
C	1.35594000	1.05962500	-0.75513300
C	1.94967500	2.31563000	-0.80171000
C	1.14436300	3.43076300	-0.53910800
C	-0.21110400	3.28486500	-0.23685800
H	-1.85018300	1.91319900	0.05108700
H	3.00632300	2.43041300	-1.01751000
H	1.58770800	4.42237500	-0.56836300
H	-0.81839800	4.16218400	-0.03452200
N	1.94571000	-0.18611600	-0.99154400
C	1.02241300	-1.21918500	-0.84028800
O	1.28829300	-2.40204900	-0.97791500
C	-0.28982600	-0.55694000	-0.48498400
C	3.32673400	-0.42616000	-1.38621400
H	3.57674500	0.24371300	-2.21768000
H	3.34176200	-1.45328300	-1.76426600
C	4.33449800	-0.26321200	-0.26075200
C	4.20975400	-1.01791800	0.91409400
C	5.41373900	0.61670700	-0.38921300
C	5.14376200	-0.88565400	1.94050200
H	3.38179600	-1.71494700	1.01494000
C	6.35334000	0.74821900	0.63684700
H	5.52484700	1.20119200	-1.30062000
C	6.21799600	-0.00091800	1.80552100
H	5.03691900	-1.47723300	2.84594900
H	7.18601400	1.43724100	0.52232200
H	6.94522100	0.10047300	2.60664500
C	-1.46059700	-1.19033600	-0.25441500
Cl	-1.58507700	-2.94584800	-0.34988600
C	-2.74499600	-0.52927200	0.09479300
C	-3.63778100	-0.15100700	-0.93053200
C	-3.06751400	-0.30178000	1.44914700
C	-4.84217100	0.46348300	-0.57626900
C	-4.28455800	0.31571500	1.75308900
C	-5.18699900	0.70345500	0.75789300
H	-5.52963100	0.76126300	-1.36546700
H	-4.53311200	0.49666000	2.79683600
C	-2.11849700	-0.69751100	2.55719000
H	-1.87193000	-1.76469000	2.51445800
H	-1.17290000	-0.14623400	2.49037700
H	-2.55846700	-0.49168800	3.53753300
C	-3.30369200	-0.38682100	-2.38576900
H	-2.40611500	0.16676800	-2.68639100
H	-3.10915500	-1.44663200	-2.58696600
H	-4.12760300	-0.06642000	-3.03038300
C	-6.51123800	1.33818800	1.11364300
H	-6.83207600	2.05281400	0.34798500
H	-7.30172800	0.58081900	1.20176500
H	-6.45891400	1.86632600	2.07151800

Zero-point correction = 0.404056 (Hartree/Particle)
Thermal correction to Energy = 0.429575
Thermal correction to Enthalpy = 0.430519
Thermal correction to Gibbs Free Energy = 0.344385
Sum of electronic and zero-point Energies = -1555.685114
Sum of electronic and thermal Energies = -1555.659594
Sum of electronic and thermal Enthalpies = -1555.658650
Sum of electronic and thermal Free Energies = -1555.744784
E(RM06L) = -1556.37270989

TS_Isom_Vb

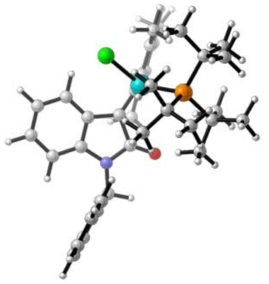

Pd	0.92661100	-0.08538100	0.68197500
C	-1.51692900	2.01763900	1.07673200
C	-1.27583400	2.54767200	2.34235000
C	-2.84067100	1.88844800	0.60391600
C	-2.36504800	2.97269500	3.11052900
H	-0.26471900	2.59514100	2.73327000
C	-3.93003500	2.29889700	1.36200700
C	-3.67041400	2.85092200	2.62436800
H	-2.19410000	3.38431200	4.10070600
H	-4.94626400	2.18376800	1.00028900
H	-4.50533900	3.17708300	3.23846000
P	1.52385500	-2.40476400	-0.20737300
C	-0.62451900	1.50482400	0.02831300
C	0.75450200	1.66628900	-0.06604900
C	1.68631400	2.71093100	-0.35784300
C	3.03432100	2.67202300	0.12203600
C	1.26319200	3.83947800	-1.14223100
C	3.89503800	3.72680100	-0.17249400
C	2.17689800	4.85394800	-1.41105800
C	3.49318500	4.82823600	-0.93375600
H	4.91358300	3.68836000	0.20651400
H	1.85287300	5.69693100	-2.01705600
C	3.35869000	-2.92685900	0.20040100
C	1.28215900	-2.48094000	-2.14043500
C	0.31386800	-3.68523000	0.62674500
C	3.68558300	-2.48658100	1.64665700
H	3.50537000	-1.42144100	1.80559600
H	3.10807000	-3.01973200	2.40033500
H	4.74846200	-2.68992600	1.83641200
C	4.34281400	-2.16670600	-0.71706900
H	5.36232600	-2.35301200	-0.35536100
H	4.30552500	-2.49515600	-1.75751300
H	4.18344200	-1.08493700	-0.69244100
C	3.65294700	-4.43697900	0.06811200
H	3.44894200	-4.82726000	-0.93130800
H	4.71921600	-4.60384000	0.27344800
H	3.09309600	-5.03504100	0.79020900
C	0.73463500	-3.95805800	2.08829800
H	-0.07092000	-4.53067800	2.56726000
H	1.64048400	-4.56465100	2.16162900
H	0.86881000	-3.03494600	2.65842100
C	0.19568900	-5.04166400	-0.09973200
H	-0.47456500	-5.68881100	0.48206000
H	-0.23150000	-4.95701500	-1.10097500
H	1.15487700	-5.55918300	-0.18068300
C	-1.08045400	-3.01715300	0.70373100
H	-1.04429900	-2.10782200	1.31232100
H	-1.49476300	-2.76708000	-0.27411400
H	-1.77467100	-3.71631000	1.18944600
C	-0.22084500	-2.47715400	-2.49811300
H	-0.74463900	-1.62238500	-2.06658400
H	-0.30655300	-2.37713700	-3.58813200
H	-0.73272300	-3.40072200	-2.22078300
C	1.85811200	-1.17978800	-2.75001600
H	2.92103700	-1.03604800	-2.55074600
H	1.73197300	-1.22399400	-3.84014500
H	1.30971500	-0.30543600	-2.39038100
C	1.93324200	-3.69835400	-2.83211300
H	1.67854900	-3.66788800	-3.89998500
H	3.02351300	-3.69374600	-2.76605500
H	1.57134900	-4.65192100	-2.43946900
C	-0.12551900	3.95655900	-1.71997300
H	-0.36255700	3.11408200	-2.37800400
H	-0.88890400	3.98602200	-0.93391100
H	-0.21386600	4.87798700	-2.30286700
C	3.54654600	1.53501700	0.95987600
H	2.97100200	1.40819200	1.88480700
H	3.47218900	0.58395900	0.41757400
H	4.59744100	1.68798700	1.22586300
C	4.43918600	5.96843900	-1.21357000
H	5.48404100	5.64648300	-1.15943000
H	4.26288300	6.40223700	-2.20373900
H	4.30667300	6.77447700	-0.47892800
C	-1.52122500	1.07498100	-1.10315000
O	-1.21285500	0.65816000	-2.21536600
N	-2.82368400	1.31542200	-0.67992600
C	-3.97522500	1.12115000	-1.54428700
H	-4.47973100	2.08518000	-1.69326700
H	-3.55766500	0.81692100	-2.51050300
C	-4.97897900	0.09196600	-1.04910400
C	-4.56828300	-1.07050300	-0.38617000
C	-6.34345100	0.28205300	-1.29856800
C	-5.50415400	-2.02395300	0.01620500
H	-3.51401500	-1.22346500	-0.17387800
C	-7.28087300	-0.67410300	-0.90319900
H	-6.67556700	1.18487400	-1.80750600
C	-6.86297700	-1.83071400	-0.24302500
H	-5.17023700	-2.91835400	0.53538800
H	-8.33641400	-0.51049000	-1.10397200
H	-7.59076200	-2.57347300	0.07229300
Cl	1.03277200	-0.44892500	3.12692100

Zero-point correction = 0.777014 (Hartree/Particle)
Thermal correction to Energy = 0.823381
Thermal correction to Enthalpy = 0.824325
Thermal correction to Gibbs Free Energy = 0.697054
Sum of electronic and zero-point Energies = -2496.962441
Sum of electronic and thermal Energies = -2496.916074
Sum of electronic and thermal Enthalpies = -2496.915130
Sum of electronic and thermal Free Energies = -2497.042401
E(RM06L) = -2499.44140973

Vb

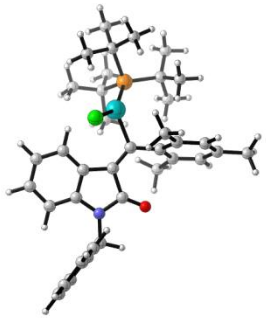

Pd	1.12304400	-0.55546700	1.05677900
C	-1.86762100	-1.34756800	-0.37923700
C	-1.25715200	-2.58095200	-0.16158600
C	-3.19734900	-1.32366200	-0.85748500
C	-1.96233100	-3.76049000	-0.42704500
H	-0.25234500	-2.62392400	0.24335000
C	-3.91350300	-2.48498200	-1.12228300
C	-3.27428400	-3.71141000	-0.90264500
H	-1.48702500	-4.72064000	-0.24734800
H	-4.94095400	-2.44505400	-1.46787900
H	-3.81462900	-4.63393100	-1.09735900
P	3.18091000	-0.73780300	-0.25034800
C	4.15705800	0.86543100	-0.75817500
C	4.20739900	-1.66482100	1.13400600
C	3.02012500	-1.90920700	-1.79367600
C	-1.41755200	0.04650000	-0.24278700
C	-0.26198500	0.61486600	0.17007400
C	1.85934300	-1.38565500	-2.67032000
H	2.04265600	-0.39335900	-3.08217800
H	1.72466000	-2.07177500	-3.51679900
H	0.91980300	-1.36028000	-2.11074200
C	2.60200300	-3.32679000	-1.34469900
H	2.35148000	-3.90816100	-2.24113600
H	3.39685900	-3.86582100	-0.82518100
H	1.71106600	-3.31082800	-0.71099800

C	4.29330300	-2.03447900	-2.65705400
H	4.09896900	-2.75379500	-3.46369800
H	4.57676900	-1.09273400	-3.13203400
H	5.15259200	-2.40408400	-2.09227900
C	3.60464500	1.42082400	-2.08810500
H	4.06613000	2.40064900	-2.26557100
H	3.84355800	0.79280000	-2.94886600
H	2.52620500	1.57699800	-2.04763400
C	3.88545600	1.95077700	0.30996900
H	4.29175000	1.70247500	1.29040400
H	4.36514200	2.88317600	-0.01560100
H	2.81760800	2.14875500	0.42157100
C	5.68090200	0.67817600	-0.91517600
H	6.17602500	0.42556600	0.02490200
H	5.94232100	-0.08186500	-1.65571900
H	6.11183300	1.62859200	-1.25720200
C	5.45625100	-2.41832500	0.63249200
H	6.16399600	-1.77048100	0.11087600
H	5.97923600	-2.84247900	1.50022700
H	5.20658100	-3.25283800	-0.02701700
C	3.27394200	-2.67528200	1.84844900
H	3.84712500	-3.17178000	2.64295500
H	2.42210500	-2.19059300	2.34548900
H	2.88180500	-3.45279700	1.19441500
C	4.64370500	-0.66109100	2.22600500
H	5.42727100	0.02244400	1.89386200
H	3.80147400	-0.07348500	2.60568200
H	5.05017300	-1.23110800	3.07118100
Cl	-0.25358200	-1.12748500	2.90370000
C	-2.60627500	0.89387700	-0.66787000
O	-2.72251300	2.11019000	-0.71776700
N	-3.61890200	0.00262600	-1.00831200
C	-4.91425800	0.44186900	-1.49893600
H	-5.10827800	-0.02827900	-2.47178900
H	-4.80477400	1.51895600	-1.66454000
C	-6.07291500	0.16634100	-0.55425400
C	-5.97418500	0.46748100	0.81032000
C	-7.27398500	-0.35538600	-1.04701500
C	-7.05553900	0.24692500	1.66271200
H	-5.04627300	0.87323300	1.20426900
C	-8.36056400	-0.57172700	-0.19611200
H	-7.36136800	-0.59360800	-2.10546500
C	-8.25269900	-0.27247000	1.16227700
H	-6.96327200	0.48104900	2.71990200
H	-9.28600100	-0.97958100	-0.59446200
H	-9.09384800	-0.44487200	1.82841600
C	0.09487000	2.03827800	0.08635400
C	0.26601900	2.83301600	1.25711000
C	0.26598200	2.64995500	-1.18979300
C	0.64558700	4.16917000	1.12446000
C	0.66279400	3.98734900	-1.25953800
C	0.86270100	4.76821200	-0.11993000
H	0.76541400	4.76485700	2.02716400
H	0.79636100	4.43861700	-2.24100400
C	-0.04495700	1.94883100	-2.49210000
H	0.53939600	2.37720500	-3.31370600
H	0.13863100	0.87424900	-2.45681100
H	-1.10491700	2.09019900	-2.73355700
C	0.01959400	2.29258200	2.64267500
H	-0.92426500	1.74505800	2.70437400
H	0.79496800	1.58254800	2.95179800
H	-0.00292200	3.11023200	3.37035800
C	1.25092700	6.22318100	-0.22568300
H	0.36584600	6.87132700	-0.17007900
H	1.91986200	6.52124100	0.58936900
H	1.75216100	6.43775000	-1.17548400

Zero-point correction = 0.777600 (Hartree/Particle)
Thermal correction to Energy = 0.824728
Thermal correction to Enthalpy = 0.825672
Thermal correction to Gibbs Free Energy = 0.695490
Sum of electronic and zero-point Energies = -2496.979689
Sum of electronic and thermal Energies = -2496.932561
Sum of electronic and thermal Enthalpies = -2496.931617
Sum of electronic and thermal Free Energies = -2497.061798
E(RM06L) = -2499.46161949

TS_RE_Vb

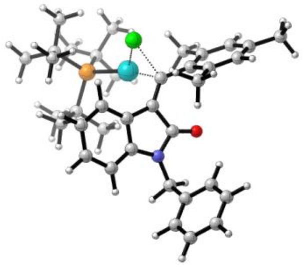

C	0.56752500	-1.73066800	2.19690300
C	1.18717300	-1.24242100	1.04289400
C	2.09353200	-2.08111900	0.34731100
C	2.35511700	-3.38486700	0.75283500
C	1.70304400	-3.86098100	1.89655100
C	0.82793900	-3.04088200	2.61386200
H	-0.10487900	-1.09643000	2.75982400
H	3.06390800	-4.00755800	0.21748600
H	1.89539300	-4.87572300	2.23425100
H	0.34473000	-3.41921100	3.51026600
N	2.64589300	-1.38416700	-0.73192300
C	2.20712600	-0.06382600	-0.75508300
O	2.58417200	0.77220800	-1.56673100
C	1.19838500	0.06299000	0.36661100
C	3.59271200	-1.90505600	-1.71038300
H	3.50979000	-1.24104200	-2.57621000
H	3.26121100	-2.90429500	-2.01479300
C	5.03150100	-1.95159300	-1.22384900
C	5.71756500	-3.16550000	-1.11942200
C	5.69831400	-0.76298100	-0.89162200
C	7.04472200	-3.19983300	-0.68293300
H	5.21374900	-4.09263100	-1.38613600
C	7.02110400	-0.79578100	-0.45259200
H	5.17510000	0.18450400	-0.99170000
C	7.69793900	-2.01460800	-0.34546500
H	7.56413600	-4.15143000	-0.60567500
H	7.52808000	0.13167200	-0.19927900
H	8.72940200	-2.03788200	-0.00399400
C	0.55481600	1.27087600	0.55427800
Cl	-0.58812200	1.46729500	2.55765900
Pd	-1.31598900	0.59942500	0.22308700
P	-3.38875800	-0.60056000	-0.34587100
C	-2.91247800	-2.22271500	-1.31637700
C	-4.35069500	-1.10597800	1.27083800
C	-4.58642900	0.45235200	-1.46525800
C	-1.79458400	-1.86425300	-2.32569300
C	-2.27969700	-3.24099100	-0.34109100
C	-4.06212600	-2.92182700	-2.07025100
C	-3.30815800	-1.53831300	2.32955400
C	-5.40118700	-2.22311300	1.10584000
C	-5.04795800	0.13717200	1.86745700
C	-4.63524700	1.89245200	-0.90092900
C	-3.98487000	0.57860600	-2.88283500
C	-6.03001700	-0.07587900	-1.59439800
H	-1.45002900	-2.78773500	-2.81026900
H	-2.11957200	-1.18527100	-3.11449300
H	-0.93612600	-1.40980700	-1.81988800
H	-1.85789600	-4.06585600	-0.93034600
H	-1.46184000	-2.80415000	0.23957300
H	-3.00428000	-3.67978200	0.34835800
H	-4.46501500	-2.31104700	-2.88206000
H	-3.67890200	-3.84544200	-2.52519500
H	-4.88888500	-3.20230700	-1.41283500
H	-3.83885900	-1.77914900	3.26086500
H	-2.73274500	-2.41896800	2.04460600
H	-2.60602100	-0.72717600	2.54353900
H	-4.95459500	-3.18060400	0.82672700
H	-5.91003600	-2.37596400	2.06737400
H	-6.16857100	-1.97781100	0.36734800
H	-5.91682600	0.46088400	1.28965900
H	-5.40860000	-0.11952800	2.87199200
H	-4.35721400	0.97958400	1.97560000
H	-3.63374000	2.32375100	-0.82594700
H	-5.22077900	2.51909200	-1.58719000

H	-5.10357000	1.95932700	0.08056300
H	-4.56412600	1.32505900	-3.44196800
H	-2.94527800	0.92165500	-2.85785400
H	-4.03015600	-0.35275800	-3.45125400
H	-6.56805700	-0.05423500	-0.64352500
H	-6.58391000	0.56909200	-2.29037400
H	-6.07575200	-1.09410900	-1.98773900
C	1.09893300	2.60329300	0.31036800
C	0.43663600	3.54506700	-0.51591100
C	2.33013000	2.96481400	0.93245200
C	1.02964800	4.78612200	-0.75098300
C	2.86501400	4.22733600	0.68465200
C	2.24403500	5.14906500	-0.16508800
H	0.52217800	5.49369300	-1.40309800
H	3.79889400	4.50202400	1.17064000
C	3.06992200	2.03969800	1.87066100
H	2.39211600	1.54126700	2.56933700
H	3.61391100	1.26090300	1.32266600
H	3.80709000	2.60303300	2.45135100
C	-0.89851500	3.25452300	-1.14820400
H	-0.91448700	2.27050300	-1.63930600
H	-1.69350400	3.26068300	-0.39184300
H	-1.15133800	4.00828800	-1.90173600
C	2.87693600	6.48969200	-0.44733400
H	3.36455300	6.89848300	0.44462800
H	3.64693500	6.40369300	-1.22596700
H	2.13784500	7.21782300	-0.79700600

Zero-point correction = 0.776237 (Hartree/Particle)
Thermal correction to Energy = 0.822983
Thermal correction to Enthalpy = 0.823927
Thermal correction to Gibbs Free Energy = 0.693925
Sum of electronic and zero-point Energies = -2496.957778
Sum of electronic and thermal Energies = -2496.911032
Sum of electronic and thermal Enthalpies = -2496.910088
Sum of electronic and thermal Free Energies = -2497.040091
E(RM06L) = -2499.42774262

***trans*-5b**

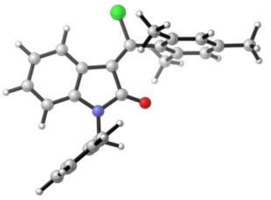

C	0.87530200	3.34387400	0.56552600
C	0.76194400	2.04243800	0.07295700
C	1.91103100	1.40969400	-0.46139200
C	3.14837500	2.03950800	-0.50611500
C	3.23628300	3.34535800	-0.00771000
C	2.11637100	3.99023500	0.51982100
H	0.01570000	3.85453300	0.97927600
H	4.02348500	1.53307200	-0.89833200
H	4.19425000	3.85765600	-0.03069500
H	2.20489400	5.00246300	0.90307100
N	1.58222500	0.12492400	-0.90928500
C	0.23997900	-0.15642000	-0.68255800
O	-0.30175400	-1.21361700	-0.96753500
C	-0.34553200	1.08429100	-0.03832700
C	2.47876900	-0.81880500	-1.56426700
H	3.03588500	-0.28836200	-2.34540300
H	1.82277700	-1.54650100	-2.05212600
C	3.43843800	-1.52341900	-0.62041700
C	2.94394800	-2.29186300	0.44282500
C	4.82125800	-1.43977100	-0.81075000
C	3.81960500	-2.95370000	1.30207300
H	1.86957200	-2.37440700	0.58591100
C	5.70087500	-2.10552100	0.04714700
H	5.21522300	-0.85383900	-1.63908800
C	5.20113400	-2.86142700	1.10738500
H	3.42458600	-3.54736600	2.12226000
H	6.77317400	-2.02988900	-0.11284500
H	5.88217700	-3.37869700	1.77784000
C	-1.64983400	1.13328400	0.30528900
Cl	-2.30551800	2.60674200	1.05354900
C	-2.67044200	0.06938300	0.13327100
C	-2.91847700	-0.83485300	1.18570000
C	-3.41119300	-0.00102400	-1.06416700
C	-3.91516600	-1.79954300	1.01853400
C	-4.39851500	-0.98151300	-1.18361500
C	-4.66980800	-1.88830400	-0.15454900
H	-4.10439900	-2.50183200	1.82787400
H	-4.96842800	-1.03966300	-2.10882900
C	-3.13369500	0.93784500	-2.21493900
H	-3.14142800	1.98776300	-1.90026900
H	-2.14913700	0.73689100	-2.65458200
H	-3.88194000	0.81738100	-3.00422200
C	-2.11521600	-0.78957800	2.46472900
H	-1.05790800	-1.01124700	2.27588100
H	-2.15832600	0.19742500	2.94004800
H	-2.48774500	-1.52540200	3.18379500
C	-5.76573000	-2.91841900	-0.29818400
H	-6.73880100	-2.50841600	0.00473900
H	-5.86529300	-3.25515800	-1.33580600
H	-5.57427700	-3.79706000	0.32684300

Zero-point correction = 0.403974 (Hartree/Particle)
Thermal correction to Energy = 0.429526
Thermal correction to Enthalpy = 0.430471
Thermal correction to Gibbs Free Energy = 0.344364
Sum of electronic and zero-point Energies = -1555.686377
Sum of electronic and thermal Energies = -1555.660824
Sum of electronic and thermal Enthalpies = -1555.659880
Sum of electronic and thermal Free Energies = -1555.745987
E(RM06L) = -1556.37306993

e) reaction of aryl chloride **4a** (R = TIPS), L = PA-Ph

4a

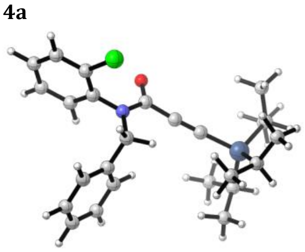

C	3.80648300	-1.85927200	0.33798900
C	5.12585400	-2.17919800	0.01730200
C	3.08565100	-0.92136100	-0.41609100
C	5.73679900	-1.56403500	-1.07472300
H	5.65877700	-2.90895600	0.61733600
C	3.71717500	-0.31612600	-1.50783700
C	5.03213000	-0.63378000	-1.84037400
H	6.76300900	-1.81717000	-1.32528200
H	3.15585700	0.40722000	-2.08953600
H	5.50426800	-0.15494700	-2.69297700
C	0.71901600	-1.14611400	-0.80203800
C	-0.63851700	-0.75093000	-0.44065600
C	-1.81557300	-0.49451400	-0.24283900
O	0.91484500	-1.94630800	-1.70751800
N	1.74256300	-0.56589300	-0.07489700
C	1.54114900	0.41003000	1.00868700
H	0.48280900	0.36928300	1.27945900
H	2.11134300	0.07489200	1.88136300
C	1.93870800	1.82855900	0.63995300
C	1.26459000	2.51779900	-0.37891100
C	2.97490400	2.47634700	1.32008000
C	1.62166600	3.82473900	-0.70802800
H	0.45501900	2.02672700	-0.91384900
C	3.33298600	3.78742600	0.99523000
H	3.50653400	1.95125100	2.11056900
C	2.65778100	4.46408600	-0.02052500

H	1.08897000	4.34725000	-1.49832400
H	4.14098900	4.27567700	1.53363900
H	2.93484300	5.48325700	-0.27639400
Cl	3.05283700	-2.66387100	1.70518500
Si	-3.61386600	-0.12783000	0.01798700
C	-4.63528600	-1.59630300	-0.68905200
C	-3.93524800	0.07558700	1.90524800
C	-4.01602400	1.50936700	-0.91364000
H	-5.58538500	-1.56528300	-0.13082900
C	-4.98291300	-1.47558400	-2.18567200
C	-3.97408900	-2.96465000	-0.42209200
H	-4.82223100	0.72824600	1.95812800
C	-4.28793500	-1.23067000	2.64255900
C	-2.78423500	0.78953900	2.64255300
H	-5.10168500	1.45745200	-1.09908700
C	-3.75322700	2.79034000	-0.09817400
C	-3.30998900	1.61325700	-2.28058800
H	-5.62583800	-2.31089100	-2.49449000
H	-5.51512200	-0.54776100	-2.42237100
H	-4.08197200	-1.51642700	-2.80954100
H	-4.62356900	-3.77839700	-0.77228500
H	-3.02048700	-3.04854400	-0.95519700
H	-3.77334800	-3.13825100	0.63961200
H	-4.53437400	-1.02115800	3.69235300
H	-5.14792200	-1.74534300	2.20105100
H	-3.44316300	-1.93014700	2.64421400
H	-3.05465600	0.97584700	3.69082500
H	-1.87922000	0.17039400	2.64284500
H	-2.52511800	1.75274000	2.19269200
H	-2.68512500	2.91474400	0.11946500
H	-4.07075700	3.67547500	-0.66606400
H	-4.29303400	2.80278100	0.85467700
H	-3.52415200	0.76261700	-2.93380300
H	-3.62527300	2.52403700	-2.80778800
H	-2.22193000	1.66328600	-2.15610500

Zero-point correction = 0.513354 (Hartree/Particle)
Thermal correction to Energy = 0.545536
Thermal correction to Enthalpy = 0.546480
Thermal correction to Gibbs Free Energy = 0.445419
Sum of electronic and zero-point Energies = -1851.042881
Sum of electronic and thermal Energies = -1851.010699
Sum of electronic and thermal Enthalpies = -1851.009755
Sum of electronic and thermal Free Energies = -1851.110816
E(RM06L) = -1851.85204651

PC_4a_PAPh

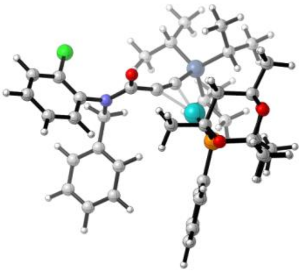

C	4.63337800	-2.16737700	-2.23474400
C	5.13756100	-3.23843000	-2.97266800
C	3.44773700	-2.29150800	-1.49566900
C	4.44894100	-4.45082300	-2.98246600
H	6.05612400	-3.11330000	-3.53589100
C	2.77238400	-3.51621200	-1.51910800
C	3.26417300	-4.59023000	-2.25780000
H	4.84094100	-5.28317900	-3.56001300
H	1.85700100	-3.60890600	-0.94456800
H	2.72494500	-5.53277700	-2.26471400
C	1.94672500	-0.42778500	-1.26891500
C	1.46527800	0.70977800	-0.47021000
C	1.35683300	1.86768700	-0.00235800
Si	1.56532100	3.64965000	0.50381000
C	3.30513800	4.15020100	-0.14129500
H	3.30244900	3.80830400	-1.18763400
C	0.13375100	4.58564000	-0.36243300
H	-0.75644100	3.99202700	-0.10226000
C	1.42759100	3.65915100	2.41950400
H	2.08327800	2.83429400	2.73953600
C	3.59177100	5.66511000	-0.15909600
H	3.57639500	6.10136200	0.84647300
H	4.58789600	5.86036400	-0.57970100
H	2.86784300	6.21507300	-0.76966700
C	4.43324300	3.38829400	0.58176800
H	4.28725100	2.30311700	0.53860100
H	5.40641000	3.60728400	0.12181100
H	4.50406600	3.67386900	1.63883700
C	0.27117200	4.54321200	-1.89756300
H	-0.61528800	4.98042300	-2.37705700
H	1.14054400	5.11683700	-2.24326800
H	0.37772700	3.51777800	-2.26878600
C	-0.11318000	6.02552400	0.12907100
H	0.74127300	6.68290600	-0.07005000
H	-0.98036400	6.46098300	-0.38607000
H	-0.31905500	6.06798600	1.20431400
C	0.00253600	3.31914200	2.89909600
H	-0.36377500	2.38146000	2.45743000
H	-0.02292000	3.20583300	3.99178200
H	-0.71107900	4.11054800	2.63764400
C	1.94114400	4.94169900	3.10424700
H	1.36359300	5.82570100	2.80929300
H	1.85762800	4.85251300	4.19610900
H	2.99296900	5.14212300	2.87376800
N	2.94380900	-1.20467400	-0.71218700
C	3.52900900	-0.99406800	0.62318400
H	3.14093300	-0.04119600	0.98986900
H	4.61378000	-0.88522800	0.51323800
C	3.21502100	-2.10703800	1.60671400
C	4.23856300	-2.90738700	2.12438700
C	1.89465400	-2.34367500	2.01783900
C	3.95516300	-3.92648900	3.03743800
H	5.26532300	-2.73326600	1.81004900
C	1.60841700	-3.36349300	2.92522000
H	1.08943400	-1.72580700	1.62484800
C	2.63902600	-4.15757900	3.43774100
H	4.76190800	-4.53996500	3.43008500
H	0.58021300	-3.53428700	3.23262000
H	2.41545100	-4.95152000	4.14564300
O	1.47626500	-0.65904400	-2.37791300
Cl	5.50827000	-0.64423500	-2.25481300
C	-2.91996300	-1.20461500	-1.47718300
C	-4.13895600	0.54943800	0.27326400
C	-2.94405900	-0.00896100	-2.44598800
C	-5.36388000	-0.35378300	0.08256200
H	-3.05084300	-0.39305300	-3.46748500
H	-2.00945100	0.55580500	-2.37972800
C	-4.14948100	0.89259900	-2.14295500
C	-5.34929200	-0.97321300	-1.32359600
H	-5.39952500	-1.13359200	0.84866000
H	-6.26869700	0.25947600	0.17449000
C	-2.73582600	-1.80616200	1.48625700
C	-3.45605600	-2.99918700	1.29143500
C	-2.11120500	-1.59611100	2.72856900
C	-3.54825500	-3.94567200	2.31406600
H	-3.94072100	-3.18425700	0.33967500
C	-2.22438300	-2.53382600	3.75678900
H	-1.52343100	-0.69386900	2.88020700
C	-2.94124700	-3.71445400	3.55012800
H	-4.10130600	-4.86583000	2.14347200
H	-1.74449000	-2.34486400	4.71357500
H	-3.02305900	-4.45070500	4.34540800

P	-2.51879300	-0.46603700	0.23043700
C	-1.91212200	-2.26272800	-1.90872200
H	-2.21614500	-2.66970100	-2.87999700
H	-0.91618900	-1.82022400	-2.01018400
H	-1.86567500	-3.08787100	-1.19094000
C	-4.23360300	1.40232300	1.53028300
H	-3.36506200	2.06351100	1.61172600
H	-5.13920200	2.01900200	1.49134400
H	-4.27839600	0.76977300	2.42332100
C	-4.29613000	2.05620500	-3.10536200
H	-3.41556600	2.70217600	-3.04828900
H	-4.40482800	1.68461600	-4.12802400
H	-5.18425200	2.63632700	-2.84022200
C	-6.57568400	-1.81282900	-1.63273900
H	-7.47774300	-1.19954800	-1.55534200
H	-6.49769900	-2.20507600	-2.65027000
H	-6.64908600	-2.64987300	-0.93216900
O	-5.32683300	0.09403400	-2.26496200
O	-4.06030600	1.46697800	-0.83372100
O	-4.22054400	-1.83661100	-1.49673800
Pd	-0.55215000	0.77390300	0.27130900

Zero-point correction= 0.858989 (Hartree/Particle)
Thermal correction to Energy= 0.913704
Thermal correction to Enthalpy= 0.914648
Thermal correction to Gibbs Free Energy= 0.763137
Sum of electronic and zero-point Energies= -3166.874581
Sum of electronic and thermal Energies= -3166.819865
Sum of electronic and thermal Enthalpies= -3166.818921
Sum of electronic and thermal Free Energies= -3166.970433
E(RM06L) = -3169.52185104

TS_OA_4a_PAPh

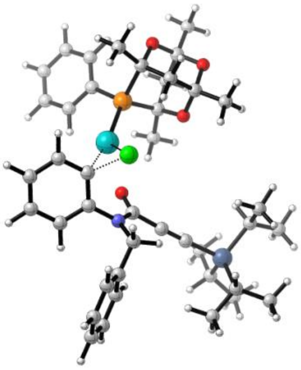

Pd	1.53620400	1.55286300	-0.31109400
C	0.31991900	3.12143400	-0.73932500
C	1.13974400	4.26257000	-0.73913800
C	-0.93502000	3.13915300	-0.08662200
C	0.75318100	5.38806500	-0.00354500
H	2.04894500	4.26750700	-1.33129500
C	-1.29302000	4.27156100	0.64415600
C	-0.45959100	5.39589100	0.68385100
H	1.39721000	6.26358100	0.00891700
H	-2.23332000	4.26468500	1.18586900
H	-0.76851200	6.27360900	1.24356700
C	-1.49081100	0.86126900	0.49537300
C	-2.43103600	-0.24262700	0.41910500
C	-3.15056700	-1.22883300	0.42556400
Si	-4.20016400	-2.76486800	0.44330900
C	-5.74954600	-2.33409700	-0.60099600
H	-5.33519300	-1.90742700	-1.52760400
C	-3.08791500	-4.11053800	-0.34876700
H	-2.13336200	-4.01500800	0.19038000
C	-4.58063300	-3.06429800	2.29906800
H	-4.94581100	-2.08936800	2.65727000
C	-6.62259600	-3.53832100	-1.00676300
H	-7.05219500	-4.04839500	-0.13670700
H	-7.46231100	-3.20768000	-1.63309600
H	-6.06049700	-4.28178700	-1.58207300
C	-6.60362200	-1.23652400	0.06477100
H	-6.01758900	-0.33859700	0.29156600
H	-7.42728600	-0.93295800	-0.59531000
H	-7.05364000	-1.58749500	1.00193400
C	-2.80236600	-3.80981800	-1.83399500
H	-2.07361600	-4.52438200	-2.23938600
H	-3.70977600	-3.89084700	-2.44577800
H	-2.39107200	-2.80407100	-1.97565800
C	-3.57575500	-5.56117700	-0.16359900
H	-4.54484100	-5.73650500	-0.64521100
H	-2.86100200	-6.26359000	-0.61354300
H	-3.67697400	-5.83264200	0.89283900
C	-3.30081200	-3.39234400	3.09434100
H	-2.52168600	-2.63619200	2.94740000
H	-3.51639900	-3.44394200	4.17010500
H	-2.88252100	-4.36357800	2.80192500
C	-5.68779500	-4.09824800	2.58537300
H	-5.42096000	-5.09879200	2.22565800
H	-5.86195000	-4.18099600	3.66687700
H	-6.64179000	-3.82353300	2.12238400
Cl	0.37341400	2.04283800	-2.58386000
N	-1.82587400	2.01719100	-0.16068200
C	-2.96376300	2.11858100	-1.09320900
H	-3.30594300	1.09897200	-1.29073600
H	-2.57834800	2.51826600	-2.03613400
C	-4.11760900	2.96772300	-0.59068400
C	-4.58532700	4.04365400	-1.35217200
C	-4.75434700	2.67108800	0.62365200
C	-5.67062000	4.80847500	-0.91647300
H	-4.09519600	4.28636400	-2.29221400
C	-5.83418000	3.43624800	1.06365600
H	-4.39777100	1.83960300	1.22645200
C	-6.29675000	4.50730500	0.29311000
H	-6.02025700	5.64202000	-1.51984200
H	-6.31735800	3.19554600	2.00700900
H	-7.13869700	5.10264200	0.63606800
O	-0.44632500	0.73099200	1.14777400
P	3.28896900	-0.00776300	0.15920700
C	4.34227400	-0.51005600	-1.34019000
C	2.75622600	-1.80832600	0.50756900
C	4.44500800	0.47225800	1.51631100
C	3.34458500	-1.07573900	-2.36762200
C	5.14406000	0.65696100	-1.90343800
O	5.29941100	-1.54387500	-1.01700000
C	3.97523800	-2.72493800	0.67179100
C	1.79623300	-1.88933900	1.68531900
O	2.01487900	-2.22674800	-0.65592400
C	5.79794700	0.09635500	1.60705000
C	3.90951900	1.29999300	2.51954800
H	3.88315900	-1.27246600	-3.30260000
H	2.54247200	-0.35918600	-2.56965700
C	2.76565900	-2.40568900	-1.85917200
H	5.71695000	0.31877300	-2.77447400
H	4.46988000	1.46139900	-2.21420100
H	5.84433400	1.05460200	-1.16280300
C	4.75478500	-2.81656200	-0.64841200
H	4.62280600	-2.37275300	1.47994600
H	3.62262500	-3.73121300	0.92940800
H	0.92854200	-1.24273700	1.52682000
H	1.45460800	-2.92466400	1.80533100
H	2.30044100	-1.58154700	2.60823100
C	6.58441700	0.53683100	2.67328500
H	6.23327400	-0.53317800	0.83933400
C	4.69438200	1.72454600	3.59248100
H	2.87137200	1.61716500	2.45136600
C	1.82836100	-3.07516200	-2.84689100
O	3.86165600	-3.29707400	-1.64721900
C	5.92739200	-3.77946000	-0.60039600
C	6.03568800	1.34580300	3.67045200
H	7.63028100	0.24462800	2.72390200
H	4.25924500	2.35928100	4.35982600
H	0.96724400	-2.42960700	-3.04052100
H	2.35162900	-3.27214900	-3.78675800
H	1.47892000	-4.02279800	-2.42787000
H	5.57413600	-4.78632300	-0.36111300

H	6.42086900	-3.79735300	-1.57590400
H	6.64846300	-3.46212400	0.15868400
H	6.65133900	1.68317600	4.50018100

Zero-point correction = 0.857952 (Hartree/Particle)
Thermal correction to Energy = 0.912314
Thermal correction to Enthalpy = 0.913258
Thermal correction to Gibbs Free Energy = 0.762534
Sum of electronic and zero-point Energies = -3166.842648
Sum of electronic and thermal Energies = -3166.788287
Sum of electronic and thermal Enthalpies = -3166.787343
Sum of electronic and thermal Free Energies = -3166.938067
E(RM06L) = -3169.48472737

IVa_PAPh

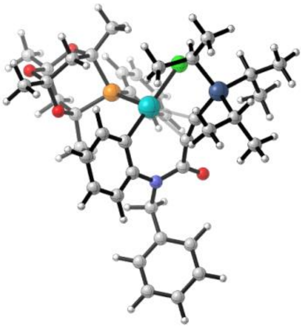

Pd	-0.06729200	-0.60547800	0.03421800
C	0.35164400	0.86453500	-1.32032700
C	-0.06289100	0.67887800	-2.64365400
C	1.19704500	1.95894100	-1.04255000
C	0.29648700	1.56708000	-3.66104700
H	-0.69421100	-0.16801400	-2.89244800
C	1.56218400	2.85108100	-2.06385600
C	1.10065900	2.66605800	-3.36495100
H	-0.05802900	1.40076400	-4.67516500
H	2.23094000	3.67443000	-1.83793100
H	1.39007100	3.36634900	-4.14353800
C	2.12021400	-0.17801400	0.46133100
C	2.27425600	-1.35041900	0.09795600
Si	2.91191300	-3.08660100	-0.23420800
C	4.62001900	-2.79421500	-1.06600500
C	1.67793700	-4.03462900	-1.35341500
C	3.07235600	-3.98712200	1.45451900
H	5.04675600	-3.80302400	-1.18478300
C	4.52647000	-2.15847700	-2.46659700
C	5.58516300	-1.98280800	-0.17871700
H	0.88167600	-4.32680000	-0.65452100
C	1.01286200	-3.20105500	-2.46328500
C	2.29389900	-5.32259600	-1.93907900
H	2.08942200	-4.46005300	1.58893100
C	3.28501400	-3.05450700	2.66278400
C	4.14006800	-5.10177200	1.43362500
H	5.52985000	-2.00940200	-2.88881800
H	3.96565800	-2.78234800	-3.17019900
H	4.03928400	-1.17614000	-2.43065200
H	6.56607100	-1.88852600	-0.66440500
H	5.20918600	-0.96734200	-0.00459000
H	5.74574400	-2.44660800	0.79999800
H	1.73257100	-2.84375100	-3.20852100
H	0.26488900	-3.80563600	-2.99556200
H	0.50004800	-2.32397400	-2.05298000
H	1.53900000	-5.88374000	-2.50633700
H	3.11887400	-5.10457300	-2.62909500
H	2.67877900	-5.99389800	-1.16333600
H	2.46389000	-2.34030000	2.76529200
H	3.33355000	-3.64468600	3.58817600
H	4.22120300	-2.48762700	2.59011600
H	5.15242200	-4.69369600	1.32527900
H	4.11919000	-5.66298500	2.37738400
H	3.98852000	-5.82466600	0.62357400
Cl	-0.41100000	-2.57278200	1.46005700
N	1.71594500	2.18570400	0.27347400
C	1.71044100	3.53898000	0.86512300
H	0.82707700	4.05296200	0.47640400
H	1.57909300	3.39318600	1.94143600
C	2.95003700	4.38506000	0.61816200
C	4.21529400	3.96080200	1.05191800
C	2.83450100	5.63188900	-0.00897600
C	5.33682000	4.76369600	0.84513800
H	4.31100400	3.00828700	1.56348200
C	3.95682200	6.43917900	-0.21055000
H	1.85748300	5.97556900	-0.34295500
C	5.21259600	6.00413200	0.21315600
H	6.31095200	4.42257000	1.18572900
H	3.84777900	7.40373300	-0.69944500
H	6.08869800	6.62767500	0.05615400
C	2.18812000	1.16129700	1.05383900
O	2.63911300	1.31507200	2.18737800
P	-2.34274200	-0.04759700	0.39094000
C	-3.67127400	-1.21804400	-0.33623800
C	-3.14068200	1.50791600	-0.39292600
C	-2.59424600	0.11589200	2.20623200
C	-3.54453100	-1.09862500	-1.86578800
C	-3.56606700	-2.66016100	0.13795400
O	-4.98694200	-0.77250700	0.04945100
C	-4.60198200	1.63548500	0.06926700
C	-2.35414900	2.77843800	-0.10618000
O	-3.12145900	1.31608800	-1.81657100
C	-3.78847400	-0.22477100	2.86527100
C	-1.52056600	0.61432500	2.96362200
H	-4.21408400	-1.83773000	-2.32083600
H	-2.52274400	-1.31816900	-2.18912600
C	-3.98152200	0.29805800	-2.33685700
H	-4.38265000	-3.23088900	-0.31941900
H	-2.60544600	-3.09513200	-0.14270400
H	-3.65229300	-2.73049900	1.22449100
C	-5.43827800	0.47717100	-0.48546700
H	-4.66953700	1.67435800	1.15958000
H	-5.00297800	2.57331100	-0.33322400
H	-1.34141800	2.70375500	-0.50502000
H	-2.85604500	3.62785200	-0.58369800
H	-2.30594000	2.96241300	0.97240000
C	-3.89923900	-0.06225300	4.24730400
H	-4.62512000	-0.61963300	2.30164700
C	-1.64209600	0.78741000	4.34187100
H	-0.57489600	0.84107700	2.48100500
C	-3.97176900	0.46481400	-3.84496700
O	-5.32529900	0.49395000	-1.90341200
C	-6.91592300	0.57192300	-0.15305100
C	-2.83182300	0.44762200	4.98775900
H	-4.82472500	-0.34010000	4.74468800
H	-0.79819300	1.16904900	4.90941500
H	-2.95670900	0.33998300	-4.23135100
H	-4.63017300	-0.27492800	-4.30853800
H	-4.32697700	1.46716000	-4.09836600
H	-7.33250100	1.49485500	-0.56573800
H	-7.44033000	-0.28218000	-0.58965500
H	-7.06203100	0.56465200	0.93090300
H	-2.92317200	0.57080600	6.06352900

Zero-point correction = 0.859824 (Hartree/Particle)
Thermal correction to Energy = 0.914187
Thermal correction to Enthalpy = 0.915132
Thermal correction to Gibbs Free Energy = 0.768978
Sum of electronic and zero-point Energies = -3166.882579
Sum of electronic and thermal Energies = -3166.828215
Sum of electronic and thermal Enthalpies = -3166.827271
Sum of electronic and thermal Free Energies = -3166.973425
E(RM06L) = -3169.55042090

TS_AI_IVa_PAPh

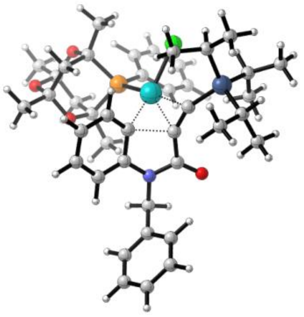

Pd	-0.07799000	-0.82327300	-0.33825900
C	0.63450300	1.06345400	-1.05350200
C	0.37254700	1.05113400	-2.43302600
C	0.98451700	2.29000600	-0.45384300
C	0.27644300	2.24573100	-3.15027700
H	0.21001400	0.10788800	-2.94685800
C	0.88106000	3.49055200	-1.16604700
C	0.49394800	3.46570900	-2.50438200
H	0.03461700	2.22007900	-4.20940100
H	1.16526700	4.42594800	-0.69794100
H	0.42073300	4.39839800	-3.05639600
C	1.93865200	-0.02158800	0.04055100
C	2.06066300	-1.25144600	-0.28606200
Si	3.05237700	-2.84157400	-0.47994600
C	4.89320700	-2.28741100	-0.53702000
C	2.53177400	-3.72208600	-2.10634000
C	2.65006500	-3.96305100	1.02471100
H	5.45325700	-3.21548600	-0.73529400
C	5.19451200	-1.31291600	-1.69307000
C	5.40654200	-1.70917200	0.79688200
H	1.66732900	-4.33077600	-1.80875700
C	2.04387100	-2.77876200	-3.22226500
C	3.62286400	-4.67196400	-2.64310100
H	1.69466000	-4.42703800	0.74619600
C	2.40985600	-3.20741900	2.34558300
C	3.69042000	-5.08664800	1.21425100
H	6.26391400	-1.06160400	-1.71648700
H	4.93480300	-1.73056800	-2.67189700
H	4.64220700	-0.37218900	-1.57770700
H	6.45813300	-1.40374000	0.70109100
H	4.83399400	-0.82935100	1.11201900
H	5.35340300	-2.44040300	1.61017300
H	2.82464400	-2.08148700	-3.55058400
H	1.73372800	-3.35809300	-4.10305500
H	1.18073500	-2.19247300	-2.89205900
H	3.24596100	-5.23648800	-3.50675700
H	4.51152500	-4.12374700	-2.97975400
H	3.95152500	-5.40426500	-1.89675000
H	1.57747400	-2.50296800	2.25376100
H	2.15570700	-3.91778100	3.14461500
H	3.28966700	-2.64353400	2.67466700
H	4.67906800	-4.69308300	1.48057700
H	3.38032500	-5.75587400	2.02833900
H	3.81046400	-5.70507400	0.31719300
Cl	-0.68625900	-3.17296400	-0.27199600
N	1.60981900	2.20237500	0.80556500
C	1.77180100	3.32493200	1.73598000
H	0.81502700	3.85408800	1.78499600
H	1.95580300	2.86507000	2.71165400
C	2.89099100	4.30230300	1.40939200
C	4.21209700	3.85992800	1.24996500
C	2.61947100	5.67223900	1.31361600
C	5.23273200	4.77226500	0.98343000
H	4.43875700	2.80286700	1.35051900
C	3.64246200	6.58731300	1.05311800
H	1.59957700	6.02844300	1.44675100
C	4.95194100	6.13774400	0.88301900
H	6.25226500	4.41648200	0.86102400
H	3.41384600	7.64731800	0.98110800
H	5.75020800	6.84568200	0.67718100
C	2.25856200	1.01050500	1.05526700
O	2.97301000	0.80166500	2.02811300
P	-2.39855700	-0.33321400	0.22116900
C	-3.72757800	-0.62670000	-1.11304600
C	-2.99405900	1.45241400	0.55878800
C	-2.84897300	-1.27330200	1.73970300
C	-3.42917100	0.39338500	-2.22817100
C	-3.76107400	-2.05643900	-1.63596600
O	-5.04706300	-0.35830400	-0.59308700
C	-4.48040500	1.45362600	0.94860400
C	-2.13730600	2.14730600	1.60688600
O	-2.83366800	2.18614500	-0.66749800
C	-4.11386700	-1.83557500	1.98244300
C	-1.84622700	-1.42034200	2.71319800
H	-4.09625100	0.18104900	-3.07194700
H	-2.39491100	0.30696200	-2.57266800
C	-3.71153600	1.82317800	-1.73996800
H	-4.54342400	-2.13173800	-2.40019600
H	-2.79664000	-2.33495300	-2.06658200
H	-3.98085900	-2.76852400	-0.83708500
C	-5.33910600	0.99749600	-0.23798500
H	-4.65816500	0.81982000	1.82118000
H	-4.76813500	2.48076700	1.20406500
H	-1.08388400	2.13556800	1.31885100
H	-2.46320900	3.18898300	1.70992100
H	-2.24254900	1.64983400	2.57648900
C	-4.36208200	-2.52373000	3.17179400
H	-4.90190600	-1.73355200	1.24593700
C	-2.10383100	-2.09388300	3.90665200
H	-0.85144100	-1.02646500	2.52520400
C	-3.53633100	2.88183400	-2.81201200
O	-5.07472000	1.87621900	-1.32519800
C	-6.83202700	1.05256200	0.02942000
C	-3.36281100	-2.65090500	4.13760600
H	-5.34225100	-2.96200600	3.34062500
H	-1.31443700	-2.19763800	4.64589200
H	-2.49857500	2.90094700	-3.15266800
H	-4.19680000	2.66990400	-3.65754200
H	-3.79466100	3.85964400	-2.39622100
H	-7.13032700	2.07590900	0.27343400
H	-7.37149900	0.72757400	-0.86421800
H	-7.09207400	0.39358000	0.86281600
H	-3.56150000	-3.18679100	5.06189200

Zero-point correction = 0.859114 (Hartree/Particle)
Thermal correction to Energy = 0.912671
Thermal correction to Enthalpy = 0.913616
Thermal correction to Gibbs Free Energy = 0.770285
Sum of electronic and zero-point Energies = -3166.865514
Sum of electronic and thermal Energies = -3166.811957
Sum of electronic and thermal Enthalpies = -3166.811013
Sum of electronic and thermal Free Energies = -3166.954343
E(RM06L) = -3169.53419297

f) reaction of aryl chloride **4a** (R = TIPS), L = P*t*Bu_3

PC_4a

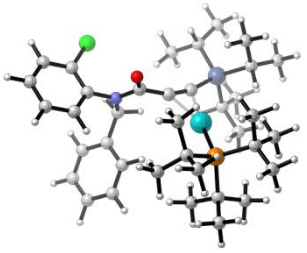

C	5.03464200	-0.54694500	-1.55997900
C	6.05603700	-1.31210900	-2.12305500
C	3.90753600	-1.15299000	-0.98523900
C	5.95413500	-2.70276500	-2.12100500
H	6.91484400	-0.81583000	-2.56217200
C	3.82581400	-2.54932900	-0.99338200
C	4.83764200	-3.32296600	-1.55812300
H	6.74873600	-3.29736400	-2.56274000
H	2.95372500	-3.01443700	-0.54630100
H	4.75474800	-4.40575100	-1.55628700
C	1.76260700	-0.07892000	-1.15266700
C	0.72313600	0.76009700	-0.53742900
C	0.11198000	1.79942400	-0.18703700
Si	-0.34525500	3.56116000	0.21688400
C	1.15047500	4.61268200	-0.37971800
H	1.33168200	4.25009300	-1.40311900
C	-1.95121500	3.92024800	-0.76642500
H	-2.59989100	3.06719500	-0.51560700
C	-0.59507800	3.60391400	2.12103100
H	0.28900600	3.08031300	2.51735500
C	0.89259800	6.12999100	-0.47424900
H	0.66298000	6.57287700	0.50196900
H	1.78322300	6.64511600	-0.86006400
H	0.06294000	6.36689700	-1.14868000
C	2.42719600	4.32580700	0.43456800
H	2.67787600	3.25939100	0.43948700
H	3.28665700	4.86226100	0.01001700
H	2.32666200	4.65141300	1.47769000
C	-1.70906700	3.87253900	-2.28834600
H	-2.65773500	3.96452000	-2.83488000
H	-1.06413200	4.69523000	-2.62203400
H	-1.23805600	2.93213700	-2.59588700
C	-2.70719600	5.20254600	-0.36695100
H	-2.11768400	6.10700400	-0.55661600
H	-3.63463300	5.29636500	-0.94876200
H	-2.98619000	5.20654400	0.69251600
C	-1.83373600	2.79757500	2.55961800
H	-1.81644600	1.77652900	2.15731500
H	-1.88356900	2.72800900	3.65514000
H	-2.76370900	3.27257100	2.22217100
C	-0.60932600	5.01193500	2.74898900
H	-1.44163400	5.62073800	2.37656800
H	-0.72344700	4.94363600	3.83967500
H	0.31673100	5.56284100	2.55259000
N	2.87140600	-0.37412800	-0.37866400
C	3.08641700	0.09200600	1.00149300
H	2.28343700	0.79803200	1.22338700
H	4.03105500	0.64647200	1.03883000
C	3.10041000	-1.02715600	2.02671800
C	4.27340300	-1.34968700	2.71658800
C	1.92992000	-1.74833500	2.30485000
C	4.28302600	-2.37219500	3.66905500
H	5.18656600	-0.79688500	2.50739700
C	1.93723500	-2.77032400	3.25367500
H	1.01189500	-1.50166900	1.77569700
C	3.11485800	-3.08527400	3.93878800
H	5.20289000	-2.61086300	4.19646800
H	1.02176000	-3.31719000	3.46433900
H	3.11921700	-3.88054100	4.67958000
O	1.64354700	-0.48893400	-2.30162700
Cl	5.17838800	1.20337100	-1.59109700
Pd	-1.27092200	0.08124900	-0.16363900
P	-2.88394200	-1.67790400	-0.26263000
C	-3.33211100	-2.38352700	1.49980800
C	-4.51816200	-1.00064900	-1.07869800
C	-2.20312700	-3.13795700	-1.35951400
C	-4.06252300	-3.74134700	1.52611600
C	-2.01849800	-2.50012100	2.30983600
C	-4.19193500	-1.35134700	2.26291200
C	-5.77749500	-1.87485500	-0.91355000
C	-4.79303100	0.40256000	-0.48476400
C	-4.27778500	-0.76954800	-2.58723700
C	-1.46704900	-2.53187300	-2.57982100
C	-1.12730100	-3.91210900	-0.56587700
C	-3.25955600	-4.14436200	-1.85961900
H	-4.30305500	-3.99888200	2.56695400
H	-5.00302200	-3.72589500	0.96942400
H	-3.44767100	-4.55390900	1.13091600
H	-1.49555600	-1.53898600	2.34187600
H	-2.26372200	-2.79463900	3.33960300
H	-1.32999800	-3.24604900	1.91132500
H	-5.21013300	-1.27014000	1.87611700
H	-4.27097200	-1.67048600	3.31057800
H	-3.73283600	-0.35711100	2.25732700
H	-6.09110200	-1.96810400	0.12926800
H	-5.64740100	-2.88104700	-1.32007200
H	-6.61013600	-1.40784100	-1.45753500
H	-5.64387400	0.85117600	-1.01557900
H	-5.04226700	0.38697600	0.57640200
H	-3.92562800	1.05828300	-0.62074800
H	-5.12870500	-0.20545000	-2.99174700
H	-3.37269300	-0.18085700	-2.76744500
H	-4.21052400	-1.69918100	-3.15649400
H	-2.12957800	-2.00183700	-3.26402200
H	-1.00592400	-3.35295700	-3.14584800
H	-0.67279100	-1.84187100	-2.28025300
H	-0.36661800	-3.24030900	-0.15534400
H	-1.54158600	-4.51472500	0.24614800
H	-0.62121600	-4.60240100	-1.25326900
H	-3.97533500	-3.68948100	-2.54906700
H	-3.82059600	-4.61298300	-1.04711200
H	-2.75149200	-4.94680900	-2.41183800

Zero-point correction= 0.885741 (Hartree/Particle)
Thermal correction to Energy= 0.940046
Thermal correction to Enthalpy= 0.940990
Thermal correction to Gibbs Free Energy= 0.792388
Sum of electronic and zero-point Energies= -2792.349041
Sum of electronic and thermal Energies= -2792.294736
Sum of electronic and thermal Enthalpies= -2792.293791
Sum of electronic and thermal Free Energies= -2792.442394
E(RM06L) = -2794.93264189

TS_OA_4a

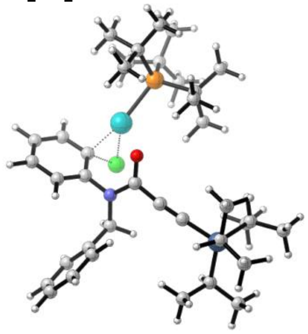

Pd	-2.30769500	0.82053500	0.26421900
C	-1.43245300	2.63437000	0.49434200
C	-2.46929000	3.57724500	0.36634700
C	-0.20334600	2.83301200	-0.18064400
C	-2.32104300	4.65372100	-0.51425600
H	-3.36161900	3.47324300	0.97489700
C	-0.08490600	3.91033500	-1.05812000
C	-1.13368200	4.82239400	-1.22553300
H	-3.13228900	5.36911600	-0.62316500
H	0.84112200	4.03034000	-1.61117600
H	-1.01106100	5.66461400	-1.89986700
P	-3.69205000	-1.13961500	-0.14097800
C	-4.59567200	-0.95239400	-1.85394700
C	-2.62331500	-2.76695400	-0.17946800
C	-5.03738600	-1.31412000	1.25629400
C	0.85842400	0.67966400	-0.47430500

C	-5.57536300	0.10153000	1.57873900
H	-6.25033100	0.03315800	2.44282300
H	-4.75720200	0.78079500	1.84115500
H	-6.13912100	0.54932000	0.75997800
C	-4.35973700	-1.80034200	2.55717900
H	-4.04713500	-2.84588800	2.51260400
H	-3.49366300	-1.18352100	2.81809200
H	-5.08383100	-1.71580800	3.37833700
C	-6.22535400	-2.24569000	0.94233100
H	-6.88084000	-2.30037000	1.82257900
H	-6.83618700	-1.87986500	0.11284800
H	-5.91030800	-3.26540400	0.70693600
C	-1.57710300	-2.66690700	0.95772500
H	-2.01446900	-2.65686100	1.95614000
H	-0.91280900	-3.54022800	0.89878300
H	-0.96361700	-1.76841200	0.84451900
C	-1.80025000	-2.81882300	-1.48705600
H	-1.21834900	-1.90510300	-1.63776700
H	-1.08916800	-3.65249100	-1.40764500
H	-2.41267600	-3.00918800	-2.37146800
C	-3.40478900	-4.08878900	-0.03893100
H	-2.70145400	-4.92876700	-0.12370400
H	-3.89988300	-4.18477700	0.93081900
H	-4.15953900	-4.21712200	-0.81953300
C	-3.57211900	-0.36873100	-2.85868300
H	-2.73985100	-1.03860900	-3.07245000
H	-4.08686500	-0.16501900	-3.80780900
H	-3.15182200	0.57377300	-2.49123900
C	-5.71111200	0.11006800	-1.73610300
H	-6.07487700	0.34550900	-2.74504400
H	-6.57176600	-0.23339500	-1.15745700
H	-5.33984000	1.04139900	-1.29555500
C	-5.21101200	-2.23828900	-2.44186000
H	-5.72877500	-1.99218000	-3.37935300
H	-4.45672300	-2.99145500	-2.68244100
H	-5.94602500	-2.69561500	-1.77424300
C	2.01857900	-0.16905700	-0.25716300
C	2.93769900	-0.96453800	-0.14255000
Si	4.30674200	-2.21253100	0.02089500
C	5.67358800	-1.34028100	1.04407900
H	5.13419200	-0.92609600	1.90996300
C	3.49663800	-3.69609200	0.92642900
H	2.56857500	-3.87466700	0.36217500
C	4.82469900	-2.59652400	-1.78530600
H	4.97496300	-1.60328400	-2.23586000
C	6.77403200	-2.26832300	1.59632400
H	7.34315400	-2.75578900	0.79616300
H	7.49170400	-1.69382700	2.19764000
H	6.36718200	-3.05560000	2.24009200
C	6.29066300	-0.15158300	0.27981300
H	5.53038300	0.56239300	-0.05687200
H	6.99502600	0.39648300	0.91991600
H	6.84981800	-0.48544300	-0.60332200
C	3.08997000	-3.32887600	2.36790500
H	2.52769000	-4.14992300	2.83258300
H	3.96672700	-3.13995100	3.00002600
H	2.45626200	-2.43543200	2.39995700
C	4.30748400	-5.00696200	0.89937500
H	5.27124800	-4.90771400	1.41218400
H	3.75387200	-5.80833800	1.40745900
H	4.50942400	-5.34810900	-0.12179000
C	3.68829300	-3.28491500	-2.56799600
H	2.75322900	-2.71603800	-2.51798300
H	3.95722600	-3.39093200	-3.62762800
H	3.48710000	-4.29280700	-2.18379800
C	6.14863400	-3.37118800	-1.93950600
H	6.09963800	-4.36701700	-1.48390000
H	6.38493800	-3.51503100	-3.00265800
H	6.99396300	-2.84145200	-1.48711300
Cl	-1.26893700	1.81540500	2.44934600
N	0.91080000	1.95267500	0.02906300
C	1.97687700	2.42185300	0.93336000
H	2.52898800	1.53737500	1.26305400
H	1.49098200	2.84641600	1.81697600
C	2.92933200	3.43069900	0.31668300
C	3.13491400	4.67157200	0.92851600
C	3.64266100	3.12701300	-0.85253000
C	4.03750500	5.59254800	0.39023300
H	2.58260500	4.92010900	1.83184500
C	4.54005700	4.04651800	-1.39507900
H	3.48893500	2.16756700	-1.34006000
C	4.74133200	5.28263900	-0.77356900
H	4.18372000	6.55287300	0.87759900
H	5.08494100	3.79808300	-2.30213700
H	5.44114700	5.99846600	-1.19642600
O	-0.11133800	0.23701600	-1.10267000

Zero-point correction = 0.884608 (Hartree/Particle)
Thermal correction to Energy = 0.938543
Thermal correction to Enthalpy = 0.939487
Thermal correction to Gibbs Free Energy = 0.792451
Sum of electronic and zero-point Energies = -2792.320179
Sum of electronic and thermal Energies = -2792.266244
Sum of electronic and thermal Enthalpies = -2792.265299
Sum of electronic and thermal Free Energies = -2792.412336
E(RM06L) = -2794.89719568

IVa

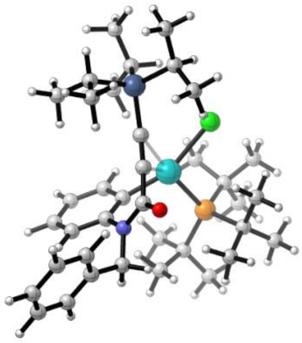

Pd	0.78060400	-0.53898300	0.05949300
C	0.05195600	0.83384700	1.36122800
C	0.31613900	0.58751600	2.71374300
C	-0.88513800	1.83733900	1.03794800
C	-0.26596300	1.35729100	3.72684700
H	1.00168100	-0.20960100	2.98878400
C	-1.47544800	2.60417900	2.05493200
C	-1.15279000	2.37925200	3.39222900
H	-0.02234100	1.15560900	4.76680300
H	-2.20765700	3.36059300	1.79387000
H	-1.61595200	2.98467200	4.16630100
P	3.07221000	0.41780200	-0.19000600
C	4.38693000	-0.92234100	0.38277900
C	3.29793200	0.77409300	-2.09883700
C	3.49675200	2.08079900	0.77229400
C	-1.36495800	-0.28368300	-0.63039800
C	-1.54194100	-1.44903300	-0.25216200
C	2.66062000	-0.36925900	-2.91837600
H	1.58608000	-0.44761100	-2.74020200
H	2.81264300	-0.14272400	-3.98226800
H	3.09311200	-1.34758800	-2.71689800
C	2.53417800	2.05837100	-2.49033800
H	2.50194400	2.11343800	-3.58566400
H	1.49814200	2.04674900	-2.13755200
H	3.02139700	2.97060400	-2.13868900
C	4.76972900	0.92844900	-2.54339700
H	5.34400400	0.00913900	-2.41667100
H	4.77709800	1.16468700	-3.61568900
H	5.29631300	1.73471800	-2.03136000
C	2.34972100	3.11027000	0.65056500
H	2.67023700	4.02791800	1.16200400
H	2.11099500	3.37983600	-0.37723400
H	1.44193200	2.76138900	1.13598400
C	3.63951500	1.79346400	2.28500900
H	2.74868700	1.31340200	2.69340300
H	4.51158700	1.18931400	2.53667100
H	3.75435900	2.75480200	2.80271500
C	4.78159200	2.78696800	0.27924000
H	5.66230000	2.14583900	0.25631900
H	4.65094300	3.22982400	-0.71104200
H	4.99789000	3.61328800	0.96936500

C	5.80744000	-0.37803300	0.65240000
H	5.86223700	0.34404100	1.46772400
H	6.44069900	-1.22832000	0.93704800
H	6.25603800	0.07001300	-0.23853400
C	3.82261100	-1.56811600	1.67034100
H	3.66405100	-0.85243300	2.48052600
H	2.87825900	-2.08055600	1.46374700
H	4.53903000	-2.31853300	2.02993900
C	4.54640100	-2.05907200	-0.65219900
H	5.05726300	-1.73484300	-1.56169000
H	5.17650600	-2.83263300	-0.19286500
H	3.59489800	-2.52003200	-0.91432000
Si	-2.23191200	-3.16933800	0.05879900
C	-4.05497800	-2.82031600	0.56535100
C	-1.24442000	-4.06289100	1.43762400
C	-2.11777900	-4.16920000	-1.57686700
H	-4.50060400	-3.81999200	0.69217600
C	-4.19065400	-2.07516500	1.90742600
C	-4.85779100	-2.08961200	-0.52916200
H	-0.36500300	-4.45314300	0.90782300
C	-0.71721500	-3.15359700	2.56145500
C	-2.01725100	-5.26212700	2.02596900
H	-1.12583400	-4.63841000	-1.52181400
C	-2.13276700	-3.31184800	-2.85694200
C	-3.17237200	-5.29329900	-1.66173500
H	-5.24950900	-1.91132300	2.15125200
H	-3.74580200	-2.63033600	2.73971000
H	-3.70913700	-1.09012000	1.86998900
H	-5.90333400	-1.95998700	-0.21724800
H	-4.45043500	-1.09020800	-0.72460000
H	-4.86308900	-2.63388800	-1.47909400
H	-1.52473700	-2.68653800	3.13711200
H	-0.10723200	-3.73557400	3.26667500
H	-0.08800100	-2.35277900	2.16119800
H	-1.38966900	-5.80293900	2.74723900
H	-2.92148400	-4.94487600	2.56036300
H	-2.32187700	-5.98345000	1.25930700
H	-1.30730100	-2.59516100	-2.86490000
H	-2.02741700	-3.95494400	-3.74157000
H	-3.06904300	-2.75251200	-2.97230800
H	-4.19123600	-4.89331000	-1.73331500
H	-3.00313200	-5.90371700	-2.55899400
H	-3.14437600	-5.97050400	-0.80011900
Cl	1.21955900	-2.75446400	-0.98074900
N	-1.25287000	2.09075600	-0.31928100
C	-1.36063500	3.46871500	-0.83699300
H	-0.60930700	4.06716100	-0.31456100
H	-1.08235400	3.41145200	-1.89355200
C	-2.72597100	4.12672600	-0.70947500
C	-3.85726200	3.57190800	-1.32705000
C	-2.86258700	5.33041100	-0.00688400
C	-5.09646900	4.20378300	-1.22573800
H	-3.75623400	2.65388200	-1.89744000
C	-4.10260700	5.96694300	0.08956100
H	-1.99085500	5.77539000	0.46895900
C	-5.22430900	5.40178800	-0.51715000
H	-5.96460900	3.76282700	-1.70859600
H	-4.18950200	6.90052000	0.63926900
H	-6.19127000	5.89213100	-0.44271100
C	-1.48645200	1.06096800	-1.19959600
O	-1.79264400	1.22499100	-2.37808700

Zero-point correction = 0.887531 (Hartree/Particle)
Thermal correction to Energy = 0.941313
Thermal correction to Enthalpy = 0.942257
Thermal correction to Gibbs Free Energy = 0.799540
Sum of electronic and zero-point Energies = -2792.347955
Sum of electronic and thermal Energies = -2792.294173
Sum of electronic and thermal Enthalpies = -2792.293229
Sum of electronic and thermal Free Energies = -2792.435946
E(RM06L) = -2794.95009611

TS_AI_IVa

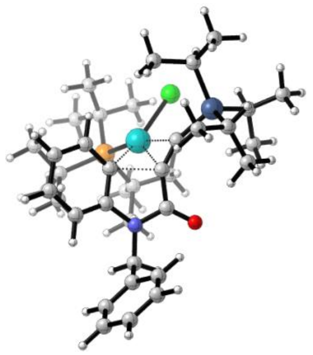

Pd	-0.93125100	0.45374900	0.25129700
C	0.51229200	-0.83759000	1.20020400
C	0.21533800	-0.72095100	2.56896700
C	1.39136900	-1.86538200	0.79725700
C	0.62776500	-1.70155200	3.47481200
H	-0.37816300	0.11498800	2.92726700
C	1.82253500	-2.83941100	1.70550500
C	1.40766400	-2.77424800	3.03476200
H	0.34022900	-1.62349300	4.51960500
H	2.51699100	-3.60840400	1.38697400
H	1.73799200	-3.53520100	3.73606400
P	-2.95519700	-1.12327500	-0.20101400
C	-4.68962600	-0.28220000	0.18110000
C	-2.83551500	-1.44542100	-2.11915200
C	-2.98819000	-2.86287400	0.70364600
C	1.23532400	0.48275400	-0.12570100
C	0.86661700	1.69301900	0.05144400
C	-2.47034900	-0.10995800	-2.80932100
H	-1.51554300	0.28037200	-2.44542500
H	-2.37216100	-0.29373300	-3.88780800
H	-3.21647700	0.67120400	-2.66926200
C	-1.66973700	-2.41521600	-2.41368700
H	-1.48409000	-2.41342800	-3.49558600
H	-0.74182900	-2.10465500	-1.92255400
H	-1.89289900	-3.44625200	-2.12795300
C	-4.10797900	-2.01514300	-2.78240800
H	-4.95527100	-1.32943500	-2.72694300
H	-3.89933900	-2.18069700	-3.84812400
H	-4.41654500	-2.97337200	-2.36026200
C	-1.57018800	-3.46936400	0.75705700
H	-1.64684900	-4.47699800	1.18794300
H	-1.09358100	-3.56797800	-0.21747300
H	-0.91764500	-2.88763300	1.40165800
C	-3.39136400	-2.66470400	2.18384800
H	-2.77458500	-1.90334200	2.67174500
H	-4.44084100	-2.40282600	2.32255700
H	-3.22156400	-3.61064900	2.71498300
C	-3.91078600	-3.92290800	0.06095400
H	-4.93690500	-3.58883900	-0.08840400
H	-3.52080600	-4.27092900	-0.89951400
H	-3.94422700	-4.79756300	0.72491600
C	-5.89587400	-1.24710600	0.22084800
H	-5.84443100	-1.98565500	1.02215300
H	-6.79770200	-0.64705200	0.40061300
H	-6.04500500	-1.77315500	-0.72615500
C	-4.56300800	0.42807100	1.55059100
H	-4.34119600	-0.25564200	2.37209600
H	-3.79526900	1.20430200	1.51899700
H	-5.52156800	0.91301200	1.77963600
C	-5.04274700	0.80771300	-0.85802800
H	-5.29837900	0.39267700	-1.83578600
H	-5.93597800	1.33245600	-0.49308400
H	-4.25221500	1.54808400	-0.96908000
Si	1.23638600	3.54482200	0.06545800
C	3.15585800	3.64400700	0.18103400
C	0.41254200	4.37474400	1.59065600
C	0.55567800	4.32000800	-1.55287200
H	3.36308600	4.72164400	0.28156800
C	3.72755300	2.94889300	1.43256200
C	3.88346800	3.14786200	-1.08434800
H	-0.58717200	4.65228300	1.23183500
C	0.20471300	3.44154900	2.79835200

C	1.13702300	5.66647600	2.02451000
H	-0.50497400	4.48808300	-1.32584300
C	0.60761500	3.39064200	-2.78025500
C	1.19757300	5.68772600	-1.86672600
H	4.81633600	3.08696600	1.48683400
H	3.30086600	3.34093800	2.36213600
H	3.53774600	1.86876400	1.41231500
H	4.97214600	3.23005100	-0.95576500
H	3.65513500	2.09959300	-1.30728800
H	3.61581800	3.73310800	-1.97009700
H	1.15194300	3.05304900	3.19288600
H	-0.29132900	3.98164900	3.61691400
H	-0.42974900	2.59129200	2.53060400
H	0.57254800	6.16964500	2.82131700
H	2.13886600	5.46033500	2.42084000
H	1.24928900	6.38521300	1.20453900
H	0.02806900	2.47835700	-2.60990400
H	0.17842200	3.89746100	-3.65586700
H	1.62944600	3.09376900	-3.04127200
H	2.26682500	5.60052300	-2.09466200
H	0.71698300	6.13736800	-2.74631600
H	1.09185000	6.40171100	-1.04155700
Cl	-2.18638600	2.51877800	-0.13045700
N	1.92569800	-1.74923300	-0.49975800
C	2.56141700	-2.84148000	-1.24386500
H	1.93186000	-3.72946200	-1.12849800
H	2.52492300	-2.53285600	-2.29306600
C	3.99562900	-3.16499200	-0.85334100
C	4.99075200	-2.17742000	-0.88383800
C	4.35035400	-4.47336200	-0.50425900
C	6.30809000	-2.49507700	-0.55412500
H	4.73187500	-1.16562000	-1.18081000
C	5.67106400	-4.79355900	-0.18008800
H	3.58850900	-5.25065000	-0.48862300
C	6.65259200	-3.80262700	-0.20028500
H	7.06980500	-1.72044400	-0.58067000
H	5.92910600	-5.81426300	0.08944200
H	7.68046200	-4.04661600	0.05411300
C	1.97798100	-0.46206500	-0.99091700
O	2.52765400	-0.13882800	-2.03662900

Zero-point correction = 0.886680 (Hartree/Particle)
Thermal correction to Energy = 0.939622
Thermal correction to Enthalpy = 0.940567
Thermal correction to Gibbs Free Energy = 0.800467
Sum of electronic and zero-point Energies = -2792.330394
Sum of electronic and thermal Energies = -2792.277451
Sum of electronic and thermal Enthalpies = -2792.276507
Sum of electronic and thermal Free Energies = -2792.416607
E(RM06L) = -2794.93276857

g) reaction of aryl chloride **4b** (R = Mes), L = P*t*Bu$_3$

4b

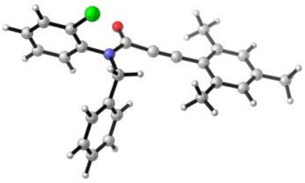

C	-3.70003100	-1.59428400	-0.68586300
C	-5.04134800	-1.87507300	-0.42432100
C	-2.91678300	-0.87901400	0.23235900
C	-5.61220100	-1.44941200	0.77425900
H	-5.62225200	-2.42954200	-1.15355300
C	-3.50949100	-0.46299300	1.42986800
C	-4.84521300	-0.74643500	1.70484500
H	-6.65552100	-1.67268500	0.97829700
H	-2.90106000	0.08666100	2.14003500
H	-5.28547300	-0.41666300	2.64113500
C	-0.56767600	-1.28660800	0.60103400
C	0.80723700	-0.90925700	0.31837000
C	2.00011800	-0.69998100	0.19427600
O	-0.82388300	-2.20234100	1.37369300
N	-1.55235800	-0.55611900	-0.04609800
C	-1.28100600	0.57542000	-0.94572700
H	-0.23238300	0.49740300	-1.24470800
H	-1.88322800	0.44615200	-1.85062600
C	-1.55723400	1.93679400	-0.32995000
C	-0.84236700	2.37250800	0.79578500
C	-2.51968100	2.78528600	-0.88675800
C	-1.08706600	3.62904100	1.34881900
H	-0.09081100	1.72288200	1.23780900
C	-2.76500800	4.04625400	-0.33681500
H	-3.08219200	2.45703500	-1.75808000
C	-2.04972800	4.47040400	0.78311300
H	-0.52429700	3.95413400	2.22001700
H	-3.51711600	4.69251200	-0.78165400
H	-2.23950100	5.44978800	1.21414600
Cl	-2.99912200	-2.16864900	-2.19112900
C	3.39914600	-0.46973300	0.05970200
C	3.87313900	0.59272300	-0.74995100
C	4.30947800	-1.31030100	0.74929900
C	5.24952000	0.79225100	-0.85224700
C	5.67632700	-1.06778900	0.61127600
C	6.16826700	-0.02682900	-0.18468000
H	5.61675500	1.60986500	-1.46910300
H	6.37814000	-1.70819200	1.14134100
C	3.80906900	-2.43989300	1.61521300
H	3.15705500	-2.07194300	2.41603000
H	3.21247800	-3.15360100	1.03512000
H	4.64261500	-2.98135700	2.07236100
C	2.90990400	1.48932000	-1.48836800
H	2.32035800	0.92191900	-2.21964900
H	2.19583900	1.96496800	-0.80564400
H	3.44281000	2.27879500	-2.02662400
C	7.65426700	0.19270100	-0.34043900
H	8.04513400	-0.35531600	-1.20852700
H	7.88971800	1.25140200	-0.49329700
H	8.20574400	-0.15622800	0.53891900

Zero-point correction = 0.401615 (Hartree/Particle)
Thermal correction to Energy = 0.428333
Thermal correction to Enthalpy = 0.429278
Thermal correction to Gibbs Free Energy = 0.339081
Sum of electronic and zero-point Energies = -1555.624886
Sum of electronic and thermal Energies = -1555.598168
Sum of electronic and thermal Enthalpies = -1555.597224
Sum of electronic and thermal Free Energies = -1555.687421
E(RM06L) = -1556.31315830

PC_4b

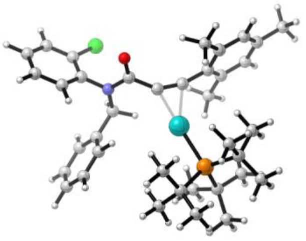

C	5.42198300	1.23146500	-0.12639400
C	6.75079700	1.18967100	-0.54871600
C	4.47506500	0.31913900	-0.61611600
C	7.14612400	0.23171700	-1.48144600
H	7.45956900	1.90893400	-0.15249700
C	4.89320500	-0.63028500	-1.55453800
C	6.21624200	-0.67701400	-1.98860600
H	8.18056000	0.20224600	-1.81186500
H	4.15889400	-1.33133200	-1.93600600
H	6.51914000	-1.42149700	-2.71889100
C	2.19977200	1.03749000	-0.92957300

C	0.82542400	1.13879700	-0.41341500
C	-0.11853500	1.89985300	-0.08100900
N	3.11723300	0.33594500	-0.16458300
C	2.81591100	-0.27191500	1.13832700
H	1.76886700	-0.04078300	1.35641700
H	3.42472900	0.21188800	1.91123600
C	3.03598300	-1.77362000	1.17469300
C	3.86693100	-2.34568500	2.14294200
C	2.39263700	-2.61475600	0.25517800
C	4.05185600	-3.72985900	2.19966800
H	4.37663400	-1.70289200	2.85733400
C	2.57955400	-3.99605000	0.30505700
H	1.74594300	-2.17810200	-0.50238800
C	3.40999400	-4.55840300	1.27939900
H	4.70300200	-4.15696800	2.95790200
H	2.07625300	-4.63531800	-0.41576700
H	3.55598600	-5.63467700	1.31754100
O	2.50564500	1.55907000	-1.99506600
Pd	-0.85306100	-0.15958800	-0.25670000
P	-2.79476200	-1.55867900	-0.18539800
C	-3.37948900	-1.92754200	1.63590900
C	-4.26580900	-0.71776500	-1.14851400
C	-2.38009900	-3.25510900	-1.05132900
C	-4.35035800	-3.11270400	1.81168900
C	-2.11660300	-2.17203300	2.49736300
C	-4.04486500	-0.66283600	2.22281900
C	-5.66064900	-1.33790100	-0.92932900
C	-4.31105500	0.77713600	-0.74899900
C	-3.95173100	-0.73235800	-2.66108000
C	-1.50519400	-2.94946100	-2.29233200
C	-1.49417100	-4.11026200	-0.11788400
C	-3.58812900	-4.10922300	-1.48604300
H	-4.64146400	-3.18268800	2.86888200
H	-5.26767300	-2.99597400	1.22894400
H	-3.89723200	-4.07020700	1.54285500
H	-1.41037900	-1.34089300	2.40010000
H	-2.41764800	-2.24887000	3.55105100
H	-1.58799600	-3.09086300	2.24260200
H	-5.02959100	-0.46046800	1.79565800
H	-4.18684300	-0.81298700	3.30116500
H	-3.41752000	0.22467100	2.09265300
H	-6.00696200	-1.23365800	0.10218500
H	-5.70126600	-2.39704200	-1.19590200
H	-6.38740500	-0.81305300	-1.56454500
H	-5.05676500	1.28397200	-1.37652500
H	-4.59684100	0.94076600	0.29009800
H	-3.34278700	1.25963900	-0.91129400
H	-4.68107000	-0.09139300	-3.17352200
H	-2.95453700	-0.33090600	-2.86941900
H	-4.03151200	-1.72651200	-3.10684500
H	-2.03517300	-2.40507400	-3.07381300
H	-1.16349600	-3.89927400	-2.72637300
H	-0.62225600	-2.36029000	-2.01959100
H	-0.62333100	-3.55654400	0.24689800
H	-2.03756300	-4.51019000	0.74088500
H	-1.12146600	-4.97113600	-0.68909300
H	-4.18995300	-3.62236500	-2.25720600
H	-4.24640700	-4.36457100	-0.65152800
H	-3.22229100	-5.05284200	-1.91399700
C	-0.92534500	3.05926800	0.16870200
C	-1.32481300	3.39756800	1.48457700
C	-1.31092000	3.86988900	-0.93096900
C	-2.10956500	4.53568500	1.67460100
C	-2.09455700	4.99741500	-0.68447400
C	-2.50454300	5.35016000	0.60708000
H	-2.41787800	4.79735000	2.68494800
H	-2.39171000	5.62144500	-1.52511800
C	-0.89715100	2.55440400	2.65958600
H	-1.15495900	1.49949100	2.50582900
H	0.19060400	2.59255500	2.80080500
H	-1.36906200	2.89978600	3.58479800
C	-0.88970300	3.51313000	-2.33533800
H	0.19345900	3.36832800	-2.41006700
H	-1.34670200	2.56694800	-2.65417600
H	-1.18978000	4.29261200	-3.04253600
C	-3.32462100	6.59630000	0.84436000
H	-3.92038000	6.51673900	1.75992300
H	-2.68103300	7.48034700	0.95040900
H	-4.00703600	6.79274700	0.01011000
Cl	4.94690100	2.47000700	1.02614200

Zero-point correction= 0.774594 (Hartree/Particle)
Thermal correction to Energy= 0.823035
Thermal correction to Enthalpy= 0.823979
Thermal correction to Gibbs Free Energy= 0.686077
Sum of electronic and zero-point Energies= -2496.926158
Sum of electronic and thermal Energies= -2496.877717
Sum of electronic and thermal Enthalpies= -2496.876773
Sum of electronic and thermal Free Energies= -2497.014676
E(RM06L) = -2499.38719323

TS_OA_4b

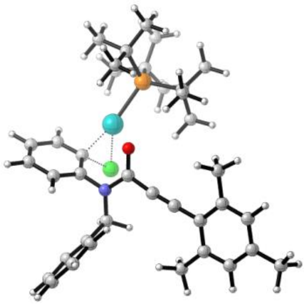

Pd	1.99427100	0.79922300	-0.26137800
C	1.00881100	2.56224300	-0.42541800
C	1.98392600	3.56334000	-0.25961300
C	-0.23012200	2.65559000	0.25415000
C	1.76753800	4.59443100	0.66032100
H	2.88095800	3.53926600	-0.86963000
C	-0.41646000	3.68997000	1.17085700
C	0.57177400	4.66045200	1.37450200
H	2.53161100	5.35543500	0.79779600
H	-1.34811900	3.72934100	1.72622800
H	0.39576300	5.46737500	2.07953300
P	3.50048900	-1.08461700	0.05838400
C	4.31747900	-0.98169000	1.82138900
C	2.54883800	-2.77732800	-0.08526600
C	4.91054900	-1.04935700	-1.28414300
C	-1.15627300	0.42741600	0.46591800
C	5.35552500	0.42238400	-1.46683000
H	6.06985300	0.47363800	-2.29991100
H	4.50069000	1.06128300	-1.71342300
H	5.84861000	0.83980000	-0.58858100
C	4.32456300	-1.47240900	-2.65007100
H	4.08922900	-2.53764200	-2.70375900
H	3.42634600	-0.89923700	-2.90151700
H	5.07345400	-1.26904900	-3.42700400
C	6.15030800	-1.91846300	-0.99157200
H	6.84492800	-1.84973300	-1.84027200
H	6.69626400	-1.58435200	-0.10543400
H	5.90270200	-2.97475100	-0.85860400
C	1.54791200	-2.65346000	-1.26013900
H	2.02641900	-2.53892900	-2.23284800
H	0.93983800	-3.56786200	-1.29948300
H	0.87375300	-1.80478100	-1.11088600
C	1.67707200	-2.98920200	1.17369200
H	1.03152400	-2.12839600	1.37147000
H	1.02713700	-3.85694800	0.99414600

H	2.26345400	-3.21445700	2.06763700
C	3.42586800	-4.02853700	-0.29167200
H	2.78053800	-4.91787100	-0.30531900
H	3.96560200	-4.01256700	-1.24203300
H	4.15523100	-4.16840600	0.51069200
C	3.21448700	-0.55749500	2.82210800
H	2.43050500	-1.30341900	2.94903300
H	3.67639700	-0.39075900	3.80504500
H	2.73404700	0.37564900	2.50914700
C	5.35610500	0.16180400	1.84151200
H	5.66341400	0.33425900	2.88148100
H	6.26069900	-0.06950300	1.27423200
H	4.93412400	1.09930500	1.46427100
C	5.00022700	-2.26720600	2.33020900
H	5.45719300	-2.06755500	3.30944600
H	4.29342800	-3.08889700	2.46953100
H	5.79524500	-2.61292500	1.66409500
C	-2.26919300	-0.46957800	0.23624400
C	-3.14617000	-1.30846600	0.13659100
Cl	0.89668700	1.81310600	-2.41187400
N	-1.28606900	1.71578800	0.01043100
C	-2.37775700	2.15063800	-0.87854400
H	-2.86456800	1.24605300	-1.25354000
H	-1.91918200	2.64775900	-1.73870800
C	-3.40327100	3.06213600	-0.22738500
C	-3.68894300	4.31291900	-0.78486800
C	-4.10406800	2.65713600	0.91869900
C	-4.65721000	5.14445300	-0.21611700
H	-3.14720100	4.63984500	-1.66945300
C	-5.06758300	3.48729300	1.49132800
H	-3.88854800	1.68916600	1.36399700
C	-5.34821900	4.73395300	0.92407700
H	-4.86480800	6.11400400	-0.66115900
H	-5.60173700	3.16074200	2.37985300
H	-6.09925800	5.38012700	1.37062300
O	-0.15188300	0.02255200	1.06773500
C	-4.16646200	-2.29515600	0.02438800
C	-5.34154800	-2.02220300	-0.72021200
C	-3.99759200	-3.54693900	0.66940800
C	-6.32253600	-3.01000400	-0.80478700
C	-5.00983700	-4.49923900	0.55298000
C	-6.17696200	-4.25458200	-0.18049600
H	-7.22699800	-2.80350600	-1.37304000
H	-4.88601800	-5.45938300	1.04943800
C	-5.52753600	-0.69373500	-1.41111500
H	-5.45611600	0.14262600	-0.70581200
H	-4.75647300	-0.53202100	-2.17490000
H	-6.50349100	-0.64103000	-1.90268200
C	-2.75299500	-3.83819900	1.47083100
H	-1.84971400	-3.73817600	0.85766900
H	-2.64046800	-3.13479800	2.30442100
H	-2.78001700	-4.85228400	1.88067600
C	-7.24155600	-5.31693600	-0.31643500
H	-7.04943800	-5.95850300	-1.18717000
H	-7.27443800	-5.96725900	0.56413400
H	-8.23443100	-4.87521900	-0.45195400

Zero-point correction = 0.773591 (Hartree/Particle)
Thermal correction to Energy = 0.821641
Thermal correction to Enthalpy = 0.822585
Thermal correction to Gibbs Free Energy = 0.687385
Sum of electronic and zero-point Energies = -2496.899351
Sum of electronic and thermal Energies = -2496.851302
Sum of electronic and thermal Enthalpies = -2496.850358
Sum of electronic and thermal Free Energies = -2496.985558
E(RM06L) = -2499.35705028

IVb

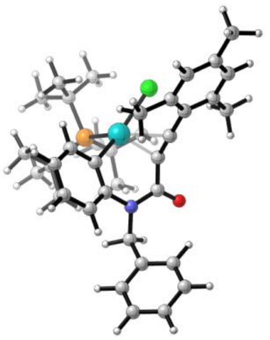

Pd	0.61913800	-0.69197300	0.08891800
C	-0.01417200	0.72441700	1.38422100
C	0.20422900	0.45609900	2.74200100
C	-0.88572900	1.78182100	1.04841600
C	-0.35943700	1.25091200	3.74670100
H	0.83828000	-0.38036800	3.02606300
C	-1.45692000	2.57514500	2.05562200
C	-1.17908200	2.32372000	3.39879100
H	-0.15296300	1.03054300	4.79107600
H	-2.13792700	3.37440100	1.78258400
H	-1.62452700	2.95023500	4.16661300
P	2.98993500	0.00789400	-0.11907400
C	4.14399900	-1.42548900	0.55610700
C	3.31172600	0.25176100	-2.03168500
C	3.53375600	1.66680600	0.78392700
C	-1.47237800	-0.31669600	-0.62602100
C	-2.05975400	-1.37464600	-0.36295100
C	2.58975200	-0.85858200	-2.82623000
H	1.50734000	-0.81621600	-2.68932800
H	2.80509500	-0.70053000	-3.89172500
H	2.90786600	-1.86549500	-2.56276100
C	2.69364700	1.58501600	-2.50762500
H	2.71437500	1.59513500	-3.60465200
H	1.64670100	1.68601900	-2.20468000
H	3.25064000	2.46171500	-2.16961800
C	4.80577400	0.23757300	-2.42642900
H	5.27550800	-0.73179300	-2.25021900
H	4.87410800	0.43428200	-3.50458600
H	5.39724500	1.00165200	-1.92042600
C	2.48017800	2.77901600	0.57696400
H	2.84633600	3.68457000	1.07942700
H	2.31364400	3.03389000	-0.46869500
H	1.52479000	2.51461300	1.02467000
C	3.60633900	1.43791900	2.31130700
H	2.66516200	1.05653200	2.71057900
H	4.41465300	0.77197100	2.61573800
H	3.79157800	2.40809400	2.79081200
C	4.89089900	2.23596500	0.30850400
H	5.71286600	1.52178400	0.36014700
H	4.83902900	2.62964500	-0.70936500
H	5.15080100	3.07972100	0.96161700
C	5.60589800	-1.01436600	0.83881900
H	5.71246100	-0.26222100	1.62163600
H	6.14462700	-1.90805100	1.17999500
H	6.11786000	-0.65525600	-0.05831800
C	3.48636300	-1.94526700	1.85643900
H	3.39428900	-1.18045100	2.63109000
H	2.49423700	-2.35771600	1.64844100
H	4.10712800	-2.75385300	2.26459500
C	4.20082300	-2.62363300	-0.41799500
H	4.76149500	-2.40248700	-1.32910000
H	4.73388000	-3.43632800	0.09375300
H	3.20904000	-2.99171700	-0.68161600
Cl	0.80666700	-2.97065000	-0.85343100
N	-1.20078700	2.05409800	-0.31645800
C	-1.19936500	3.43394000	-0.83738500
H	-0.43477700	3.98666800	-0.28444800
H	-0.88232200	3.35778100	-1.88172100
C	-2.52576800	4.17463500	-0.76501000
C	-3.65973100	3.69232900	-1.43639900
C	-2.62194300	5.38015600	-0.05909200
C	-4.86233500	4.39671800	-1.38509800

H	-3.58778300	2.77274000	-2.00887500
C	-3.82495800	6.08937200	-0.01215100
H	-1.74722500	5.76934200	0.45837200
C	-4.95011800	5.59623700	-0.67281200
H	-5.73280200	4.01158100	-1.90985500
H	-3.88051300	7.02335700	0.54100800
H	-5.88840400	6.14346600	-0.63744200
C	-1.45953300	1.03570900	-1.20575100
O	-1.67812500	1.22041200	-2.40027500
C	-2.72246200	-2.59274800	-0.11131000
C	-3.17280500	-2.90144600	1.20155800
C	-2.96120900	-3.48561400	-1.19400000
C	-3.84964700	-4.10064500	1.40454200
C	-3.64706000	-4.66896400	-0.93005300
C	-4.09173900	-4.99903700	0.35636200
H	-4.19851600	-4.34537600	2.40529400
H	-3.83795700	-5.35724400	-1.75019700
C	-2.91739300	-1.95693600	2.34775100
H	-1.84304200	-1.82911500	2.52428500
H	-3.31995100	-0.95751300	2.14573400
H	-3.37311400	-2.32996200	3.26979000
C	-2.48572700	-3.16104300	-2.58612200
H	-2.86631600	-2.19104600	-2.92631100
H	-1.39151100	-3.10368400	-2.60429800
H	-2.81088100	-3.92764100	-3.29581000
C	-4.79618600	-6.30853900	0.61359000
H	-5.48350700	-6.23587300	1.46297800
H	-5.36507000	-6.63743300	-0.26241100
H	-4.07172100	-7.10062900	0.84675100

Zero-point correction = 0.776305 (Hartree/Particle)
Thermal correction to Energy = 0.824216
Thermal correction to Enthalpy = 0.825160
Thermal correction to Gibbs Free Energy = 0.693509
Sum of electronic and zero-point Energies = -2496.928338
Sum of electronic and thermal Energies = -2496.880427
Sum of electronic and thermal Enthalpies = -2496.879483
Sum of electronic and thermal Free Energies = -2497.011133
E(RM06L) = -2499.40416403

TS_AI_IVb

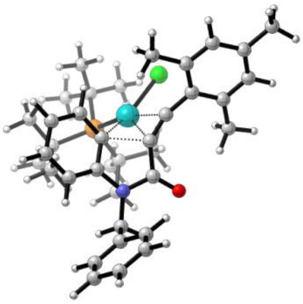

Pd	-0.89803500	0.38969300	0.26531400
C	0.81663800	-0.53293600	1.19754600
C	0.49402400	-0.51943800	2.56512600
C	1.92282200	-1.30715000	0.78472600
C	1.12933300	-1.38716000	3.45780700
H	-0.29238000	0.13452600	2.93084300
C	2.57492900	-2.16271200	1.67999700
C	2.15021200	-2.22692700	3.00663900
H	0.82472100	-1.40377200	4.50061200
H	3.43748100	-2.73297700	1.35456000
H	2.65296800	-2.89809100	3.69715300
P	-2.51262300	-1.59112700	-0.20095900
C	-4.37959700	-1.18364400	0.25364700
C	-2.38271300	-1.82479800	-2.13121000
C	-2.11105900	-3.31459000	0.64429500
C	1.17758600	0.93049100	-0.11264200
C	0.58380600	2.04353900	0.07231300
C	-2.35748400	-0.42306400	-2.78482400
H	-1.50600700	0.16586600	-2.43227000
H	-2.25650700	-0.55053500	-3.87128500
H	-3.25720600	0.16155500	-2.59766300
C	-1.03410400	-2.48896700	-2.48679000
H	-0.88657300	-2.41157200	-3.57180900
H	-0.18849600	-1.98687600	-2.00537900
H	-1.00457600	-3.55183500	-2.23405500
C	-3.51003800	-2.65601600	-2.78054400
H	-4.49012300	-2.18591800	-2.68182600
H	-3.30295700	-2.74221200	-3.85594700
H	-3.57650500	-3.66985700	-2.38124900
C	-0.58974100	-3.57516800	0.63823800
H	-0.41428700	-4.57839900	1.05012400
H	-0.13946400	-3.54797100	-0.35345500
H	-0.06649900	-2.86535100	1.27238300
C	-2.49798700	-3.25419200	2.14062800
H	-2.04908700	-2.38899200	2.63851000
H	-3.57413800	-3.23684300	2.31589500
H	-2.10457700	-4.15326600	2.63331100
C	-2.78468700	-4.54334400	-0.00707000
H	-3.86580400	-4.45578600	-0.11043000
H	-2.36321200	-4.76236400	-0.99195400
H	-2.58595500	-5.41997300	0.62467600
C	-5.32919200	-2.40168200	0.29440900
H	-5.08178300	-3.12889200	1.06930000
H	-6.33864700	-2.03167600	0.51756100
H	-5.38413600	-2.92344700	-0.66511600
C	-4.37207100	-0.49617000	1.64056400
H	-3.96631200	-1.12759700	2.43339200
H	-3.80813100	0.43897800	1.60825400
H	-5.40822700	-0.25463700	1.91368100
C	-5.00627600	-0.17682500	-0.73880900
H	-5.19057300	-0.61237400	-1.72371200
H	-5.98400800	0.11808600	-0.33440800
H	-4.40889700	0.72802500	-0.84206700
Cl	-2.58030400	2.12960600	-0.05771300
N	2.41071700	-1.04662700	-0.50894700
C	3.30672900	-1.93142500	-1.25911100
H	2.92710900	-2.95280600	-1.15452500
H	3.19442000	-1.63260000	-2.30580700
C	4.77510600	-1.87792200	-0.86466100
C	5.48039000	-0.66587100	-0.87698900
C	5.45598500	-3.05409200	-0.52935200
C	6.83419900	-0.63571800	-0.54334500
H	4.96898300	0.24831800	-1.16313500
C	6.81369300	-3.02548300	-0.20114000
H	4.92154700	-4.00237200	-0.52760400
C	7.50494800	-1.81391400	-0.20337800
H	7.36918300	0.31014800	-0.55582300
H	7.32669200	-3.94796200	0.05765800
H	8.56026100	-1.78657400	0.05425000
C	2.13241100	0.21704100	-0.98655400
O	2.57514000	0.67697300	-2.03268500
C	0.44548500	3.46965300	0.08415600
C	0.41314300	4.16110300	1.31860800
C	0.36732300	4.17301100	-1.14389300
C	0.34879700	5.55334200	1.30112100
C	0.29747000	5.56528300	-1.10210300
C	0.28764700	6.27518900	0.10371400
H	0.33789500	6.08923900	2.24794000
H	0.24761500	6.11192900	-2.04149800
C	0.42813100	3.40668900	2.62389200
H	-0.49566200	2.82414000	2.73452700
H	1.26615000	2.70141200	2.68200500
H	0.50088500	4.09227600	3.47372600
C	0.34191800	3.43646800	-2.45859800
H	1.17699600	2.73258300	-2.54954900
H	-0.58670200	2.85804600	-2.54578600
H	0.38505500	4.13740300	-3.29805700
C	0.17885200	7.78132200	0.11275800
H	-0.87245100	8.09961400	0.10941200
H	0.64616000	8.21273700	1.00444000
H	0.65395100	8.22345200	-0.76964000

Zero-point correction = 0.775326 (Hartree/Particle)
Thermal correction to Energy = 0.822475
Thermal correction to Enthalpy = 0.823419
Thermal correction to Gibbs Free Energy = 0.693740
Sum of electronic and zero-point Energies = -2496.906032
Sum of electronic and thermal Energies = -2496.858884
Sum of electronic and thermal Enthalpies = -2496.857940
Sum of electronic and thermal Free Energies = -2496.987618

E(RM06L) = -2499.38468144

h) reaction of aryl bromide **1a** (R = TIPS), L = P*t*Bu$_3$

1a

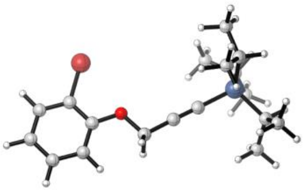

C	4.09923700	-0.06945900	0.00056900
C	5.46555000	0.18687200	0.00305700
C	3.16591200	0.98505300	-0.00195700
C	5.93372300	1.50361100	0.00308300
H	6.15817800	-0.64796500	0.00500300
C	3.64610100	2.30155700	-0.00203700
C	5.02042700	2.55425100	0.00050100
H	7.00202800	1.69698900	0.00509000
H	2.95441600	3.13595800	-0.00413500
H	5.36846500	3.58331400	0.00042500
O	1.85000300	0.64133400	-0.00414000
C	0.88261900	1.69419300	-0.00506700
H	1.01973700	2.33048300	0.88206200
H	1.01970900	2.32903300	-0.89325300
C	-0.45352900	1.10959500	-0.00431200
C	-1.58116000	0.65131500	-0.00332400
Br	3.48535200	-1.87359100	0.00053700
Si	-3.28141500	-0.07640600	-0.00003300
C	-3.55562800	-0.98279100	1.67646700
H	-4.64964700	-0.98357100	1.81351100
C	-4.55085200	1.35844900	-0.20579500
H	-5.43935000	0.87744100	-0.64636400
C	-3.41397300	-1.31600300	-1.46776800
H	-4.20411400	-2.02209500	-1.16348400
C	-3.85845800	-0.68347600	-2.80066600
H	-3.11063400	0.02605600	-3.17558600
H	-3.97970600	-1.45843300	-3.56974700
H	-4.81266200	-0.15181800	-2.71754500
C	-2.11763600	-2.12320900	-1.68426000
H	-1.79713800	-2.65680300	-0.78464000
H	-2.25640800	-2.86844600	-2.47954200
H	-1.29292300	-1.46684400	-1.98398400
C	-3.09161200	-2.45186800	1.68947100
H	-3.33669000	-2.92158400	2.65187500
H	-3.56481700	-3.05000600	0.90341900
H	-2.00506300	-2.52801500	1.56146300
C	-2.93067700	-0.23450200	2.87152300
H	-3.17834200	-0.74063000	3.81466400
H	-1.83839100	-0.20914200	2.78600500
H	-3.27778500	0.80025100	2.95176700
C	-4.99613900	2.01177500	1.11651000
H	-5.76947300	2.76906500	0.92840700
H	-5.41167800	1.28646800	1.82427900
H	-4.16050900	2.52123300	1.61194100
C	-4.07052000	2.44720400	-1.18665100
H	-4.85296800	3.20357400	-1.33827500
H	-3.18478100	2.96123300	-0.79559200
H	-3.80730300	2.04353100	-2.16903200

Zero-point correction = 0.409632 (Hartree/Particle)
Thermal correction to Energy = 0.435256
Thermal correction to Enthalpy = 0.436200
Thermal correction to Gibbs Free Energy = 0.351648
Sum of electronic and zero-point Energies = -3637.834565
Sum of electronic and thermal Energies = -3637.808941
Sum of electronic and thermal Enthalpies = -3637.807997
Sum of electronic and thermal Free Energies = -3637.892549
E(RM06L) = -3641.09909095

PC_1a

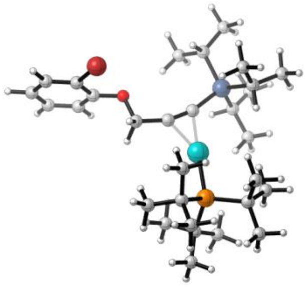

C	4.77012300	-2.18464900	-0.09903300
C	5.74549600	-3.13908100	0.18762900
C	4.15611800	-1.45392100	0.92901500
C	6.11643900	-3.37484400	1.51216700
H	6.21031900	-3.68762100	-0.62449400
C	4.54007000	-1.70047500	2.25176000
C	5.51489900	-2.65314300	2.54492600
H	6.87839300	-4.11739900	1.73054300
H	4.06724000	-1.11712900	3.03638700
H	5.80551200	-2.82830100	3.57679700
C	1.87038300	-0.94272700	0.56152700
C	0.98058600	0.20648200	0.34887000
C	0.62850700	1.39877100	0.19862600
Si	0.65023700	3.25092100	0.04333500
C	2.49363400	3.69612900	-0.27075200
H	2.78314000	3.03032700	-1.09807500
C	-0.48670200	3.67280800	-1.44357500
H	-1.40412500	3.09993900	-1.23701300
C	-0.01638300	3.91954500	1.71719300
H	0.54620600	3.34450600	2.46899300
C	2.75786900	5.14190600	-0.73619800
H	2.46689900	5.88044200	0.01988200
H	3.82876500	5.28944000	-0.93411300
H	2.22060000	5.38531600	-1.65940700
C	3.38677100	3.33082100	0.93203600
H	3.29178500	2.27364500	1.20076700
H	4.44386500	3.51795300	0.69739100
H	3.14226000	3.93225500	1.81725000
C	0.09048000	3.13639800	-2.76867000
H	-0.62474600	3.27813000	-3.59085400
H	1.01289400	3.66014000	-3.04983100
H	0.31910800	2.06637300	-2.70713500
C	-0.89025800	5.15380200	-1.57926800
H	-0.02491000	5.80294400	-1.75757900
H	-1.57353400	5.28964400	-2.42931900
H	-1.40369000	5.52706600	-0.68630300
C	-1.50854000	3.59080500	1.91993400
H	-1.71066900	2.52145100	1.78150900
H	-1.83307600	3.86620600	2.93329100
H	-2.14351800	4.14423200	1.21619500
C	0.25640500	5.41236700	1.98805600
H	-0.23247600	6.06178900	1.25218800
H	-0.12862400	5.69969100	2.97650600
H	1.32638900	5.64577700	1.97525000
Pd	-1.13944600	0.06996200	0.12691200
P	-3.07250100	-1.30858900	0.01971500
C	-3.60131100	-1.92128200	1.79371700
C	-4.54586800	-0.28562200	-0.74210700
C	-2.76477500	-2.87240500	-1.10334700
C	-4.61321800	-3.08353800	1.84284900
C	-2.31713400	-2.33368500	2.55440800

C	-4.18189100	-0.73005800	2.58766100
C	-5.95985800	-0.86319300	-0.53178300
C	-4.48492700	1.14002700	-0.14086900
C	-4.31429800	-0.10401900	-2.25891100
C	-1.94874500	-2.42038700	-2.33883200
C	-1.85797000	-3.86988800	-0.34885600
C	-4.02536100	-3.62312900	-1.57809200
H	-4.86674700	-3.29359000	2.89118200
H	-5.54671900	-2.85395900	1.32245700
H	-4.20904400	-4.00811000	1.42254800
H	-1.58519800	-1.51924900	2.54862200
H	-2.58145600	-2.55572700	3.59742900
H	-1.83718600	-3.22267000	2.14423700
H	-5.16857200	-0.41936000	2.23684200
H	-4.29497600	-1.03276400	3.63702300
H	-3.51012500	0.13438100	2.56474600
H	-6.25049500	-0.88896600	0.52141000
H	-6.06691200	-1.87228200	-0.93820500
H	-6.68814900	-0.22338600	-1.04914900
H	-5.23833900	1.76645900	-0.63798600
H	-4.68903700	1.17068100	0.92942300
H	-3.50214700	1.59532200	-0.30837800
H	-5.03540000	0.63411800	-2.63439000
H	-3.31000100	0.27520800	-2.47332500
H	-4.47119400	-1.02254200	-2.82880300
H	-2.50979800	-1.77526900	-3.01524300
H	-1.65023500	-3.31122700	-2.90822200
H	-1.04438800	-1.88272200	-2.03610500
H	-0.95880300	-3.38246800	0.04192900
H	-2.36856100	-4.37567300	0.47370800
H	-1.53399000	-4.64740500	-1.05307600
H	-4.65111400	-3.01522100	-2.23641600
H	-4.64570800	-3.97471400	-0.74974000
H	-3.71916300	-4.50668400	-2.15506300
Br	4.28753100	-1.85796600	-1.91667300
O	3.23468200	-0.47160700	0.66704100
H	1.59564700	-1.47308300	1.48355500
H	1.78826000	-1.64943200	-0.27343200

Zero-point correction= 0.781638 (Hartree/Particle)
Thermal correction to Energy= 0.829538
Thermal correction to Enthalpy= 0.830482
Thermal correction to Gibbs Free Energy= 0.695813
Sum of electronic and zero-point Energies= -4579.139818
Sum of electronic and thermal Energies= -4579.091919
Sum of electronic and thermal Enthalpies= -4579.090975
Sum of electronic and thermal Free Energies= -4579.225644
E(RM06L) = -4584.17230018

TS_OA_1a

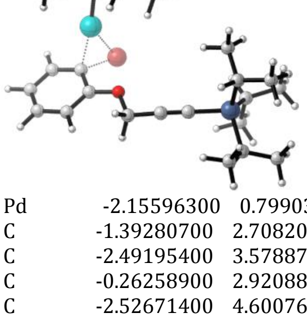

Pd	-2.15596300	0.79903500	0.11845800
C	-1.39280700	2.70820900	0.10645600
C	-2.49195400	3.57887700	0.04217700
C	-0.26258900	2.92088000	-0.72790500
C	-2.52671400	4.60076200	-0.91396200
H	-3.30091600	3.46119200	0.75563700
C	-0.31439800	3.95028900	-1.67269900
C	-1.43946200	4.78176100	-1.76318700
H	-3.39118200	5.25598600	-0.97276400
H	0.51789600	4.11720500	-2.34734700
H	-1.44744700	5.57866700	-2.50151100
P	-3.19401700	-1.35030300	-0.16662300
C	-4.55632400	-1.27252100	-1.55624400
C	-1.85738100	-2.66880200	-0.68109200
C	-4.03112900	-1.92718900	1.49235600
Br	-0.92324600	1.92615700	2.14114500
O	0.80177200	2.08739500	-0.54799000
C	1.97144300	2.33332200	-1.32917000
H	1.74427600	2.20603700	-2.39876700
H	2.30745100	3.37108900	-1.18418700
C	-4.74482900	-0.70313300	2.11756800
H	-5.12902100	-0.98930300	3.10611300
H	-4.04777400	0.13015800	2.25323700
H	-5.59174000	-0.34613700	1.53115100
C	-2.93322400	-2.32189300	2.50507000
H	-2.43467100	-3.26067600	2.25377500
H	-2.17818100	-1.53639000	2.60993200
H	-3.40336200	-2.46103700	3.48737500
C	-5.03438700	-3.09217500	1.37281400
H	-5.39128400	-3.36157200	2.37639100
H	-5.91590000	-2.82731600	0.78271200
H	-4.58942400	-3.98905500	0.93408400
C	-0.58295900	-2.39789500	0.15597400
H	-0.71456300	-2.58676800	1.22157300
H	0.21773300	-3.06136600	-0.19860600
H	-0.24527100	-1.36254300	0.03971600
C	-1.44422100	-2.43618400	-2.15162500
H	-1.15802000	-1.39513600	-2.33473500
H	-0.56708300	-3.06001300	-2.36805800
H	-2.22172900	-2.71720300	-2.86570200
C	-2.26540100	-4.14768600	-0.51986500
H	-1.44952200	-4.78489500	-0.88798800
H	-2.43859700	-4.42360700	0.52354500
H	-3.16163900	-4.40358900	-1.09153500
C	-3.99886000	-0.40960400	-2.71461400
H	-3.16256600	-0.87177300	-3.23953600
H	-4.79806300	-0.24643000	-3.45050700
H	-3.66915600	0.57011200	-2.35077400
C	-5.78290200	-0.50173700	-1.02113700
H	-6.45634700	-0.28857600	-1.86174600
H	-6.35777000	-1.06905500	-0.28554500
H	-5.49432400	0.45740000	-0.57843700
C	-5.03477500	-2.62693800	-2.11648600
H	-5.82283200	-2.45000200	-2.86142900
H	-4.23658600	-3.17776400	-2.62078000
H	-5.45723200	-3.27492100	-1.34394800
C	3.02382100	1.40685500	-0.92614700
C	3.93168500	0.65747300	-0.61615600
Si	5.31214300	-0.47059200	-0.11337400
C	5.13703400	-0.66274500	1.78640200
H	4.07308600	-0.90472700	1.93118900
C	5.01783200	-2.10182500	-1.08038800
H	4.85891500	-1.76824900	-2.11757600
C	6.91495100	0.44281800	-0.64243700
H	6.78722100	1.45662200	-0.23311300
C	5.95628300	-1.80697300	2.41482200
H	7.03518100	-1.66322300	2.28320300
H	5.76847300	-1.86350200	3.49582000
H	5.69832900	-2.78231600	1.98809300
C	5.40003000	0.66962500	2.51672800
H	4.77920000	1.48072400	2.12040700
H	5.17701000	0.57482400	3.58801900
H	6.44981800	0.97792500	2.43114100
C	3.72250500	-2.80152100	-0.62281200
H	3.49902400	-3.66515900	-1.26444300
H	3.80847900	-3.17649700	0.40478200
H	2.85971900	-2.12679900	-0.65908300
C	6.20149700	-3.08864900	-1.09597600
H	6.45512200	-3.44511900	-0.09087700
H	5.95613200	-3.97386800	-1.69902300
H	7.10498100	-2.64267800	-1.52585000
C	7.01013900	0.57845400	-2.17527600
H	6.11147200	1.03979000	-2.60047600

H 7.86995300 1.20060600 -2.45923800
H 7.14362500 -0.39744700 -2.65902300
C 8.22459700 -0.12220900 -0.05822700
H 8.41624700 -1.14882400 -0.39151500
H 9.08171800 0.48574200 -0.37929400
H 8.22008500 -0.12585300 1.03707400
Zero-point correction = 0.781680 (Hartree/Particle)
Thermal correction to Energy = 0.828895
Thermal correction to Enthalpy = 0.829840
Thermal correction to Gibbs Free Energy = 0.699958
Sum of electronic and zero-point Energies = -4579.116809
Sum of electronic and thermal Energies = -4579.069593
Sum of electronic and thermal Enthalpies = -4579.068649
Sum of electronic and thermal Free Energies = -4579.198531
E(RM06L) = -4584.14813722

Ia

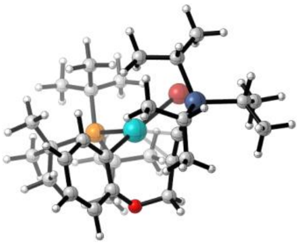

Pd 0.21913300 -0.07881900 -0.14653800
C 0.14315200 1.92171600 0.14036100
C -0.01167600 2.47944400 1.41396500
C 0.03173800 2.77177800 -0.96840100
C -0.20602700 3.85859400 1.57655600
H 0.02864000 1.84332900 2.29450400
C -0.15127400 4.14773300 -0.82091800
C -0.25593800 4.69514700 0.46034200
H -0.31013600 4.27385600 2.57594100
H -0.20820700 4.77091700 -1.70913200
H -0.39293000 5.76612900 0.58191800
P 2.71579000 -0.24054100 0.08247800
C 3.17050700 -1.72828500 1.29048600
C 3.39896700 -0.63140100 -1.70443500
C 3.69230800 1.33341900 0.75414200
Br -0.33586700 -2.62190400 -0.37779300
C -1.49129100 0.59153800 -1.54108400
C -2.20555800 0.10417300 -0.65644300
O 0.13713800 2.25520800 -2.25509400
C 2.44871100 -1.62928900 -2.40153200
H 1.44168000 -1.21962100 -2.50968000
H 2.84660500 -1.83001500 -3.40564200
H 2.35180500 -2.58109300 -1.88147600
C 3.39352200 0.65038400 -2.56805200
H 3.58981900 0.35592200 -3.60742800
H 2.42840600 1.16582100 -2.54804800
H 4.17915200 1.35495800 -2.28476800
C 4.82793400 -1.21969300 -1.72923600
H 4.89541300 -2.19089000 -1.23665700
H 5.11319800 -1.37252700 -2.77866000
H 5.57321000 -0.55831100 -1.28584900
C 3.24364800 2.65587300 0.08113800
H 3.99736800 3.41774000 0.32146300
H 3.16699400 2.60446000 -1.00265200
H 2.29268500 3.00649800 0.47039400
C 3.39434400 1.51585900 2.25978200
H 2.32011300 1.56681600 2.45250400
H 3.82716900 0.74123900 2.89361000
H 3.82615100 2.47372600 2.57828700
C 5.22430900 1.24813100 0.54414800
H 5.68014600 0.31922400 0.88144200
H 5.49509400 1.40279300 -0.50377100
H 5.68729300 2.06474900 1.11378200
C 4.60872700 -1.71271000 1.85488600
H 4.83494500 -0.85085800 2.48329200
H 4.72396800 -2.60407500 2.48502600
H 5.36593200 -1.77921700 1.06865400
C 2.15837400 -1.67450700 2.45897900
H 2.17979900 -0.73072700 3.00871400
H 1.14216700 -1.84909200 2.09609700
H 2.40240900 -2.47523700 3.16978700
C 2.99640700 -3.10570700 0.61142100
H 3.75672600 -3.30466300 -0.14689700
H 3.11661900 -3.87065900 1.39040600
H 2.00558300 -3.23464400 0.17892000
C -0.92893900 1.37952100 -2.65238800
H -1.74185900 1.97567900 -3.09410100
H -0.51592600 0.72880100 -3.42877600
Si -3.73455700 -0.33410100 0.34241000
C -4.84060800 -1.46576200 -0.75179400
C -3.38135800 -1.18330900 2.02698100
C -4.62826300 1.34784800 0.66486800
H -5.49143300 -1.96265800 -0.01296400
C -5.76299400 -0.72299300 -1.73754500
C -4.05762900 -2.56378000 -1.49865500
H -4.25724600 -0.87701900 2.62472900
C -3.36409900 -2.72459000 2.00701000
C -2.12448900 -0.65593600 2.74565800
H -5.68463400 1.05438900 0.78664100
C -4.20458300 2.07100500 1.95812200
C -4.55226300 2.33502600 -0.51720100
H -6.41670800 -1.43764300 -2.25595000
H -6.40762700 0.01300600 -1.24470000
H -5.18521800 -0.20070900 -2.51027100
H -4.75364700 -3.26957600 -1.97277900
H -3.43605600 -2.12875000 -2.28956400
H -3.38545100 -3.12961600 -0.84914600
H -3.29275900 -3.10921600 3.03393900
H -4.27139000 -3.14872500 1.56303900
H -2.49996300 -3.09867300 1.44956300
H -2.05595200 -1.08192300 3.75644500
H -1.22163700 -0.95291400 2.20113500
H -2.11729300 0.43405500 2.84367200
H -3.16033900 2.40225500 1.90718900
H -4.82025300 2.96844200 2.10951900
H -4.31792100 1.44373500 2.84839700
H -4.88147600 1.89309200 -1.46269700
H -5.18532200 3.21231400 -0.32581600
H -3.52656900 2.69594400 -0.65517900
Zero-point correction = 0.784575 (Hartree/Particle)
Thermal correction to Energy = 0.831271
Thermal correction to Enthalpy = 0.832215
Thermal correction to Gibbs Free Energy = 0.707565
Sum of electronic and zero-point Energies = -4579.132300
Sum of electronic and thermal Energies = -4579.085604
Sum of electronic and thermal Enthalpies = -4579.084659
Sum of electronic and thermal Free Energies = -4579.209309
E(RM06L) = -4584.18585821

TS_AI_Ia

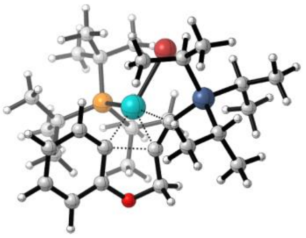

Pd -0.17561400 0.02505000 -0.15768800
C -0.13975000 2.18026100 -0.28474700
C -0.16071200 2.45989300 -1.66246400
C -0.40838500 3.23067800 0.60425100
C -0.57172100 3.71552700 -2.12108600
H 0.11992400 1.69586100 -2.38127900
C -0.80460400 4.49119800 0.16160500
C -0.91271200 4.72130200 -1.21082000
H -0.61508200 3.90756200 -3.18949300
H -0.99112000 5.27433000 0.89002000
H -1.22071700 5.69960900 -1.56896800
P -2.73709300 -0.50629500 0.07109100
C -3.29743200 -2.15335600 -0.84212000
C -2.92869800 -0.78389000 1.99441700
C -4.02627300 0.86969800 -0.46508800

Br	0.60570700	-2.34520300	-0.79657800
C	1.21101600	1.35451500	0.86654800
C	1.93955000	0.44471400	0.33883100
O	-0.16917100	3.01869600	1.93683800
C	-1.69767100	-1.58371400	2.48154500
H	-0.76762200	-1.04291700	2.28013300
H	-1.78051400	-1.72535600	3.56785600
H	-1.60950900	-2.56796800	2.02204000
C	-2.88614600	0.57179700	2.73525200
H	-2.79303600	0.37066200	3.81097600
H	-2.03081800	1.18861600	2.44355600
H	-3.79890500	1.15779400	2.60095200
C	-4.20737900	-1.52455900	2.44206600
H	-4.25719800	-2.54897400	2.06864000
H	-4.20804600	-1.58350700	3.53907400
H	-5.12246600	-1.00834000	2.14591400
C	-3.50440000	2.27084500	-0.08261000
H	-4.30048200	2.99902100	-0.28952700
H	-3.23432400	2.37301900	0.96708300
H	-2.64464800	2.54944800	-0.68706900
C	-4.14439200	0.89547800	-2.00654000
H	-3.16233200	0.96477100	-2.48569100
H	-4.67454800	0.03684000	-2.42080200
H	-4.70985600	1.79258400	-2.29169000
C	-5.43664400	0.72578400	0.14953900
H	-5.89572200	-0.24915000	-0.01114200
H	-5.43323300	0.93020500	1.22331300
H	-6.09099900	1.47633900	-0.31437100
C	-4.82440700	-2.37140600	-0.93202400
H	-5.33614200	-1.62148500	-1.53743100
H	-4.99599800	-3.34147500	-1.41727300
H	-5.30764000	-2.41401300	0.04772600
C	-2.71565300	-2.11772300	-2.27598400
H	-3.09383400	-1.28869800	-2.87585100
H	-1.62512300	-2.07368900	-2.25679800
H	-3.00347600	-3.04633800	-2.78727200
C	-2.71115500	-3.40775500	-0.15353000
H	-3.17180600	-3.61582000	0.81495900
H	-2.92596900	-4.27006500	-0.79891600
H	-1.62881800	-3.35105900	-0.04034300
C	0.96847200	2.16275100	2.10189400
H	1.84611300	2.78228100	2.33593900
H	0.76805500	1.50034900	2.94850200
Si	3.59708000	-0.37585100	0.00771600
C	4.90457100	0.93490800	0.54530100
C	3.79785100	-0.78956300	-1.85916700
C	3.74703400	-1.98096000	1.05355400
H	5.87079100	0.46109200	0.30921600
C	4.82535400	2.24748400	-0.25843700
C	4.90616400	1.24112400	2.05512500
H	3.35051300	-1.78707000	-1.95687400
C	3.01607700	0.13766500	-2.80856700
C	5.27535800	-0.88939700	-2.29185800
H	3.24916000	-2.73784500	0.43390800
C	2.98596100	-1.94192700	2.39244100
C	5.20871000	-2.42663600	1.26582200
H	5.61271800	2.94556100	0.05879900
H	4.94599900	2.08592500	-1.33459800
H	3.86238000	2.75192300	-0.10958600
H	5.70328300	1.95511500	2.30542200
H	3.95930500	1.69339400	2.37568100
H	5.06398400	0.34570900	2.66459000
H	3.34681900	1.18138400	-2.74013900
H	3.15408100	-0.18016500	-3.85151100
H	1.94416400	0.10353000	-2.59174500
H	5.34325700	-1.22801700	-3.33466600
H	5.78730300	0.07918400	-2.23492800
H	5.84720900	-1.60024400	-1.68428400
H	1.91855800	-1.76106100	2.23287500
H	3.08485700	-2.90368300	2.91475100
H	3.36841100	-1.16700100	3.06855100
H	5.77676400	-1.71047400	1.87233500
H	5.23863300	-3.38959800	1.79375500
H	5.74893600	-2.55996700	0.32155600

Zero-point correction = 0.783044 (Hartree/Particle)
Thermal correction to Energy = 0.829321
Thermal correction to Enthalpy = 0.830265
Thermal correction to Gibbs Free Energy = 0.706654
Sum of electronic and zero-point Energies = -4579.116960
Sum of electronic and thermal Energies = -4579.070682
Sum of electronic and thermal Enthalpies = -4579.069738
Sum of electronic and thermal Free Energies = -4579.193349
E(RM06L) = -4584.17014097

IIa

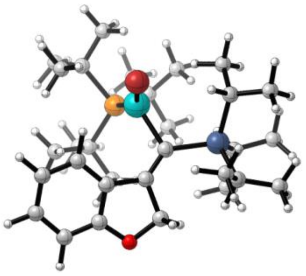

Pd	-0.28234500	-0.13411300	0.98884800
C	0.25781300	2.88945400	-0.30170700
C	-0.55888300	3.17547900	0.79741800
C	0.36398800	3.84210200	-1.32998900
C	-1.27704300	4.37612300	0.81996700
H	-0.60065400	2.49139800	1.63669900
C	-0.35619100	5.03076000	-1.33698500
C	-1.18874900	5.28380600	-0.24213100
H	-1.90006000	4.60920700	1.67883800
H	-0.25045700	5.73485000	-2.15582800
H	-1.75542600	6.21066100	-0.20852100
P	-2.30747700	-0.88324500	-0.31321800
C	-2.26957400	-2.66521700	-1.11249400
C	-3.53885500	-0.94078200	1.20977200
C	-3.01623500	0.34980300	-1.64212400
Br	0.90590100	0.61954200	3.03054300
C	1.15140200	1.76047800	-0.64845100
C	1.23435200	0.48782000	-0.20222000
O	1.24529700	3.48185100	-2.30447400
C	1.99321800	2.35727900	-1.79563500
H	2.16699800	1.67599200	-2.62820200
H	2.95360100	2.74206400	-1.42906000
C	-1.86847300	0.71009200	-2.61562500
H	-1.54527100	-0.12949500	-3.23182300
H	-2.22315900	1.49499800	-3.29638100
H	-0.99864500	1.09998400	-2.08313000
C	-3.43426700	1.66934600	-0.96009900
H	-3.66598900	2.40112900	-1.74456600
H	-4.33232500	1.56579400	-0.34662100
H	-2.63279000	2.09412300	-0.35212000
C	-4.22076800	-0.16884700	-2.45540100
H	-4.55126300	0.63168800	-3.13068100
H	-3.97286500	-1.02901900	-3.08062400
H	-5.07465300	-0.43607400	-1.82810700
C	-1.63531700	-2.59393100	-2.51913600
H	-1.43334500	-3.61804800	-2.85883800
H	-2.29087500	-2.13440500	-3.26106600
H	-0.68343700	-2.05786600	-2.51644300
C	-1.33836700	-3.56545200	-0.26932800
H	-1.68955400	-3.72262700	0.75063500
H	-1.27852400	-4.55114400	-0.74991800
H	-0.32652600	-3.16055100	-0.21565200
C	-3.64027000	-3.36308900	-1.23900200
H	-4.08321700	-3.59777000	-0.26850800
H	-4.36534900	-2.78442100	-1.81517300
H	-3.49650200	-4.31801500	-1.76233000
C	-5.03823400	-0.99207700	0.85094600
H	-5.30153900	-1.84721500	0.22469200
H	-5.61652900	-1.07858000	1.78088400
H	-5.37714200	-0.08303400	0.34862600
C	-3.28951900	0.30525800	2.09605300
H	-3.98653400	0.26999000	2.94434100
H	-2.27677200	0.31778300	2.51717900
H	-3.44581000	1.25130100	1.57902000
C	-3.20310300	-2.15826600	2.10084300

H	-3.48835000	-3.11156300	1.65152300
H	-2.14007400	-2.19893000	2.36320200
H	-3.76339700	-2.06330100	3.03986000
Si	2.68556500	-0.78815700	-0.39774400
C	4.26630600	0.25074900	-0.80673000
H	3.97080200	0.91243600	-1.62834500
C	2.95708300	-1.77366000	1.23750100
H	2.85397100	-1.01407700	2.02246900
C	2.21231200	-1.96104800	-1.85286500
H	1.21723900	-2.33092700	-1.56825000
C	4.36045400	-2.40016200	1.38673400
H	4.42739600	-2.92511800	2.34960200
H	4.58171400	-3.13714700	0.60523100
H	5.15818700	-1.65247600	1.37462200
C	1.88938800	-2.84462400	1.52369800
H	1.90100100	-3.65042100	0.77910900
H	2.06746700	-3.30490300	2.50527100
H	0.88254600	-2.41480200	1.54757000
C	5.47700300	-0.53793100	-1.35182900
H	6.30878400	0.15136800	-1.55496500
H	5.84777000	-1.29067500	-0.65038900
H	5.24444000	-1.04727100	-2.29298500
C	4.68331200	1.15316300	0.37384100
H	3.84972200	1.75696100	0.74911900
H	5.05862800	0.56556500	1.21925700
H	5.48916800	1.83722500	0.07322800
C	2.05569600	-1.22413700	-3.19664800
H	1.33223000	-0.40336900	-3.13135700
H	3.00953800	-0.80424200	-3.54073500
H	1.70687700	-1.90833700	-3.98310400
C	3.11041900	-3.20287600	-2.02495500
H	2.71446400	-3.85317900	-2.81802400
H	4.13335600	-2.93687500	-2.31156100
H	3.17091900	-3.80300600	-1.11174500

Zero-point correction = 0.786026 (Hartree/Particle)
Thermal correction to Energy = 0.832078
Thermal correction to Enthalpy = 0.833022
Thermal correction to Gibbs Free Energy = 0.710496
Sum of electronic and zero-point Energies = -4579.171201
Sum of electronic and thermal Energies = -4579.125149
Sum of electronic and thermal Enthalpies = -4579.124205
Sum of electronic and thermal Free Energies = -4579.246731
E(RM06L) = -4584.22763589

TS_RE_IIa

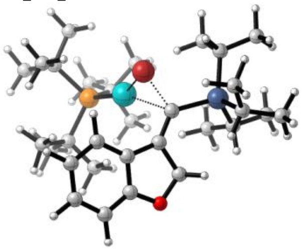

Pd	0.71532000	-0.26373200	-0.42524800
C	-1.96520000	0.42662000	-0.27137800
P	2.92919600	-0.55859300	0.10859200
C	3.26091400	-2.20898600	1.10441400
C	3.99988800	-0.63335600	-1.52932700
C	3.59941000	0.92874700	1.18716700
Br	-1.35774500	0.32017300	-2.15210800
C	-1.73543300	1.59345100	0.38954200
C	-1.11025700	2.87581600	0.04031000
C	-2.21205300	1.79267900	1.83583700
C	-1.26713700	3.72870700	1.14981100
H	-3.29139900	1.65168100	1.95605000
H	-1.69238600	1.13466400	2.53819800
O	-1.90744700	3.14984700	2.19900500
C	-0.46396100	3.38120900	-1.10005300
C	-0.81341800	5.04312800	1.16969400
H	-0.95857900	5.66038100	2.04989800
C	-0.00185800	4.69779800	-1.09802900
C	-0.17449100	5.51947400	0.02396800
H	-0.31595200	2.75818700	-1.97024700
H	0.49850100	5.08712500	-1.97974200
H	0.19303700	6.54194300	0.00524900
C	3.45922400	0.43931200	-2.50563600
H	3.95933000	0.31618500	-3.47656500
H	2.38066400	0.32066800	-2.65112500
H	3.64265800	1.46085000	-2.17134200
C	5.52102600	-0.43608800	-1.36879400
H	5.97536300	-1.16336100	-0.69160100
H	6.00297700	-0.55795200	-2.34904700
H	5.77640400	0.56514000	-1.01229400
C	3.75088000	-1.98761600	-2.22930100
H	2.68011500	-2.19125300	-2.33581100
H	4.18425300	-1.94619200	-3.23762700
H	4.21987900	-2.82948100	-1.71485500
C	2.37484500	-3.31841200	0.48926300
H	2.66456700	-3.59202300	-0.52566000
H	2.45640500	-4.22200800	1.10963800
H	1.32601900	-3.00743800	0.46544100
C	4.72079000	-2.70436300	1.15183100
H	4.77372900	-3.60118600	1.78509200
H	5.09860500	-2.98907300	0.16666600
H	5.40507900	-1.96598200	1.57738500
C	2.76033300	-2.03919800	2.55590700
H	1.73530700	-1.65703700	2.58676500
H	2.76279900	-3.02310100	3.04407500
H	3.39640800	-1.38390100	3.15521700
C	2.52685800	1.26396700	2.25129300
H	2.38810100	0.47476300	2.99113600
H	2.83477700	2.17023700	2.79102100
H	1.56000700	1.45770500	1.77565900
C	3.70726100	2.18990700	0.30145800
H	3.88326800	3.05835400	0.95031400
H	4.53914500	2.14565300	-0.40538100
H	2.78121300	2.37371200	-0.25257100
C	4.95435000	0.71313100	1.89183100
H	5.23757600	1.63843500	2.41294100
H	4.91464300	-0.07637700	2.64653500
H	5.76073800	0.47427500	1.19399300
Si	-3.17835800	-0.99348600	0.22057400
C	-2.86380300	-1.49254900	2.05828100
H	-3.26965400	-0.65903000	2.65217900
C	-3.62939800	-2.76082400	2.49730200
H	-3.23587700	-3.65333000	1.99815800
H	-3.50930400	-2.91907700	3.57777000
H	-4.70269000	-2.71012300	2.29104000
C	-1.37432700	-1.65269100	2.42936800
H	-1.25702500	-1.74027700	3.51863400
H	-0.95705900	-2.56264600	1.98517700
H	-0.74768100	-0.82436300	2.07985200
C	-4.89793200	-0.12698100	0.03846600
H	-4.74550800	0.86318700	0.49533000
C	-6.07819800	-0.77730000	0.78653000
H	-6.99361300	-0.18753400	0.63931800
H	-6.28906100	-1.79158900	0.42862100
H	-5.90040200	-0.83574700	1.86587400
C	-5.26236900	0.12717800	-1.43947500
H	-4.46754900	0.65591000	-1.97677000
H	-5.45784700	-0.80878200	-1.97618400
H	-6.17293500	0.73729500	-1.51184100
C	-2.99154400	-2.45531300	-1.00860700
H	-2.98536600	-1.97886800	-1.99789900
C	-4.17667700	-3.44532100	-0.99962000
H	-4.02908500	-4.21168400	-1.77271100
H	-4.27222200	-3.97022000	-0.04289200
H	-5.13423300	-2.95650100	-1.20703800
C	-1.66018300	-3.22043800	-0.86714400
H	-1.62377000	-3.79303500	0.06762400
H	-1.54401700	-3.93943700	-1.68994200
H	-0.78859200	-2.55334400	-0.88492500

Zero-point correction = 0.784076 (Hartree/Particle)
Thermal correction to Energy = 0.829995
Thermal correction to Enthalpy = 0.830939
Thermal correction to Gibbs Free Energy = 0.705463
Sum of electronic and zero-point Energies = -4579.156778
Sum of electronic and thermal Energies = -4579.110860
Sum of electronic and thermal Enthalpies = -4579.109916
Sum of electronic and thermal Free Energies = -4579.235392

E(RM06L) = -4584.20263426

***cis*-2a**

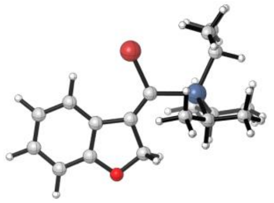

C	2.66445400	-0.06457500	-0.00026700
C	1.24257600	-1.96036000	-0.00059800
C	3.39539000	-1.26809200	-0.00008800
H	0.76493200	-2.38284000	0.88884400
H	0.76581800	-2.38232100	-0.89077900
O	2.61749700	-2.38203000	-0.00003600
C	3.37588500	1.14568500	-0.00032900
C	4.78523300	-1.31365800	0.00005300
H	5.30356100	-2.26662500	0.00019600
C	4.77099700	1.11978300	-0.00020600
C	5.46716600	-0.09581000	-0.00000700
H	2.85158900	2.09066300	-0.00048500
H	5.32095200	2.05617300	-0.00026800
H	6.55388100	-0.09465300	0.00009100
C	1.23910900	-0.42446700	-0.00028300
C	0.08485500	0.28286300	-0.00007800
Br	0.21242100	2.22686900	0.00009600
Si	-1.70841700	-0.40654300	0.00006600
C	-3.08732500	0.93075100	0.00020000
H	-3.98819300	0.29458600	0.00039200
C	-1.89446100	-1.52844900	1.56737800
H	-1.40496100	-2.47721500	1.29746900
C	-1.89460000	-1.52837600	-1.56726700
H	-1.40469300	-2.47699100	-1.29757100
C	-1.17688900	-0.98644800	-2.82058400
H	-0.11169000	-0.80540000	-2.64441000
H	-1.26321200	-1.69897600	-3.65231700
H	-1.61270400	-0.03976000	-3.15950300
C	-3.35952100	-1.87771800	-1.90668200
H	-3.93166600	-0.98993700	-2.19942500
H	-3.39845300	-2.57923700	-2.75109500
H	-3.88599000	-2.34896800	-1.06999600
C	-3.17165800	1.80929900	1.26617900
H	-4.06473800	2.44784400	1.22275500
H	-3.24128100	1.21612500	2.18383300
H	-2.30165800	2.46591300	1.35871400
C	-3.17206300	1.80909700	-1.26588900
H	-4.06498500	2.44784200	-1.22216600
H	-2.30196500	2.46550700	-1.35896300
H	-3.24225700	1.21575700	-2.18339200
C	-1.17617400	-0.98682700	2.82049500
H	-1.61149200	-0.03995300	3.15953900
H	-1.26256100	-1.69932000	3.65225100
H	-0.11095100	-0.80622200	2.64402400
C	-3.35938600	-1.87734300	1.90725400
H	-3.39824500	-2.57900400	2.75155200
H	-3.93111500	-0.98942100	2.20037300
H	-3.88633500	-2.34825100	1.07067800

Zero-point correction = 0.411633 (Hartree/Particle)
Thermal correction to Energy = 0.436293
Thermal correction to Enthalpy = 0.437237
Thermal correction to Gibbs Free Energy = 0.357322
Sum of electronic and zero-point Energies = -3637.877049
Sum of electronic and thermal Energies = -3637.852388
Sum of electronic and thermal Enthalpies = -3637.851444
Sum of electronic and thermal Free Energies = -3637.931360
E(RM06L) = -3641.14431049

TS_Isom_IIa

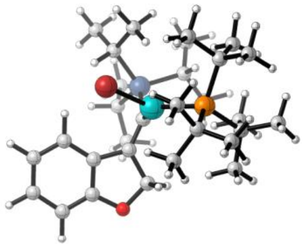

Pd	0.39878300	-0.30487700	0.33543400
C	-2.64193700	-2.03507000	-0.39026500
C	-3.30291300	-2.11726300	0.84020500
C	-2.87846300	-3.00785400	-1.37624900
C	-4.20708800	-3.15915200	1.04646200
H	-3.09122600	-1.39974900	1.62623800
C	-3.77718300	-4.05183200	-1.18797800
C	-4.44101100	-4.10949800	0.04112700
H	-4.72329300	-3.24413300	1.99785300
H	-3.94255400	-4.78918700	-1.96635500
H	-5.14472900	-4.91721300	0.22308400
P	2.72436400	0.06050800	-0.36038000
Br	0.68787700	-1.53388000	2.56152400
C	-1.68169400	-1.07652600	-0.94102600
C	-1.31994900	0.15466000	-0.45766600
O	-2.15443800	-2.81146200	-2.51367500
C	-1.27747800	-1.68753400	-2.28838100
H	-0.24965700	-2.06243800	-2.27862300
H	-1.39488800	-0.98378600	-3.11878200
C	3.56990500	1.43342000	0.73111300
C	2.79193800	0.63326300	-2.22888900
C	3.74503900	-1.59609800	-0.20491500
C	3.22261900	1.18016200	2.21655100
H	2.14675100	1.10288000	2.38138200
H	3.67463000	0.27351000	2.61595500
H	3.60310300	2.02667300	2.80402900
C	2.96539600	2.80910200	0.37458100
H	3.30780300	3.53998700	1.11814900
H	3.28154200	3.17676100	-0.60436000
H	1.87131600	2.79576600	0.40937800
C	5.10593400	1.52145400	0.60033900
H	5.44380600	1.71586600	-0.41930300
H	5.45876000	2.35309000	1.22510000
H	5.60549400	0.61943800	0.96058000
C	4.12358700	-1.87132500	1.26762400
H	4.53309000	-2.88879000	1.32099000
H	4.90005200	-1.19722300	1.63702800
H	3.25664700	-1.82956400	1.93060300
C	5.04913600	-1.64117900	-1.03210200
H	5.55117600	-2.59547000	-0.82456600
H	4.88547100	-1.59827400	-2.11080700
H	5.74729700	-0.84495200	-0.75981300
C	2.81735400	-2.75863300	-0.63500800
H	1.94097500	-2.81920900	0.01673700
H	2.47953900	-2.68553800	-1.67034700
H	3.37392400	-3.70040400	-0.53865700
C	2.47542900	-0.56036500	-3.15731500
H	1.52812100	-1.03805500	-2.89383600
H	2.37557700	-0.18190000	-4.18288900
H	3.25522500	-1.32284200	-3.17057300
C	1.66857800	1.66165700	-2.48184700
H	1.80067200	2.59412800	-1.93249600
H	1.65632000	1.91087800	-3.55124500
H	0.69398700	1.24822900	-2.21323100
C	4.13106000	1.25722500	-2.67873800
H	4.07867000	1.45446600	-3.75798600
H	4.32837600	2.21559100	-2.19218400
H	4.98894100	0.60388600	-2.50928700
Si	-2.30192300	1.70028500	0.09290300
C	-1.45882500	3.21424800	-0.74496300
H	-0.39239700	3.07284800	-0.51504900
C	-4.14294700	1.47074500	-0.46213000
H	-4.56690100	0.76517400	0.26926100
C	-2.19422700	1.77486100	2.01907000
H	-2.25989100	0.71955800	2.32197100
C	-4.96650300	2.77645500	-0.37392400

H	-4.66718100	3.48596900	-1.15443700
H	-6.03279100	2.56255200	-0.52977800
H	-4.87269700	3.28540000	0.58949300
C	-3.34555800	2.51831400	2.72515300
H	-4.32657200	2.09754500	2.47994200
H	-3.22389000	2.44707300	3.81452900
H	-3.36649900	3.58617200	2.47465000
C	-1.87412000	4.58637500	-0.17470800
H	-1.28059900	5.38643300	-0.63883000
H	-2.92707800	4.80989700	-0.37725600
H	-1.72429800	4.65513100	0.90712800
C	-4.34734700	0.85005200	-1.85808600
H	-3.92364400	1.48007100	-2.64851000
H	-3.90158000	-0.14353300	-1.94409400
H	-5.41995600	0.74358000	-2.07062900
C	-0.83340600	2.28537200	2.52720600
H	-0.68746500	3.35013100	2.30918600
H	-0.75849700	2.15753100	3.61492200
H	-0.00304800	1.72686900	2.08310000
C	-1.60490400	3.22245300	-2.27962300
H	-1.32126700	2.26689200	-2.73388800
H	-2.63753700	3.43577500	-2.57932900
H	-0.97243300	4.00181800	-2.72670100

Zero-point correction = 0.784398 (Hartree/Particle)
Thermal correction to Energy = 0.830269
Thermal correction to Enthalpy = 0.831214
Thermal correction to Gibbs Free Energy = 0.707448
Sum of electronic and zero-point Energies = -4579.158141
Sum of electronic and thermal Energies = -4579.112270
Sum of electronic and thermal Enthalpies = -4579.111325
Sum of electronic and thermal Free Energies = -4579.235091
E(RM06L) = -4584.21534984

IIIa

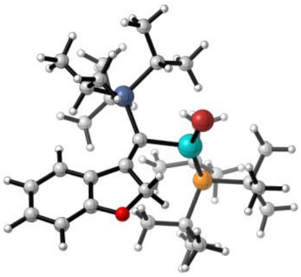

Pd	0.70363200	-0.42937200	-0.96869700
C	-2.48141100	2.26068700	0.08393100
C	-3.29790300	2.26169800	1.22345700
C	-2.46456300	3.42122000	-0.71570300
C	-4.10438800	3.36689100	1.50180900
H	-3.30288700	1.42187300	1.90432800
C	-3.26533000	4.53099100	-0.45911100
C	-4.09555700	4.48698800	0.66160700
H	-4.73786300	3.35662000	2.38395500
H	-3.22352300	5.39591600	-1.11285800
H	-4.73002500	5.33907200	0.89015600
P	2.64156500	0.30407400	0.46447300
C	2.97049200	-0.61267200	2.15466300
C	4.06207000	-0.20404600	-0.78018300
C	2.75994300	2.22081700	0.78950300
Br	-0.37788100	-1.09624300	-3.09878900
C	-1.48844000	1.32092900	-0.48400900
C	-1.09861700	0.06897000	-0.14432600
O	-1.57909500	3.36914400	-1.73862400
C	-0.90246600	2.09693000	-1.67412700
H	-1.07885000	1.55229400	-2.60541400
H	0.17071800	2.28820500	-1.58628200
C	1.41955100	2.68062800	1.41293600
H	1.26476400	2.30072800	2.42308800
H	1.42069000	3.77664300	1.47505200
H	0.56112700	2.37899300	0.81002700
C	2.90171200	2.97105300	-0.55305800
H	2.75305200	4.04231100	-0.36767800
H	3.89097000	2.85792700	-1.00237500
H	2.15236300	2.66108900	-1.28596800
C	3.91325300	2.67820600	1.70771400
H	3.89548000	3.77478300	1.76629200
H	3.81217200	2.30508700	2.72905000
H	4.89810600	2.38952600	1.33273500
C	2.10004800	0.01862400	3.26452400
H	2.14367300	-0.63170500	4.14764300
H	2.45367900	1.00337200	3.57648000
H	1.04976100	0.10102600	2.97160100
C	2.49320900	-2.07594000	2.02108700
H	3.06857100	-2.65921900	1.30250300
H	2.59711900	-2.56726900	2.99766800
H	1.44305600	-2.12896100	1.73088000
C	4.43801300	-0.61920700	2.63299400
H	5.08946600	-1.19745700	1.97347200
H	4.85987200	0.38260700	2.73752900
H	4.47982800	-1.09671100	3.62127900
C	5.43907500	0.43629600	-0.51099700
H	5.81888400	0.22230200	0.49021700
H	6.16229300	0.02749500	-1.22950600
H	5.43023800	1.51977500	-0.64989400
C	3.61743900	0.16697400	-2.21692000
H	4.40575300	-0.14639700	-2.91454100
H	2.70102800	-0.35563100	-2.51987600
H	3.45477100	1.23416700	-2.36441100
C	4.23096700	-1.74097900	-0.78088900
H	4.70347900	-2.12043500	0.12746300
H	3.27920600	-2.26279600	-0.92682000
H	4.88267500	-2.01433800	-1.62062600
Si	-2.06853300	-1.42155300	0.61583700
C	-3.92626000	-1.01166100	0.27636200
H	-4.03040400	0.05690500	0.48422400
C	-1.63190800	-3.07310600	-0.28830200
H	-1.64992100	-2.79700600	-1.35004300
C	-1.58864700	-1.55702000	2.48478800
H	-0.49261300	-1.63986800	2.44487000
C	-2.67232500	-4.19805900	-0.09008400
H	-2.39594600	-5.06470900	-0.70650700
H	-2.72174800	-4.54822500	0.94717600
H	-3.68163600	-3.90250000	-0.38700900
C	-0.22808600	-3.63044100	0.00051500
H	-0.10230800	-3.91508900	1.05315400
H	-0.04350100	-4.52956900	-0.60372700
H	0.55386600	-2.90546700	-0.25204900
C	-4.94569800	-1.72445800	1.18923900
H	-5.96738700	-1.41591300	0.92647500
H	-4.90635800	-2.81516100	1.09933900
H	-4.79535800	-1.47587000	2.24629000
C	-4.29284100	-1.18037700	-1.21458800
H	-3.59569100	-0.64755900	-1.86970600
H	-4.29609500	-2.22970700	-1.52779400
H	-5.29966700	-0.78273300	-1.40267400
C	-1.90277200	-0.30943900	3.32862800
H	-1.48227100	0.60196700	2.89026600
H	-2.98437500	-0.16042200	3.44171600
H	-1.49069500	-0.41083900	4.34272900
C	-2.10238800	-2.82093300	3.20347700
H	-1.73741300	-2.84467000	4.24028500
H	-3.19659700	-2.85406100	3.24751700
H	-1.76308200	-3.74070000	2.71856200

Zero-point correction = 0.785711 (Hartree/Particle)
Thermal correction to Energy = 0.831925
Thermal correction to Enthalpy = 0.832869
Thermal correction to Gibbs Free Energy = 0.709077
Sum of electronic and zero-point Energies = -4579.169847
Sum of electronic and thermal Energies = -4579.123634
Sum of electronic and thermal Enthalpies = -4579.122690
Sum of electronic and thermal Free Energies = -4579.246482
E(RM06L) = -4584.22388830

TS_RE_IIIa

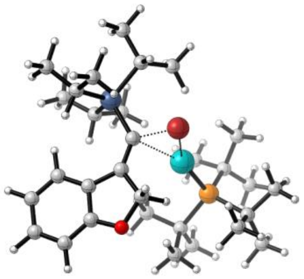

Pd	0.87233300	-0.41198100	-0.44725800
C	-1.80000200	-0.00827100	-0.78969000
P	3.04037700	-0.08194700	0.24051300
C	3.75038200	-1.59366300	1.25887300
C	4.19564100	0.16630400	-1.31924100
C	3.19735600	1.51606500	1.35733100
Br	-1.04833400	-0.95182700	-2.38197300
C	-1.68387200	1.34658500	-0.87289200
C	-2.20998700	2.42563100	-0.02272100
C	-0.94652400	2.07997300	-1.99268800
C	-1.71118100	3.63653400	-0.54592400
H	0.08513600	1.74097400	-2.10683000
H	-1.46519700	1.96985300	-2.95461100
O	-0.93140400	3.47693600	-1.64322700
C	-3.05962200	2.49045000	1.09247700
C	-1.99872200	4.87926600	0.01006300
H	-1.58688600	5.78223100	-0.42807000
C	-3.36349000	3.72780900	1.66102400
C	-2.83196600	4.90974900	1.12875700
H	-3.49602100	1.59653300	1.51677000
H	-4.02273100	3.77158800	2.52275400
H	-3.07785000	5.86471500	1.58496900
C	3.44116500	1.05370600	-2.33871200
H	4.03729100	1.11880400	-3.25973500
H	2.46966400	0.61357800	-2.58608500
H	3.27156900	2.07212000	-1.98727500
C	5.58149900	0.78887900	-1.05315000
H	6.17379300	0.21914100	-0.33306000
H	6.14853800	0.81460500	-1.99423200
H	5.51404200	1.82003500	-0.69626200
C	4.39459900	-1.19108300	-2.02989000
H	3.43847700	-1.69403500	-2.20863500
H	4.86181600	-1.00808300	-3.00682100
H	5.05280600	-1.87118800	-1.48445900
C	3.24458300	-2.89203800	0.58536500
H	3.67411700	-3.06082500	-0.40256700
H	3.52068000	-3.74919900	1.21522700
H	2.15548100	-2.87686400	0.47733400
C	5.28490500	-1.66789400	1.39932200
H	5.54368100	-2.53319200	2.02559800
H	5.79004400	-1.80786300	0.44046400
H	5.70822900	-0.78222600	1.88003500
C	3.13945400	-1.59431400	2.67751500
H	2.04838900	-1.52245600	2.64982300
H	3.39611200	-2.54334200	3.16728700
H	3.52695300	-0.79339700	3.31137100
C	2.01115600	1.51158200	2.35140200
H	2.06316500	0.70633600	3.08485500
H	2.00970400	2.46082900	2.90497200
H	1.05902800	1.42097200	1.81832100
C	2.99371800	2.77084500	0.48002700
H	2.88241800	3.64355000	1.13733600
H	3.83962700	2.97443000	-0.18084100
H	2.08395700	2.69661400	-0.12472100
C	4.51177700	1.68198000	2.14638000
H	4.48086400	2.63308800	2.69624000
H	4.65858700	0.89204800	2.88700300
H	5.39334500	1.70941500	1.50104600
Si	-2.86426700	-1.13346300	0.35846700
C	-2.29481800	-0.74388100	2.16697700
H	-1.98687900	0.30904000	2.14350600
C	-3.36853000	-0.88253600	3.26482900
H	-3.82221300	-1.88109200	3.27789400
H	-2.91933500	-0.71830600	4.25389400
H	-4.17896800	-0.15445400	3.15537700
C	-1.03432100	-1.53611000	2.56911000
H	-0.63240000	-1.14077600	3.51231700
H	-1.25492800	-2.59692900	2.73062300
H	-0.24249900	-1.45758000	1.81229300
C	-4.65829300	-0.55729800	-0.11879600
H	-4.59222500	0.53990500	-0.12773100
C	-5.83887200	-0.92173900	0.80368500
H	-6.77045400	-0.50143700	0.39976800
H	-5.98758000	-2.00397600	0.88414000
H	-5.72040300	-0.53189600	1.81784700
C	-4.98445600	-0.98352900	-1.56820800
H	-4.19973100	-0.69667500	-2.27670300
H	-5.12382300	-2.06860100	-1.64930000
H	-5.91869900	-0.51261200	-1.90260000
C	-2.66232300	-2.98367800	-0.13992600
H	-2.92537100	-2.98407000	-1.20685400
C	-3.68763800	-3.88919400	0.57875100
H	-3.57557000	-4.92877200	0.24206400
H	-3.54096300	-3.88841700	1.66629100
H	-4.72243500	-3.59433300	0.38176500
C	-1.26129100	-3.62362900	-0.02704300
H	-1.02238600	-3.88037500	1.00928800
H	-1.23520900	-4.55823600	-0.60419200
H	-0.45902800	-2.98094800	-0.40242300

Zero-point correction = 0.784823 (Hartree/Particle)
Thermal correction to Energy = 0.830556
Thermal correction to Enthalpy = 0.831500
Thermal correction to Gibbs Free Energy = 0.706500
Sum of electronic and zero-point Energies = -4579.152233
Sum of electronic and thermal Energies = -4579.106500
Sum of electronic and thermal Enthalpies = -4579.105556
Sum of electronic and thermal Free Energies = -4579.230556
E(RM06L) = -4584.19916478

***trans*-2a**

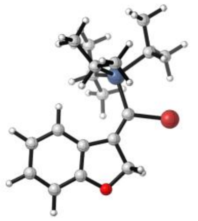

C	2.36022800	-0.25099400	0.00161700
C	2.23325000	2.11192400	0.00867900
C	3.63701200	0.34334300	-0.04648500
H	2.01898500	2.74902600	-0.85585100
H	2.09413400	2.71092200	0.91570700
O	3.61001200	1.69849100	-0.05905800
C	2.28584600	-1.64991000	0.04649200
C	4.81700200	-0.39480200	-0.07037700
H	5.77649800	0.10985500	-0.10970000
C	3.45662900	-2.40825600	0.02682700
C	4.71003800	-1.78509300	-0.03500000
H	1.33190000	-2.15537600	0.10509000
H	3.39245000	-3.49164100	0.06178100
H	5.61250900	-2.38997800	-0.05056300
C	1.37793900	0.84620800	0.01704100
C	0.02329500	0.85615200	0.03154100
Br	-0.72547200	2.67576400	0.12273700
Si	-1.29406900	-0.54657700	0.04325600
C	-3.04975300	0.24118400	-0.00763000
H	-3.00107200	1.06212300	0.72069400
C	-0.97996800	-1.44440100	1.72011400
H	0.11450600	-1.52753700	1.78081000
C	-0.98087500	-1.70976200	-1.46870600
H	-0.15307100	-2.37143700	-1.18060700
C	-0.52613600	-0.95668800	-2.73627100
H	0.39757000	-0.39384300	-2.57069900
H	-0.34245500	-1.66466700	-3.55606700
H	-1.28748000	-0.24852900	-3.08293600
C	-2.17307400	-2.63466500	-1.79920100
H	-3.03728500	-2.06941900	-2.16418300
H	-1.89248400	-3.34026000	-2.59308500
H	-2.50286600	-3.22670500	-0.93851200

C	-3.42654100	0.86595100	-1.36782400
H	-4.38285500	1.40086400	-1.28783500
H	-2.67903300	1.58317600	-1.71889300
H	-3.55089700	0.10127900	-2.14271300
C	-4.17861600	-0.70482200	0.45795200
H	-5.13802200	-0.16946900	0.45767400
H	-4.29517700	-1.56942900	-0.20442200
H	-4.02017400	-1.08301800	1.47269600
C	-1.41158100	-0.57939200	2.92272600
H	-2.49975800	-0.45721900	2.97146500
H	-1.09627000	-1.04581400	3.86595700
H	-0.96752400	0.42193100	2.88598700
C	-1.54247700	-2.87467600	1.82969400
H	-1.26693300	-3.32319600	2.79410700
H	-2.63623400	-2.89560500	1.76547900
H	-1.15390000	-3.53143600	1.04257000

Zero-point correction = 0.411507 (Hartree/Particle)
Thermal correction to Energy = 0.436077
Thermal correction to Enthalpy = 0.437021
Thermal correction to Gibbs Free Energy = 0.358015
Sum of electronic and zero-point Energies = -3637.878195
Sum of electronic and thermal Energies = -3637.853625
Sum of electronic and thermal Enthalpies = -3637.852681
Sum of electronic and thermal Free Energies = -3637.931687
E(RM06L) = -3641.14702517

i) reaction of aryl bromide **1b** (R = Mes), L = P*t*Bu_3

1b

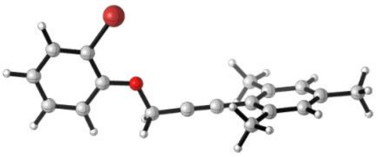

C	-3.83443000	0.33580500	0.00243200
C	-5.21753400	0.19825200	-0.00114400
C	-2.99404700	-0.79455500	-0.00499900
C	-5.79757900	-1.07315600	-0.01217000
H	-5.83546900	1.08963400	0.00474200
C	-3.58687200	-2.06487900	-0.01583200
C	-4.97781400	-2.19825400	-0.01938400
H	-6.87858300	-1.17389600	-0.01493100
H	-2.96993800	-2.95584800	-0.02145400
H	-5.41302700	-3.19360000	-0.02783800
O	-1.65428700	-0.56542600	-0.00103200
C	-0.77886700	-1.70049500	-0.01025100
H	-0.97635400	-2.32669500	0.87236900
H	-0.97683400	-2.31258600	-0.90261100
C	0.60194400	-1.23896600	-0.00704300
C	1.76092300	-0.88135300	-0.00497000
C	3.12359000	-0.45340100	-0.00261600
C	3.79071500	-0.22064000	-1.23062800
C	3.79704200	-0.25441900	1.22656200
C	5.11889900	0.20414200	-1.20153300
C	5.12631900	0.17096800	1.20152100
C	5.80703600	0.40304700	0.00156600
H	5.63164800	0.38782100	-2.14365800
H	5.64464400	0.32801500	2.14520800
Br	-3.06833700	2.08108700	0.01786200
C	3.07464600	-0.42276800	-2.54348300
H	2.72999300	-1.45804400	-2.65474200
H	2.18401800	0.21288800	-2.61403600
H	3.73000100	-0.18936400	-3.38806700
C	3.08898200	-0.49185200	2.53786200
H	2.19664500	0.13871800	2.62899500
H	2.74851000	-1.53082800	2.62520700
H	3.74814400	-0.27734700	3.38448400
C	7.25453100	0.83512800	0.00017400
H	7.53351900	1.30643000	0.94826300
H	7.92437000	-0.02276500	-0.14834000
H	7.45989200	1.54721100	-0.80687500

Zero-point correction = 0.297852 (Hartree/Particle)
Thermal correction to Energy = 0.318072
Thermal correction to Enthalpy = 0.319016
Thermal correction to Gibbs Free Energy = 0.244120
Sum of electronic and zero-point Energies = -3342.414718
Sum of electronic and thermal Energies = -3342.394498
Sum of electronic and thermal Enthalpies = -3342.393554
Sum of electronic and thermal Free Energies = -3342.468450
E(RM06L) = -3345.55818281

PC_1b

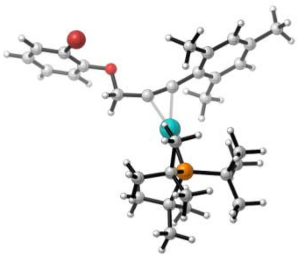

C	5.36137500	-1.17409500	-0.12245800
C	6.52253800	-1.91774600	0.08449100
C	4.62423900	-0.67393800	0.96155700
C	6.95921300	-2.17310300	1.38518100
H	7.07838600	-2.28849500	-0.76996200
C	5.07651000	-0.93741500	2.25947600
C	6.23662500	-1.68050900	2.47350300
H	7.86544500	-2.75091500	1.54191200
H	4.50547500	-0.53195900	3.08957100
H	6.57649600	-1.87081400	3.48753100
C	2.27519200	-0.63210300	0.64893100
C	1.16409600	0.30989600	0.46083800
C	0.64167600	1.43457500	0.33135700
Pd	-0.89190100	-0.19853400	0.21362400
P	-2.99383300	-1.28071600	-0.03859700
C	-3.27214500	-2.55302200	1.41095000
C	-4.43448900	0.03154400	0.00315600
C	-3.09081100	-2.24622500	-1.72975500
C	-4.39469000	-3.58955700	1.20516000
C	-1.93334100	-3.29376200	1.65002000
C	-3.55278000	-1.77866300	2.71784700
C	-5.84998200	-0.52863500	0.24919800
C	-4.10208400	1.07139400	1.10091600
C	-4.44451400	0.82189600	-1.32412300
C	-2.44728900	-1.35641800	-2.82132500
C	-2.19865100	-3.50497900	-1.64687300
C	-4.49850800	-2.67419000	-2.19115700
H	-4.47988600	-4.20931000	2.10854500
H	-5.37021500	-3.12654200	1.03526500
H	-4.19143800	-4.26764600	0.37243800
H	-1.11839100	-2.57965200	1.81258200
H	-2.03041700	-3.91768900	2.54917300
H	-1.64768000	-3.94988100	0.82752600
H	-4.54110300	-1.31378600	2.73708200
H	-3.51375600	-2.48600700	3.55686200
H	-2.79649800	-1.00779900	2.89837500
H	-5.95545800	-0.98128800	1.23848300
H	-6.14637600	-1.27045000	-0.49687000
H	-6.57423500	0.29597500	0.19609300
H	-4.85400500	1.87169600	1.06518200
H	-4.11402300	0.65698200	2.10920200

H	-3.11719000	1.51848700	0.93122900
H	-5.12370100	1.67744800	-1.21331600
H	-3.45286100	1.21707100	-1.56681600
H	-4.80626400	0.23353100	-2.17034600
H	-3.02418600	-0.45849600	-3.04420800
H	-2.37317700	-1.93759500	-3.75074200
H	-1.43991100	-1.04524300	-2.52567000
H	-1.19043000	-3.26261100	-1.29461500
H	-2.61641900	-4.28623100	-1.00773100
H	-2.10299600	-3.93100900	-2.65430000
H	-5.14524800	-1.82023800	-2.40890900
H	-5.00815200	-3.30815500	-1.46099800
H	-4.40995500	-3.25286000	-3.12105500
Br	4.78691200	-0.81573000	-1.90749800
C	0.26678400	2.81135600	0.19258300
C	-0.12093800	3.55885700	1.33264600
C	0.28665600	3.41960600	-1.08789700
C	-0.48175900	4.89654600	1.16668300
C	-0.08248400	4.76052900	-1.19860600
C	-0.46731800	5.51900200	-0.08680300
H	-0.78183600	5.47048700	2.04136200
H	-0.06873300	5.22780600	-2.18144800
C	-0.14525800	2.91893000	2.69809200
H	-0.78748600	2.02936000	2.70612600
H	0.85513900	2.58385700	2.99873700
H	-0.51117000	3.62048800	3.45437400
C	0.69598100	2.62981300	-2.30560400
H	1.72602800	2.26399500	-2.21623200
H	0.06093700	1.74409000	-2.43270000
H	0.62700100	3.24052000	-3.21128000
C	-0.82932600	6.97841900	-0.23224100
H	-1.53998900	7.29467900	0.53903300
H	0.05802300	7.61949600	-0.13837500
H	-1.27538000	7.18586200	-1.21108200
O	3.51263900	0.10761200	0.77952400
H	2.11702300	-1.23805300	1.55162700
H	2.34342200	-1.31418200	-0.20761200

Zero-point correction= 0.670480 (Hartree/Particle)
Thermal correction to Energy= 0.712638
Thermal correction to Enthalpy= 0.713582
Thermal correction to Gibbs Free Energy= 0.589261
Sum of electronic and zero-point Energies= -4283.715078
Sum of electronic and thermal Energies= -4283.672920
Sum of electronic and thermal Enthalpies= -4283.671976
Sum of electronic and thermal Free Energies= -4283.796297
E(RM06L) = -4288.62312280

TS_OA_1b

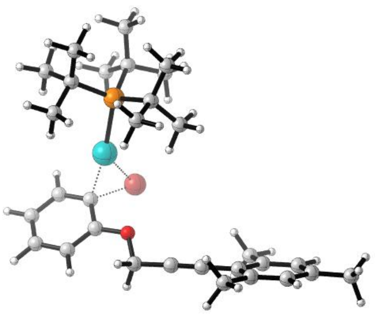

Pd	1.76413400	0.70091500	-0.15250600
C	1.08415700	2.64331600	-0.17738400
C	2.21960800	3.46840100	-0.15692300
C	-0.01630100	2.91904200	0.67833000
C	2.31814700	4.50797500	0.77536800
H	3.00584600	3.30246800	-0.88597200
C	0.09917800	3.96591700	1.59809800
C	1.25896300	4.75209100	1.64422700
H	3.20978700	5.12801800	0.80038800
H	-0.71007300	4.18161900	2.28701900
H	1.31671300	5.56403400	2.36376600
P	2.73071000	-1.47697100	0.16740200
C	4.08296700	-1.41419900	1.56805800
C	1.36269400	-2.76206800	0.68424300
C	3.56934700	-2.08858800	-1.47860000
Br	0.53938900	1.83981300	-2.17854800
O	-1.11490300	2.12468300	0.54159100
C	-2.26511700	2.43964100	1.33081300
H	-2.02515700	2.34240800	2.40047700
H	-2.56043900	3.48487500	1.15264800
C	4.31370900	-0.88576800	-2.10898600
H	4.69246600	-1.18603100	-3.09543200
H	3.63710500	-0.03643500	-2.24884800
H	5.16807700	-0.54641900	-1.52283300
C	2.47123500	-2.46978300	-2.49622800
H	1.95132400	-3.39552800	-2.23996200
H	1.73328500	-1.67001400	-2.61424400
H	2.94557100	-2.62806600	-3.47361000
C	4.54682400	-3.27326700	-1.34111300
H	4.91032100	-3.55466600	-2.33907500
H	5.42642200	-3.02393800	-0.74149200
H	4.07800000	-4.15887000	-0.90418200
C	0.10100600	-2.47606800	-0.16699000
H	0.23715000	-2.68287800	-1.22861800
H	-0.71639400	-3.11730400	0.19005900
H	-0.21593600	-1.43271400	-0.06748100
C	0.94157800	-2.50344800	2.14801200
H	0.67524000	-1.45450800	2.31535200
H	0.05001600	-3.10663700	2.36412600
H	1.70706200	-2.79132800	2.87229600
C	1.74438900	-4.24986600	0.54354800
H	0.91415100	-4.86797100	0.91204200
H	1.92127200	-4.54041100	-0.49522700
H	2.63139500	-4.51471700	1.12538400
C	3.53350000	-0.52808200	2.71296100
H	2.68303100	-0.96760100	3.23456400
H	4.32935300	-0.37424300	3.45452300
H	3.22761700	0.45457300	2.33637100
C	5.33006000	-0.67464900	1.03636300
H	6.00027300	-0.46691300	1.88092200
H	5.89947300	-1.26159900	0.31203300
H	5.06575700	0.28572900	0.58139800
C	4.52867000	-2.77194400	2.14719600
H	5.31586800	-2.60292300	2.89491800
H	3.71582500	-3.29906700	2.65342600
H	4.94163700	-3.43907800	1.38584000
C	-3.34972300	1.53518700	0.97317400
C	-4.27118000	0.80162200	0.68371900
C	-5.35174100	-0.06092600	0.32450300
C	-6.08796600	-0.72905600	1.33172900
C	-5.67488600	-0.24679200	-1.04220400
C	-7.13705000	-1.56766400	0.95176100
C	-6.73000100	-1.09918600	-1.36952300
C	-7.47710300	-1.76538600	-0.39096000
H	-7.70313400	-2.08325300	1.72501400
H	-6.97652600	-1.24780300	-2.41900600
C	-5.74227800	-0.53840600	2.78837500
H	-4.70034600	-0.81467400	2.99021900
H	-5.85250700	0.51006200	3.09173000
H	-6.38822900	-1.14676700	3.42879400
C	-4.88888300	0.45831000	-2.12010300
H	-4.94947100	1.54765500	-2.00883000
H	-3.82391100	0.20162700	-2.07435600
H	-5.26320100	0.19543000	-3.11416300
C	-8.63624700	-2.65464900	-0.77553000
H	-8.83814100	-3.40653800	-0.00530600
H	-9.55694400	-2.07072400	-0.91043900
H	-8.44516500	-3.17754800	-1.71913300

Zero-point correction = 0.670107 (Hartree/Particle)
Thermal correction to Energy = 0.711731
Thermal correction to Enthalpy = 0.712675
Thermal correction to Gibbs Free Energy = 0.591830
Sum of electronic and zero-point Energies = -4283.694656
Sum of electronic and thermal Energies = -4283.653032

Sum of electronic and thermal Enthalpies = -4283.652088
Sum of electronic and thermal Free Energies = -4283.772933
E(RM06L) = -4288.60467183

Ib

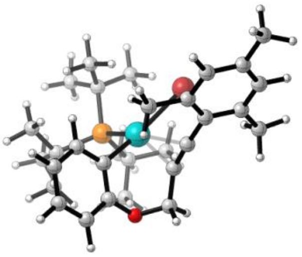

Pd	-0.07347300	0.09946800	0.07655200
C	-0.46548200	2.05992800	-0.19500300
C	-0.46548400	2.61058100	-1.48195000
C	-0.56139000	2.93576500	0.89297200
C	-0.61211900	3.99129100	-1.67452400
H	-0.36220300	1.96239200	-2.34899000
C	-0.71609500	4.31168000	0.71748900
C	-0.75609300	4.84002100	-0.57558700
H	-0.62049100	4.39685800	-2.68327800
H	-0.80636500	4.94901200	1.59288800
H	-0.88318500	5.90948700	-0.72008200
P	-2.44891400	-0.65203200	-0.17643900
C	-2.57010600	-2.12416900	-1.47531000
C	-2.92666600	-1.31090200	1.59736800
C	-3.81394300	0.66507500	-0.70731600
Br	1.14150300	-2.20513500	-0.03276900
C	1.41257000	1.09800000	1.48739200
C	2.44735300	0.74172500	0.90819200
O	-0.52933200	2.43005700	2.18497600
C	-1.72911100	-2.09156300	2.18327300
H	-0.83859900	-1.46349900	2.26458600
H	-2.00577400	-2.42788700	3.19188200
H	-1.45101800	-2.96914000	1.60108400
C	-3.17362900	-0.11660100	2.54596200
H	-3.23139800	-0.50426200	3.57168200
H	-2.36106500	0.61602500	2.51426200
H	-4.11864800	0.39466600	2.34664300
C	-4.16821500	-2.22904400	1.63655600
H	-4.02899900	-3.15069800	1.06897400
H	-4.34181200	-2.51955500	2.68134300
H	-5.07787600	-1.74223000	1.28284900
C	-3.66333900	2.01887300	0.03168100
H	-4.58868800	2.58674700	-0.13437700
H	-3.52281800	1.93227600	1.10711000
H	-2.84439500	2.60866300	-0.36935900
C	-3.65811700	1.00064600	-2.20874100
H	-2.64130200	1.32011600	-2.44741300
H	-3.93375800	0.18420800	-2.87694000
H	-4.32353500	1.84350500	-2.43759600
C	-5.26304400	0.18793500	-0.44347800
H	-5.48906500	-0.80433500	-0.83005200
H	-5.50404500	0.20614200	0.62266200
H	-5.94568700	0.89416400	-0.93453800
C	-3.99049700	-2.44405700	-1.99373000
H	-4.45712700	-1.63385600	-2.55479100
H	-3.90609700	-3.29691800	-2.67973100
H	-4.66801500	-2.74661200	-1.19048400
C	-1.66108100	-1.73430200	-2.66575800
H	-1.95494500	-0.79607700	-3.14213000
H	-0.61768800	-1.65741900	-2.34728000
H	-1.72263200	-2.52380700	-3.42668100
C	-2.01968700	-3.45017000	-0.90308600
H	-2.67027700	-3.88392500	-0.13995100
H	-1.97711400	-4.17036700	-1.73131000
H	-1.00918800	-3.34708700	-0.50934000
C	0.73725000	1.88225600	2.55518700
H	1.40449200	2.69961500	2.86694100
H	0.54658100	1.24129200	3.42149400
C	3.66275400	0.30219300	0.32843400
C	4.44312300	-0.67114600	1.01024500
C	4.11398000	0.86342100	-0.89489600
C	5.66235100	-1.04970800	0.45224500
C	5.33652300	0.43976000	-1.41100200
C	6.12411300	-0.51571700	-0.75702800
H	6.26779000	-1.78957300	0.97104000
H	5.68580200	0.86476900	-2.34951200
C	3.27734400	1.87532200	-1.63351700
H	2.33423300	1.42910300	-1.97126900
H	3.00707000	2.72481800	-0.99618400
H	3.80818600	2.25653300	-2.51102400
C	3.96966400	-1.27609900	2.30681900
H	3.84127000	-0.51381800	3.08552400
H	3.00126100	-1.76634500	2.15750900
H	4.68569500	-2.01719800	2.67445700
C	7.42882200	-0.98098400	-1.35661100
H	8.14760600	-1.26791100	-0.58168100
H	7.27285900	-1.86011700	-1.99641900
H	7.88599200	-0.20358900	-1.97790400

Zero-point correction = 0.672762 (Hartree/Particle)
Thermal correction to Energy = 0.713992
Thermal correction to Enthalpy = 0.714936
Thermal correction to Gibbs Free Energy = 0.600119
Sum of electronic and zero-point Energies = -4283.714835
Sum of electronic and thermal Energies = -4283.673606
Sum of electronic and thermal Enthalpies = -4283.672662
Sum of electronic and thermal Free Energies = -4283.787478
E(RM06L) = -4288.64112281

TS_AI_Ib

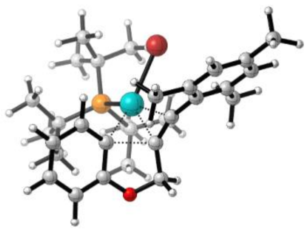

Pd	0.06162500	0.11377900	-0.13189700
C	-0.13151500	2.25630100	-0.18810400
C	-0.12403800	2.56704100	-1.55837400
C	-0.53488100	3.25156000	0.71261600
C	-0.63800900	3.78819700	-2.00756900
H	0.24990900	1.84674600	-2.28067200
C	-1.03628400	4.47767400	0.27894500
C	-1.11233800	4.73175300	-1.09146600
H	-0.65939300	4.00066500	-3.07284300
H	-1.33141400	5.21862500	1.01558200
H	-1.50296300	5.68351900	-1.44054500
P	-2.42285100	-0.72504400	-0.00078400
C	-2.77484500	-2.39872900	-0.96549700
C	-2.60084300	-1.08585500	1.90999200
C	-3.85608900	0.50783900	-0.51257400
Br	1.15421900	-2.02672900	-1.06545000
C	1.22236000	1.50450700	1.03675200
C	2.11695500	0.72490500	0.57052800
O	-0.34545800	3.03073200	2.05172800
C	-1.28861500	-1.74997200	2.39249000
H	-0.42450600	-1.10248600	2.21355600
H	-1.36348900	-1.92565900	3.47453000
H	-1.08371000	-2.70713800	1.91314800
C	-2.72374900	0.24271500	2.68854700
H	-2.61377000	0.02498000	3.75952500
H	-1.94613900	0.96306500	2.41872500
H	-3.69771800	0.72199900	2.56153300
C	-3.78426400	-1.98738900	2.32279300
H	-3.70534100	-2.99835200	1.91894100
H	-3.78512000	-2.07888100	3.41763800
H	-4.75420500	-1.57813900	2.03285900
C	-3.48594800	1.94666800	-0.09723300
H	-4.34359700	2.59783200	-0.31456000
H	-3.25185000	2.05757000	0.96034600
H	-2.64081000	2.31457900	-0.67475900
C	-3.97569600	0.55316800	-2.05325300
H	-3.00636100	0.73052900	-2.53034300

H	-4.41658400	-0.34749300	-2.48299200
H	-4.63055800	1.39290100	-2.32137100
C	-5.24342500	0.19192200	0.08844300
H	-5.59253500	-0.82033800	-0.11572000
H	-5.26619700	0.35141400	1.16953200
H	-5.97450900	0.88471400	-0.35037200
C	-4.26073700	-2.81375000	-1.04596000
H	-4.86764700	-2.13095300	-1.64386600
H	-4.30963500	-3.79409700	-1.53850200
H	-4.72793000	-2.92197200	-0.06408900
C	-2.22362000	-2.25337200	-2.40476500
H	-2.72522000	-1.47697600	-2.98358400
H	-1.14968100	-2.05667200	-2.39956700
H	-2.38538700	-3.20489900	-2.92956700
C	-2.01026500	-3.57571000	-0.31685800
H	-2.42411400	-3.86923200	0.65097300
H	-2.10969200	-4.44424700	-0.98155700
H	-0.94430300	-3.36817100	-0.21528700
C	0.84690700	2.26560000	2.26716600
H	1.65916000	2.94656400	2.56142000
H	0.65049800	1.57449700	3.09190300
C	3.43173100	0.20191800	0.35560100
C	3.90362100	-0.89115100	1.12656400
C	4.27184200	0.81352200	-0.61044200
C	5.21231900	-1.33002800	0.92797600
C	5.56962600	0.33156000	-0.77055900
C	6.06023100	-0.74056000	-0.01586400
H	5.58057000	-2.16050200	1.52646900
H	6.21526800	0.80190100	-1.50944500
C	3.01913400	-1.55808400	2.14768500
H	2.15967000	-2.02314900	1.65028300
H	3.56619500	-2.33312000	2.69336800
H	2.63071900	-0.83913600	2.88004100
C	3.76578000	1.94610100	-1.46844200
H	3.35672800	2.76600500	-0.86652100
H	4.56610400	2.34947500	-2.09620100
H	2.95856100	1.60361100	-2.12778300
C	7.45809100	-1.26634500	-0.23737200
H	7.86012000	-1.73273700	0.66832600
H	7.47037700	-2.02813800	-1.02865700
H	8.14390700	-0.46925500	-0.54454000

Zero-point correction = 0.671536 (Hartree/Particle)
Thermal correction to Energy = 0.712116
Thermal correction to Enthalpy = 0.713061
Thermal correction to Gibbs Free Energy = 0.600053
Sum of electronic and zero-point Energies = -4283.692306
Sum of electronic and thermal Energies = -4283.651725
Sum of electronic and thermal Enthalpies = -4283.650781
Sum of electronic and thermal Free Energies = -4283.763789
E(RM06L) = -4288.62239928

IIb

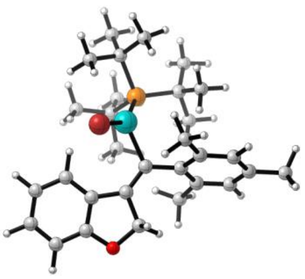

Pd	0.02329500	-0.54874700	0.87244200
C	-3.04376500	-0.76800100	-0.70248900
C	-2.66071200	-2.10608000	-0.56845900
C	-4.29853200	-0.47940500	-1.26796300
C	-3.52655200	-3.11503600	-0.99717100
H	-1.71371300	-2.35503600	-0.10324500
C	-5.17476900	-1.46738800	-1.70307300
C	-4.76886900	-2.79703800	-1.55973900
H	-3.23584600	-4.15552900	-0.88381700
H	-6.13522300	-1.20118200	-2.13201500
H	-5.43256400	-3.59348800	-1.88606000
P	2.06880500	-0.88870100	-0.40564200
C	1.79679100	-1.91950800	-2.03139600
C	3.25406200	0.60632200	-0.77952400
C	2.93752200	-2.03145300	0.92333000
Br	-1.53373000	-1.09304600	2.74749700
C	-2.38641300	0.51466900	-0.42460300
C	-1.16991800	0.86994700	0.03093300
O	-4.56213900	0.85363400	-1.36099500
C	-3.46377800	1.56207600	-0.75453500
H	-3.14203200	2.34041900	-1.44871300
H	-3.82990600	2.04313100	0.16130700
C	1.87316800	-2.97089200	1.54626000
H	1.41805200	-3.65656500	0.83272700
H	2.36175800	-3.57806400	2.32017800
H	1.06465000	-2.42616200	2.05293000
C	3.46125800	-1.16702900	2.09331300
H	3.77299800	-1.83929300	2.90277200
H	4.32965700	-0.56076100	1.82809600
H	2.68368000	-0.51063500	2.49767500
C	4.09724200	-2.89613600	0.38918200
H	4.53654100	-3.44830700	1.23095300
H	3.76369800	-3.63960200	-0.33893000
H	4.89643200	-2.30703500	-0.06563100
C	1.18325800	-3.29510800	-1.68800600
H	0.87726200	-3.77617900	-2.62579700
H	1.88942200	-3.96842000	-1.19777000
H	0.28907100	-3.20274000	-1.06476900
C	0.73698000	-1.18571800	-2.88480900
H	1.06446600	-0.20481100	-3.22917500
H	0.52474500	-1.79166300	-3.77544500
H	-0.19994100	-1.06388100	-2.33357400
C	3.06222900	-2.15663500	-2.88272600
H	3.48727000	-1.23144700	-3.27814500
H	3.84704600	-2.68607700	-2.33667600
H	2.79057300	-2.77817000	-3.74635300
C	4.74643800	0.23898100	-0.91741100
H	4.93175000	-0.50006100	-1.70082100
H	5.30254400	1.14666400	-1.18738100
H	5.17866400	-0.13349700	0.01356400
C	3.08815800	1.64708700	0.35288300
H	3.70489600	2.52269100	0.11036200
H	2.05297800	1.98362300	0.43933800
H	3.41164300	1.28232900	1.32768200
C	2.81393000	1.31250100	-2.07823900
H	2.99318200	0.71471700	-2.97424100
H	1.76282900	1.59940800	-2.04446300
H	3.39954400	2.23499700	-2.18110000
C	-0.65185800	2.25461900	0.03599700
C	-0.39842600	2.92364200	-1.19740100
C	-0.42325200	2.96882400	1.25110300
C	0.10064000	4.23226300	-1.18612400
C	0.07045600	4.27231000	1.20174000
C	0.35086500	4.92606900	-0.00369100
H	0.29049800	4.72353100	-2.13895400
H	0.23167500	4.80007000	2.13990400
C	-0.68897100	2.31441700	-2.55310700
H	0.06427800	2.62304500	-3.28656200
H	-0.72858300	1.22595600	-2.52565400
H	-1.65586600	2.66217800	-2.94142600
C	-0.71704300	2.36743200	2.60203400
H	-1.69382900	1.87904700	2.63079500
H	0.00805900	1.58919100	2.86667100
H	-0.68596600	3.13881000	3.37865000
C	0.88615100	6.33798900	-0.01300300
H	0.17931000	7.03459300	0.45552300
H	1.82606900	6.41306400	0.54811200
H	1.07450200	6.68921500	-1.03247000

Zero-point correction = 0.674500 (Hartree/Particle)
Thermal correction to Energy = 0.714934
Thermal correction to Enthalpy = 0.715878
Thermal correction to Gibbs Free Energy = 0.602696
Sum of electronic and zero-point Energies = -4283.761715
Sum of electronic and thermal Energies = -4283.721282
Sum of electronic and thermal Enthalpies = -4283.720338
Sum of electronic and thermal Free Energies = -4283.833520
E(RM06L) = -4288.69446378

TS_RE_IIb

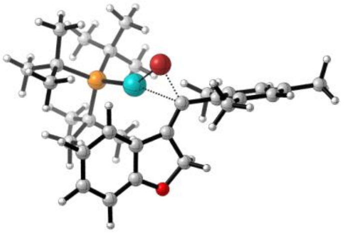

Pd	0.47250000	-0.31452500	-0.35591900
C	-2.14874400	0.34707600	-0.33362900
P	2.68836900	-0.73482400	0.08102600
C	3.06579200	-2.65359900	0.06638600
C	3.82342400	0.10099100	-1.27658700
C	3.23733300	-0.03537600	1.82182900
Br	-1.57626800	0.22456500	-2.21560400
C	-1.76815300	1.39918800	0.43110900
C	-0.99624600	2.62464800	0.20874800
C	-2.29431800	1.52421900	1.86368600
C	-1.01691000	3.34118400	1.42041100
H	-3.38592100	1.63667100	1.88860500
H	-2.01854500	0.67912400	2.49926000
O	-1.70116400	2.71423500	2.41611200
C	-0.34641800	3.18882500	-0.89823400
C	-0.39804700	4.57654300	1.57550000
H	-0.43424000	5.09418400	2.52822300
C	0.27345000	4.43163900	-0.76191400
C	0.25326400	5.11363000	0.46241100
H	-0.31856200	2.66302500	-1.84354000
H	0.77895000	4.87230200	-1.61609600
H	0.74523000	6.07868000	0.54908000
C	3.24874900	1.50700300	-1.56971300
H	3.79942800	1.94467800	-2.41437800
H	2.19058900	1.44099400	-1.84046300
H	3.33415000	2.19709800	-0.73035800
C	5.32144100	0.23510800	-0.93689500
H	5.79810700	-0.72474700	-0.72333200
H	5.84546300	0.67402800	-1.79756300
H	5.49744200	0.89829700	-0.08631500
C	3.68773500	-0.67997100	-2.60207500
H	2.63709800	-0.82674300	-2.87357100
H	4.15994500	-0.09458500	-3.40258800
H	4.18492900	-1.65241900	-2.58194700
C	2.26844500	-3.28438900	-1.10114700
H	2.61344900	-2.96242900	-2.08389000
H	2.37887700	-4.37695500	-1.05553300
H	1.20415800	-3.03903200	-1.02462000
C	4.54809500	-3.06031100	-0.06442100
H	4.62563300	-4.15513400	-0.00492500
H	4.98139900	-2.76374000	-1.02307000
H	5.17158900	-2.64718300	0.73251100
C	2.50019100	-3.30180000	1.34971100
H	1.45416100	-3.02557800	1.51476000
H	2.54036100	-4.39350900	1.23617700
H	3.07308400	-3.05317300	2.24619600
C	2.10676700	-0.34477500	2.83243700
H	1.96673500	-1.41092200	3.01344300
H	2.35381000	0.12374800	3.79527600
H	1.15452200	0.06641700	2.48218900
C	3.31325200	1.50622700	1.75223200
H	3.41774900	1.89679200	2.77348300
H	4.17342900	1.86741800	1.18362600
H	2.40251300	1.93772300	1.32500000
C	4.57150300	-0.56252300	2.38998000
H	4.78383400	-0.05038600	3.33904100
H	4.54115900	-1.63334200	2.60725300
H	5.41861900	-0.37652600	1.72491200
C	-3.23789400	-0.60472900	0.02079500
C	-4.55433000	-0.29020100	-0.41397200
C	-3.02068100	-1.77044800	0.79012500
C	-5.60481700	-1.14963600	-0.09117100
C	-4.10985500	-2.60358000	1.08033900
C	-5.40454300	-2.32068100	0.64772700
H	-6.60886400	-0.89676300	-0.42628600
H	-3.93213100	-3.50120300	1.66917000
C	-4.84849700	0.95749800	-1.21591900
H	-4.45288900	1.85786700	-0.73188000
H	-4.39320500	0.91169000	-2.21179700
H	-5.92710800	1.08819900	-1.34404200
C	-1.67036100	-2.14157100	1.35029800
H	-1.46370500	-1.60709500	2.28761400
H	-1.62523700	-3.21289400	1.57296500
H	-0.84348100	-1.89242900	0.66500300
C	-6.55420700	-3.25269700	0.94975700
H	-6.80358200	-3.86930500	0.07590400
H	-6.31342900	-3.93291400	1.77303000
H	-7.45983800	-2.69804300	1.22076400

Zero-point correction = 0.672527 (Hartree/Particle)
Thermal correction to Energy = 0.712726
Thermal correction to Enthalpy = 0.713670
Thermal correction to Gibbs Free Energy = 0.598479
Sum of electronic and zero-point Energies = -4283.742492
Sum of electronic and thermal Energies = -4283.702293
Sum of electronic and thermal Enthalpies = -4283.701349
Sum of electronic and thermal Free Energies = -4283.816540
E(RM06L) = -4288.66159476

***cis*-2b**

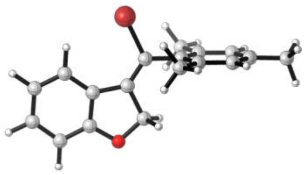

C	2.40773900	-0.30858000	-0.00000200
C	0.69943600	-1.95804300	0.00010300
C	2.93995600	-1.61162400	0.00010400
H	0.15278300	-2.29578600	0.88763600
H	0.15283800	-2.29589800	-0.88742200
O	1.99426800	-2.59002400	0.00018000
C	3.28700100	0.78202800	-0.00008300
C	4.30702400	-1.86559100	0.00013400
H	4.67818200	-2.88496200	0.00021700
C	4.66296700	0.54522500	-0.00005400
C	5.16460800	-0.76279700	0.00005400
H	2.90400100	1.79443900	-0.00016700
H	5.35024400	1.38606400	-0.00011500
H	6.23895800	-0.92642100	0.00007600
C	0.95056900	-0.44779400	0.00000700
C	-0.07905500	0.41540100	-0.00002400
C	-1.50876300	0.01870000	0.00002100
C	-2.18840900	-0.16828600	1.22436900
C	-2.18842300	-0.16861000	-1.22430000
C	-3.52954300	-0.56142800	1.19973300
C	-3.52952900	-0.56174400	-1.19959500
C	-4.21723200	-0.77028300	0.00011000
H	-4.05220000	-0.70243900	2.14378500
H	-4.05213100	-0.70297600	-2.14365500
Br	0.21212200	2.33296300	-0.00014500
C	-1.49324100	0.07029300	2.54545200
H	-0.62860200	-0.59098300	2.67888500
H	-1.11675300	1.09754800	2.61594400
H	-2.17749700	-0.09950700	3.38223300
C	-1.49323500	0.06961800	-2.54544300
H	-1.11634500	1.09671700	-2.61602600
H	-0.62886600	-0.59201400	-2.67889300
H	-2.17760700	-0.09997500	-3.38217100
C	-5.65770700	-1.22625600	0.00002700
H	-5.72662300	-2.32251900	-0.00869900
H	-6.18969800	-0.87514900	0.89064700
H	-6.19408200	-0.86104100	-0.88231900

Zero-point correction = 0.299655 (Hartree/Particle)
Thermal correction to Energy = 0.318828
Thermal correction to Enthalpy = 0.319772
Thermal correction to Gibbs Free Energy = 0.249698
Sum of electronic and zero-point Energies = -3342.468518
Sum of electronic and thermal Energies = -3342.449345
Sum of electronic and thermal Enthalpies = -3342.448401

Sum of electronic and thermal Free Energies = -3342.518475
E(RM06L) = -3345.61042622

TS_Isom_IIb

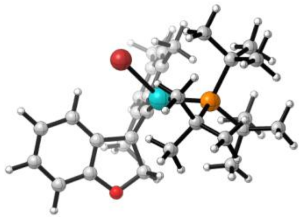

Pd	-0.15138600	-0.03318900	-0.28265300
C	2.26136400	-2.16962400	0.17074500
C	2.66598600	-2.40604800	-1.14677300
C	2.45881100	-3.15371400	1.15333400
C	3.27035000	-3.62828500	-1.44670700
H	2.46332500	-1.67320000	-1.91997000
C	3.05947800	-4.37421100	0.87054900
C	3.46439700	-4.59548800	-0.45038700
H	3.57956800	-3.83678900	-2.46643100
H	3.20026300	-5.11732500	1.64844400
H	3.93152600	-5.54249100	-0.70724700
P	-2.58053100	0.33281700	0.35971600
C	1.67073700	-1.00338200	0.83826700
C	1.64046100	0.30652700	0.38513800
C	2.54527300	1.40646400	0.23277100
C	2.20560400	2.54015900	-0.57689000
C	3.83158500	1.39352100	0.87854500
C	3.11433600	3.58849300	-0.71164100
C	4.69110800	2.47409400	0.70737500
C	4.36201800	3.58170300	-0.08406900
H	2.83970500	4.43776200	-1.33293800
H	5.65678900	2.45393000	1.20774200
C	-3.59445300	1.53720900	-0.79849600
C	-2.65621300	1.06555700	2.17165500
C	-3.45360400	-1.41048300	0.35720200
C	-3.22342100	1.24158600	-2.26949100
H	-2.14924900	1.32139700	-2.44685000
H	-3.52528800	0.24950700	-2.60196500
H	-3.73702400	1.97574900	-2.90554900
C	-3.16949900	3.00080500	-0.54358200
H	-3.62734300	3.62898000	-1.31856000
H	-3.50362400	3.38919000	0.42064600
H	-2.08640700	3.13483300	-0.61925400
C	-5.12878000	1.44952400	-0.65069200
H	-5.47622500	1.64862100	0.36549300
H	-5.58570100	2.20352700	-1.30614400
H	-5.52128700	0.47909300	-0.96223400
C	-3.76274300	-1.83583700	-1.09630500
H	-4.07100400	-2.88974200	-1.08196200
H	-4.58709700	-1.26998100	-1.53657400
H	-2.88506800	-1.75461900	-1.74447000
C	-4.75445100	-1.50627600	1.18104100
H	-5.17227800	-2.51447700	1.05662300
H	-4.59397500	-1.35887600	2.25197600
H	-5.51660300	-0.79683200	0.84987700
C	-2.43612200	-2.44712500	0.89240300
H	-1.52742900	-2.45868900	0.28233000
H	-2.15344800	-2.28101100	1.93348100
H	-2.89141600	-3.44471800	0.83409500
C	-2.22173200	-0.00560700	3.19449000
H	-1.25182600	-0.43915900	2.93915900
H	-2.11642000	0.47386200	4.17661300
H	-2.94587800	-0.81447100	3.30785300
C	-1.60472500	2.19631600	2.28052700
H	-1.82436600	3.05611800	1.64690200
H	-1.57896800	2.55451600	3.31863900
H	-0.60531800	1.83127100	2.02266400
C	-4.02824300	1.61258500	2.62004500
H	-3.95319500	1.93798000	3.66674900
H	-4.34724900	2.48102100	2.04006000
H	-4.81860800	0.85989200	2.56960000
C	4.29512900	0.26318100	1.76513200
H	3.70069300	0.19567200	2.68402900
H	4.23630200	-0.70770600	1.26516500
H	5.33494100	0.42219300	2.06516300
C	0.90008000	2.63000100	-1.31369000
H	0.79127100	1.82392700	-2.05069700
H	0.05096800	2.53577000	-0.62304900
H	0.81112400	3.58903200	-1.83486500
C	5.33854600	4.71503000	-0.27288400
H	4.83126800	5.63631100	-0.57647900
H	5.90023700	4.91807700	0.64558300
H	6.07290200	4.47279000	-1.05321600
C	1.39717600	-1.48607600	2.26874300
H	1.80874700	-0.81907200	3.03377800
H	0.32894200	-1.61913100	2.45928000
O	2.02719300	-2.77710700	2.39366500
Br	-0.36750900	-0.95728600	-2.70813300

Zero-point correction = 0.672788 (Hartree/Particle)
Thermal correction to Energy = 0.712696
Thermal correction to Enthalpy = 0.713640
Thermal correction to Gibbs Free Energy = 0.601349
Sum of electronic and zero-point Energies = -4283.741054
Sum of electronic and thermal Energies = -4283.701146
Sum of electronic and thermal Enthalpies = -4283.700202
Sum of electronic and thermal Free Energies = -4283.812493
E(RM06L) = -4288.67223167

IIIb

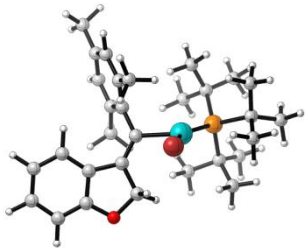

Pd	-0.58704700	-0.85879700	-0.70909900
C	3.51008900	-0.73459500	0.72525400
C	4.42603900	0.29271200	0.47484900
C	3.95301400	-1.90107800	1.37411800
C	5.75057800	0.14365000	0.89538300
H	4.11555700	1.19235800	-0.04558900
C	5.26654600	-2.06479900	1.80333600
C	6.16192800	-1.02041400	1.55684700
H	6.46704600	0.93676900	0.70178200
H	5.57217200	-2.97933900	2.30094900
H	7.19556500	-1.12038400	1.87745100
P	-2.42295800	0.09727600	0.57346300
C	-2.54809700	-0.66257000	2.35794000
C	-2.69352200	2.02103400	0.67136800
C	-3.85033200	-0.66077800	-0.53013700
Br	0.33252300	-2.49390100	-2.37371200
C	2.07991600	-0.92791700	0.41990200
C	1.21236500	-0.07023400	-0.14708700
O	2.98316000	-2.83841000	1.52885800
C	1.79330200	-2.36206900	0.85496700
H	1.59082300	-3.00976100	-0.00394400
H	0.95824100	-2.43999300	1.55906300
C	-3.43421300	-2.08514100	-0.97816800
H	-3.30279200	-2.78617500	-0.15543000
H	-4.22467100	-2.48534100	-1.62740000
H	-2.51459900	-2.09522100	-1.57896200
C	-3.98770700	0.15913300	-1.83359300
H	-4.65371900	-0.38691200	-2.51376100
H	-4.42741700	1.14586000	-1.67768400
H	-3.02701400	0.27866100	-2.34508800
C	-5.23207300	-0.75000300	0.14880300
H	-5.95708700	-1.12658100	-0.58527500
H	-5.23851000	-1.44890000	0.98881200
H	-5.59952200	0.21550500	0.50310700
C	-2.67493900	-2.19876300	2.25363000
H	-2.55133100	-2.62373900	3.25788200
H	-3.65097900	-2.52326600	1.88668100
H	-1.89661800	-2.63125700	1.61618500
C	-1.21312200	-0.39818700	3.09040200

H	-1.03300900	0.65714900	3.29416500
H	-1.23513900	-0.91911400	4.05643300
H	-0.36526200	-0.78591100	2.51970400
C	-3.70761200	-0.13256100	3.22767200
H	-3.61377900	0.93308700	3.44958900
H	-4.68868200	-0.30250600	2.77844000
H	-3.69348100	-0.66212500	4.18962700
C	-4.15858600	2.46309300	0.87172400
H	-4.60801400	2.04399600	1.77558900
H	-4.17922300	3.55634500	0.97393000
H	-4.79776600	2.21121700	0.02290700
C	-2.14432700	2.65233500	-0.62972700
H	-2.21786000	3.74432200	-0.54118900
H	-1.09304800	2.40230700	-0.78743000
H	-2.70192400	2.36136700	-1.52016400
C	-1.85843300	2.61180200	1.82669300
H	-2.24346500	2.34196000	2.81258900
H	-0.80872300	2.32333400	1.76235200
H	-1.89932800	3.70606000	1.75317600
C	1.42298200	1.35575800	-0.43961400
C	1.63864700	2.27489400	0.62906800
C	1.44777800	1.84903200	-1.77825200
C	1.82928300	3.63143900	0.34296800
C	1.64789000	3.21140200	-2.00419200
C	1.82885600	4.12695500	-0.96137900
H	1.99577200	4.31863600	1.17049300
H	1.67521200	3.56760800	-3.03223100
C	1.75319700	1.84136900	2.07260500
H	1.50352500	2.66794000	2.74656600
H	1.11340400	0.98993500	2.30607900
H	2.78092900	1.53207500	2.30031300
C	1.29166700	0.93691500	-2.96842400
H	1.92305900	0.04858700	-2.88771500
H	0.26560500	0.56182800	-3.06112200
H	1.54444900	1.46638600	-3.89288200
C	2.01805200	5.59782800	-1.24503100
H	2.73784400	5.75885100	-2.05617000
H	1.07561400	6.06796900	-1.55645600
H	2.37809300	6.13425100	-0.36125400

Zero-point correction = 0.674379 (Hartree/Particle)
Thermal correction to Energy = 0.714785
Thermal correction to Enthalpy = 0.715729
Thermal correction to Gibbs Free Energy = 0.603248
Sum of electronic and zero-point Energies = -4283.767515
Sum of electronic and thermal Energies = -4283.727109
Sum of electronic and thermal Enthalpies = -4283.726165
Sum of electronic and thermal Free Energies = -4283.838646
E(RM06L) = -4288.69809335

TS_RE_IIIb

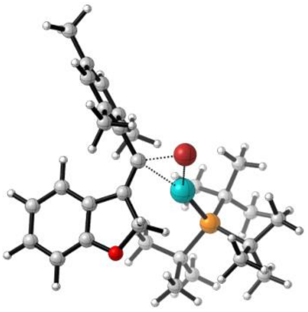

Pd	0.67814000	-0.47060900	-0.32142100
C	-1.93793900	-0.16134000	-0.80081400
P	2.88175200	-0.26894200	0.28358200
C	3.41521400	-1.70785100	1.49642000
C	4.05795800	-0.35134400	-1.27787100
C	3.22309900	1.43118700	1.19160300
Br	-1.22959700	-1.26653400	-2.28887100
C	-1.64672500	1.16203300	-0.83527700
C	-2.11464900	2.21898700	0.06535900
C	-0.87060300	1.89661500	-1.92543700
C	-1.53990900	3.42110400	-0.38695900
H	0.13085400	1.50544300	-2.10323600
H	-1.43264400	1.88384400	-2.87084000
O	-0.75522900	3.26733700	-1.48729500
C	-2.93909300	2.23777700	1.19748700
C	-1.75678000	4.63841400	0.24935800
H	-1.29799700	5.54689200	-0.12638700
C	-3.16756700	3.45213200	1.84689500
C	-2.58108800	4.63580700	1.37772100
H	-3.39612700	1.32466100	1.56199400
H	-3.80757100	3.47887300	2.72385800
H	-2.77070500	5.57110400	1.89747000
C	3.40292900	0.47331900	-2.41202900
H	4.00509800	0.36053000	-3.32434700
H	2.39247700	0.10617500	-2.61816400
H	3.33670900	1.53970500	-2.19454000
C	5.50521400	0.14076500	-1.07469500
H	6.03125500	-0.40280000	-0.28607900
H	6.06825500	-0.00953600	-2.00647200
H	5.55400900	1.20723000	-0.84227300
C	4.10356900	-1.80220800	-1.80687800
H	3.09673900	-2.21149800	-1.94073800
H	4.59563200	-1.80068300	-2.78883800
H	4.67496800	-2.47603100	-1.16451300
C	2.77738600	-3.02151100	0.98188300
H	3.19011000	-3.35745200	0.03028200
H	2.95964800	-3.81621500	1.71874700
H	1.69606700	-2.90814000	0.85435700
C	4.93047900	-1.93009200	1.67906000
H	5.08783700	-2.72800100	2.41841900
H	5.42085600	-2.25193300	0.75718600
H	5.44877000	-1.04096400	2.04684900
C	2.79457500	-1.46164200	2.89008400
H	1.72044900	-1.26077700	2.82749200
H	2.92758400	-2.36796900	3.49602000
H	3.27199200	-0.64236800	3.43284000
C	2.04687000	1.69729700	2.16108000
H	2.00043000	0.98948600	2.98924700
H	2.16466600	2.70091800	2.59255800
H	1.08917100	1.66017300	1.63223100
C	3.17254000	2.57895500	0.15841500
H	3.17707600	3.53547100	0.69806100
H	4.03294700	2.58812800	-0.51460700
H	2.25664300	2.54602800	-0.44065500
C	4.54801600	1.54132700	1.97353300
H	4.63342700	2.55455400	2.39066400
H	4.59660500	0.84693700	2.81606500
H	5.42712500	1.37440500	1.34602000
C	-2.99808900	-0.83801000	-0.00936400
C	-4.29968900	-0.90374700	-0.57323500
C	-2.76462000	-1.39580800	1.26895300
C	-5.31930400	-1.54402500	0.13306800
C	-3.82182200	-2.02780500	1.93566200
C	-5.10094000	-2.12501800	1.38608800
H	-6.31330900	-1.58639500	-0.30770800
H	-3.63352800	-2.45282300	2.91946700
C	-4.61417700	-0.27695000	-1.91230700
H	-4.39568200	0.79764800	-1.91178200
H	-4.02240600	-0.72450400	-2.71872100
H	-5.67244400	-0.40391800	-2.15896100
C	-1.43417300	-1.28447300	1.96833500
H	-1.25702700	-0.26141600	2.32281200
H	-1.39358800	-1.95084300	2.83635500
H	-0.58849100	-1.52850900	1.30515300
C	-6.21064600	-2.84844700	2.11170300
H	-6.28888100	-3.89079100	1.77439800
H	-6.03729000	-2.86835000	3.19273900
H	-7.18293000	-2.37688400	1.93099700

Zero-point correction = 0.672796 (Hartree/Particle)
Thermal correction to Energy = 0.712798
Thermal correction to Enthalpy = 0.713742
Thermal correction to Gibbs Free Energy = 0.599445
Sum of electronic and zero-point Energies = -4283.745011
Sum of electronic and thermal Energies = -4283.705009
Sum of electronic and thermal Enthalpies = -4283.704065
Sum of electronic and thermal Free Energies = -4283.818363
E(RM06L) = -4288.66390769

***trans*-2b**

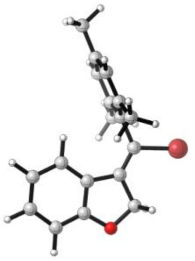

C	-1.87687400	-0.76090500	0.00002200
C	-2.48297900	1.53223800	-0.00014200
C	-3.27330200	-0.59521600	-0.00002900
H	-2.50692400	2.17495100	0.88716200
H	-2.50686900	2.17484800	-0.88752100
O	-3.66721400	0.70691000	-0.00013000
C	-1.33851200	-2.05300900	0.00011600
C	-4.15234000	-1.67316100	0.00002100
H	-5.22503200	-1.51110100	-0.00001400
C	-2.20750500	-3.14612900	0.00016700
C	-3.59599500	-2.95507600	0.00012200
H	-0.26453600	-2.20268200	0.00015700
H	-1.80239800	-4.15370800	0.00024800
H	-4.25607200	-3.81835600	0.00016800
C	-1.29374300	0.58428300	-0.00004300
C	-0.00270600	0.95092300	-0.00001400
C	1.19449300	0.08304500	0.00017500
C	1.76577400	-0.32838700	-1.22473700
C	1.76483000	-0.32896200	1.22492600
C	2.89076400	-1.15712900	-1.19987500
C	2.89010200	-1.15791200	1.20026500
C	3.47044600	-1.58154200	0.00042400
H	3.32667900	-1.47833300	-2.14387200
H	3.32540100	-1.47964400	2.14431500
Br	0.37804800	2.86447300	-0.00013600
C	1.17250200	0.10774600	-2.54431700
H	0.15252600	-0.27441000	-2.67170200
H	1.11534000	1.20025400	-2.61453700
H	1.77526300	-0.25588600	-3.38205300
C	1.17097500	0.10630000	2.54453200
H	1.11317500	1.19874400	2.61519500
H	0.15119500	-0.27651100	2.67154100
H	1.77373600	-0.25732500	3.38227100
C	4.70735900	-2.44927900	-0.00032600
H	4.76721600	-3.06376900	0.90422200
H	4.72839000	-3.11771000	-0.86789100
H	5.61920100	-1.83818100	-0.03963500

Zero-point correction = 0.299670 (Hartree/Particle)
Thermal correction to Energy = 0.318803
Thermal correction to Enthalpy = 0.319748
Thermal correction to Gibbs Free Energy = 0.249525
Sum of electronic and zero-point Energies = -3342.471491
Sum of electronic and thermal Energies = -3342.452358
Sum of electronic and thermal Enthalpies = -3342.451414
Sum of electronic and thermal Free Energies = -3342.521637
E(RM06L) = -3345.61426709

j) reaction of aryl chloride **1c** (R = TIPS), L = P*t*Bu$_3$

1c

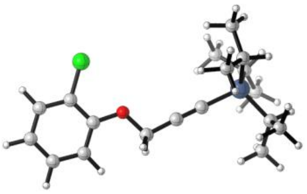

C	-4.44138900	0.60470900	0.00036800
C	-5.82184100	0.43789600	0.00318100
C	-3.57798400	-0.50934300	0.00123600
C	-6.37397300	-0.84577900	0.00698600
H	-6.45693200	1.31764400	0.00241900
C	-4.14326800	-1.79135900	0.00500700
C	-5.53126300	-1.95378600	0.00788500
H	-7.45249400	-0.96987600	0.00920500
H	-3.50759100	-2.66904800	0.00567300
H	-5.94572200	-2.95789600	0.01081600
O	-2.24357900	-0.24742900	-0.00172400
C	-1.34621000	-1.36041700	0.00106100
H	-1.52449200	-1.98315000	0.89047600
H	-1.52495600	-1.98820800	-0.88470000
C	0.02620400	-0.86716300	-0.00052400
C	1.18512100	-0.49490800	-0.00112800
Si	2.94704200	0.06842000	-0.00219500
C	3.25444100	1.14695500	1.56287300
H	4.33399300	1.04242400	1.76060100
C	4.06648300	-1.50012900	0.03513600
H	5.01886400	-1.17045100	-0.41192800
C	3.26307200	1.08543300	-1.60464200
H	4.13263300	1.71918000	-1.36477800
C	3.64487000	0.23796600	-2.83371700
H	2.81833800	-0.41423500	-3.14153100
H	3.87656900	0.88805400	-3.68842100
H	4.52173500	-0.39341700	-2.65433900
C	2.08548600	2.01486400	-1.96496600
H	1.82377300	2.70145000	-1.15438900
H	2.33153700	2.62277700	-2.84644700
H	1.18737800	1.43362100	-2.20297600
C	2.97081300	2.64991900	1.37520500
H	3.23625900	3.20432600	2.28570600
H	3.54113700	3.08609700	0.54799500
H	1.90685400	2.83533900	1.18455700
C	2.49592000	0.63008200	2.80223500
H	2.75835300	1.22296300	3.68912000
H	1.41269700	0.71122800	2.65728900
H	2.71901900	-0.41724600	3.02778000
C	4.38488300	-2.02409300	1.44876900
H	5.07328400	-2.87851400	1.39388100
H	4.85501100	-1.26564400	2.08386400
H	3.47880500	-2.37301300	1.95901900
C	3.51603200	-2.65237200	-0.82935200
H	4.21971800	-3.49609100	-0.84056200
H	2.56439400	-3.02047600	-0.42871000
H	3.34061400	-2.35511300	-1.86755900
Cl	-3.76430000	2.21988200	-0.00419000

Zero-point correction= 0.410093 (Hartree/Particle)
Thermal correction to Energy= 0.435520
Thermal correction to Enthalpy= 0.436465
Thermal correction to Gibbs Free Energy= 0.352621
Sum of electronic and zero-point Energies= -1526.623063
Sum of electronic and thermal Energies= -1526.597635
Sum of electronic and thermal Enthalpies= -1526.596691
Sum of electronic and thermal Free Energies= -1526.680534
E(RM06L) = -1527.24423854

PC_1c

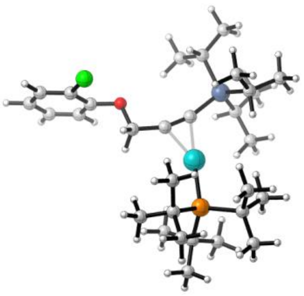

C	4.69055100	-2.84590800	-0.61308900
C	5.56037000	-3.91760800	-0.41071400
C	4.21726400	-2.09031500	0.47164300
C	5.96406900	-4.24728800	0.88362000
H	5.91662100	-4.48161900	-1.26646100
C	4.63292000	-2.43313400	1.76261500
C	5.50225700	-3.50307100	1.97096900
H	6.64300800	-5.08109500	1.03655200
H	4.27018100	-1.83089100	2.59035300
H	5.81975700	-3.75219900	2.97941000
C	1.99297900	-1.30514700	0.22428900
C	1.23299600	-0.05093500	0.13704300
C	1.01447500	1.18051200	0.07668900
Si	1.23696300	3.02548800	0.02237300
C	3.08762800	3.28588100	-0.42642300
H	3.23175400	2.64663700	-1.31087800
C	0.04027200	3.66569300	-1.33396000
H	-0.91699400	3.18364100	-1.08145100
C	0.78613100	3.65050500	1.78288900
H	1.33704100	2.96887700	2.44943600
C	3.47584500	4.72105300	-0.83412500
H	3.33104600	5.43741400	-0.01707600
H	4.53743700	4.76418300	-1.11502300
H	2.89791500	5.07895900	-1.69331700
C	4.02743500	2.74974500	0.67226900
H	3.83382500	1.69465500	0.89189100
H	5.07650700	2.83406100	0.35628400
H	3.92606600	3.31770600	1.60621800
C	0.44493100	3.15875000	-2.73251000
H	-0.31260600	3.43201700	-3.48023000
H	1.39577200	3.59633300	-3.06205400
H	0.55384900	2.06844600	-2.75232400
C	-0.20133500	5.18756000	-1.34962100
H	0.71547100	5.74896200	-1.56463100
H	-0.92999100	5.45302900	-2.12839000
H	-0.59629700	5.55447300	-0.39583600
C	-0.71319100	3.47298300	2.09259700
H	-1.04623900	2.44434500	1.90711500
H	-0.92381900	3.71065400	3.14481100
H	-1.33370700	4.14010000	1.48053100
C	1.24718900	5.08351500	2.11525300
H	0.77979800	5.82970400	1.46183500
H	0.97464100	5.34441200	3.14748900
H	2.33262100	5.19994700	2.02628600
Pd	-0.89988700	0.07012600	0.06413200
P	-2.99181900	-1.05742700	0.01752400
C	-3.46361500	-1.74006800	1.78210100
C	-4.37710900	0.19047400	-0.55056200
C	-2.96289700	-2.55367900	-1.23264500
C	-4.60632500	-2.77389200	1.83239800
C	-2.18929500	-2.36062500	2.40559900
C	-3.83341700	-0.55429200	2.70054000
C	-5.83336200	-0.22955300	-0.26744300
C	-4.09627200	1.54718000	0.14062900
C	-4.23356100	0.46142200	-2.06489800
C	-2.18733500	-2.10537400	-2.49521500
C	-2.13582700	-3.70823200	-0.62494300
C	-4.33804200	-3.10927400	-1.65454300
H	-4.80778200	-3.03449900	2.88068000
H	-5.53878100	-2.39387000	1.40696200
H	-4.35238300	-3.70382300	1.31703700
H	-1.36286000	-1.64230500	2.39321400
H	-2.40193100	-2.62757000	3.44991500
H	-1.85692300	-3.26744500	1.89943200
H	-4.79590300	-0.10174100	2.45159800
H	-3.90915300	-0.92299300	3.73199900
H	-3.06262900	0.22325800	2.68431000
H	-6.04903400	-0.30325000	0.80142600
H	-6.09442100	-1.18349800	-0.73298200
H	-6.51123300	0.53119000	-0.67879700
H	-4.79989200	2.29551800	-0.24934400
H	-4.21723400	1.51791000	1.22350000
H	-3.07916300	1.89413700	-0.07444300
H	-4.88213200	1.30710700	-2.32945100
H	-3.20771400	0.73428800	-2.33256500
H	-4.54367800	-0.38454100	-2.68231900
H	-2.71184800	-1.34873700	-3.07908000
H	-2.04050300	-2.97791500	-3.14636600
H	-1.20525000	-1.70155600	-2.22864800
H	-1.15726900	-3.36382800	-0.27483300
H	-2.64537900	-4.21313800	0.19883000
H	-1.96250400	-4.46074000	-1.40540200
H	-4.92950500	-2.38080100	-2.21483200
H	-4.93527800	-3.44985300	-0.80485500
H	-4.18679600	-3.97394500	-2.31545200
O	3.40608900	-0.99911000	0.28949100
Cl	4.20431800	-2.42688300	-2.24568000
H	1.70056800	-1.86669700	1.12195400
H	1.79106700	-1.93601400	-0.65025100

Zero-point correction= 0.781881 (Hartree/Particle)
Thermal correction to Energy= 0.829629
Thermal correction to Enthalpy= 0.830573
Thermal correction to Gibbs Free Energy= 0.696080
Sum of electronic and zero-point Energies= -2467.928027
Sum of electronic and thermal Energies= -2467.880279
Sum of electronic and thermal Enthalpies= -2467.879335
Sum of electronic and thermal Free Energies= -2468.013828
E(RM06L) = -2470.31711709

TS_OA_1c

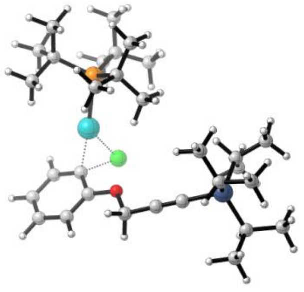

Pd	-2.24573700	0.92602000	0.30410600
C	-1.66816300	2.87233100	0.39936200
C	-2.79697400	3.70074100	0.37649900
C	-0.50478400	3.21593300	-0.33783500
C	-2.82375000	4.82529600	-0.45709700
H	-3.63414200	3.47025500	1.02770600
C	-0.54681300	4.34934600	-1.15489500
C	-1.70002400	5.14487400	-1.21334300
H	-3.71058100	5.45175200	-0.49068900
H	0.31417900	4.62601700	-1.75343800
H	-1.70107200	6.02471400	-1.85056300
P	-2.95097900	-1.34548500	-0.12417500

C	-4.53404900	-1.39980800	-1.25425300
C	-1.50296800	-2.27698200	-1.03213600
C	-3.34144500	-2.26552100	1.54606900
O	0.57511700	2.39310200	-0.20143200
C	1.76283300	2.73741400	-0.91456900
H	1.55915200	2.76668200	-1.99604000
H	2.10322600	3.74015700	-0.61522700
C	-4.14308300	-1.29440200	2.44711600
H	-4.27259900	-1.75982300	3.43356500
H	-3.60573800	-0.35085100	2.58659100
H	-5.13768500	-1.06647400	2.06311300
C	-2.01885200	-2.53030700	2.29974600
H	-1.41025300	-3.30987600	1.83576400
H	-1.41743200	-1.62078700	2.39652400
H	-2.26205700	-2.87357800	3.31381200
C	-4.11108400	-3.59562000	1.41980900
H	-4.22154500	-4.04031400	2.41834500
H	-5.11904700	-3.46073600	1.01863800
H	-3.59166100	-4.32611400	0.79395300
C	-0.17220900	-1.83921800	-0.37091400
H	-0.07072800	-2.17248800	0.66183400
H	0.66112600	-2.27386600	-0.94012700
H	-0.05823000	-0.74982000	-0.38287200
C	-1.41569600	-1.78974000	-2.49566700
H	-1.37584200	-0.69712300	-2.55847800
H	-0.48759600	-2.17782200	-2.93536800
H	-2.23892800	-2.14750400	-3.11813100
C	-1.58697700	-3.81663200	-1.03587600
H	-0.74609700	-4.21957400	-1.61704700
H	-1.51276300	-4.24177800	-0.03184700
H	-2.50742900	-4.18769600	-1.49458500
C	-4.37666100	-0.30675300	-2.33969100
H	-3.57189400	-0.50795800	-3.04691100
H	-5.31029400	-0.24267000	-2.91497100
H	-4.19128300	0.67398000	-1.88691500
C	-5.77007200	-0.98331000	-0.42628400
H	-6.61408100	-0.83348400	-1.11238500
H	-6.07613400	-1.74186900	0.29739500
H	-5.60475100	-0.03928700	0.10334000
C	-4.83491400	-2.75102800	-1.93350300
H	-5.77052000	-2.66646400	-2.50335500
H	-4.05615800	-3.04708200	-2.64087900
H	-4.96459600	-3.56278300	-1.21298800
C	2.79475500	1.74661200	-0.62815500
C	3.67632600	0.93533200	-0.41238200
Si	4.99985600	-0.31026000	-0.05458700
C	4.23804800	-1.47959800	1.26055900
H	3.25592700	-1.74493000	0.83976900
C	5.34677700	-1.17123000	-1.73467100
H	5.47956300	-0.33372700	-2.43702800
C	6.48832500	0.70891900	0.59814300
H	6.04437800	1.35027400	1.37490200
C	5.00390600	-2.79565900	1.49983900
H	6.01375600	-2.62096800	1.88906800
H	4.47768500	-3.41541500	2.23889800
H	5.10090800	-3.39152000	0.58556100
C	3.97678500	-0.74301900	2.58983700
H	3.37315300	0.15949800	2.44431600
H	3.43773600	-1.39217900	3.29303400
H	4.91359300	-0.44592900	3.07820900
C	4.12628900	-1.97990100	-2.21846200
H	4.28863200	-2.35910700	-3.23678500
H	3.93946900	-2.84989100	-1.57612200
H	3.21393300	-1.37262600	-2.23045900
C	6.62927700	-2.02379000	-1.79608400
H	6.59829400	-2.86687800	-1.09610300
H	6.76231100	-2.44546800	-2.80202600
H	7.52630200	-1.43799400	-1.56796600
C	7.05893500	1.64089400	-0.48955600
H	6.28179200	2.27341600	-0.93363000
H	7.82754100	2.30423300	-0.06994800
H	7.53100300	1.07333700	-1.30153200
C	7.61155700	-0.10645400	1.26832100
H	8.08816900	-0.80503500	0.57066900
H	8.39917100	0.56188300	1.64280400
H	7.24658000	-0.68851000	2.12144200
Cl	-1.25991400	2.02492000	2.34206100

Zero-point correction= 0.781031 (Hartree/Particle)
Thermal correction to Energy= 0.828384
Thermal correction to Enthalpy= 0.829329
Thermal correction to Gibbs Free Energy= 0.697760
Sum of electronic and zero-point Energies= -2467.892817
Sum of electronic and thermal Energies= -2467.845463
Sum of electronic and thermal Enthalpies= -2467.844519
Sum of electronic and thermal Free Energies= -2467.976087
E(RM06L) = -2470.28339551

Ic

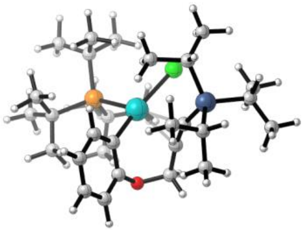

Pd	-0.19071200	-0.17229000	0.15462200
C	-0.12162400	1.81484400	-0.19747200
C	0.02501600	2.33082100	-1.48983500
C	-0.00953000	2.70271700	0.88120300
C	0.21383000	3.70425400	-1.69941700
H	-0.01847100	1.66502500	-2.34795700
C	0.16808600	4.07344900	0.68704600
C	0.26612000	4.57796600	-0.61213200
H	0.31189600	4.08624800	-2.71258900
H	0.22659400	4.72625000	1.55361700
H	0.39926100	5.64469000	-0.77003100
P	-2.67884200	-0.37493600	-0.04800500
C	-3.11551200	-1.92377500	-1.18098000
C	-3.33059000	-0.70078700	1.76248300
C	-3.68526300	1.15286400	-0.77693200
C	1.51241400	0.53455500	1.53032900
C	2.22045100	-0.00233700	0.66979500
O	-0.10999600	2.22971800	2.18543900
C	-2.35344900	-1.65351600	2.48601100
H	-1.34988700	-1.22671100	2.55358800
H	-2.72927200	-1.81212900	3.50600100
H	-2.25348700	-2.62673800	2.00840900
C	-3.33142800	0.61725400	2.56908200
H	-3.50248200	0.36591400	3.62417400
H	-2.37618900	1.14743300	2.50645600
H	-4.13510000	1.29484500	2.27090000
C	-4.74946800	-1.30839100	1.83248000
H	-4.80920800	-2.29775500	1.37651900
H	-5.01617600	-1.42621700	2.89124900
H	-5.51150700	-0.67471400	1.37663500
C	-3.24653000	2.50716600	-0.16527000
H	-3.99431400	3.25616300	-0.45879200
H	-3.19191000	2.51076400	0.92113100
H	-2.28740500	2.83546200	-0.55351500
C	-3.40121300	1.27293900	-2.29154200
H	-2.32873000	1.31810500	-2.49636000

H	-3.83765000	0.47077900	-2.88801000
H	-3.83921700	2.21493200	-2.64677100
C	-5.21381800	1.05707700	-0.54994000
H	-5.66050800	0.11105700	-0.85049000
H	-5.47703800	1.24555800	0.49439300
H	-5.69227800	1.84711000	-1.14386300
C	-4.56215500	-1.96100200	-1.72160400
H	-4.81175400	-1.13421100	-2.38743200
H	-4.67192700	-2.88370000	-2.30603200
H	-5.30566600	-2.00089300	-0.92073700
C	-2.12525200	-1.90222800	-2.36920400
H	-2.18064800	-0.98664400	-2.96226900
H	-1.09842800	-2.03472500	-2.01946900
H	-2.36295900	-2.74144200	-3.03635000
C	-2.89926700	-3.26478500	-0.44277800
H	-3.64479700	-3.44538800	0.33483900
H	-3.01384200	-4.06679000	-1.18451400
H	-1.89975400	-3.34906600	-0.01917500
C	0.96063000	1.37357500	2.60966500
H	1.77776300	1.98824400	3.01690700
H	0.55509600	0.75780900	3.41791800
Si	3.73427700	-0.54855800	-0.29637100
C	4.75586400	-1.71757000	0.84055000
C	3.34177500	-1.41038100	-1.96556000
C	4.72242700	1.07481900	-0.64217200
H	5.39139500	-2.26844600	0.12709000
C	5.70019100	-1.00521300	1.82799000
C	3.89292800	-2.74879800	1.59436100
H	4.23311600	-1.16570600	-2.56867100
C	3.23869800	-2.94741800	-1.91335500
C	2.11657900	-0.82854500	-2.69653700
H	5.76295700	0.72461600	-0.74894900
C	4.34637000	1.79444900	-1.95216000
C	4.68785800	2.08678000	0.52062800
H	6.30129500	-1.74344400	2.37615800
H	6.39501600	-0.31930800	1.33109100
H	5.13893900	-0.43186000	2.57630000
H	4.53530800	-3.47642200	2.10957600
H	3.27240700	-2.25703200	2.35182600
H	3.21001000	-3.29625600	0.94071100
H	3.14490000	-3.34895600	-2.93201800
H	4.12241900	-3.41112700	-1.46155800
H	2.35707900	-3.26301600	-1.34739500
H	2.02473600	-1.26949700	-3.69903100
H	1.19791900	-1.06334000	-2.14756700
H	2.16925500	0.25809000	-2.81499100
H	3.31740600	2.17287500	-1.91930700
H	5.00366300	2.66035400	-2.11145600
H	4.44040700	1.14728400	-2.83034200
H	4.98652800	1.64695600	1.47701300
H	5.36587200	2.92736100	0.31865500
H	3.68067600	2.50164000	0.64282800
Cl	0.29937300	-2.58337000	0.44626000

Zero-point correction= 0.784974 (Hartree/Particle)
Thermal correction to Energy= 0.831448
Thermal correction to Enthalpy= 0.832392
Thermal correction to Gibbs Free Energy= 0.708926
Sum of electronic and zero-point Energies= -2467.916756
Sum of electronic and thermal Energies= -2467.870281
Sum of electronic and thermal Enthalpies= -2467.869337
Sum of electronic and thermal Free Energies= -2467.992803
E(RM06L) = -2470.33231534

TS_AI_Ic

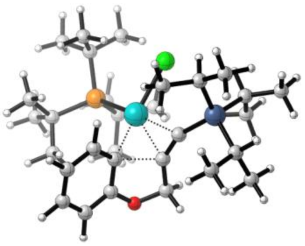

Pd	-0.13118300	-0.05618300	-0.17229100
C	-0.08435700	2.10083600	-0.18427600
C	-0.11688700	2.45918900	-1.54431100
C	-0.35102200	3.10004200	0.76379600
C	-0.54094200	3.73483200	-1.92869700
H	0.16618000	1.73952000	-2.30655000
C	-0.76094900	4.37987400	0.39579100
C	-0.88331100	4.68506200	-0.96086400
H	-0.59334100	3.98667100	-2.98420400
H	-0.94445800	5.12051000	1.16805600
H	-1.20137500	5.67944200	-1.26128800
P	-2.67829900	-0.61340600	-0.00113300
C	-3.19969500	-2.20211400	-1.03228200
C	-2.89523500	-1.01603000	1.89442200
C	-3.96954900	0.78451800	-0.47136400
C	1.27912800	1.22462100	0.89032700
C	1.98271100	0.32800700	0.30929100
O	-0.08615600	2.81853400	2.07823800
C	-1.67247000	-1.84731500	2.34968200
H	-0.73792900	-1.30058900	2.19106800
H	-1.77089500	-2.04960800	3.42503100
H	-1.58018400	-2.80394600	1.83652300
C	-2.86097100	0.29114800	2.71743300
H	-2.77432500	0.02587600	3.77954600
H	-2.00458900	0.92498700	2.46726100
H	-3.77413900	0.88203300	2.61149800
C	-4.18152400	-1.78145700	2.27282700
H	-4.22438200	-2.77888000	1.83167800
H	-4.20052600	-1.91201100	3.36348100
H	-5.09152200	-1.24626500	1.99510400
C	-3.46824400	2.16136200	0.01196900
H	-4.25464800	2.90071100	-0.19253200
H	-3.24973500	2.20622400	1.07776900
H	-2.58118700	2.47026500	-0.53480500
C	-4.05480800	0.90887600	-2.01044200
H	-3.06372100	1.01821600	-2.46254100
H	-4.56569200	0.07360300	-2.49104100
H	-4.62430600	1.81667800	-2.24969900
C	-5.39106500	0.58970000	0.10238100
H	-5.83573200	-0.37784100	-0.12879700
H	-5.41331900	0.72691800	1.18681500
H	-6.04386100	1.36147500	-0.32794700
C	-4.72169000	-2.42254700	-1.18052400
H	-5.22415800	-1.63992700	-1.75135400
H	-4.87209500	-3.36153400	-1.72968000
H	-5.22921200	-2.52901100	-0.21801800
C	-2.57238700	-2.06875100	-2.44124200
H	-2.93846200	-1.20297800	-2.99560500
H	-1.48320300	-2.01846600	-2.38343500
H	-2.83631700	-2.96266900	-3.02252500
C	-2.62779600	-3.49629300	-0.40799600
H	-3.12129800	-3.76795800	0.52820500
H	-2.81639400	-4.31527100	-1.11513500
H	-1.55103500	-3.44132200	-0.25354100
C	1.05901100	1.96163700	2.17436400
H	1.93843300	2.57226000	2.42536800
H	0.87866100	1.25202000	2.98628700

Si	3.59134000	-0.55093700	-0.09259300
C	4.96714200	0.71134100	0.39051500
C	3.69290300	-0.97632000	-1.96417400
C	3.70616700	-2.16528800	0.94422100
H	5.90745600	0.20914700	0.11240700
C	4.89262600	2.02774400	-0.40758800
C	5.04004200	1.01371100	1.89935200
H	3.20598600	-1.95747800	-2.03470900
C	2.90289000	-0.02681600	-2.88415800
C	5.14467200	-1.13112500	-2.46399200
H	3.14223500	-2.89057300	0.34337600
C	3.01098200	-2.09617700	2.31690000
C	5.15153700	-2.68491500	1.08749300
H	5.71670900	2.69910400	-0.12819300
H	4.95573900	1.86446900	-1.48840100
H	3.95482500	2.56224500	-0.21100600
H	5.86975900	1.70027900	2.11834100
H	4.12207000	1.49675200	2.25759000
H	5.19243200	0.11201600	2.50107200
H	3.26960100	1.00592700	-2.83333600
H	2.98814200	-0.35146200	-3.93064700
H	1.84020700	-0.02651500	-2.62367600
H	5.15235400	-1.48341500	-3.50444100
H	5.69040500	-0.17979700	-2.44321500
H	5.72088500	-1.85367700	-1.87466300
H	1.95141700	-1.84356800	2.21091600
H	3.07050500	-3.06948700	2.82347900
H	3.47451900	-1.35773300	2.98313100
H	5.77975000	-2.00290700	1.67390000
H	5.15843300	-3.65366000	1.60560300
H	5.64205700	-2.83406700	0.11884400
Cl	0.56301300	-2.32520400	-0.78598400

Zero-point correction= 0.782666 (Hartree/Particle)
Thermal correction to Energy= 0.828993
Thermal correction to Enthalpy= 0.829937
Thermal correction to Gibbs Free Energy= 0.705920
Sum of electronic and zero-point Energies= -2467.900198
Sum of electronic and thermal Energies= -2467.853871
Sum of electronic and thermal Enthalpies= -2467.852927
Sum of electronic and thermal Free Energies= -2467.976945
E(RM06L) = -2470.31541905

IIc

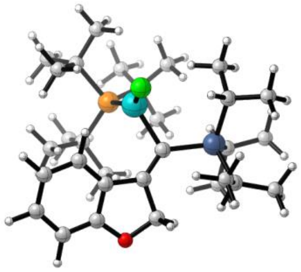

Pd	0.27826100	-0.16803400	-1.13959100
C	-0.21742400	2.91292400	-0.01375700
C	0.61690600	3.12652200	-1.11632800
C	-0.31314100	3.91551600	0.96666500
C	1.36770100	4.30556200	-1.18360500
H	0.64898900	2.40390700	-1.92329200
C	0.43951200	5.08317900	0.92962000
C	1.29219500	5.26243600	-0.16441400
H	2.00523100	4.48409500	-2.04487900
H	0.34253800	5.82774100	1.71297200
H	1.88444100	6.17120900	-0.23296800
P	2.21238000	-0.88678300	0.27119900
C	2.11387700	-2.65338900	1.10033600
C	3.50391300	-0.98727800	-1.19782100
C	2.87918800	0.36140200	1.60561600
C	-1.14755100	1.82710200	0.37019900
C	-1.24933100	0.53494700	-0.00992400
O	-1.22077100	3.62541500	1.94093800
C	-1.99657000	2.50378600	1.46641200
H	-2.21178900	1.86820400	2.32514000
H	-2.93468000	2.90342900	1.06022700
C	1.69761700	0.75302000	2.52473000
H	1.33972500	-0.07093100	3.14263300
H	2.03398700	1.54677100	3.20438800
H	0.85526700	1.14144400	1.94926500
C	3.34217800	1.66273400	0.91766200
H	3.54825600	2.40701000	1.69745900
H	4.26530400	1.53651500	0.34705900
H	2.57397400	2.08354600	0.26574000
C	4.04292000	-0.15695200	2.47662400
H	4.35934100	0.65355300	3.14655200
H	3.75752100	-0.99924200	3.11001300
H	4.91575100	-0.45160700	1.88928700
C	1.42564600	-2.54830100	2.47936500
H	1.19575100	-3.56380300	2.82685900
H	2.05729800	-2.08575600	3.24004000
H	0.48250700	-1.99937700	2.42939600
C	1.20151900	-3.55368000	0.23732700
H	1.58841300	-3.73516400	-0.76554000
H	1.10682700	-4.52939100	0.73240900
H	0.19985000	-3.13233500	0.13865200
C	3.46779400	-3.36870200	1.29505100
H	3.95078700	-3.62168500	0.34848200
H	4.17396400	-2.79259800	1.89699800
H	3.28692500	-4.31473800	1.82304000
C	4.98650300	-1.05534900	-0.77687200
H	5.20884500	-1.89865000	-0.11974400
H	5.60024700	-1.17526300	-1.67991100
H	5.32127000	-0.13952500	-0.28394300
C	3.31394700	0.24584000	-2.11661200
H	4.04345500	0.17955300	-2.93497400
H	2.31858700	0.26927600	-2.57702500
H	3.46838100	1.19852500	-1.61140700
C	3.18422600	-2.21561700	-2.08018100
H	3.43354200	-3.16493800	-1.60231800
H	2.13209600	-2.24372400	-2.38548100
H	3.78399600	-2.14775100	-2.99685200
Si	-2.75404900	-0.67650100	0.16802700
C	-4.30576300	0.43478600	0.48228300
H	-4.01544800	1.11220300	1.29312600
C	-2.99809300	-1.69455000	-1.45065100
H	-2.83489000	-0.96090700	-2.25000600
C	-2.39086200	-1.81939600	1.67712600
H	-1.40157100	-2.24082800	1.44880500
C	-4.41752200	-2.27280100	-1.63928000
H	-4.46758100	-2.81824800	-2.59171000
H	-4.69465000	-2.98237100	-0.85027500
H	-5.18744400	-1.49727500	-1.67507500
C	-1.96266400	-2.81329500	-1.66476300
H	-2.03084100	-3.59484300	-0.89699000
H	-2.12314100	-3.29715200	-2.63795000
H	-0.94032700	-2.42133000	-1.66461000
C	-5.56495400	-0.29019500	1.00607400
H	-6.37804600	0.43509700	1.15146300
H	-5.93605000	-1.05467200	0.31745500
H	-5.38858800	-0.77250400	1.97342200
C	-4.64776200	1.31228600	-0.74061200
H	-3.78275200	1.87962600	-1.10112500
H	-5.00735100	0.70986200	-1.58211700

H	-5.44356700	2.02827800	-0.49236600
C	-2.25972800	-1.04278300	3.00146500
H	-1.50371000	-0.25147700	2.93924800
H	-3.20960700	-0.57594900	3.29183600
H	-1.96900700	-1.71172800	3.82384100
C	-3.34670300	-3.01741100	1.85104600
H	-3.00725600	-3.65989500	2.67605000
H	-4.36706900	-2.70088600	2.09241000
H	-3.39984000	-3.64099000	0.95301600
Cl	-0.80330200	0.49368500	-3.13353500

Zero-point correction= 0.786596 (Hartree/Particle)
Thermal correction to Energy= 0.832330
Thermal correction to Enthalpy= 0.833275
Thermal correction to Gibbs Free Energy= 0.712331
Sum of electronic and zero-point Energies= -2467.951202
Sum of electronic and thermal Energies= -2467.905468
Sum of electronic and thermal Enthalpies= -2467.904524
Sum of electronic and thermal Free Energies= -2468.025467
E(RM06L) = -2470.37096075

TS_RE_IIc

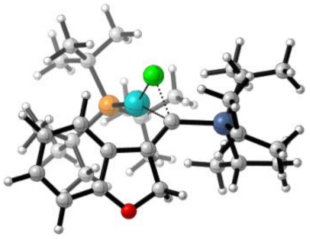

Pd	0.68916200	-0.22953200	-0.48101700
C	-2.08305500	0.45714500	-0.44683800
P	2.88268000	-0.54943300	0.05579100
C	3.15911100	-2.11367000	1.19832600
C	3.94579900	-0.80950000	-1.56805100
C	3.60557200	1.00558800	0.99583800
C	-1.85158000	1.62186600	0.21347300
C	-1.19916700	2.88541300	-0.15145300
C	-2.34907000	1.85315800	1.64485900
C	-1.36435800	3.76315500	0.93660500
H	-3.43159100	1.72615000	1.75146500
H	-1.84744600	1.20445600	2.36903100
O	-2.03473900	3.21547200	1.98431900
C	-0.52368200	3.35236900	-1.29084000
C	-0.89017600	5.07053600	0.93586000
H	-1.04144000	5.71044100	1.79867600
C	-0.04136500	4.66172900	-1.30901600
C	-0.22268600	5.51032600	-0.20842600
H	-0.36755000	2.70698100	-2.14378600
H	0.48184600	5.02443700	-2.18877900
H	0.16127300	6.52633200	-0.24278000
C	3.43230800	0.18397400	-2.63819800
H	3.93460100	-0.03320500	-3.59139600
H	2.35211900	0.07398900	-2.77999100
H	3.63364900	1.22710200	-2.39269900
C	5.47243100	-0.64353700	-1.42540100
H	5.90499500	-1.31640700	-0.68093100
H	5.94898700	-0.87358100	-2.38873800
H	5.75947100	0.37878300	-1.16603000
C	3.65654300	-2.21454700	-2.14079600
H	2.58021000	-2.39743500	-2.22607800
H	4.08612800	-2.27698900	-3.14967900
H	4.10485500	-3.01888100	-1.55284700
C	2.23912800	-3.24509200	0.68130000
H	2.52705400	-3.62258000	-0.30019400
H	2.28456200	-4.08848200	1.38459700
H	1.20235900	-2.90098200	0.61692500
C	4.60212300	-2.64894200	1.29707700
H	4.62467800	-3.48767500	2.00712600
H	4.97309200	-3.03131400	0.34284000
H	5.30865600	-1.89809700	1.65968600
C	2.66094400	-1.79566000	2.62529500
H	1.64913200	-1.37843500	2.61426200
H	2.62952700	-2.73010700	3.20166100
H	3.31694600	-1.10916200	3.16525900
C	2.54654700	1.47240100	2.02339000
H	2.38051800	0.75747300	2.82995900
H	2.88726000	2.41159400	2.48098300
H	1.58655800	1.65659800	1.53031200
C	3.75569300	2.17728300	0.00040100
H	3.96703600	3.09310800	0.56857000
H	4.58117200	2.03912000	-0.70177000
H	2.83353500	2.34441900	-0.56528000
C	4.95307000	0.80699700	1.71956000
H	5.27049700	1.76564200	2.15335900
H	4.88568100	0.09284000	2.54425000
H	5.74929200	0.47540200	1.04840900
Si	-3.29674500	-0.96512500	0.03728400
C	-3.01212400	-1.45199600	1.88176100
H	-3.43610700	-0.61833100	2.46324600
C	-3.77594400	-2.72497000	2.31049300
H	-3.36637600	-3.61536400	1.82035100
H	-3.67451800	-2.88028400	3.39324400
H	-4.84559600	-2.68328600	2.08423500
C	-1.52901900	-1.59872800	2.28250900
H	-1.43240000	-1.67990600	3.37425700
H	-1.09633600	-2.50785900	1.85163900
H	-0.90102200	-0.76755200	1.94176100
C	-5.01898900	-0.11472700	-0.17920200
H	-4.88799300	0.87110100	0.29405400
C	-6.21327400	-0.78491600	0.52796200
H	-7.13038300	-0.20335200	0.36021500
H	-6.40279800	-1.79772400	0.15417500
H	-6.06598400	-0.85227700	1.61131000
C	-5.34396300	0.15317600	-1.66383200
H	-4.53993300	0.69665000	-2.17227300
H	-5.51462800	-0.77841100	-2.21656300
H	-6.25816100	0.75516500	-1.75559700
C	-3.05843600	-2.41734100	-1.19320500
H	-3.03733300	-1.93509000	-2.17999100
C	-4.22631200	-3.42712400	-1.21682100
H	-4.04723100	-4.18938200	-1.98730100
H	-4.33703800	-3.95544500	-0.26345600
H	-5.18625600	-2.95317400	-1.44702600
C	-1.71735600	-3.15987900	-1.02241900
H	-1.69627600	-3.73901300	-0.09117800
H	-1.56722400	-3.87064400	-1.84682200
H	-0.85548000	-2.47963700	-1.01107500
Cl	-1.45883300	0.33960600	-2.14126300

Zero-point correction= 0.784635 (Hartree/Particle)
Thermal correction to Energy= 0.830277
Thermal correction to Enthalpy= 0.831221
Thermal correction to Gibbs Free Energy= 0.706688
Sum of electronic and zero-point Energies= -2467.940322
Sum of electronic and thermal Energies= -2467.894681
Sum of electronic and thermal Enthalpies= -2467.893737
Sum of electronic and thermal Free Energies= -2468.018270
E(RM06L) = -2470.34594385

***cis*-2c**

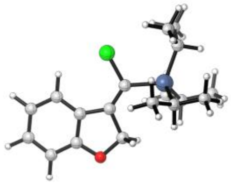

C	2.67367500	0.19479200	-0.00023100
C	1.28250900	-1.72462700	-0.00032700
C	3.42541600	-0.99536700	-0.00000300
H	0.81216300	-2.15611000	0.88909200
H	0.81282000	-2.15585900	-0.89022900
O	2.66570500	-2.12259500	0.00012700
C	3.35937500	1.41927800	-0.00036800
C	4.81569900	-1.01416900	0.00010400
H	5.35316600	-1.95647800	0.00028400
C	4.75507700	1.41976300	-0.00027400
C	5.47368100	0.21727100	-0.00003400
H	2.81702800	2.35459600	-0.00055700
H	5.28801400	2.36594500	-0.00039100
H	6.56016700	0.23924900	0.00003800
C	1.25677500	-0.19139600	-0.00021400
C	0.10077300	0.51251700	-0.00010800
Si	-1.69063400	-0.18336000	0.00004900
C	-3.04579800	1.17591500	0.00020100
H	-3.96317700	0.56395500	0.00021800
C	-1.87979000	-1.30410700	1.56667700
H	-1.39533500	-2.25523800	1.29535300
C	-1.87998600	-1.30405600	-1.56658400
H	-1.39515100	-2.25505200	-1.29547000
C	-1.15765000	-0.76725500	-2.81946000
H	-0.09278300	-0.58678500	-2.64052500
H	-1.24239800	-1.48260100	-3.64892500
H	-1.59141900	0.17863800	-3.16328400
C	-3.34689500	-1.64348100	-1.90728700
H	-3.91246400	-0.75083500	-2.19855100
H	-3.39072900	-2.34309400	-2.75301100
H	-3.87713900	-2.11227200	-1.07158000
C	-3.10528900	2.05686900	1.26602100
H	-3.98068600	2.71947200	1.22307600
H	-3.19143400	1.46578800	2.18370600
H	-2.21875600	2.69114100	1.35997100
C	-3.10540800	2.05700800	-1.26551400
H	-3.98068300	2.71975700	-1.22232000
H	-2.21878600	2.69114200	-1.35959300
H	-3.19185400	1.46602700	-2.18323200
C	-1.15686700	-0.76757300	2.81932700
H	-1.59018100	0.17848200	3.16328700
H	-1.24161300	-1.48288700	3.64881900
H	-0.09199200	-0.58749000	2.64005500
C	-3.34668200	-1.64310900	1.90787300
H	-3.39041800	-2.34282300	2.75351900
H	-3.91185700	-0.75032500	2.19947400
H	-3.87739600	-2.11161400	1.07230300
Cl	0.21967300	2.29726200	-0.00011900

Zero-point correction= 0.412144 (Hartree/Particle)
Thermal correction to Energy= 0.436552
Thermal correction to Enthalpy= 0.437496
Thermal correction to Gibbs Free Energy= 0.358827
Sum of electronic and zero-point Energies= -1526.665027
Sum of electronic and thermal Energies= -1526.640619
Sum of electronic and thermal Enthalpies= -1526.639675
Sum of electronic and thermal Free Energies= -1526.718344
E(RM06L) = -1527.29049084

TS_Isom_IIc

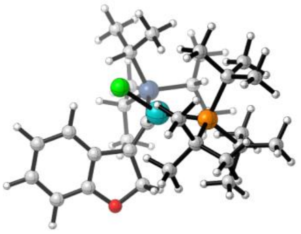

Pd	-0.40613700	-0.30791400	-0.49806200
C	2.57651200	-2.13610100	0.03781200
C	3.20261900	-2.10810100	-1.21299100
C	2.82144400	-3.20290400	0.91855100
C	4.08384400	-3.13759100	-1.54308600
H	2.97876400	-1.31631900	-1.92059200
C	3.69733600	-4.23653900	0.60600700
C	4.32831100	-4.18383500	-0.64051600
H	4.57305100	-3.13798600	-2.51237200
H	3.87000200	-5.04844900	1.30449400
H	5.01352500	-4.97990200	-0.91887600
P	-2.76196600	-0.06485500	0.16415900
C	1.64555800	-1.22241900	0.70318500
C	1.29907200	0.05758300	0.35206200
O	2.12793500	-3.10853600	2.08776900
C	1.27141300	-1.94998700	2.00046200
H	0.23492000	-2.29971900	1.99612100
H	1.43614000	-1.32964100	2.88757000
C	-3.66368300	1.33259600	-0.85098600
C	-2.88851800	0.38300100	2.06341700
C	-3.68779500	-1.75907900	-0.12329800
C	-3.27139000	1.20600800	-2.34116500
H	-2.19034600	1.23640900	-2.48661300
H	-3.63021600	0.28955100	-2.80666100
H	-3.71786500	2.05161900	-2.88182100
C	-3.15296100	2.71488100	-0.38760600
H	-3.52288700	3.47164900	-1.09111100
H	-3.51529500	2.99588600	0.60386000
H	-2.05965000	2.77149800	-0.39336000
C	-5.20416500	1.31986700	-0.74780200
H	-5.56893800	1.41725300	0.27661300
H	-5.59648500	2.17358200	-1.31676300
H	-5.64282700	0.41921200	-1.18293700
C	-4.02128400	-1.95200700	-1.61971200
H	-4.37807400	-2.98227600	-1.75087200
H	-4.82279000	-1.29323800	-1.96254500
H	-3.14338500	-1.82101300	-2.25634100
C	-4.99825500	-1.92713500	0.67521000
H	-5.45041700	-2.88738300	0.39377100
H	-4.84785200	-1.95198700	1.75651800
H	-5.73021500	-1.14718200	0.44912400
C	-2.70519000	-2.89624100	0.24683900
H	-1.80835700	-2.85424700	-0.37828200
H	-2.40247200	-2.88502200	1.29563600
H	-3.20103600	-3.85820600	0.06063500
C	-2.50354700	-0.84574500	2.91649300
H	-1.52839300	-1.24728000	2.62837500
H	-2.42972300	-0.52907700	3.96494400
H	-3.23645300	-1.65283100	2.87563300
C	-1.83505900	1.46253700	2.39590500
H	-2.02637000	2.42065700	1.91234300
H	-1.84334200	1.63662700	3.48018700
H	-0.83410600	1.13255600	2.10963400
C	-4.26923000	0.88913000	2.53441700
H	-4.23593100	1.02990900	3.62327000
H	-4.52810200	1.85673200	2.09805600
H	-5.07970200	0.18908600	2.32167900
Si	2.27719000	1.65756000	-0.01231600

C 1.42423800 3.05679800 0.99585200
H 0.36013200 2.93987600 0.74274100
C 4.12006300 1.36851200 0.50631000
H 4.54460600 0.76952300 -0.31474400
C 2.17206100 1.95904200 -1.91563200
H 2.26230800 0.94994500 -2.34405900
C 4.93659500 2.67874500 0.59076900
H 4.63350600 3.27777900 1.45758400
H 6.00397100 2.45176400 0.71758100
H 4.83993800 3.30926500 -0.29754500
C 3.30971600 2.80757700 -2.51781400
H 4.29825900 2.37685100 -2.32586900
H 3.19003800 2.87467200 -3.60767800
H 3.31047700 3.83429800 -2.13173700
C 1.83484400 4.49035400 0.60041600
H 1.23195200 5.22449200 1.15300400
H 2.88450400 4.69487300 0.83737300
H 1.69223500 4.68908700 -0.46646300
C 4.33368900 0.56792400 1.80622900
H 3.91262600 1.08498400 2.67647500
H 3.89227800 -0.43032700 1.76097000
H 5.40777400 0.43848200 1.99787700
C 0.80177100 2.50083800 -2.36399300
H 0.62937400 3.52441800 -2.00937200
H 0.73610200 2.51567600 -3.45972800
H -0.01803100 1.86967300 -2.00439800
C 1.55882500 2.87497600 2.52082400
H 1.27262800 1.86972200 2.84991800
H 2.58876000 3.04983900 2.85348400
H 0.92150000 3.59202600 3.05641400
Cl -0.69549800 -1.21495400 -2.74349200

Zero-point correction= 0.784443 (Hartree/Particle)
Thermal correction to Energy= 0.830117
Thermal correction to Enthalpy= 0.831062
Thermal correction to Gibbs Free Energy= 0.708217
Sum of electronic and zero-point Energies= -2467.938841
Sum of electronic and thermal Energies= -2467.893167
Sum of electronic and thermal Enthalpies= -2467.892222
Sum of electronic and thermal Free Energies= -2468.015067
E(RM06L) = -2470.35708450

IIIc

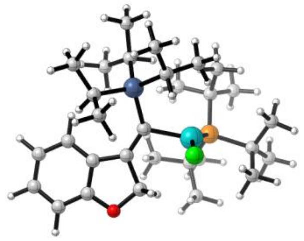

Pd 0.72392300 -0.32568200 -1.17626600
C -2.52307700 2.15956800 0.14438200
C -3.32247800 2.02624100 1.28771500
C -2.56249000 3.38322700 -0.55257000
C -4.17605300 3.06514600 1.66368700
H -3.27364800 1.13261100 1.89507200
C -3.41209100 4.42815900 -0.19903200
C -4.22878100 4.24984200 0.91847700
H -4.79712300 2.95348000 2.54759900
H -3.41628200 5.34698200 -0.77605700
H -4.89997500 5.04886900 1.22161200
P 2.61030900 0.23385600 0.35897000
C 2.89858900 -0.88223100 1.92629500
C 4.06162500 -0.10287200 -0.90865600
C 2.69590600 2.09663600 0.91909100
C -1.49863600 1.31414400 -0.50737500
C -1.08542600 0.04166900 -0.29685600
O -1.68032100 3.45809200 -1.57664900
C -0.91899200 2.23118200 -1.59630400
H -1.01760200 1.76929100 -2.58110700
H 0.13286500 2.48656400 -1.43410200
C 1.33648900 2.46585200 1.56026400
H 1.19040200 2.01040600 2.54028000
H 1.29895700 3.55380800 1.70086100
H 0.49304400 2.17646800 0.93092500
C 2.86450800 3.00601900 -0.31903700
H 2.72231600 4.04736100 -0.00408100
H 3.85959600 2.93895300 -0.76411000
H 2.12501400 2.79705700 -1.09650300
C 3.82332200 2.44641300 1.91343800
H 3.78755700 3.52583100 2.11303900
H 3.70945600 1.94228200 2.87558400
H 4.81891700 2.22101900 1.52401400
C 1.98702800 -0.39373400 3.07412900
H 1.99763500 -1.14990000 3.86958700
H 2.33002600 0.54215100 3.51971600
H 0.94893400 -0.26862800 2.75466600
C 2.44293800 -2.31978800 1.59420100
H 3.05530400 -2.80491000 0.83444800
H 2.51111500 -2.92762800 2.50602900
H 1.40797300 -2.34245400 1.25227500
C 4.35010400 -0.94128100 2.44875900
H 5.02989500 -1.42033900 1.73994300
H 4.75706800 0.03928100 2.70436700
H 4.36469500 -1.54808900 3.36424100
C 5.42540500 0.51417500 -0.53602900
H 5.79721400 0.17392200 0.43316000
H 6.16333600 0.21375900 -1.29216200
H 5.40524500 1.60625600 -0.52870800
C 3.63751000 0.43776500 -2.29772400
H 4.43080700 0.19970700 -3.01881300
H 2.71874700 -0.03349300 -2.67070900
H 3.48734700 1.51653000 -2.32116100
C 4.25238300 -1.62586600 -1.09329800
H 4.70828200 -2.10831100 -0.22605500
H 3.31218700 -2.13678400 -1.32662500
H 4.92840000 -1.78647600 -1.94271200
Si -2.05975700 -1.54104600 0.24583700
C -3.92017200 -1.09729300 -0.03388300
H -4.04060400 -0.08221600 0.35438600
C -1.60587100 -3.03459900 -0.89218700
H -1.60691000 -2.60030600 -1.89976200
C -1.59994600 -1.94259800 2.07945400
H -0.50368300 -2.01833100 2.04530200
C -2.64802400 -4.17544900 -0.88538200
H -2.34309000 -4.95405400 -1.59818300
H -2.74105100 -4.65864400 0.09401700
H -3.64418000 -3.84019500 -1.18430200
C -0.20551000 -3.63215800 -0.67155800
H -0.10013800 -4.09331800 0.31908300
H -0.00535300 -4.41410200 -1.41693200
H 0.57856200 -2.87472200 -0.78005000
C -4.93827200 -1.96702800 0.73343300
H -5.96067900 -1.62602300 0.51769800
H -4.88830100 -3.02553100 0.45737900
H -4.79899800 -1.90193000 1.81854700
C -4.27488700 -1.01450300 -1.53417500
H -3.58260900 -0.36813300 -2.08342600
H -4.26006600 -1.99535300 -2.02134100
H -5.28661300 -0.60521500 -1.66088800
C -1.93419400 -0.82898500 3.08809800

H	-1.50434000	0.13603000	2.79772400
H	-3.01812000	-0.69471200	3.19644600
H	-1.54513800	-1.07717200	4.08583100
C	-2.11475300	-3.29828200	2.60622800
H	-1.74785200	-3.47345000	3.62767900
H	-3.20883400	-3.33618600	2.64746000
H	-1.77794000	-4.13813600	1.99144900
Cl	-0.30086000	-0.72627500	-3.26817400

Zero-point correction= 0.786378 (Hartree/Particle)
Thermal correction to Energy= 0.832224
Thermal correction to Enthalpy= 0.833168
Thermal correction to Gibbs Free Energy= 0.711501
Sum of electronic and zero-point Energies= -2467.949560
Sum of electronic and thermal Energies= -2467.903715
Sum of electronic and thermal Enthalpies= -2467.902771
Sum of electronic and thermal Free Energies= -2468.024438
E(RM06L) = -2470.36692935

TS_RE_IIIc

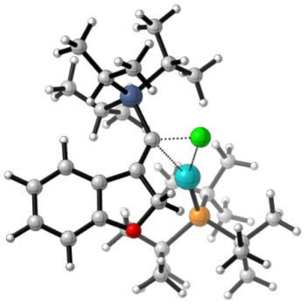

Pd	0.85811000	-0.41480100	-0.57460000
C	-1.90879700	-0.01379000	-0.98885700
P	3.00415200	-0.13987800	0.15026200
C	3.65085000	-1.67533800	1.17519900
C	4.20197600	0.09575100	-1.37993700
C	3.16803000	1.44516300	1.28386100
C	-1.75702900	1.33821500	-0.97131300
C	-2.25417400	2.37232300	-0.05299400
C	-0.98691800	2.11713200	-2.03675800
C	-1.72205800	3.59980900	-0.49785200
H	0.03937300	1.76022100	-2.15296400
H	-1.48961400	2.07795400	-3.01213600
O	-0.94585800	3.49068500	-1.60411200
C	-3.10284300	2.38670700	1.06411500
C	-1.97748200	4.81145000	0.13735800
H	-1.54130000	5.72974100	-0.24125300
C	-3.37433700	3.59229100	1.71178900
C	-2.81112400	4.79145100	1.25595000
H	-3.56180900	1.47833000	1.43020900
H	-4.03259100	3.59804500	2.57533000
H	-3.03218600	5.72122100	1.77289200
C	3.49263100	1.00870900	-2.40878300
H	4.11045800	1.06480600	-3.31597300
H	2.51553000	0.59558200	-2.68003900
H	3.34105200	2.02824000	-2.05304500
C	5.59734200	0.67964700	-1.07699100
H	6.15749200	0.08663300	-0.34995900
H	6.18583200	0.69830300	-2.00499900
H	5.55012400	1.70878000	-0.71137500
C	4.38279300	-1.26183500	-2.09443600
H	3.41803200	-1.73759800	-2.29916700
H	4.87945300	-1.08662000	-3.05821800
H	5.00828400	-1.96168500	-1.53565600
C	3.13530200	-2.95811800	0.47940200
H	3.58021500	-3.12630300	-0.50171000
H	3.38370900	-3.82561800	1.10671400
H	2.04877600	-2.92146300	0.35204400
C	5.17860000	-1.78369400	1.35781200
H	5.40127700	-2.65219600	1.99347200
H	5.70466100	-1.93956700	0.41282500
H	5.60963800	-0.90578600	1.84576000
C	2.99820400	-1.67149100	2.57515400
H	1.91112100	-1.56464500	2.51538800
H	3.21057100	-2.63318800	3.06130600
H	3.39136500	-0.88972300	3.22902100
C	1.95418200	1.46797600	2.24368500
H	1.95961500	0.65329700	2.96839600
H	1.96729100	2.41071600	2.80815700
H	1.01562100	1.41375400	1.68235300
C	3.02683700	2.71242700	0.41189200
H	2.91911800	3.58201700	1.07390600
H	3.89891300	2.89777900	-0.21952000
H	2.13543900	2.66866300	-0.22238600
C	4.46395400	1.56735000	2.11136500
H	4.44934600	2.51929200	2.66040200
H	4.56286500	0.77324900	2.85572100
H	5.36392100	1.56467700	1.49140000
Si	-2.97499300	-1.20386600	0.09430500
C	-2.40723000	-0.92868000	1.92450300
H	-2.05648200	0.11091400	1.95020300
C	-3.49740500	-1.06994600	3.00497900
H	-3.97380600	-2.05782300	2.98661100
H	-3.05730100	-0.94194600	4.00340900
H	-4.28956400	-0.32041200	2.90382200
C	-1.18436800	-1.78818400	2.30215300
H	-0.79429900	-1.46981000	3.27887100
H	-1.44206100	-2.84977100	2.38731900
H	-0.37103000	-1.68511900	1.57230000
C	-4.76936200	-0.59884100	-0.33091100
H	-4.71961100	0.49242500	-0.20512500
C	-5.95315700	-1.09194700	0.52593300
H	-6.88366200	-0.61899700	0.18245700
H	-6.10007200	-2.17430300	0.44555000
H	-5.84072200	-0.85487200	1.58668200
C	-5.07738200	-0.85173300	-1.82402100
H	-4.29481900	-0.46510800	-2.48583300
H	-5.19403200	-1.92160800	-2.03601600
H	-6.01928800	-0.36285500	-2.10720400
C	-2.75112200	-3.00832500	-0.54187500
H	-3.02398200	-2.93555200	-1.60447000
C	-3.74930600	-3.98817000	0.11518400
H	-3.60900700	-4.99933900	-0.29041300
H	-3.60028800	-4.05626500	1.20032500
H	-4.79282800	-3.71103800	-0.06089000
C	-1.33507100	-3.62351900	-0.48714400
H	-1.08069700	-3.94817300	0.52624500
H	-1.29428300	-4.51363000	-1.12995100
H	-0.54867200	-2.93948200	-0.82119100
Cl	-1.18722900	-0.79371800	-2.47059200

Zero-point correction= 0.784970 (Hartree/Particle)
Thermal correction to Energy= 0.830582
Thermal correction to Enthalpy= 0.831527
Thermal correction to Gibbs Free Energy= 0.706117
Sum of electronic and zero-point Energies= -2467.935791
Sum of electronic and thermal Energies= -2467.890178
Sum of electronic and thermal Enthalpies= -2467.889234
Sum of electronic and thermal Free Energies= -2468.014644
E(RM06L) = -2470.34151313

***trans*-2c**

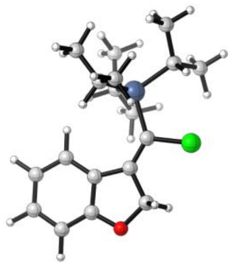

C	2.28239400	-0.09892900	0.01220600
C	2.28193600	2.26701500	0.09296200
C	3.58933200	0.42666700	-0.01834500
H	2.10510500	2.94035300	-0.75249600
H	2.17543100	2.84584400	1.01745200
O	3.63519100	1.78165800	0.01310400
C	2.12956400	-1.49173000	0.00923100
C	4.72662500	-0.37417100	-0.06895700
H	5.71284700	0.07704600	-0.09346600
C	3.25682200	-2.31247400	-0.03811600
C	4.54247600	-1.75713200	-0.08057900
H	1.14759100	-1.94276000	0.04945500
H	3.13346300	-3.39129600	-0.04083400
H	5.41020000	-2.40999000	-0.11857900
C	1.36320400	1.04748100	0.06330600
C	0.01170700	1.13844100	0.07950300
Si	-1.39108300	-0.18213700	0.05550600
C	-3.07628400	0.74593400	0.00203200
H	-2.97093200	1.54622400	0.74718500
C	-1.15417600	-1.13698700	1.71277700
H	-0.07011900	-1.31476300	1.76756800
C	-1.14990000	-1.34942500	-1.46633600
H	-0.39509400	-2.08894000	-1.16578700
C	-0.59864900	-0.62946700	-2.71412000
H	0.36947800	-0.15703800	-2.52158000
H	-0.46343600	-1.34239700	-3.53902100
H	-1.28313300	0.15047500	-3.06784100
C	-2.41518600	-2.15598500	-1.83205700
H	-3.21486800	-1.50842600	-2.20642000
H	-2.18553200	-2.87664100	-2.62871900
H	-2.81562900	-2.72253900	-0.98436600
C	-3.37525200	1.42566400	-1.35144600
H	-4.27815300	2.04674400	-1.27382100
H	-2.55948900	2.07458800	-1.68345100
H	-3.56087800	0.68824100	-2.14083900
C	-4.28746100	-0.10928500	0.43452800
H	-5.19935400	0.50349000	0.42843200
H	-4.46345000	-0.95169000	-0.24282700
H	-4.17682000	-0.51336300	1.44571500
C	-1.51224800	-0.26621100	2.93475100
H	-2.58677600	-0.05499200	2.98631200
H	-1.23826200	-0.77773800	3.86744800
H	-0.98617300	0.69515300	2.92028100
C	-1.83622800	-2.51649000	1.79247000
H	-1.59696900	-3.00914600	2.74503200
H	-2.92822500	-2.44306200	1.73421600
H	-1.50731400	-3.18590000	0.98910500
Cl	-0.57952600	2.84127400	0.20571000

Zero-point correction= 0.412235 (Hartree/Particle)
Thermal correction to Energy= 0.436480
Thermal correction to Enthalpy= 0.437424
Thermal correction to Gibbs Free Energy= 0.359754
Sum of electronic and zero-point Energies= -1526.665485
Sum of electronic and thermal Energies= -1526.641240
Sum of electronic and thermal Enthalpies= -1526.640296
Sum of electronic and thermal Free Energies= -1526.717965
E(RM06L) = -1527.29268168

k) reaction of aryl chloride **1d** (R = Mes), L = P*t*Bu$_3$

1d

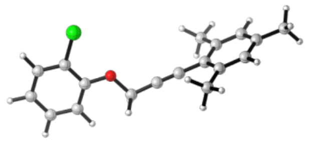

C	4.16864400	0.70138000	-0.45135400
C	5.55733000	0.63691100	-0.42465200
C	3.38479600	-0.29278000	0.16889000
C	6.19864400	-0.42234600	0.22297900
H	6.12868900	1.41952300	-0.91290000
C	4.03987200	-1.34984400	0.81519700
C	5.43581100	-1.41015400	0.83958800
H	7.28321300	-0.46761400	0.24076600
H	3.46737400	-2.13076800	1.30195100
H	5.92009200	-2.23975200	1.34690600
O	2.03627500	-0.14542300	0.08931900
C	1.21724600	-1.13769300	0.72072600
H	1.43910200	-2.12707400	0.29407100
H	1.45132600	-1.17947900	1.79493500
C	-0.18535900	-0.80453500	0.51779200
C	-1.36373000	-0.55994800	0.36691700
C	-2.75105600	-0.27275400	0.18552500
C	-3.37875600	0.70975800	0.98940200
C	-3.49000300	-0.96898300	-0.80179600
C	-4.73465700	0.97218200	0.79012600
C	-4.84395800	-0.67076900	-0.96035900
C	-5.48716700	0.29198800	-0.17434600
H	-5.21741600	1.73015700	1.40381700
H	-5.41290100	-1.20214600	-1.72055700
C	-2.59463700	1.46225200	2.03634400
H	-2.17408600	0.78111600	2.78614400
H	-1.74827700	1.99932000	1.59209100
H	-3.22837800	2.18891700	2.55353700
C	-2.82344900	-2.00928900	-1.66818700
H	-1.98582900	-1.58112100	-2.23158300
H	-2.40984100	-2.82637100	-1.06459900
H	-3.53240700	-2.43907700	-2.38235100
C	-6.96150000	0.57289800	-0.34532100
H	-7.29261500	0.36936200	-1.36924600
H	-7.56496900	-0.05656200	0.32286800
H	-7.20127900	1.61567600	-0.11115800
Cl	3.37997500	2.03645000	-1.26612200

Zero-point correction= 0.298346 (Hartree/Particle)
Thermal correction to Energy= 0.318295
Thermal correction to Enthalpy= 0.319239
Thermal correction to Gibbs Free Energy= 0.245817
Sum of electronic and zero-point Energies= -1231.203207
Sum of electronic and thermal Energies= -1231.183258
Sum of electronic and thermal Enthalpies= -1231.182314
Sum of electronic and thermal Free Energies= -1231.255736
E(RM06L) = -1231.70334504

PC_1d

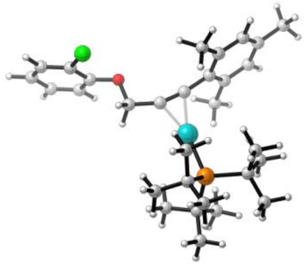

C	5.50370800	-1.51696100	-0.61020000
C	6.62341900	-2.33599900	-0.46441000
C	4.86065700	-0.97068800	0.51250000
C	7.11122700	-2.62169500	0.81130700
H	7.10526600	-2.73974100	-1.34881400
C	5.36401200	-1.26632500	1.78405900
C	6.48264300	-2.08451700	1.93633800
H	7.98437400	-3.25833600	0.92049700
H	4.86625800	-0.82551000	2.64276400
H	6.86343300	-2.29883200	2.93081100
C	2.50718600	-0.77436400	0.30526400
C	1.44860800	0.24081800	0.22483700
C	0.98163700	1.39522200	0.17166500
Pd	-0.64674800	-0.14793800	0.11294000
P	-2.76038700	-1.22599400	-0.01598900
C	-3.11822200	-2.23805600	1.61080700
C	-4.16459200	0.10682700	-0.23868800
C	-2.82694000	-2.45282400	-1.52995200
C	-4.26108400	-3.27021600	1.53738800
C	-1.80826700	-2.95765600	2.01599800
C	-3.41891800	-1.25356900	2.76267200
C	-5.60225300	-0.37247200	0.04567800
C	-3.83605500	1.30471400	0.68593400
C	-4.11164400	0.66827500	-1.67687900
C	-2.13078900	-1.76634500	-2.73063600
C	-1.96839800	-3.69729300	-1.21180300
C	-4.22989800	-2.92422500	-1.96248400
H	-4.39229900	-3.73047000	2.52652300
H	-5.21801200	-2.82150300	1.25806200
H	-4.04851200	-4.08060100	0.83538400
H	-0.98048700	-2.24389600	2.08886300
H	-1.95163500	-3.42366200	3.00060500
H	-1.51408800	-3.74588600	1.32256200
H	-4.39480800	-0.77133200	2.67130000
H	-3.42420000	-1.81364800	3.70714800
H	-2.64862900	-0.47937400	2.84103300
H	-5.75086600	-0.65492800	1.09097500
H	-5.89648500	-1.21911600	-0.57998500
H	-6.30078500	0.44888100	-0.16596800
H	-4.55808100	2.10896500	0.48860000
H	-3.89877700	1.06338800	1.74719100
H	-2.83070600	1.69074800	0.48676700
H	-4.76804000	1.54679700	-1.73084000
H	-3.10169100	0.99293300	-1.94718300
H	-4.46571000	-0.04236300	-2.42709100
H	-2.68366400	-0.91145100	-3.12054200
H	-2.03711300	-2.49580800	-3.54681100
H	-1.12834700	-1.42169600	-2.45633900
H	-0.96604600	-3.41934200	-0.86930000
H	-2.42441200	-4.35458300	-0.46796300
H	-1.85237300	-4.28496200	-2.13197200
H	-4.85003000	-2.10427500	-2.33376100
H	-4.77515100	-3.42483900	-1.15816800
H	-4.12724500	-3.64500000	-2.78550800
C	0.67536500	2.79432000	0.10115200
C	0.38541600	3.51765200	1.28490800
C	0.66460800	3.44835400	-1.15658800
C	0.09266200	4.87830300	1.18487000
C	0.36619500	4.81058700	-1.20102400
C	0.08056000	5.54629400	-0.04496800
H	-0.13179100	5.43390000	2.09341000
H	0.35677800	5.31310300	-2.16636700
C	0.38977000	2.82793600	2.62601400
H	-0.30232400	1.97648400	2.63353300
H	1.38205800	2.42514500	2.86395200
H	0.10208100	3.51908100	3.42450900
C	0.96648200	2.68410800	-2.42113900
H	1.97934900	2.26389800	-2.40136900
H	0.28007800	1.83688200	-2.54347000
H	0.88161500	3.33042000	-3.30033500
C	-0.20526300	7.02777600	-0.12012000
H	-0.86053100	7.35223100	0.69553400
H	0.72009500	7.61551900	-0.04627300
H	-0.68385300	7.29681200	-1.06810200
O	3.79482600	-0.11783900	0.38707800
Cl	4.90986400	-1.14849300	-2.21962800
H	2.36509300	-1.40696700	1.19205100
H	2.47912300	-1.42280200	-0.57926000

Zero-point correction= 0.670726 (Hartree/Particle)
Thermal correction to Energy= 0.712738
Thermal correction to Enthalpy= 0.713682
Thermal correction to Gibbs Free Energy= 0.589360
Sum of electronic and zero-point Energies= -2172.503333
Sum of electronic and thermal Energies= -2172.461321
Sum of electronic and thermal Enthalpies= -2172.460377
Sum of electronic and thermal Free Energies= -2172.584698
E(RM06L) = -2174.76780230

TS_OA_1d

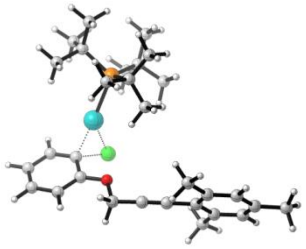

Pd	-1.89830300	0.82338100	0.34352200
C	-1.39806300	2.79247500	0.38353900
C	-2.55726900	3.57787200	0.38155100
C	-0.27193700	3.16240000	-0.39782300
C	-2.65241400	4.68303700	-0.47278400
H	-3.36401500	3.33043700	1.06417400
C	-0.38257700	4.27640300	-1.23520500
C	-1.56593800	5.02775900	-1.27135700
H	-3.56254900	5.27586600	-0.48971300
H	0.44792900	4.57094700	-1.86735100
H	-1.62026100	5.89355500	-1.92532600
P	-2.50434300	-1.48271600	-0.05284900
C	-4.28763600	-1.63433700	-0.81580700
C	-1.22026200	-2.23468200	-1.30766900
C	-2.44166100	-2.50802600	1.59986400
O	0.84003300	2.38144300	-0.28150000
C	2.00340400	2.76802200	-1.01848500
H	1.78044100	2.77156500	-2.09628200
H	2.29567300	3.79114100	-0.73805100
C	-3.10308100	-1.65857200	2.71285300
H	-2.97226900	-2.17812800	3.67173000
H	-2.62969600	-0.67501600	2.79425400
H	-4.17350100	-1.51043500	2.56849100
C	-0.96863400	-2.67943000	2.03341100

H	-0.41453600	-3.37810100	1.40237200
H	-0.43781200	-1.72227200	2.05470500
H	-0.95382500	-3.08641500	3.05290600
C	-3.10741500	-3.89827900	1.55732200
H	-2.95838400	-4.39487700	2.52592600
H	-4.18658600	-3.84170600	1.39190000
H	-2.67989400	-4.54829700	0.78929900
C	0.18388600	-1.70817800	-0.91901400
H	0.54077100	-2.08788800	0.03809800
H	0.90261800	-2.02827500	-1.68622200
H	0.20403000	-0.61384100	-0.87095600
C	-1.49557900	-1.67423700	-2.72096600
H	-1.55887100	-0.58080700	-2.71956500
H	-0.65871600	-1.95421000	-3.37422600
H	-2.40388500	-2.07751000	-3.17440400
C	-1.17886000	-3.77363900	-1.39380900
H	-0.45442400	-4.06984500	-2.16501100
H	-0.85458200	-4.23586100	-0.45808900
H	-2.14399100	-4.20673800	-1.66994900
C	-4.46314300	-0.48508900	-1.83847400
H	-3.82274900	-0.58116900	-2.71542900
H	-5.50347000	-0.48148500	-2.19125600
H	-4.25919600	0.48736500	-1.37635800
C	-5.33994200	-1.37194000	0.28459300
H	-6.32376700	-1.27038700	-0.19220600
H	-5.41643400	-2.18782900	1.00659700
H	-5.13871600	-0.44177800	0.82611200
C	-4.62094500	-2.97643400	-1.49807300
H	-5.66394100	-2.95476100	-1.84300900
H	-3.99882000	-3.16620000	-2.37641800
H	-4.51962600	-3.82822600	-0.82048400
C	3.08247200	1.83203800	-0.73291700
C	3.99832100	1.06828200	-0.51092100
C	5.07506500	0.16939500	-0.24065500
C	5.48714500	-0.75544800	-1.23032300
C	5.71941100	0.20285600	1.02029100
C	6.53945100	-1.62494700	-0.94027900
C	6.76505600	-0.68927600	1.26159200
C	7.19456200	-1.60720700	0.29624400
H	6.85565100	-2.33802200	-1.69907900
H	7.25818500	-0.66849200	2.23140100
C	4.79640900	-0.80251100	-2.57124800
H	3.72405500	-1.00428700	-2.45977200
H	4.88083100	0.15552200	-3.09858800
H	5.22743000	-1.58084600	-3.20839300
C	5.27780200	1.17897000	2.08281300
H	5.37952600	2.21533600	1.73877500
H	4.22138100	1.03885100	2.34055600
H	5.87192800	1.06234000	2.99426100
C	8.35185300	-2.53767100	0.57383400
H	8.27281300	-3.46167400	-0.00889300
H	9.30990600	-2.06859800	0.31114000
H	8.40424500	-2.80760800	1.63414000
Cl	-0.89723500	2.00203900	2.32931500

Zero-point correction= 0.669986 (Hartree/Particle)
Thermal correction to Energy= 0.711534
Thermal correction to Enthalpy= 0.712478
Thermal correction to Gibbs Free Energy= 0.591016
Sum of electronic and zero-point Energies= -2172.470277
Sum of electronic and thermal Energies= -2172.428729
Sum of electronic and thermal Enthalpies= -2172.427785
Sum of electronic and thermal Free Energies= -2172.549247
E(RM06L) = -2174.73909021

Id

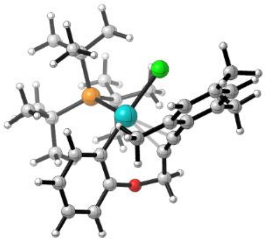

Pd	-0.00768800	0.00108700	0.11710700
C	-0.36033400	1.96320800	-0.18529900
C	-0.34447000	2.51150700	-1.47377500
C	-0.44622900	2.84586200	0.89882400
C	-0.46929200	3.89349000	-1.67172200
H	-0.24680400	1.86015100	-2.33911100
C	-0.58153600	4.22321500	0.71808100
C	-0.60792400	4.74791500	-0.57673100
H	-0.46562800	4.29582000	-2.68183000
H	-0.66547900	4.86473900	1.59102200
H	-0.71970700	5.81851300	-0.72542100
P	-2.36586300	-0.74017900	-0.19164300
C	-2.42445300	-2.22526700	-1.47947800
C	-2.91196600	-1.38411800	1.56785300
C	-3.71048700	0.57149700	-0.78645400
C	1.50526100	0.98039800	1.50721700
C	2.51813300	0.58039800	0.91903400
O	-0.41704100	2.34536300	2.19347800
C	-1.73655400	-2.15128800	2.21313400
H	-0.86436000	-1.50790400	2.35132000
H	-2.06476000	-2.50211500	3.20116800
H	-1.40815900	-3.01595500	1.63889800
C	-3.20761000	-0.18471200	2.49615700
H	-3.29948000	-0.56521500	3.52195300
H	-2.40125100	0.55549500	2.48947800
H	-4.15003700	0.31403500	2.25744700
C	-4.14936800	-2.30898500	1.55765900
H	-3.97417400	-3.24141500	1.01803200
H	-4.38078200	-2.57901200	2.59674600
H	-5.03934300	-1.83506400	1.14187200
C	-3.58543300	1.92668900	-0.04712700
H	-4.47449700	2.52049600	-0.29930900
H	-3.54888100	1.84156400	1.03704200
H	-2.71501500	2.48857800	-0.37228400
C	-3.49115000	0.89641200	-2.28236500
H	-2.46810200	1.22416900	-2.47916000
H	-3.72879800	0.07032400	-2.95345900
H	-4.15424800	1.73002100	-2.54870300
C	-5.17042200	0.09983000	-0.58162600
H	-5.38513800	-0.89162000	-0.97748600
H	-5.45583200	0.11948500	0.47334600
H	-5.82960500	0.80841300	-1.10060700
C	-3.82164800	-2.55269800	-2.05224100
H	-4.27254500	-1.74269600	-2.62623500
H	-3.70694500	-3.40277000	-2.73738200
H	-4.52648300	-2.86253300	-1.27527700
C	-1.46704900	-1.84064700	-2.63248500
H	-1.75056700	-0.91433600	-3.13777000
H	-0.44105800	-1.74330600	-2.26645900
H	-1.48213400	-2.64279900	-3.38243100
C	-1.89167900	-3.54390800	-0.87420200
H	-2.56978500	-3.97241100	-0.13243200
H	-1.81724400	-4.27160700	-1.69370300
H	-0.89798000	-3.43198900	-0.44269600
C	0.84905900	1.79568900	2.56325100
H	1.52717600	2.61406300	2.84792200
H	0.66233600	1.17720500	3.44648300

C 3.70503300 0.08619900 0.32271700
C 4.44437800 -0.93001800 0.98710500
C 4.16950800 0.63530800 -0.90133400
C 5.63320700 -1.36847000 0.40716500
C 5.36011100 0.15179700 -1.43922600
C 6.10436800 -0.85078700 -0.80533600
H 6.20674000 -2.14257400 0.91207300
H 5.71896100 0.56701500 -2.37849100
C 3.38346200 1.70433100 -1.61506000
H 2.40565100 1.32551100 -1.93495100
H 3.18435200 2.56444100 -0.96565800
H 3.91894400 2.05832600 -2.50117500
C 3.96621100 -1.51321300 2.29203800
H 3.90063100 -0.74669800 3.07466900
H 2.96867700 -1.94777500 2.16730100
H 4.64910800 -2.29343500 2.64135700
C 7.37313200 -1.38045200 -1.42830600
H 8.08825700 -1.70867600 -0.66649500
H 7.16112300 -2.24686500 -2.06934300
H 7.86039200 -0.62471200 -2.05340100
Cl 1.06028500 -2.23353900 0.15107100
Zero-point correction= 0.672697 (Hartree/Particle)
Thermal correction to Energy= 0.713856
Thermal correction to Enthalpy= 0.714800
Thermal correction to Gibbs Free Energy= 0.600187
Sum of electronic and zero-point Energies= -2172.499406
Sum of electronic and thermal Energies= -2172.458247
Sum of electronic and thermal Enthalpies= -2172.457303
Sum of electronic and thermal Free Energies= -2172.571916
E(RM06L) = -2174.78697676

TS_AI_Id

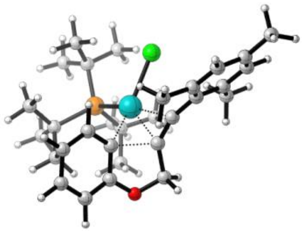

Pd 0.13692100 0.01848100 -0.15810400
C -0.02411000 2.16100500 -0.09743200
C -0.02974800 2.55005300 -1.44873700
C -0.42021500 3.10640500 0.86008400
C -0.54923900 3.79333600 -1.82396500
H 0.33967700 1.87310300 -2.21391200
C -0.92627400 4.35411300 0.50139000
C -1.01612400 4.68375200 -0.85232200
H -0.58049800 4.06554800 -2.87531600
H -1.21218900 5.05351900 1.28097500
H -1.41007300 5.65320100 -1.14427300
P -2.35047300 -0.79577200 -0.09924500
C -2.67890600 -2.39667900 -1.18426400
C -2.56425200 -1.27892300 1.77962200
C -3.77007200 0.47599500 -0.55596100
C 1.34097600 1.35341900 1.04038400
C 2.20705500 0.57332300 0.52211200
O -0.20366900 2.81149700 2.18049600
C -1.26750700 -1.98513700 2.24198800
H -0.39519800 -1.33650600 2.11494900
H -1.36250600 -2.22376500 3.31028600
H -1.06497900 -2.91398300 1.70952400
C -2.68949300 -0.00110600 2.63909200
H -2.60285200 -0.28744700 3.69583900
H -1.89877400 0.72601800 2.43019300
H -3.65640900 0.49446900 2.52291800
C -3.76456600 -2.19272300 2.10746700
H -3.68364200 -3.17716500 1.64267400
H -3.79399000 -2.35276800 3.19406500
H -4.72292300 -1.75667800 1.81872600
C -3.40947500 1.88219100 -0.03309100
H -4.25661000 2.55075400 -0.23893500
H -3.21702600 1.92201700 1.03804000
H -2.54256200 2.28366900 -0.55284700
C -3.85254600 0.62903400 -2.09232000
H -2.87344300 0.84451600 -2.53204700
H -4.27652600 -0.24083100 -2.59586200
H -4.50601100 1.48253900 -2.31660000
C -5.17237200 0.12478000 -0.01101600
H -5.51540400 -0.87317700 -0.28415600
H -5.22380000 0.21916500 1.07688700
H -5.89267600 0.84342400 -0.42557800
C -4.16355500 -2.79222200 -1.34588000
H -4.75022600 -2.05894000 -1.90244200
H -4.20142300 -3.72848100 -1.91864500
H -4.66083500 -2.97949500 -0.39062600
C -2.07299300 -2.15100500 -2.58764100
H -2.55096500 -1.33132900 -3.12606600
H -0.99985500 -1.95991000 -2.52593400
H -2.21686900 -3.06036100 -3.18693100
C -1.94890700 -3.62491300 -0.59328400
H -2.40324700 -3.98049800 0.33478400
H -2.03249500 -4.44278400 -1.32149400
H -0.88640400 -3.43687400 -0.43755300
C 1.00081100 2.04674900 2.32201600
H 1.81677400 2.71903900 2.62620500
H 0.83453200 1.31238600 3.11499100
C 3.49409200 0.01258100 0.24566700
C 3.94834400 -1.12679400 0.95862400
C 4.32528200 0.62796700 -0.72562200
C 5.23027400 -1.60871500 0.69626000
C 5.59605800 0.10176800 -0.95080100
C 6.06809800 -1.01772400 -0.25573600
H 5.58470800 -2.47520500 1.25052100
H 6.23455600 0.57481300 -1.69408300
C 3.07767700 -1.79426500 1.99146700
H 2.17539500 -2.19659600 1.51725000
H 3.61080300 -2.61625900 2.47907200
H 2.76001200 -1.08925500 2.77038200
C 3.83843700 1.81460400 -1.51944900
H 3.49071100 2.62704500 -0.87051600
H 4.63160800 2.20748000 -2.16279000
H 2.99213000 1.53488700 -2.15894200
C 7.43422700 -1.59068400 -0.54709600
H 7.86699400 -2.06816600 0.33861600
H 7.37988500 -2.35511700 -1.33406400
H 8.12927500 -0.81785800 -0.89266300
Cl 1.09915000 -2.05969400 -1.03155500
Zero-point correction= 0.671446 (Hartree/Particle)
Thermal correction to Energy= 0.711924
Thermal correction to Enthalpy= 0.712868
Thermal correction to Gibbs Free Energy= 0.600098
Sum of electronic and zero-point Energies= -2172.474835
Sum of electronic and thermal Energies= -2172.434357
Sum of electronic and thermal Enthalpies= -2172.433413
Sum of electronic and thermal Free Energies= -2172.546182
E(RM06L) = -2174.76666856

IId

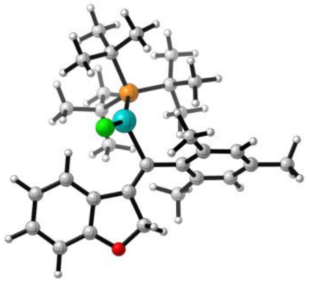

Pd	0.06748300	-0.54006800	1.07331200
C	-3.03809400	-1.18010300	-0.25118500
C	-2.51480600	-2.45675800	-0.02517600
C	-4.34100200	-1.06716700	-0.76796200
C	-3.29049100	-3.57982000	-0.32270700
H	-1.52965900	-2.56853100	0.41353900
C	-5.12913800	-2.17106900	-1.07394700
C	-4.58221900	-3.43653200	-0.84428400
H	-2.89108300	-4.57260500	-0.13593400
H	-6.13073100	-2.03946800	-1.46996900
H	-5.17446500	-4.32011800	-1.06751300
P	2.03053900	-0.77074500	-0.31867400
C	1.75478400	-1.92513800	-1.85753600
C	3.04164300	0.79956800	-0.86060000
C	3.08498100	-1.74247500	1.01330600
C	-2.50453500	0.18165200	-0.11701300
C	-1.30382300	0.69237500	0.21494800
O	-4.74569200	0.22068200	-0.95132000
C	-3.71006700	1.08096100	-0.43787100
H	-3.51684100	1.85576200	-1.18117500
H	-4.08511400	1.56091900	0.47547600
C	2.14601800	-2.71907800	1.76723900
H	1.69315500	-3.47701400	1.12980500
H	2.73519400	-3.24169000	2.53283800
H	1.33828200	-2.20428200	2.30673500
C	3.61041800	-0.76285600	2.08787700
H	4.03505200	-1.35328500	2.90980900
H	4.40236700	-0.10653600	1.72239200
H	2.80951700	-0.14739600	2.51021900
C	4.27834000	-2.54204400	0.45217100
H	4.82327400	-2.99158600	1.29321700
H	3.96522400	-3.36196500	-0.19838600
H	4.98672500	-1.91986500	-0.09914500
C	1.29573800	-3.32512700	-1.39309400
H	0.98504300	-3.89299700	-2.27927200
H	2.08710300	-3.89894400	-0.90645800
H	0.43176800	-3.27170900	-0.72450900
C	0.57901700	-1.35008100	-2.67989500
H	0.78835200	-0.36837600	-3.10445900
H	0.37253200	-2.03108300	-3.51594700
H	-0.32949400	-1.27976600	-2.07497900
C	2.98269200	-2.09852000	-2.77595900
H	3.28523500	-1.16633400	-3.25828800
H	3.84901000	-2.50820400	-2.25106800
H	2.72295700	-2.80334500	-3.57695500
C	4.54929400	0.55749800	-1.08486400
H	4.74914900	-0.20582300	-1.84104800
H	5.00047100	1.49378100	-1.43956800
H	5.07527000	0.27867400	-0.16944300
C	2.85506400	1.88740300	0.22319100
H	3.35948000	2.80216600	-0.11520900
H	1.80033200	2.12614600	0.37582100
H	3.28707300	1.61627900	1.18638100
C	2.45462500	1.38431200	-2.16235800
H	2.63221800	0.75356200	-3.03597100
H	1.38561700	1.57905200	-2.07374200
H	2.94448300	2.34750900	-2.35467300
C	-0.93862000	2.12012100	0.10144000
C	-0.84873500	2.73466600	-1.18120300
C	-0.70459500	2.92645500	1.25626400
C	-0.50135800	4.08819200	-1.27778800
C	-0.36668300	4.27064100	1.10014100
C	-0.24936200	4.87625000	-0.15647200
H	-0.43533900	4.53782900	-2.26714300
H	-0.19941800	4.86932800	1.99363800
C	-1.16152900	2.01367300	-2.47574200
H	-0.49111500	2.34490700	-3.27676700
H	-1.08895500	0.93067500	-2.38109000
H	-2.18129800	2.23986200	-2.81549500
C	-0.82808600	2.37609100	2.65460500
H	-1.74350000	1.79496200	2.78950900
H	-0.00886600	1.68788100	2.89363300
H	-0.81420700	3.18880500	3.38825400
C	0.11969800	6.33510300	-0.28214800
H	-0.62659200	6.97766200	0.20218200
H	1.08269200	6.54720700	0.19912200
H	0.19359100	6.64146500	-1.33043800
Cl	-1.27095700	-1.03610800	2.97530100

Zero-point correction= 0.674121 (Hartree/Particle)
Thermal correction to Energy= 0.714530
Thermal correction to Enthalpy= 0.715474
Thermal correction to Gibbs Free Energy= 0.602391
Sum of electronic and zero-point Energies= -2172.543742
Sum of electronic and thermal Energies= -2172.503332
Sum of electronic and thermal Enthalpies= -2172.502388
Sum of electronic and thermal Free Energies= -2172.615471
E(RM06L) = -2174.83832254

TS_RE_IId

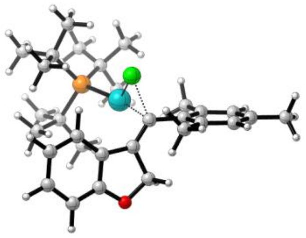

Pd	-0.16009100	-0.35486300	0.20485200
C	1.82744000	0.13769400	0.23426100
P	-2.55248600	-0.67547600	-0.06388800
C	-2.98827000	-2.52276700	-0.50565400
C	-3.49988200	-0.20715300	1.57417900
C	-3.20639400	0.46973400	-1.49980500
C	1.75092200	1.37155200	-0.36216500
C	1.09640600	2.64467500	-0.05374400
C	2.58129100	1.66207900	-1.62128900
C	1.44592600	3.54526100	-1.07871900
H	3.65990200	1.62718400	-1.41858000
H	2.36470400	0.97842000	-2.44639200
O	2.23825900	2.99602700	-2.04166500
C	0.31565100	3.10926100	1.01438800
C	1.02567300	4.87005600	-1.09367900
H	1.31644200	5.52972100	-1.90457700
C	-0.10837500	4.43967100	1.01798700
C	0.23523900	5.30768800	-0.02735100
H	0.05465800	2.44128700	1.82498000
H	-0.71022500	4.80484000	1.84527200
H	-0.10789500	6.33858700	-0.00749500
C	-2.82903600	1.05573000	2.16550100
H	-3.31765200	1.29574300	3.11984000
H	-1.76856000	0.87629700	2.36552700
H	-2.91072300	1.93492500	1.52641100

C	-5.01499000	0.04261400	1.43132600
H	-5.54873000	-0.81562300	1.01505200
H	-5.43814800	0.23917300	2.42602300
H	-5.23948800	0.91488600	0.81243800
C	-3.28372000	-1.31521400	2.62911400
H	-2.22419800	-1.56412000	2.74665500
H	-3.64204900	-0.94372400	3.59809600
H	-3.84177200	-2.22895900	2.41207300
C	-2.12814800	-3.44288000	0.39466000
H	-2.41856100	-3.41426400	1.44458500
H	-2.24163600	-4.48020900	0.05150300
H	-1.06821900	-3.17883300	0.33423600
C	-4.46876900	-2.92985400	-0.35957600
H	-4.58526200	-3.97373200	-0.68242200
H	-4.81646400	-2.87607300	0.67488500
H	-5.13726400	-2.32262100	-0.97471700
C	-2.54612100	-2.81926300	-1.95606700
H	-1.50588900	-2.52844700	-2.13409000
H	-2.62074700	-3.90085300	-2.12974400
H	-3.17671800	-2.33130600	-2.70249500
C	-2.18063000	0.41374000	-2.65807800
H	-2.11290500	-0.56549300	-3.13321700
H	-2.48236000	1.13340900	-3.43106800
H	-1.18064200	0.69185000	-2.30856200
C	-3.20824200	1.93974100	-1.02266100
H	-3.39729900	2.58499200	-1.89071300
H	-3.99145500	2.15129900	-0.29136600
H	-2.24309600	2.23566200	-0.59985700
C	-4.60608800	0.13469300	-2.05387100
H	-4.87894600	0.88332600	-2.81034800
H	-4.64011000	-0.84091200	-2.54576000
H	-5.38090900	0.15394000	-1.28342500
C	2.97731500	-0.77962700	0.10155400
C	4.24020800	-0.36823000	0.62768100
C	2.88559500	-2.02624200	-0.56473300
C	5.35026400	-1.19385800	0.45985600
C	4.03849400	-2.80541800	-0.72498700
C	5.27642100	-2.41739400	-0.21689800
H	6.30581700	-0.87241100	0.86947300
H	3.95726200	-3.75115200	-1.25703200
C	4.41842300	0.93278900	1.37807100
H	4.31423600	1.80760500	0.72458000
H	3.67583500	1.03842400	2.17323600
H	5.41619700	0.97798900	1.82508000
C	1.59369600	-2.57746100	-1.11457700
H	0.91154600	-1.78693200	-1.45682000
H	1.78475500	-3.25683500	-1.95314300
H	1.05846800	-3.14810900	-0.34625400
C	6.49885000	-3.29006200	-0.37088200
H	6.79389600	-3.73026300	0.59063300
H	6.31938400	-4.11262400	-1.07036700
H	7.35781700	-2.71482200	-0.73643100
Cl	1.18514800	0.05276900	2.38468700

Zero-point correction= 0.672122 (Hartree/Particle)
Thermal correction to Energy= 0.712375
Thermal correction to Enthalpy= 0.713319
Thermal correction to Gibbs Free Energy= 0.598657
Sum of electronic and zero-point Energies= -2172.521907
Sum of electronic and thermal Energies= -2172.481655
Sum of electronic and thermal Enthalpies= -2172.480711
Sum of electronic and thermal Free Energies= -2172.595373
E(RM06L) = -2174.80193783

***cis*-2d**

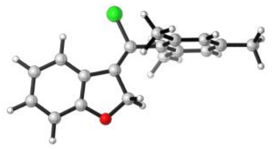

C	2.43121400	0.01066400	-0.00000500
C	0.75146800	-1.66857100	0.00013800
C	2.98709800	-1.28225700	0.00006200
H	0.21149800	-2.01715800	0.88765600
H	0.21142700	-2.01729000	-0.88728400
O	2.05851800	-2.27741200	0.00013100
C	3.28839200	1.11854700	-0.00007500
C	4.35864800	-1.51001000	0.00005800
H	4.74969100	-2.52192500	0.00011000
C	4.66883800	0.90770500	-0.00007800
C	5.19492600	-0.39063600	-0.00001300
H	2.88772300	2.12442400	-0.00012500
H	5.34043100	1.76113000	-0.00013000
H	6.27218800	-0.53376600	-0.00001700
C	0.97757300	-0.15636600	0.00002200
C	-0.05728700	0.70127500	-0.00003100
C	-1.48680400	0.29438800	0.00002200
C	-2.16495400	0.10537300	1.22416200
C	-2.16499900	0.10513300	-1.22413500
C	-3.50581100	-0.28948200	1.19972700
C	-3.50579000	-0.28970300	-1.19962900
C	-4.19335100	-0.49814400	0.00010300
H	-4.02799300	-0.43244000	2.14373100
H	-4.02798300	-0.43283200	-2.14361200
C	-1.46971200	0.33958500	2.54643100
H	-0.59159600	-0.30586600	2.66718500
H	-1.11437700	1.37313300	2.63281000
H	-2.14764600	0.14412400	3.38278200
C	-1.46971000	0.33911300	-2.54642100
H	-1.11425800	1.37261000	-2.63291500
H	-0.59166300	-0.30645100	-2.66708300
H	-2.14765300	0.14362400	-3.38275900
C	-5.63344500	-0.95549900	-0.00002100
H	-5.70131500	-2.05181000	-0.00764300
H	-6.16604000	-0.60394900	0.89005600
H	-6.16980500	-0.59162300	-0.88290900
Cl	0.21302800	2.46051000	-0.00016500

Zero-point correction= 0.300233 (Hartree/Particle)
Thermal correction to Energy= 0.319172
Thermal correction to Enthalpy= 0.320116
Thermal correction to Gibbs Free Energy= 0.251007
Sum of electronic and zero-point Energies= -1231.256389
Sum of electronic and thermal Energies= -1231.237450
Sum of electronic and thermal Enthalpies= -1231.236506
Sum of electronic and thermal Free Energies= -1231.305615
E(RM06L) = -1231.75556271

TS_Isom_IId

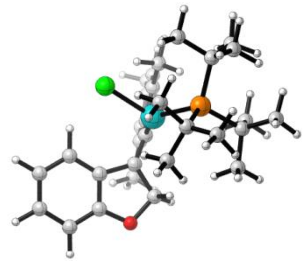

Pd	-0.15911900	-0.05219600	-0.42331800
C	2.21830500	-2.22149500	-0.11329000
C	2.60009400	-2.37752700	-1.44908400
C	2.42407700	-3.26503700	0.80354700

C	3.19773300	-3.57975000	-1.83241100
H	2.38622000	-1.59807800	-2.17248700
C	3.01831000	-4.46648000	0.43768400
C	3.40467400	-4.60616200	-0.89993500
H	3.48984800	-3.72653800	-2.86779900
H	3.16690100	-5.25712000	1.16570200
H	3.86554700	-5.53640100	-1.22127000
P	-2.60383000	0.26682900	0.19300200
C	1.63734000	-1.09769700	0.63314500
C	1.62592600	0.24051400	0.27014600
C	2.54313600	1.33769800	0.18466900
C	2.21510000	2.51933500	-0.55728000
C	3.82690200	1.27396200	0.83045600
C	3.13237400	3.56616400	-0.62771000
C	4.69675300	2.35508300	0.72500900
C	4.37887100	3.51056600	0.00055400
H	2.86679000	4.45317500	-1.19817800
H	5.66125800	2.29652200	1.22460100
C	-3.56411700	1.58262600	-0.88398600
C	-2.73274000	0.82742900	2.06060300
C	-3.48838700	-1.45954600	-0.00137800
C	-3.13359000	1.41786600	-2.35973600
H	-2.05292800	1.51118900	-2.48426400
H	-3.41790900	0.45926000	-2.79058000
H	-3.62174000	2.20666700	-2.94871100
C	-3.14409400	3.01332000	-0.47881800
H	-3.56556900	3.71300200	-1.21210000
H	-3.51838500	3.31485500	0.50175700
H	-2.05830800	3.14541700	-0.49507500
C	-5.10341000	1.49562900	-0.80345200
H	-5.48726200	1.60667200	0.21325600
H	-5.53094100	2.30942100	-1.40503600
H	-5.48953500	0.56056100	-1.21448700
C	-3.76064600	-1.74510900	-1.49591300
H	-4.07913300	-2.79226900	-1.58602900
H	-4.56740400	-1.13228000	-1.90478100
H	-2.86354800	-1.61548100	-2.10804900
C	-4.81149000	-1.61957500	0.77561900
H	-5.23320500	-2.60740900	0.54585300
H	-4.67857100	-1.57473600	1.85937800
H	-5.55937400	-0.87445600	0.49324200
C	-2.49136700	-2.55064300	0.45914600
H	-1.57435900	-2.52060200	-0.13743400
H	-2.22274900	-2.47779900	1.51455200
H	-2.95680900	-3.53381000	0.30840800
C	-2.33074800	-0.33818500	2.98932800
H	-1.35448400	-0.74991900	2.72145100
H	-2.25346900	0.04486200	4.01540000
H	-3.05889300	-1.15125900	3.00343300
C	-1.68257300	1.93763700	2.30643200
H	-1.88549400	2.85637900	1.75542600
H	-1.68216000	2.19171000	3.37509400
H	-0.67774400	1.59582700	2.03799500
C	-4.11663000	1.33740000	2.51640900
H	-4.07270600	1.56551200	3.59027100
H	-4.41597100	2.25686700	2.00894900
H	-4.90703400	0.59664300	2.37347300
C	4.27644000	0.08799300	1.64892800
H	3.67800000	-0.02837300	2.56029300
H	4.20940800	-0.85053400	1.09140200
H	5.31678800	0.21797600	1.96082200
C	0.91165500	2.66057500	-1.29061100
H	0.80105800	1.90043000	-2.07435900
H	0.06047900	2.53105400	-0.60819300
H	0.82961400	3.64899100	-1.75479600
C	5.36575900	4.64427300	-0.12031500
H	4.86468700	5.59079200	-0.34708300
H	5.94418000	4.77307500	0.80104600
H	6.08447200	4.45258400	-0.92878400
C	1.36664300	-1.67347100	2.02947900
Cl	-0.41541800	-0.80051100	-2.77840000
H	1.77484300	-1.05546400	2.83602500
H	0.29911300	-1.82514500	2.21143500
O	2.00546100	-2.96610800	2.06965100

Zero-point correction= 0.673142 (Hartree/Particle)
Thermal correction to Energy= 0.712829
Thermal correction to Enthalpy= 0.713773
Thermal correction to Gibbs Free Energy= 0.602494
Sum of electronic and zero-point Energies= -2172.521424
Sum of electronic and thermal Energies= -2172.481738
Sum of electronic and thermal Enthalpies= -2172.480793
Sum of electronic and thermal Free Energies= -2172.592072
E(RM06L) = -2174.81384629

IIId

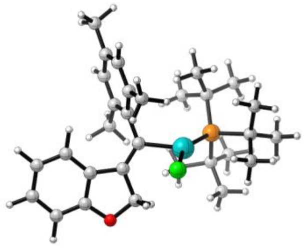

Pd	-0.59329400	-0.79744400	-1.02775100
C	3.51180800	-1.02868100	0.34236200
C	4.44378400	0.01410500	0.30950400
C	3.94066500	-2.31629600	0.71149500
C	5.77046600	-0.24470000	0.66524700
H	4.14440200	1.01104200	0.00425900
C	5.25602800	-2.59092700	1.07320900
C	6.16786100	-1.53211600	1.04780400
H	6.49939600	0.56016000	0.63896100
H	5.55051800	-3.59731600	1.35223300
H	7.20336800	-1.71650500	1.32152100
P	-2.38918100	-0.11633500	0.43534800
C	-2.49804800	-1.22274100	2.02907000
C	-2.65192800	1.74805900	0.92165300
C	-3.82799600	-0.63609200	-0.78740400
C	2.07454300	-1.12774700	0.02586500
C	1.21301600	-0.15473500	-0.32062300
O	2.95585900	-3.24981800	0.67130500
C	1.76736700	-2.61871700	0.13542700
H	1.54684900	-3.05662100	-0.84279700
H	0.93852000	-2.83812800	0.81651600
C	-3.40794300	-1.93411800	-1.52397000
H	-3.25550400	-2.78795800	-0.86564200
H	-4.20530000	-2.20184200	-2.23038300
H	-2.49915400	-1.80774500	-2.12910200
C	-3.98793700	0.43151400	-1.89415000
H	-4.66469200	0.03081700	-2.65957400
H	-4.42710500	1.36395300	-1.53491700
H	-3.03689200	0.65669100	-2.38740300
C	-5.20109000	-0.87138000	-0.12626300
H	-5.93458400	-1.08950100	-0.91414100
H	-5.19748300	-1.72928800	0.55049100
H	-5.56477100	-0.00164800	0.42530100
C	-2.62893400	-2.70575500	1.61622900
H	-2.50058200	-3.32557300	2.51268200
H	-3.60752600	-2.94867700	1.19692100
H	-1.85535500	-3.00006200	0.89922100
C	-1.15545600	-1.11170800	2.78666500

H	-0.97551200	-0.12078800	3.20320200
H	-1.16681800	-1.82099200	3.62445800
H	-0.31235100	-1.36933000	2.13995800
C	-3.64885500	-0.87836000	2.99734400
H	-3.54534100	0.11723000	3.43537100
H	-4.63290500	-0.94395400	2.52780700
H	-3.63455700	-1.59659100	3.82794000
C	-4.11419400	2.13916700	1.22251500
H	-4.55245500	1.55015900	2.03221200
H	-4.13274900	3.19099500	1.53762000
H	-4.76445000	2.05774200	0.34915400
C	-2.11433600	2.62521400	-0.23317300
H	-2.18818700	3.67810300	0.06956200
H	-1.06422400	2.41282700	-0.44463100
H	-2.67880000	2.51379300	-1.15889200
C	-1.80330100	2.09775900	2.16240800
H	-2.17309700	1.63180300	3.07843100
H	-0.75281000	1.83741200	2.02724600
H	-1.85076900	3.18399300	2.31196300
C	1.44389800	1.29751000	-0.31370300
C	1.69489600	1.97403900	0.91564900
C	1.45353000	2.05389100	-1.52326100
C	1.90628300	3.35750200	0.90921200
C	1.67608100	3.43045100	-1.47015300
C	1.89305000	4.10974900	-0.26597000
H	2.09950400	3.85800900	1.85625200
H	1.69172700	3.98951000	-2.40382100
C	1.82365000	1.25185800	2.23739100
H	1.61167700	1.92993900	3.07107700
H	1.16198100	0.38811600	2.30916900
H	2.84543100	0.87560100	2.37234700
C	1.25457500	1.40548300	-2.86975300
H	1.86920600	0.50941500	-2.98726800
H	0.21935100	1.07195700	-3.01032000
H	1.49678900	2.10831200	-3.67362300
C	2.10416000	5.60476400	-0.24583400
H	2.81108500	5.91908100	-1.02261400
H	1.16473800	6.14225900	-0.43242600
H	2.49095900	5.94272500	0.72093400
Cl	0.30452700	-1.97382800	-2.90283000

Zero-point correction= 0.674161 (Hartree/Particle)
Thermal correction to Energy= 0.714542
Thermal correction to Enthalpy= 0.715486
Thermal correction to Gibbs Free Energy= 0.603103
Sum of electronic and zero-point Energies= -2172.549296
Sum of electronic and thermal Energies= -2172.508915
Sum of electronic and thermal Enthalpies= -2172.507970
Sum of electronic and thermal Free Energies= -2172.620354
E(RM06L) = -2174.84231267

TS_RE_IIId

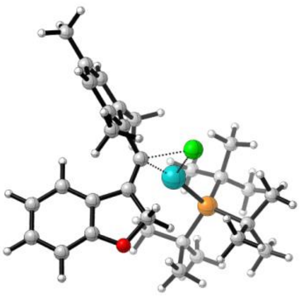

Pd	-0.44674200	-0.32314800	0.28883900
C	1.56779400	-0.16538100	0.61020200
P	-2.81425400	-0.16192500	-0.19079100
C	-3.52824300	-1.84052500	-0.87714600
C	-3.78819300	0.29217200	1.43569800
C	-3.14228600	1.23142800	-1.51391000
C	1.65653400	1.20454800	0.57608800
C	2.50685300	2.02006400	-0.29281500
C	1.05436600	2.20312400	1.56825300
C	2.30154200	3.36466900	0.06933700
H	-0.02599400	2.17350800	1.68786000
H	1.51644300	2.05812500	2.55476700
O	1.41084500	3.51891600	1.08381500
C	3.37506300	1.72027400	-1.35034700
C	2.94476900	4.41674100	-0.57375300
H	2.76810900	5.44213900	-0.26616900
C	4.02774000	2.76534800	-2.00649000
C	3.81333100	4.09643100	-1.62139600
H	3.53801800	0.69093600	-1.65246300
H	4.70677900	2.54435300	-2.82483700
H	4.32937300	4.89643300	-2.14540300
C	-2.95547400	1.33925700	2.21467800
H	-3.46838400	1.56048300	3.16056500
H	-1.96316900	0.94569900	2.45637700
H	-2.83342500	2.28385900	1.68357100
C	-5.22021200	0.82995300	1.23930300
H	-5.86100100	0.13608400	0.68933000
H	-5.67946500	0.98734300	2.22485500
H	-5.24019000	1.79276600	0.72218600
C	-3.85007700	-0.94498700	2.35965400
H	-2.86148800	-1.39071800	2.50878900
H	-4.21619400	-0.62310600	3.34338900
H	-4.53720300	-1.71437400	2.00026900
C	-2.90686600	-2.99371300	-0.05167900
H	-3.24836800	-3.01903500	0.98275800
H	-3.18774600	-3.94990800	-0.51369200
H	-1.81434000	-2.93056600	-0.03930700
C	-5.06364300	-1.98492400	-0.85952600
H	-5.33719100	-2.94743300	-1.31355000
H	-5.47209200	-1.98355200	0.15387200
H	-5.56780700	-1.20096300	-1.43015800
C	-3.03970200	-2.05171600	-2.32767200
H	-1.95481200	-1.93546800	-2.41533500
H	-3.28693000	-3.07713100	-2.63257000
H	-3.52109500	-1.38093600	-3.04273400
C	-2.05670600	1.11172400	-2.61115500
H	-2.13150600	0.19643100	-3.19897100
H	-2.16238600	1.95636300	-3.30535200
H	-1.05258600	1.15461600	-2.17585300
C	-2.91890700	2.61568900	-0.86475400
H	-2.92509200	3.37627800	-1.65644700
H	-3.70137700	2.88769700	-0.15309500
H	-1.94833600	2.67684300	-0.36164200
C	-4.53309100	1.22411400	-2.18031500
H	-4.60949200	2.08502700	-2.85862800
H	-4.70355500	0.32840600	-2.78315800
H	-5.34817500	1.30644700	-1.45700000
C	2.60295800	-1.13727900	0.23232200
C	3.87684100	-1.06313300	0.87378100
C	2.38934000	-2.13350400	-0.75500400
C	4.88534000	-1.94586700	0.49156100
C	3.44451600	-2.97825800	-1.11683400
C	4.69672800	-2.90869900	-0.50654500
H	5.85113900	-1.88055100	0.98866300
H	3.27420000	-3.72202600	-1.89242600
C	4.17414300	-0.05805000	1.96219500
H	4.29799100	0.95198700	1.55304900
H	3.37008500	-0.02294000	2.70264100
H	5.10395600	-0.32042200	2.47639400
C	1.06181800	-2.33642800	-1.43961500
H	0.56554200	-1.38407300	-1.67612200

H	1.18409000	-2.90101800	-2.37078100
H	0.37610300	-2.90295800	-0.79798600
C	5.80856800	-3.85671900	-0.88630800
H	5.96214800	-4.61650100	-0.10856700
H	5.58720300	-4.38155300	-1.82107100
H	6.76088300	-3.32791500	-1.00990300
Cl	0.69620300	-0.81731900	2.59598600

Zero-point correction= 0.672434 (Hartree/Particle)
Thermal correction to Energy= 0.712521
Thermal correction to Enthalpy= 0.713465
Thermal correction to Gibbs Free Energy= 0.599734
Sum of electronic and zero-point Energies= -2172.525332
Sum of electronic and thermal Energies= -2172.485246
Sum of electronic and thermal Enthalpies= -2172.484302
Sum of electronic and thermal Free Energies= -2172.598033
E(RM06L) = -2174.80513490

***trans*-2d**

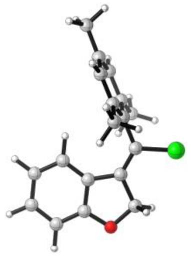

C	-1.80976600	-0.47876700	0.00002100
C	-2.50906400	1.78874000	-0.00003300
C	-3.21186000	-0.37000500	0.00001100
H	-2.56048500	2.42966400	0.88749400
H	-2.56047700	2.42963600	-0.88758000
O	-3.65870800	0.91536500	-0.00002400
C	-1.21976800	-1.74809000	0.00006000
C	-4.04653000	-1.48251600	0.00003600
H	-5.12489100	-1.36382500	0.00002800
C	-2.04397600	-2.87552600	0.00008500
C	-3.43891000	-2.74110100	0.00007300
H	-0.14059100	-1.85502900	0.00006800
H	-1.59822900	-3.86580900	0.00011400
H	-4.06354600	-3.63034800	0.00009300
C	-1.28331300	0.88854900	-0.00001200
C	-0.01019200	1.31532700	-0.00002100
C	1.22363800	0.49459200	-0.00001300
C	1.81103200	0.10865000	-1.22456800
C	1.81110000	0.10877100	1.22454500
C	2.97388700	-0.66692400	-1.20004100
C	2.97395300	-0.66680400	1.20003100
C	3.57339700	-1.06267300	-0.00000100
H	3.42336300	-0.96880600	-2.14401000
H	3.42348100	-0.96859300	2.14400600
C	1.19569900	0.51107800	-2.54524200
H	0.18897300	0.09264700	-2.66271200
H	1.10125800	1.60002600	-2.62886900
H	1.80617000	0.15935900	-3.38250200
C	1.19583300	0.51132200	2.54521100
H	1.10136900	1.60027600	2.62873100
H	0.18912200	0.09288000	2.66277700
H	1.80635600	0.15970300	3.38247400
C	4.84908600	-1.87243000	0.00000200
H	4.91873700	-2.51154300	0.88673400
H	4.91867700	-2.51164500	-0.88666100
H	5.73217300	-1.21930200	-0.00006500
Cl	0.26486700	3.08439000	-0.00004700

Zero-point correction= 0.300347 (Hartree/Particle)
Thermal correction to Energy= 0.319184
Thermal correction to Enthalpy= 0.320128
Thermal correction to Gibbs Free Energy= 0.251394
Sum of electronic and zero-point Energies= -1231.258841
Sum of electronic and thermal Energies= -1231.240004
Sum of electronic and thermal Enthalpies= -1231.239060
Sum of electronic and thermal Free Energies= -1231.307794
E(RM06L) = -1231.75899322

l) potential side-reactions of carbamoyl chloride **3a** (R = TIPS), L = PA-Ph

TS_OA_C-Si_3a

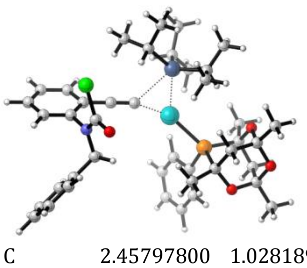

C	2.45797800	1.02818900	1.02036200
C	1.24890400	1.11079000	0.80900200
C	3.86252200	0.91926500	1.22138900
C	4.67737700	0.23807600	0.28364900
C	4.49192400	1.48486000	2.34947200
C	6.05259000	0.12246400	0.48016600
C	5.86786600	1.37912300	2.53242200
H	3.87939800	2.00978800	3.07583700
C	6.65421900	0.69860000	1.59938300
H	6.64266800	-0.41677500	-0.25387600
H	6.32846300	1.82743000	3.40866100
H	7.72727900	0.61163500	1.74260200
N	4.06193600	-0.40933300	-0.83973000
C	3.46116900	-1.74868000	-0.60906300
H	2.98114100	-1.71560500	0.37239100
H	2.68299400	-1.87489900	-1.36378700
C	4.47082400	-2.87578100	-0.68081100
C	4.89768300	-3.52847000	0.48119500
C	4.99398300	-3.28051700	-1.91861500
C	5.82930900	-4.56750300	0.41364900
H	4.49750600	-3.22197300	1.44499600
C	5.92676000	-4.31467500	-1.98704400
H	4.66341800	-2.77928100	-2.82450100
C	6.34645200	-4.96164600	-0.82079900
H	6.14997900	-5.06616100	1.32463000
H	6.32301200	-4.62067500	-2.95178800
H	7.07084900	-5.77002800	-0.87614400
C	3.91653500	0.12704200	-2.07620200
O	3.40250900	-0.39135100	-3.03503900
Cl	4.60487600	1.80638200	-2.22642100
Si	-0.44995100	3.03422000	0.19766900
C	1.10654200	3.93772200	-0.52971100
C	-1.92334100	3.07786700	-1.07065900
C	-0.86087200	3.77268400	1.93458500
H	1.58378000	3.19725900	-1.18499400
C	0.77440400	5.17636900	-1.39400800
C	2.15348900	4.35516000	0.52492200
H	-2.64198700	2.33878900	-0.69215400
C	-1.48993200	2.59861500	-2.47111700
C	-2.68563200	4.41833600	-1.15657100

H	0.04644700	3.54803300	2.51461900
C	-2.04358800	3.10438500	2.65460700
C	-1.04728400	5.30623200	1.94499600
H	0.27472000	5.96176800	-0.81384600
H	1.70460000	5.61157100	-1.78408000
H	0.14164900	4.94667300	-2.25460200
H	2.42125500	3.53799700	1.19567600
H	3.07264000	4.68848300	0.02524200
H	1.79767000	5.19420500	1.13448600
H	-2.36310200	2.48283100	-3.12880100
H	-0.81554700	3.30635100	-2.96359500
H	-0.96883600	1.63496600	-2.42846100
H	-2.04569100	5.24950900	-1.46948500
H	-3.49898100	4.33929900	-1.89231700
H	-3.14335900	4.69038200	-0.19980200
H	-1.91097300	2.01984700	2.72845900
H	-2.15674500	3.50342600	3.67254900
H	-2.98915800	3.29182900	2.12945500
H	-1.96318600	5.60798900	1.42534100
H	-1.13144000	5.66768600	2.97957400
H	-0.21351300	5.84089800	1.48138600
C	-2.75645400	-1.88133100	-1.23301900
C	-4.42233100	-0.43220800	0.42582200
C	-3.19914400	-0.87124700	-2.30605600
C	-5.28785700	-1.69906500	0.39445900
H	-3.21005700	-1.38189700	-3.27631800
H	-2.50279400	-0.03164200	-2.36472200
C	-4.62198800	-0.37394800	-2.00882500
C	-5.12134900	-2.42653200	-0.94703200
H	-5.04169000	-2.36080000	1.22943000
H	-6.33930100	-1.40244900	0.49091400
C	-2.28653300	-2.10142600	1.74510600
C	-2.60173400	-3.47218400	1.68726900
C	-1.69008900	-1.60001900	2.91726200
C	-2.33065000	-4.30665800	2.77290600
H	-3.05241000	-3.88397300	0.79207100
C	-1.43398700	-2.43559000	4.00553100
H	-1.40866500	-0.55124600	2.96899600
C	-1.75230200	-3.79235800	3.93527200
H	-2.57340500	-5.36409200	2.70775800
H	-0.97502200	-2.02570900	4.90105700
H	-1.54547700	-4.44664000	4.77791300
P	-2.56358100	-0.88541900	0.37924400
C	-1.47155900	-2.60343200	-1.62095800
H	-1.63155600	-3.15228800	-2.55577600
H	-0.66169200	-1.88183800	-1.76925300
H	-1.16557000	-3.31676500	-0.85018200
C	-4.76053700	0.46658300	1.60454900
H	-4.16176600	1.38000000	1.57994900
H	-5.82068000	0.74212900	1.56335300
H	-4.57019800	-0.05189100	2.55030200
C	-5.17609400	0.56983700	-3.05922200
H	-4.56300100	1.47305100	-3.11653700
H	-5.18614100	0.07810500	-4.03579200
H	-6.19850200	0.84840000	-2.79012000
C	-6.01660700	-3.64235600	-1.09940000
H	-7.06597300	-3.34559400	-1.01964100
H	-5.84789600	-4.09472300	-2.08025100
H	-5.79173700	-4.37932000	-0.32291400
O	-5.47911200	-1.51155700	-1.97701000
O	-4.66950400	0.33932300	-0.76417800
O	-3.77854000	-2.89421200	-1.12013100
Pd	-0.60573200	0.61452000	0.49989100

Zero-point correction= 0.859748 (Hartree/Particle)
Thermal correction to Energy= 0.913696
Thermal correction to Enthalpy= 0.914640
Thermal correction to Gibbs Free Energy= 0.765767
Sum of electronic and zero-point Energies= -3166.847442
Sum of electronic and thermal Energies= -3166.793493
Sum of electronic and thermal Enthalpies= -3166.792549
Sum of electronic and thermal Free Energies= -3166.941422
E(RM06L) = -3169.50218964

TS_OA_Ar-N_3a

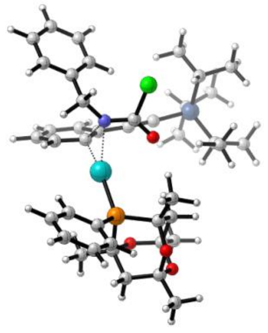

Pd	0.93999300	1.01805000	0.58332800
C	-0.74475200	1.80545600	1.30346300
C	-0.34099700	2.97614700	1.96170300
C	-1.83566800	1.03546800	1.78344200
C	-0.91959600	3.32955100	3.18356900
H	0.43137600	3.60435000	1.52704900
C	-2.40016800	1.42959200	3.01680500
C	-1.95072600	2.55054500	3.70999100
H	-0.57657800	4.21966000	3.70375000
H	-3.21587300	0.83146500	3.41169200
H	-2.42218400	2.82738700	4.64837300
P	3.08003200	0.04375000	0.03851100
C	3.01421700	-1.49831400	-1.06974200
C	3.82160600	-0.91842600	1.50910000
C	4.40331700	1.15177100	-0.60958900
C	2.01758800	-2.44858300	-0.38312200
C	2.60170600	-1.17148600	-2.49906300
O	4.29939700	-2.15500500	-1.14602700
C	5.10719300	-1.65097400	1.10846900
C	4.01264400	-0.02581300	2.72679500
O	2.81908700	-1.88784300	1.86970900
C	5.48187500	0.73750900	-1.41288500
C	4.29796200	2.51371600	-0.27709700
H	1.85473000	-3.31092600	-1.04049600
H	1.05639800	-1.95118900	-0.22374900
C	2.60492700	-2.95984500	0.94043100
H	2.60117400	-2.09436100	-3.09015900
H	1.59429800	-0.74563200	-2.50953300
H	3.29567500	-0.46566900	-2.96559500
C	4.79207600	-2.73546200	0.06693200
H	5.85332800	-0.94941400	0.72392100
H	5.52311000	-2.14154400	1.99688300
H	3.06480300	0.44242000	3.01229500
H	4.37168900	-0.62495200	3.57157300
H	4.74569100	0.76077300	2.51762300
C	6.42566000	1.66374800	-1.86021300
H	5.57298600	-0.30555200	-1.69365800
C	5.25185400	3.43425900	-0.71275200
H	3.45383900	2.85091300	0.32009400
C	1.71872600	-3.96406800	1.65247800
O	3.83466700	-3.61874000	0.64015100
C	5.99466700	-3.57539000	-0.32321700
C	6.31811200	3.01067000	-1.50780200
H	7.24810500	1.32962000	-2.48733600
H	5.15485200	4.48200400	-0.44095200
H	0.75961300	-3.50364900	1.90568100

H	1.54399700	-4.83034600	1.00848300
H	2.21098700	-4.29573000	2.57074900
H	6.40047600	-4.07908200	0.55847900
H	5.68744400	-4.32830400	-1.05391300
H	6.77112600	-2.94457600	-0.76591300
H	7.05724000	3.72688000	-1.85662900
N	-0.68335300	2.06212900	-0.74795100
C	-0.90880400	1.05881000	-1.60015200
C	-0.86757300	3.47743400	-1.06073200
O	-0.60950000	-0.11217800	-1.49518000
H	-0.75376000	3.60351300	-2.14321400
H	-0.03426700	4.02416300	-0.60821900
C	-2.17544100	4.11485300	-0.61387500
C	-2.20040200	5.49328100	-0.36279300
C	-3.36594300	3.38933100	-0.49977900
C	-3.38778700	6.13702800	-0.01407500
H	-1.28033800	6.06989800	-0.44407300
C	-4.55429300	4.02991600	-0.14307700
H	-3.36429900	2.32054500	-0.68573900
C	-4.57151500	5.40460900	0.09810900
H	-3.38688800	7.20735500	0.17606600
H	-5.46968000	3.45017400	-0.05662100
H	-5.49817800	5.90103600	0.37385200
Cl	-1.79654500	1.53249800	-3.16780100
C	-2.40459700	-0.08716600	1.11483500
C	-2.99736700	-1.03013300	0.61150300
Si	-3.96633300	-2.42653000	-0.12360300
C	-2.70214300	-3.79948100	-0.57122600
C	-5.18402900	-2.93081200	1.27566100
C	-4.84809900	-1.68758300	-1.65814600
H	-2.03767800	-3.84442700	0.30581700
C	-3.29991800	-5.20728700	-0.76442500
C	-1.83621000	-3.40322200	-1.78453300
H	-5.63459400	-1.97408300	1.58265100
C	-4.44137500	-3.49500900	2.50334300
C	-6.33905600	-3.86126000	0.85660900
H	-4.03612900	-1.19560100	-2.21454200
C	-5.86028200	-0.59321600	-1.26549600
C	-5.49666500	-2.71609500	-2.60572200
H	-4.01710000	-5.23880100	-1.59341900
H	-2.50662300	-5.93063300	-0.99939800
H	-3.81473500	-5.56867500	0.13241200
H	-1.39014400	-2.40998900	-1.67482000
H	-1.02244500	-4.12842600	-1.92832400
H	-2.42555900	-3.39898200	-2.70998900
H	-5.13617500	-3.65921100	3.33869000
H	-3.96976200	-4.46076400	2.28233700
H	-3.65622600	-2.81453900	2.85211800
H	-5.97833000	-4.83134800	0.49583400
H	-7.00052600	-4.06122900	1.71122200
H	-6.95540000	-3.42281700	0.06429800
H	-5.40255000	0.17706600	-0.63421600
H	-6.26085700	-0.09572500	-2.15911400
H	-6.71448700	-1.00969100	-0.71614100
H	-6.31261500	-3.26618900	-2.12202800
H	-5.92448800	-2.21143500	-3.48293700
H	-4.77479300	-3.45252400	-2.97481000

Zero-point correction= 0.856412 (Hartree/Particle)
Thermal correction to Energy= 0.910871
Thermal correction to Enthalpy= 0.911815
Thermal correction to Gibbs Free Energy= 0.762658
Sum of electronic and zero-point Energies= -3166.807752
Sum of electronic and thermal Energies= -3166.753293
Sum of electronic and thermal Enthalpies= -3166.752349
Sum of electronic and thermal Free Energies= -3166.901506
E(RM06L) = -3169.45996348

TS_OA_N-Bn_3a

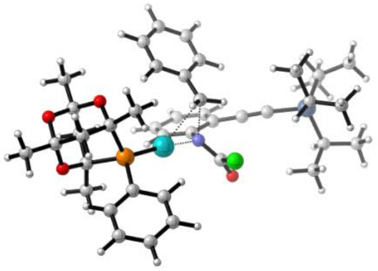

C	-2.65680300	1.46130100	1.87987200
C	-2.97756200	2.55504300	2.70776500
C	-1.30180100	1.03537300	1.79710600
C	-1.99402700	3.23535800	3.41763300
H	-4.01632700	2.86356600	2.77354300
C	-0.32511300	1.74225300	2.51039400
C	-0.66086100	2.83136000	3.31099600
H	-2.26556700	4.07701300	4.04839300
H	0.70378000	1.40083500	2.43051100
H	0.11638000	3.35509500	3.86063700
C	-1.53395600	-1.23009500	1.17325800
N	-0.90119100	-0.04338800	0.96522800
C	-0.66517700	2.06345300	-1.20962000
C	0.56288600	2.71175400	-1.48460900
C	-1.83620800	2.84766300	-1.10110000
C	0.60293200	4.08662300	-1.67400100
H	1.47676900	2.12660600	-1.54205200
C	-1.78490100	4.22738400	-1.28106600
H	-2.78292500	2.35968800	-0.89151000
C	-0.56818800	4.84970000	-1.56821300
H	1.54696000	4.57194000	-1.90531900
H	-2.69369700	4.81684400	-1.19952300
H	-0.52832400	5.92649400	-1.70952500
O	-2.20221000	-1.60879500	2.09594400
C	-0.75693700	0.62636700	-1.14211700
H	-1.74022100	0.18088700	-1.18611000
H	0.02519200	0.03497800	-1.60791200
Pd	1.17834900	-0.30779000	0.44010700
P	3.39102800	-0.69667300	0.02791600
C	4.63236900	0.22109000	1.14284100
C	4.16785200	0.02172400	-1.56544900
C	3.84589900	-2.48857500	0.06445800
C	4.35279200	1.71952700	0.92977600
C	4.50650700	-0.18744500	2.60525300
O	6.00485700	-0.03822900	0.76879900
C	5.66974800	-0.27969500	-1.64443100
C	3.42599200	-0.43358600	-2.81277700
O	3.98654600	1.45433500	-1.48232100
C	5.11256900	-3.00023800	0.40385800
C	2.81780500	-3.39542200	-0.25068300
H	4.95417000	2.29436300	1.64457200
H	3.29467200	1.94168200	1.09770900
C	4.76738100	2.13488900	-0.49049100
H	5.22503600	0.38111900	3.20678400
H	3.49493700	0.01742000	2.96930000
H	4.71138800	-1.25371700	2.73801200
C	6.41657600	0.44180100	-0.51358200
H	5.85272300	-1.35703100	-1.60376000
H	6.05424800	0.09722000	-2.60011500
H	2.36468000	-0.17586500	-2.74813500
H	3.85086700	0.05500500	-3.69737500
H	3.51227600	-1.51846100	-2.93465400
C	5.33901600	-4.37775600	0.41553200
H	5.91411000	-2.32178900	0.67174100

C	3.05331400	-4.77107900	-0.25259500
H	1.82346600	-3.01749900	-0.47418700
C	4.60147200	3.61912700	-0.76003700
O	6.15150900	1.83638400	-0.64404800
C	7.92363000	0.26992600	-0.56873000
C	4.31482900	-5.26604000	0.08106800
H	6.32061800	-4.75645000	0.68898100
H	2.24465300	-5.45334500	-0.50011100
H	3.55733300	3.91321700	-0.62146700
H	5.22907900	4.19397300	-0.07351000
H	4.90562500	3.83768600	-1.78737800
H	8.30993100	0.65979700	-1.51450700
H	8.38098300	0.82089200	0.25743300
H	8.18825000	-0.78791700	-0.48173600
H	4.49685200	-6.33749300	0.09007700
C	-3.69677600	0.83153600	1.13399000
C	-4.60518500	0.31914300	0.49554800
Si	-6.00578700	-0.55379100	-0.34665800
C	-5.24718000	-1.34781900	-1.92004700
C	-6.63518500	-1.83591800	0.93339200
C	-7.28371300	0.82439800	-0.74442200
H	-4.34298600	-1.85380500	-1.54909400
C	-6.11910800	-2.41832800	-2.60570400
C	-4.79175700	-0.28413800	-2.93881500
H	-6.73842300	-1.25086900	1.86023400
C	-5.58643200	-2.93436100	1.20407500
C	-8.01474300	-2.45262900	0.62775900
H	-6.68628300	1.60100700	-1.24724900
C	-7.85868400	1.45414500	0.53996200
C	-8.41442500	0.42424300	-1.71247400
H	-7.06508800	-2.00635400	-2.97707300
H	-5.59222500	-2.84499000	-3.47041500
H	-6.36042900	-3.24684500	-1.93156400
H	-4.12909400	0.46327200	-2.48608800
H	-4.24559100	-0.74959200	-3.77068500
H	-5.64452900	0.25295500	-3.37299400
H	-5.92285600	-3.59145500	2.01781800
H	-5.43093000	-3.56914300	0.32250600
H	-4.61475100	-2.52073600	1.49332300
H	-8.01421900	-3.02293100	-0.30857300
H	-8.30599700	-3.14813700	1.42690600
H	-8.80253900	-1.69477200	0.55318000
H	-7.06665500	1.79600700	1.21594700
H	-8.49104100	2.32046000	0.30124100
H	-8.48288100	0.74186000	1.09390300
H	-9.04520200	-0.37294200	-1.30247300
H	-9.07053900	1.28267100	-1.91331000
H	-8.02920400	0.07657000	-2.67721700
Cl	-1.26148600	-2.42386100	-0.23250500

Zero-point correction= 0.856307 (Hartree/Particle)
Thermal correction to Energy= 0.910770
Thermal correction to Enthalpy= 0.911714
Thermal correction to Gibbs Free Energy= 0.764816
Sum of electronic and zero-point Energies= -3166.822465
Sum of electronic and thermal Energies= -3166.768002
Sum of electronic and thermal Enthalpies= -3166.767058
Sum of electronic and thermal Free Energies= -3166.913956
E(RM06L) = -3169.48056617

TS_OA_CO-N_3a

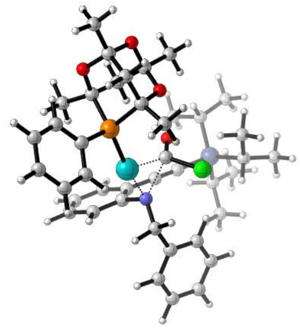

C	-1.95284200	-1.25554900	1.94703700
C	-2.14303700	-1.45815300	3.33174200
C	-0.93768300	-1.99176800	1.27550400
C	-1.38555900	-2.37852800	4.04589200
H	-2.91709100	-0.88308700	3.83053600
C	-0.19688200	-2.93430300	2.02280800
C	-0.41189600	-3.13192800	3.38291000
H	-1.56045700	-2.51706200	5.10908700
H	0.55980900	-3.52323800	1.50907800
H	0.18092800	-3.86442100	3.92450500
C	-0.09691900	0.07763500	-0.81118400
N	-0.67207300	-1.78995400	-0.10375300
C	-0.76574700	-2.96619200	-0.98720500
H	-0.12502400	-3.79766600	-0.65190600
H	-0.39072900	-2.65318500	-1.96622500
C	-2.18905500	-3.48383600	-1.13277500
C	-3.20076900	-2.65817400	-1.64214300
C	-2.50323100	-4.80323700	-0.79041600
C	-4.49401600	-3.15009500	-1.81519900
H	-2.96670700	-1.62871100	-1.89554500
C	-3.79900600	-5.29831000	-0.95990500
H	-1.72637600	-5.45142700	-0.38961100
C	-4.79770000	-4.47204000	-1.47487800
H	-5.26793500	-2.50155500	-2.21738100
H	-4.02569300	-6.32605400	-0.68747000
H	-5.80700200	-4.85245900	-1.60941000
O	-0.53478300	1.03119300	-0.25632400
Pd	1.34139500	-1.09088200	-0.09139600
P	3.42553000	0.01230200	-0.06356700
C	3.60082500	1.32386000	1.29783100
C	3.78705100	1.25036600	-1.46694100
C	4.85283700	-1.14549000	0.04855700
C	2.47821400	2.34418500	1.03899100
C	3.52395900	0.72662300	2.69772500
O	4.87150100	1.99862900	1.20394000
C	5.13904200	1.93767100	-1.22979700
C	3.68678700	0.59766100	-2.83787700
O	2.74071100	2.22824800	-1.39860300
C	6.06822600	-0.86247200	0.69846600
C	4.68586800	-2.40760100	-0.55068900
H	2.47912500	3.06817100	1.86249300
H	1.49842200	1.86115800	0.99981800
C	2.75046800	3.10747400	-0.26604400
H	3.62806500	1.52826400	3.43730200
H	2.55741000	0.23451400	2.84640600
H	4.31982400	-0.00442100	2.86804700
C	5.07733400	2.80855900	0.03457800
H	5.94689500	1.20364000	-1.15686300
H	5.34690400	2.59198400	-2.08474300
H	2.69232400	0.16765000	-2.98608700
H	3.85693000	1.35324400	-3.61330000
H	4.44005700	-0.19028200	-2.94656000
C	7.08213400	-1.82080400	0.74377500
H	6.21483500	0.10364800	1.16778900

C	5.70759400	-3.35724700	-0.51455100
H	3.74900500	-2.64478600	-1.05078200
C	1.72291900	4.18160100	-0.56249600
O	4.02189000	3.74429000	-0.13256800
C	6.34450600	3.60300300	0.29163100
C	6.90764900	-3.06617500	0.13634400
H	8.01306800	-1.59090700	1.25522100
H	5.56186600	-4.32419100	-0.98816900
H	0.73765000	3.72299800	-0.68080800
H	1.69191800	4.90681900	0.25546100
H	1.99431200	4.69766100	-1.48737000
H	6.54273500	4.27091700	-0.55105400
H	6.21809100	4.20142500	1.19774800
H	7.19602500	2.92914100	0.42416700
H	7.70184600	-3.80682600	0.17270400
Cl	-0.31761400	-0.09029500	-2.66505200
C	-2.83004900	-0.34312500	1.28814200
C	-3.65665700	0.43929800	0.84249000
Si	-4.94147500	1.63005700	0.25985800
C	-4.73513300	1.78771900	-1.64062600
C	-6.61355800	0.84151300	0.78768600
C	-4.56614200	3.27182600	1.18491400
H	-4.63004700	0.74654600	-1.98314400
C	-5.93647200	2.39587300	-2.39097500
C	-3.43007500	2.52147300	-2.01059500
H	-6.45097400	0.57378600	1.84324800
C	-6.88755600	-0.47092000	0.02675100
C	-7.84183300	1.77101200	0.73875200
H	-3.49594300	3.43841100	0.98891700
C	-4.73044100	3.11947500	2.70984200
C	-5.32269800	4.51364200	0.67401500
H	-6.13922300	3.42693100	-2.07709300
H	-5.73886900	2.42271800	-3.47167000
H	-6.85588200	1.81898400	-2.24221100
H	-2.55475700	2.07812600	-1.52546100
H	-3.26096300	2.48553800	-3.09549100
H	-3.47224600	3.58073200	-1.72587100
H	-7.77717000	-0.97597600	0.42844600
H	-7.07683400	-0.28595200	-1.03861400
H	-6.04676600	-1.16941100	0.09929300
H	-8.05232800	2.12481500	-0.27727000
H	-8.73920900	1.24028600	1.08691000
H	-7.71845000	2.65315800	1.37645700
H	-4.14746800	2.27663500	3.09778100
H	-4.39419800	4.02625500	3.23183400
H	-5.77928300	2.95694700	2.98960400
H	-6.40676300	4.42093100	0.80851100
H	-5.00624600	5.41037600	1.22528300
H	-5.13719600	4.70313200	-0.38876200

Zero-point correction= 0.856525 (Hartree/Particle)
Thermal correction to Energy= 0.910852
Thermal correction to Enthalpy= 0.911796
Thermal correction to Gibbs Free Energy= 0.763907
Sum of electronic and zero-point Energies= -3166.822379
Sum of electronic and thermal Energies= -3166.768053
Sum of electronic and thermal Enthalpies= -3166.767109
Sum of electronic and thermal Free Energies= -3166.914998
E(RM06L) = -3169.47544489

m) potential side-reactions of aryl chloride **4a** (R = TIPS), L = PA-Ph

TS_OA_C-Si_4a

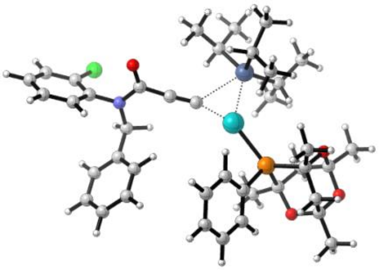

C	2.37483100	0.80937200	0.46355900
C	1.15625300	0.93730900	0.36655000
C	3.79968200	0.74516900	0.70571600
Si	-0.49966700	3.01221700	0.28474600
C	1.02395900	3.93846000	-0.48083900
C	-2.10246900	3.29745100	-0.77975200
C	-0.65824600	3.48628600	2.14593200
H	1.39905400	3.27020500	-1.26743000
C	0.66899500	5.28747800	-1.14843400
C	2.18269100	4.18036400	0.51165200
H	-2.81906300	2.55985000	-0.39146300
C	-1.86350200	2.96504600	-2.26743800
C	-2.77622900	4.67849200	-0.62490200
H	0.31764700	3.18550200	2.55402600
C	-1.73862900	2.71236400	2.91797600
C	-0.81667100	5.00213800	2.39953700
H	0.26154900	6.00761100	-0.42844000
H	1.57794400	5.73942900	-1.56837700
H	-0.05099500	5.19226800	-1.96547400
H	2.50611100	3.27910400	1.03419200
H	3.05444200	4.57399600	-0.02802800
H	1.91223100	4.92996900	1.26471000
H	-2.80409300	3.00417300	-2.83536200
H	-1.17860100	3.67454200	-2.74293200
H	-1.43301700	1.96449800	-2.39554500
H	-2.12148900	5.50321900	-0.92315700
H	-3.67280400	4.73104100	-1.26002200
H	-3.09934300	4.86311700	0.40440300
H	-1.58701900	1.63074300	2.83822900
H	-1.72312600	2.97989700	3.98383300
H	-2.74427800	2.94060200	2.54102700
H	-1.80172200	5.36580800	2.08691300
H	-0.72259600	5.21344900	3.47372500
H	-0.06230300	5.60383300	1.88407900
C	-3.54639300	-1.22336600	-1.52781100
C	-4.49907500	-0.51640200	0.96723200
C	-4.25114800	0.06544100	-1.98730300
C	-5.39943100	-1.74700300	0.78877200
H	-4.60076400	-0.07971900	-3.01639700
H	-3.56964500	0.91818100	-1.96908200
C	-5.47143900	0.34917800	-1.09863000
C	-5.72055500	-1.96609400	-0.69575000
H	-4.93402600	-2.63702400	1.22105200
H	-6.34333600	-1.56367800	1.31615700
C	-2.16973500	-2.41077000	0.89563300
C	-2.52835900	-3.69760900	0.45247400
C	-1.25690800	-2.29774000	1.96135400
C	-1.98810000	-4.83094100	1.06237600
H	-3.23017900	-3.81031500	-0.36547900
C	-0.73033700	-3.43435900	2.57770500
H	-0.94669300	-1.31282600	2.30245900
C	-1.09363900	-4.70475800	2.12775200
H	-2.27099700	-5.81702700	0.70348500
H	-0.03151700	-3.32291400	3.40216900
H	-0.68208300	-5.59120300	2.60300800

P	-2.78553700	-0.82435800	0.17129500
C	-2.50072000	-1.69632600	-2.53112400
H	-2.98619200	-1.89993600	-3.49223600
H	-1.73688700	-0.92576700	-2.67556200
H	-2.00647200	-2.61162900	-2.19331200
C	-4.38861500	-0.09542700	2.42411900
H	-3.78345700	0.80814200	2.52389300
H	-5.38927100	0.10898300	2.82217300
H	-3.93234700	-0.89261900	3.02058300
C	-6.27292900	1.56275200	-1.53070000
H	-5.65349200	2.46244300	-1.47758900
H	-6.63033200	1.43004400	-2.55553700
H	-7.13289200	1.68259200	-0.86637900
C	-6.68674800	-3.10771600	-0.95180600
H	-7.63364300	-2.91746500	-0.43923100
H	-6.87331500	-3.18943000	-2.02591800
H	-6.26518000	-4.04994800	-0.58988600
O	-6.33820700	-0.77776600	-1.17948500
O	-5.07142800	0.59699400	0.25665100
O	-4.53627100	-2.26950400	-1.44320700
Pd	-0.74859200	0.58291600	0.23581000
O	4.32792300	1.37212200	1.62108000
N	4.54010600	-0.05880700	-0.15310600
C	3.95499300	-0.84600100	-1.25171600
H	2.96496100	-0.42548800	-1.44387800
H	4.56167000	-0.68273100	-2.14847600
C	5.94078200	-0.18443400	0.09202400
C	6.88951000	0.46719700	-0.71060200
C	6.40261200	-0.99110200	1.13896900
C	8.25757700	0.30963700	-0.48560600
C	7.76548700	-1.14725100	1.37935800
H	5.66750500	-1.49060200	1.76097700
C	8.69385600	-0.49924500	0.56259800
H	8.96600600	0.82691700	-1.12383400
H	8.10099400	-1.77560800	2.19917300
H	9.75920700	-0.61708000	0.73961900
C	3.85113300	-2.33247100	-0.95830100
C	4.71111600	-3.24545400	-1.57876700
C	2.88666700	-2.81541100	-0.06089200
C	4.61386300	-4.61377500	-1.31313300
H	5.46214700	-2.88260100	-2.27704200
C	2.78619600	-4.18086400	0.20515800
H	2.21131600	-2.11499600	0.42404400
C	3.65047300	-5.08439100	-0.42036800
H	5.29009200	-5.30914300	-1.80381300
H	2.02531800	-4.53895800	0.89394000
H	3.57065500	-6.14876900	-0.21451000
Cl	6.37154500	1.51900400	-2.02003500

Zero-point correction= 0.859495 (Hartree/Particle)
Thermal correction to Energy= 0.913379
Thermal correction to Enthalpy= 0.914323
Thermal correction to Gibbs Free Energy= 0.765868
Sum of electronic and zero-point Energies= -3166.831226
Sum of electronic and thermal Energies= -3166.777342
Sum of electronic and thermal Enthalpies= -3166.776398
Sum of electronic and thermal Free Energies= -3166.924853
E(RM06L) = -3169.48666363

TS_OA_Ar-N_4a

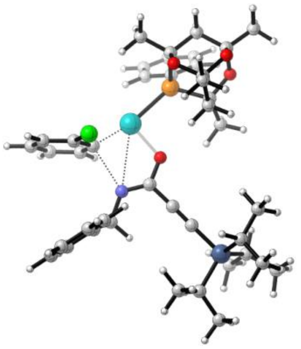

Pd	-1.52927400	1.40809900	0.35923700
C	-0.55863700	3.11239300	0.55006700
C	-0.70921400	3.68961900	1.82373600
C	-0.40160500	3.95542500	-0.56129900
C	-0.79857600	5.07778700	1.96416300
H	-0.73544400	3.04811900	2.70053800
C	-0.47491300	5.34093400	-0.42258200
C	-0.67488700	5.90047100	0.84279600
H	-0.93491300	5.51252200	2.95103500
H	-0.37500400	5.97180800	-1.30043800
H	-0.72185800	6.98077100	0.94724800
P	-3.19846500	-0.41702400	0.18731400
C	-2.52751800	-2.15939500	-0.15571900
C	-4.24764600	-0.39011000	-1.40472900
C	-4.36481000	-0.52343000	1.61073300
C	-1.70636900	-2.03728700	-1.45084400
C	-1.69442000	-2.69286400	1.00210300
O	-3.60105100	-3.10139000	-0.37583700
C	-5.20024800	-1.59079400	-1.45404200
C	-4.96542700	0.94022300	-1.58169500
O	-3.30596900	-0.48992400	-2.48678700
C	-5.01766700	-1.69551300	2.03557700
C	-4.59112300	0.66663600	2.32510300
H	-1.21356700	-3.00021200	-1.63277600
H	-0.93694100	-1.26805200	-1.34512300
C	-2.63600400	-1.74907800	-2.63754000
H	-1.33872500	-3.69951100	0.75383900
H	-0.83370100	-2.03744000	1.16321700
H	-2.28061000	-2.75363200	1.92415400
C	-4.39578500	-2.89710600	-1.55104600
H	-5.85917400	-1.60452100	-0.58093800
H	-5.82276700	-1.50660000	-2.35309800
H	-4.24529600	1.76500600	-1.59671000
H	-5.51051400	0.94217100	-2.53261600
H	-5.67934500	1.10664400	-0.76783000
C	-5.87285200	-1.66762000	3.13845800
H	-4.84709400	-2.62624100	1.50678900
C	-5.45799400	0.69424900	3.41821300
H	-4.07639400	1.57784900	2.02747600
C	-1.91892100	-1.68588400	-3.97250000
O	-3.58189600	-2.81628200	-2.71458600
C	-5.26057200	-4.13597800	-1.69703300
C	-6.10019200	-0.47505300	3.82854200
H	-6.36325200	-2.58322600	3.45863800
H	-5.62170800	1.62575500	3.95326100
H	-1.18781300	-0.87286600	-3.96490200
H	-1.40556500	-2.63173100	-4.16642400
H	-2.64743800	-1.50557300	-4.76765500
H	-5.87073900	-4.06102400	-2.60137700
H	-4.61836900	-5.01748900	-1.77269000
H	-5.91693700	-4.24606800	-0.82875800
H	-6.76927300	-0.45866200	4.68468100
Cl	-0.14725100	3.27840800	-2.16653400
N	1.53015200	1.83896600	0.80492700

C	1.29491600	0.58922900	0.56489400
C	2.84453600	2.34110400	1.15917600
C	2.33723600	-0.41969100	0.56754800
O	0.11682900	0.09988000	0.29605500
H	3.50666700	1.52195500	1.48391200
H	2.72296300	3.00669000	2.02360200
C	3.53995800	3.11765200	0.04954400
C	3.23592400	-1.24625400	0.56251900
C	4.23281700	4.29722500	0.34706600
C	3.55137600	2.64819700	-1.27023700
Si	4.58817200	-2.51126500	0.52128500
C	4.93145400	4.98884100	-0.64459100
H	4.22471900	4.67839500	1.36634000
C	4.24611200	3.33819800	-2.26468700
H	3.00377000	1.74451700	-1.52014500
C	4.17567700	-3.72879000	1.94633500
C	4.46545800	-3.30481500	-1.22180200
C	6.21326800	-1.53089700	0.79995900
C	4.94082900	4.51002900	-1.95559700
H	5.46177400	5.90420300	-0.39357400
H	4.24115000	2.96158700	-3.28448400
H	3.11510400	-3.97429800	1.78315900
C	4.95840400	-5.05649300	1.93453800
C	4.27639200	-3.04252900	3.32337700
H	4.43246400	-2.43953800	-1.90153600
C	3.14263900	-4.07577200	-1.40302900
C	5.66920200	-4.16984300	-1.64431900
H	5.99811300	-0.91207100	1.68490600
C	6.50304000	-0.56717700	-0.36859800
C	7.45500100	-2.38194400	1.13147000
H	5.47984800	5.04780300	-2.73132500
H	6.03683800	-4.90135900	2.05534100
H	4.63146700	-5.70137900	2.76197800
H	4.80741700	-5.61765400	1.00592800
H	3.67344300	-2.12853500	3.36837100
H	3.92389900	-3.71305100	4.11903600
H	5.31193200	-2.77145800	3.56482000
H	3.03231900	-4.41952700	-2.44067000
H	3.10304400	-4.96599600	-0.76251800
H	2.27283100	-3.45360800	-1.16393300
H	5.79842900	-5.04356600	-0.99484000
H	5.52951100	-4.54712800	-2.66681100
H	6.60838800	-3.60626300	-1.63127100
H	5.65748600	0.09891000	-0.57345800
H	7.37267900	0.06478100	-0.14303900
H	6.73171700	-1.11188300	-1.29343500
H	7.72213700	-3.05952800	0.31206800
H	8.32521500	-1.73551600	1.31038600
H	7.31200500	-2.99110200	2.03070600

Zero-point correction= 0.855990 (Hartree/Particle)
Thermal correction to Energy= 0.910529
Thermal correction to Enthalpy= 0.911473
Thermal correction to Gibbs Free Energy= 0.759731
Sum of electronic and zero-point Energies= -3166.794855
Sum of electronic and thermal Energies= -3166.740316
Sum of electronic and thermal Enthalpies= -3166.739372
Sum of electronic and thermal Free Energies= -3166.891115
E(RM06L) = -3169.43990930

TS_OA_N-Bn_4a

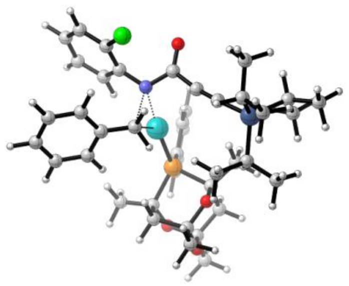

C	-2.44435600	4.21023900	0.83216600
C	-2.38486500	5.59400700	0.99112800
C	-1.28070700	3.40955000	0.89634500
C	-1.15648000	6.22499800	1.18153400
H	-3.30394400	6.16921700	0.95211400
C	-0.05651600	4.08040400	1.07706200
C	0.01260100	5.46305500	1.21306900
H	-1.11861100	7.30403700	1.30046200
H	0.84093300	3.46886400	1.12418600
H	0.97691000	5.94183200	1.35785700
C	-2.10974000	1.24272600	1.54789100
C	-2.38480100	-0.11095300	1.07041100
C	-2.79515300	-1.21555800	0.74487600
Si	-3.50887200	-2.86594800	0.29478100
C	-2.33575800	-3.60545900	-1.03230700
H	-1.32520800	-3.45273100	-0.62480100
C	-3.56139900	-3.85695500	1.93686400
H	-4.04867000	-3.16418200	2.63973100
C	-5.25527900	-2.46120300	-0.39426100
H	-5.06843700	-1.67215700	-1.13907500
C	-2.50168000	-5.11541300	-1.29367500
H	-3.50026300	-5.36305300	-1.67307400
H	-1.77740300	-5.45619000	-2.04651100
H	-2.33489000	-5.71107700	-0.38963900
C	-2.40907300	-2.81112500	-2.35170400
H	-2.23579400	-1.74026800	-2.19276900
H	-1.65051600	-3.16881700	-3.06127800
H	-3.38523300	-2.92079400	-2.84034200
C	-2.14938000	-4.13879300	2.48658700
H	-2.20586600	-4.61268100	3.47611300
H	-1.58797200	-4.81932200	1.83366400
H	-1.56434800	-3.21877600	2.59352700
C	-4.40661000	-5.14578300	1.90703500
H	-4.01345100	-5.88281800	1.19709400
H	-4.41069300	-5.62250800	2.89696700
H	-5.44986700	-4.94996800	1.63703500
C	-6.16863300	-1.85534800	0.69030600
H	-5.70366700	-0.99405900	1.18233700
H	-7.11846700	-1.51788000	0.25348000
H	-6.41436400	-2.59041500	1.46708700
C	-5.96381800	-3.61839000	-1.12591100
H	-6.16964200	-4.46325200	-0.45857500
H	-6.93036000	-3.28278100	-1.52651900
H	-5.37673900	-3.99922100	-1.96879600
N	-1.28802400	2.01193600	0.73167500
C	-0.93965100	2.58923700	-2.18549000
C	0.35209700	2.75800300	-2.73102100
C	-1.92143100	3.55744800	-2.48350900
C	0.64359300	3.83687600	-3.55551700
H	1.12150500	2.03260600	-2.48437900
C	-1.62668400	4.63944600	-3.31026900
H	-2.91952200	3.44428900	-2.07261800
C	-0.34596900	4.78433200	-3.84710700
H	1.64166900	3.94762200	-3.97067300
H	-2.39728300	5.37212300	-3.53302800
H	-0.11563200	5.63158900	-4.48728700

O	-2.59750600	1.63346500	2.60308000
Cl	-4.02381000	3.50361500	0.51590700
C	-1.28116200	1.41833600	-1.40012000
H	-2.32900500	1.24009300	-1.20275300
H	-0.71948200	0.50628000	-1.59229800
Pd	0.49886100	0.93173500	0.35386900
P	2.48000800	-0.24441100	0.30825300
C	3.63379200	-0.21396600	-1.20729800
C	2.31935800	-2.14797100	0.24950300
C	3.52713300	0.13358800	1.78820300
C	2.79793400	-0.74724000	-2.38506800
C	4.19876300	1.17528900	-1.47959700
O	4.77527500	-1.08451100	-1.04197300
C	3.69488800	-2.82437800	0.30239300
C	1.37581600	-2.65690600	1.32752300
O	1.69786100	-2.45753900	-1.01685500
C	4.92957400	0.03081600	1.84577800
C	2.84159500	0.57673400	2.93427500
H	3.38156700	-0.63574800	-3.30685400
H	1.86628900	-0.18448700	-2.49114300
C	2.49467500	-2.24161200	-2.18385500
H	4.82992100	1.14567300	-2.37522600
H	3.38791100	1.89294100	-1.63879700
H	4.80637500	1.52664200	-0.64072400
C	4.50101400	-2.48801600	-0.96008100
H	4.24192100	-2.52642400	1.20118000
H	3.54993000	-3.91113400	0.33354000
H	0.39959500	-2.17086300	1.24056200
H	1.24494000	-3.74046200	1.22363100
H	1.78295000	-2.44838700	2.32284900
C	5.61669600	0.36275100	3.01526900
H	5.48183400	-0.30463900	0.97583300
C	3.53023000	0.89079300	4.10665600
H	1.75984600	0.68627600	2.89781100
C	1.73760100	-2.86775100	-3.34037400
O	3.74385700	-2.92206200	-2.08549000
C	5.84624700	-3.18697100	-1.03231000
C	4.92161300	0.78785200	4.14900900
H	6.70081300	0.28595300	3.03850100
H	2.97814700	1.22600300	4.98051600
H	0.76365700	-2.38475000	-3.45870000
H	2.30992800	-2.76022600	-4.26599700
H	1.58553600	-3.93132800	-3.13770500
H	5.70459900	-4.27109400	-1.01620900
H	6.35049000	-2.90728700	-1.96123000
H	6.47188100	-2.89424800	-0.18407100
H	5.46123600	1.04167900	5.05759800

Zero-point correction= 0.855460 (Hartree/Particle)
Thermal correction to Energy= 0.910110
Thermal correction to Enthalpy= 0.911055
Thermal correction to Gibbs Free Energy= 0.762771
Sum of electronic and zero-point Energies= -3166.799212
Sum of electronic and thermal Energies= -3166.744561
Sum of electronic and thermal Enthalpies= -3166.743617
Sum of electronic and thermal Free Energies= -3166.891900
E(RM06L) = -3169.45873808

TS_OA_N-CO_4a

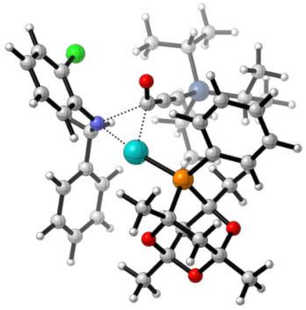

Pd	0.63955600	1.12789000	-0.19856500
P	2.35264200	-0.39833300	-0.64683700
C	2.46462000	-1.85808200	0.56314000
C	4.03516800	0.31538000	-0.10602200
C	2.53970400	-1.04398000	-2.35782700
C	2.45198800	-1.23052800	1.96893000
C	1.33634300	-2.86206200	0.36441300
O	3.69745800	-2.59134400	0.40171900
C	5.15028700	-0.72826600	-0.23939600
C	4.35951900	1.61205000	-0.83348600
O	3.86679100	0.65634900	1.28454100
C	3.18428700	-2.25194000	-2.68602800
C	1.99254100	-0.26240000	-3.39094600
H	2.40749300	-2.03879200	2.70867800
H	1.57786400	-0.58573000	2.10507700
C	3.74957000	-0.44006400	2.20247700
H	1.42802900	-3.66294600	1.10689500
H	0.36576200	-2.37127900	0.48615300
H	1.37590000	-3.30945100	-0.63304500
C	4.90425300	-1.89121500	0.73270800
H	5.22274600	-1.09100300	-1.26882400
H	6.10439200	-0.25728500	0.02633600
H	3.56143000	2.34645800	-0.68580000
H	5.29508400	2.02898700	-0.44328700
H	4.47489000	1.43265400	-1.90769300
C	3.28314600	-2.65679800	-4.01751300
H	3.59733100	-2.87445600	-1.90051200
C	2.10570200	-0.66888000	-4.72179600
H	1.45397000	0.65000500	-3.15437600
C	3.85567900	0.15766400	3.59195500
O	4.83777100	-1.34931100	2.04637300
C	6.00921900	-2.93156100	0.73444900
C	2.75070900	-1.86507400	-5.03789500
H	3.77756400	-3.59465100	-4.25707800
H	1.67457400	-0.05400200	-5.50679900
H	3.03354500	0.85756500	3.76417800
H	3.81642500	-0.63702000	4.34236100
H	4.80756900	0.68765400	3.68493600
H	6.95824200	-2.46742800	1.01653600
H	5.76705300	-3.71492700	1.45756400
H	6.10986900	-3.38051600	-0.25800400
H	2.83160000	-2.18436400	-6.07357500
C	-0.93439900	0.77553300	-1.42895200
C	-1.80460600	-0.21789400	-0.88460900
O	-0.91003700	1.18375700	-2.56605900
C	-2.54748500	-1.07944600	-0.43285800
Si	-3.75869000	-2.38478800	0.10144300
C	-3.85076600	-2.25669100	2.01316700
C	-5.39264200	-1.91522400	-0.78410200
C	-2.99371100	-4.03330600	-0.51572600
H	-3.99747700	-1.18160000	2.20072100
C	-5.03522000	-2.99701100	2.66558800
C	-2.51925000	-2.66008900	2.67737300
H	-5.08717700	-1.76015600	-1.82998000
C	-5.95339300	-0.57336500	-0.27110200

C	-6.48210300	-3.00603200	-0.78301700
H	-1.95531800	-4.00092400	-0.15103300
C	-2.93466600	-4.09050000	-2.05556500
C	-3.63766500	-5.31133400	0.05777200
H	-4.99152000	-4.07877800	2.49331000
H	-5.02962800	-2.84358400	3.75338200
H	-6.00106400	-2.64088400	2.29200000
H	-1.67269400	-2.09073200	2.27659400
H	-2.55511900	-2.48111900	3.76076200
H	-2.30249800	-3.72607300	2.53448100
H	-6.82493900	-0.26754700	-0.86515800
H	-6.28354400	-0.64456400	0.77306400
H	-5.21232700	0.23111900	-0.33508000
H	-6.82423200	-3.24860600	0.22978700
H	-7.36140700	-2.66537700	-1.34640600
H	-6.13838600	-3.93630200	-1.24839900
H	-2.42705700	-3.21676900	-2.47914700
H	-2.39227000	-4.98543900	-2.38934800
H	-3.93835200	-4.13826100	-2.49602900
H	-4.69092400	-5.40711300	-0.23025900
H	-3.11854400	-6.20284600	-0.31995200
H	-3.58958000	-5.34588000	1.15161800
N	-1.02670400	2.40880400	0.03390700
C	-1.18674900	3.55116100	-0.74885200
C	-1.82547300	2.21300400	1.24194500
C	-2.42656600	4.05081400	-1.22362700
C	-0.04249800	4.26240300	-1.17920200
H	-2.41155500	1.28578700	1.17467500
H	-2.55434700	3.02651300	1.33683900

C	-0.98662700	2.16937600	2.51351800
C	-2.51535700	5.20529400	-2.00042100
Cl	-3.93621400	3.18340500	-0.91912700
C	-0.11790000	5.39821100	-1.97737200
H	0.92187300	3.88984500	-0.84399100
C	-0.03272200	3.16271100	2.78403600
C	-1.18221500	1.15799800	3.46187600
C	-1.36155800	5.88878500	-2.37978000
H	-3.49294700	5.54462000	-2.32749100
H	0.79496500	5.90938300	-2.27184500
C	0.70034400	3.14525600	3.97059500
H	0.13024600	3.95295700	2.05611000
C	-0.44952700	1.13512000	4.65232200
H	-1.91946700	0.38211000	3.26722700
H	-1.43784200	6.78240600	-2.99231800
C	0.49458700	2.12996800	4.91108000
H	1.42914300	3.92795900	4.16632300
H	-0.61764600	0.34091800	5.37570700
H	1.06204000	2.11946900	5.83819000

Zero-point correction= 0.856268 (Hartree/Particle)
Thermal correction to Energy= 0.910719
Thermal correction to Enthalpy= 0.911663
Thermal correction to Gibbs Free Energy= 0.762730
Sum of electronic and zero-point Energies= -3166.817543
Sum of electronic and thermal Energies= -3166.763093
Sum of electronic and thermal Enthalpies= -3166.762148
Sum of electronic and thermal Free Energies= -3166.911082
E(RM06L) = -3169.47342882

Literature

1. T. Truong and O. Daugulis, *Org. Lett.*, 2012, **14**, 5964-5967.
2. T. Iwai, T. Fujihara, J. Terao and Y. Tsuji, *J. Am. Chem. Soc.*, 2010, **132**, 9602-9603.
3. C. Y. Legault, CYLview, Version 1.0b; Université de Sherbrooke, 2009 (http://www.cylview.org).
4. S. Kozuch and S. Shaik, *Acc. Chem. Res.*, 2011, **44**, 101-110.
5. J. P. Foster and F. Weinhold, *J. Am. Chem. Soc.*, 1980, **102**, 7211-7218.
6. A. E. Reed, R. B. Weinstock and F. Weinhold, *J. Chem. Phys.*, 1985, **83**, 735-746.
7. A. E. Reed and F. Weinhold, *J. Chem. Phys.*, 1985, **83**, 1736-1740.
8. A. E. Reed, L. A. Curtiss and F. Weinhold, *Chem. Rev.*, 1988, **88**, 899-926.
9. R. F. W. Bader, *Atoms in Molecules: A Quantum Theory*, Oxford University Press, New York, 1990.
10. T. Lu and F. Chen, *J. Comput. Chem.*, 2012, **33**, 580-592.
11. I. Mayer, *Chem. Phys. Lett.*, 1983, **97**, 270-274.
12. I. Mayer, *Chem. Phys. Lett.*, 2012, **544**, 83-86.
